# Synthesis and Evaluation of Diguanosine Cap Analogs Modified at the C8-Position by Suzuki–Miyaura Cross-Coupling: Discovery of 7-Methylguanosine-Based Molecular Rotors

**DOI:** 10.1021/acs.joc.3c00126

**Published:** 2023-05-20

**Authors:** Blazej A. Wojtczak, Marcelina Bednarczyk, Pawel J. Sikorski, Anna Wojtczak, Piotr Surynt, Joanna Kowalska, Jacek Jemielity

**Affiliations:** † Centre of New Technologies, University of Warsaw; S. Banacha 2c, 02-097 Warsaw, Poland; ‡ Faculty of Physics, University of Warsaw; L. Pasteura 5, 02-093, Warsaw, Poland

## Abstract

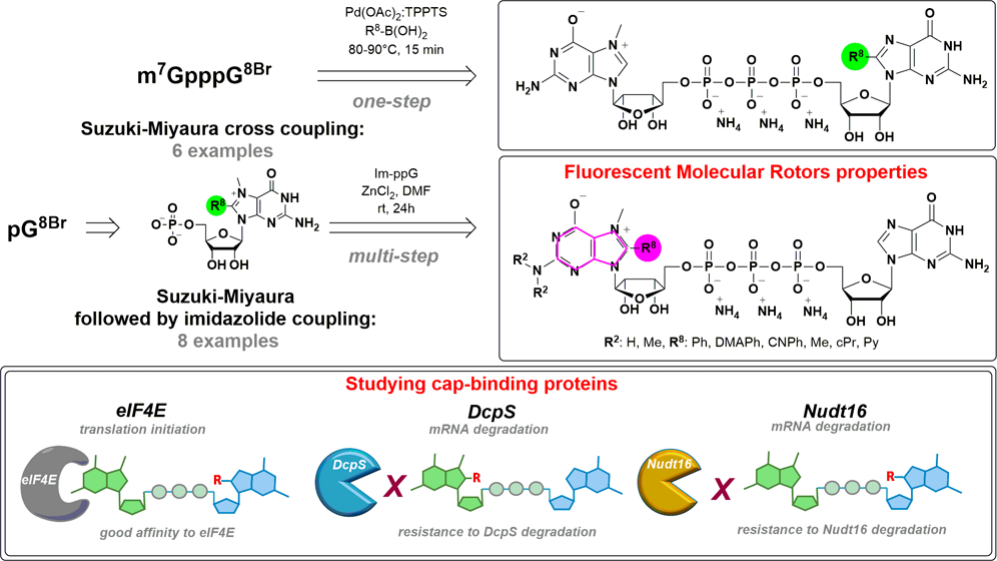

Chemical modifications of the mRNA cap structure can enhance the stability, translational properties, and half-life of mRNAs, thereby altering the therapeutic properties of synthetic mRNA. However, cap structure modification is challenging because of the instability of the 5′-5′-triphosphate bridge and N7-methylguanosine. The Suzuki–Miyaura cross-coupling reaction between boronic acid and halogen compound is a mild, convenient, and potentially applicable approach for modifying biomolecules. Herein, we describe two methods to synthesize C8-modified cap structures using the Suzuki–Miyaura cross-coupling reaction. Both methods employed phosphorimidazolide chemistry to form the 5′,5′-triphosphate bridge. However, in the first method, the introduction of the modification *via* the Suzuki–Miyaura cross-coupling reaction at the C8 position occurs postsynthetically, at the dinucleotide level, whereas in the second method, the modification was introduced at the level of the nucleoside 5′-monophosphate, and later, the triphosphate bridge was formed. Both methods were successfully applied to incorporate six different groups (methyl, cyclopropyl, phenyl, 4-dimethylaminophenyl, 4-cyanophenyl, and 1-pyrene) into either the m^7^G or G moieties of the cap structure. Aromatic substituents at the C8-position of guanosine form a push–pull system that exhibits environment-sensitive fluorescence. We demonstrated that this phenomenon can be harnessed to study the interaction with cap-binding proteins, *e.g.*, eIF4E, DcpS, Nudt16, and snurportin.

## Introduction

A unique feature of eukaryotic mRNA is the cap structure at the 5′-end. The cap consists of positively charged N7-methylguanosine (m^7^G or m^7^Guo) connected to the first transcribed nucleotide by an unusual 5′-5′-triphosphate bridge. The cap structure is recognized by specific cap-binding proteins, including eukaryotic initiation factor 4E (eIF4E) that is involved in the initiation of translation.^1^ The cap also provides protection against enzymatic degradation.^2^ Elevated levels of eIF4E have been found in some types of cancer cells,^3^ and the activity of a decapping scavenger enzyme (DcpS) is correlated with spinal muscular atrophy.^4^ Therefore, synthetic cap analogs have important potential uses such as inhibitors of cap-dependent translation,^5,6^ inhibitors of decapping enzymes,^7^ and fluorescent probes to study interactions with cap-specific proteins.^8^ Synthetic cap analogs are also used to modify the 5′-end for developing effective therapeutic mRNAs.^5,9^ Several chemical and enzymatic methods are available for the postsynthetic modification of cap analogs including click chemistry,^10^ carbamate chemistry, and methyltransferase assays.^11^ However, because of the fragile nature of N7-methylguanosine, these strategies usually involve multistep reactions. The majority of modifications occur on electron-rich atoms (*e.g.*, O6, N1, N7, and 2′-*O*) involved in cap binding, and thus, they may disturb the mRNA–protein interactions to some extent. Therefore, the development of postsynthetic, bio-orthogonal methods for cap structure modification at natural nucleobase positions is challenging, especially in the context of affinity to cap-binding proteins. Nonetheless, N7-methylguanosine is a very interesting site for modifying the cap structure because it is essential for interaction with most proteins involved in mRNA metabolism. Thus, it may be an excellent site for modifications to differentiate them and provide highly selective molecular tools.

The palladium-catalyzed Suzuki–Miyaura cross-coupling reaction^12^ is a mild, efficient, and convenient method that meets the above-mentioned criteria in some cases. The Suzuki–Miyaura reaction has been successfully used in aqueous media for modifying nucleotides,^13−15^ cyclic dinucleotides,^16^ and oligonucleotides^17−19^ and for post-transcriptional labeling of RNA.^17^ However, there have been no examples of modified cap analogs, most likely because of their limited chemical stability. The positively charged N7-methylguanosine increases the rate of depurination reaction,^20^ and it undergoes an imidazolium ring-opening side reaction^21^ at high pH. In addition, decomposition of the 5′-5′-triphosphate bridge in alkaline or acidic environment limits its use for modification. Here, we report the postsynthetic modification of mRNA cap analogs using Suzuki–Miyaura cross-coupling reaction. Up to six different groups were incorporated using the palladium cross-coupling method at the C8-position of either the m^7^G or G residue. Interestingly, some of these substituents significantly affect fluorescence properties of the modified nucleotides, which may have applications in designing molecular probes. The incorporation of both electron-donating and electron-accepting aromatic substituents yields highly emissive π-conjugated molecular rotors. In general, molecular rotors are characterized by low or no fluorescence because their intramolecular rotation effectively dissipates the excitation energy.^22^ In the current cases, the photoexcited molecular rotor forms a twisted intramolecular charge transfer (TICT) state, which can return to the ground state by either emitting fluorescence or nonradiative relaxation. The TICT state depends on the local environment, specifically on the microviscosity and polarity of the solvent. Fluorescent probes based on molecular rotors are versatile tools in fluorescence-based techniques.^23,24^ In the context of nucleotides, fluorescent probes have been applied in biological assays including hybridization,^24,25^ microenvironment monitoring,^26^ and ligand–protein interaction studies.^27^ However, only a few reported studies used molecular rotors based on modified nucleotides to examine interactions with proteins.^28^ Recent research showed that nucleotides modified with molecular rotors are sensitive to the environment and/or secondary DNA structures^29^ and that their fluorescence intensity depends on the pH and viscosity. This phenomenon is very attractive for *in vitro* and *in vivo* molecular biology techniques, especially fluorescence sensing and imaging.

Here, we report the synthesis of 19 cap analogs (Table 1) together with their photophysical and biochemical properties. We present molecular tools with interesting fluorescent properties including molecular rotors and study their biological properties in relation to three cap-dependent proteins: a translation initiation factor 4E (eIF4E) and two decapping enzymes (DcpS and Nudt16).

**Table 1:**
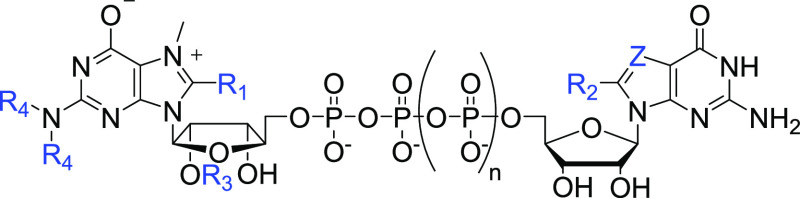

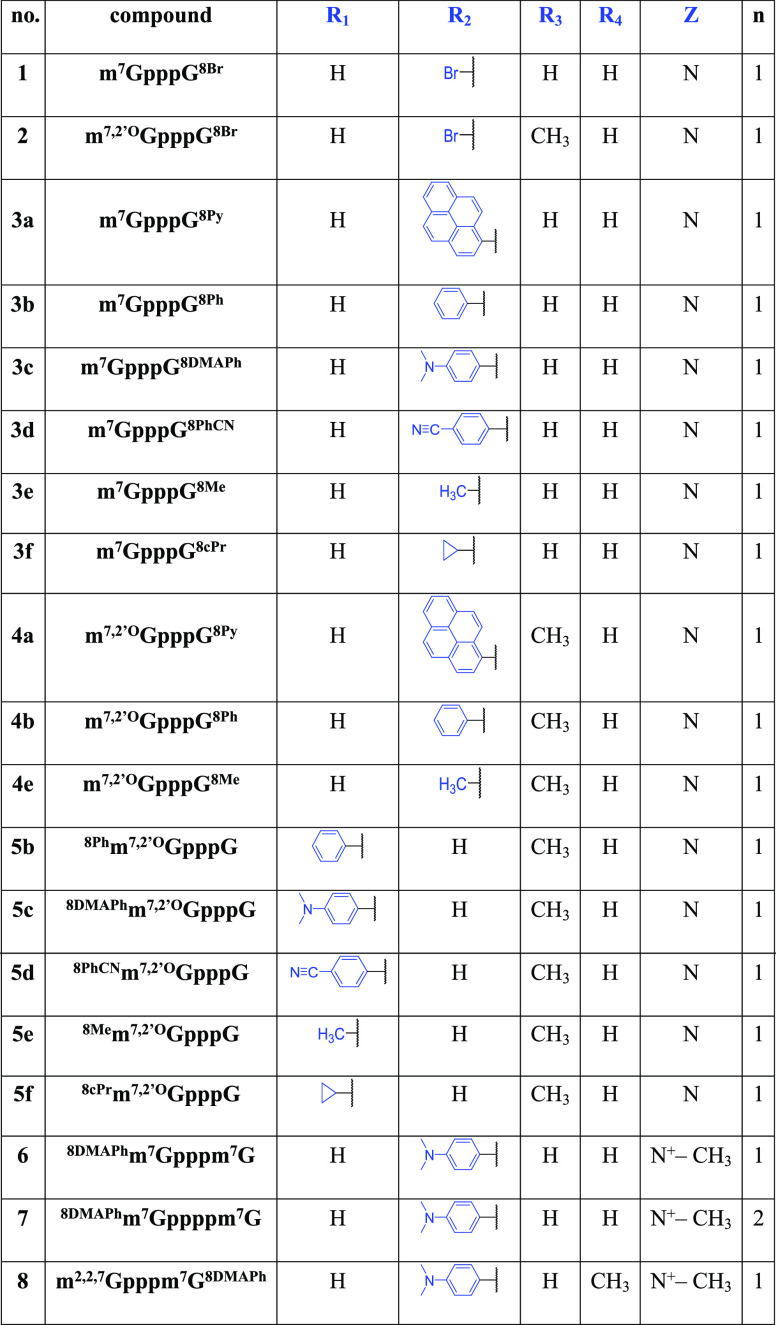
Structures of C8-Modified Cap Analogs Synthesized in This Study

## Results and Discussion

### Synthesis of Dinucleotide Cap Analogs Modified at the C8-Position

**Scheme 1:**
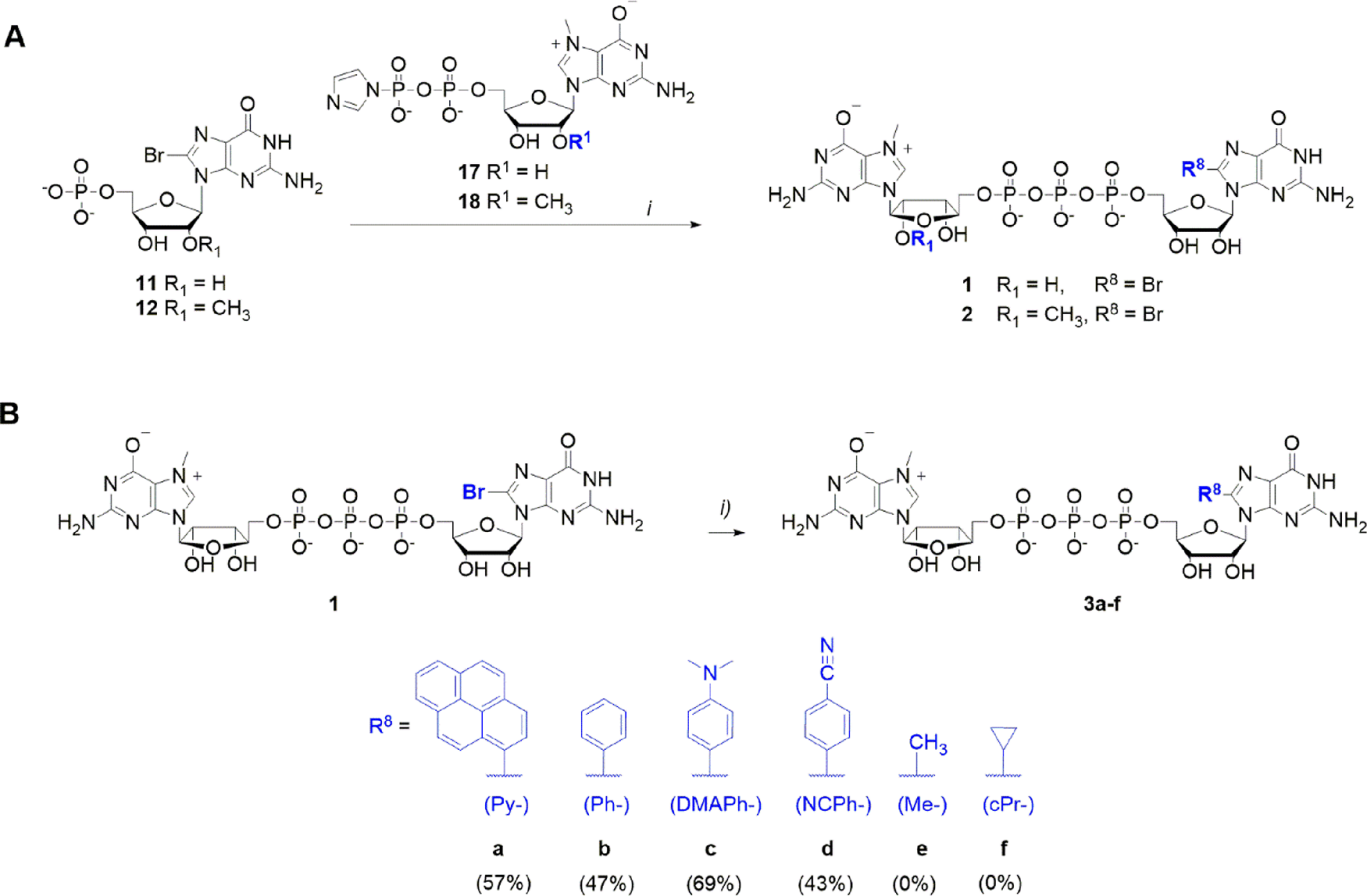
Synthesis and Postsynthetic Cap Modification (A) Synthesis of m^7^GpppG^8Br^ and m^2^′^-*O*,7^GpppG^8Br^. Reagents and conditions: (i) ZnCl_2_, DMF. (B) Postsynthetic cap modification by Suzuki–Miyaura cross-coupling reaction. Reagents and conditions: (i) Pd(OAc)_2_/TPPTS complex, NaHCO_3_ buffer (100 mM, pH 8.5), R^8^-B(OH)_2_, 80–90 °C, 15 min.

As key precursors for functionalizing the C8-position by aqueous Suzuki–Miyaura cross-coupling reactions, 8-bromoguanosine 5′-monophosphate (^8Br^GMP, **11**, Supporting Information (SI)) and 8-bromo-2′-*O*-methylguanosine 5′-monophosphate (^8Br^m^2^′^*O*^GMP, **12**, SI) were prepared from either commercially available guanosine 5′-monophosphate (GMP, **9,**SI) followed by conversion to triethylammonium salt or the m^2^′^*O*^GMP (**10,**SI) derivative obtained by the Yoshikawa procedure.^30,31^ Subsequent treatment of **9** with saturated bromine water^32^ in sodium acetate buffer (pH 4.0) afforded the product in 62% yield after ion-exchange chromatography. This derivative required repeated purification steps to remove sodium acetate contamination. Therefore, for derivative **11**, we decided to use *N*-bromosuccinimide (NBS) as the bromination agent instead. After workup and purification, **12** was isolated in 77% yield (Scheme S1, SI). Then, starting from N7-methylguanosine 5′-monophosphate-*P*-imidazolide (m^7^GDP-Im, **16**, *SI*) and 8-bromo-5′-monophosphate (**11**), we performed a coupling reaction in the presence of ZnCl_2_ as a catalyst to obtain cap analog **1** bearing a bromine at the C8-position of guanosine (m^7^GpppG^8Br^) as depicted in Scheme 1A. Cap analog **1** was then used as a model compound to determine the optimum conditions for postsynthetic cap modification via Suzuki–Miyaura cross-coupling.

**Scheme 2:**
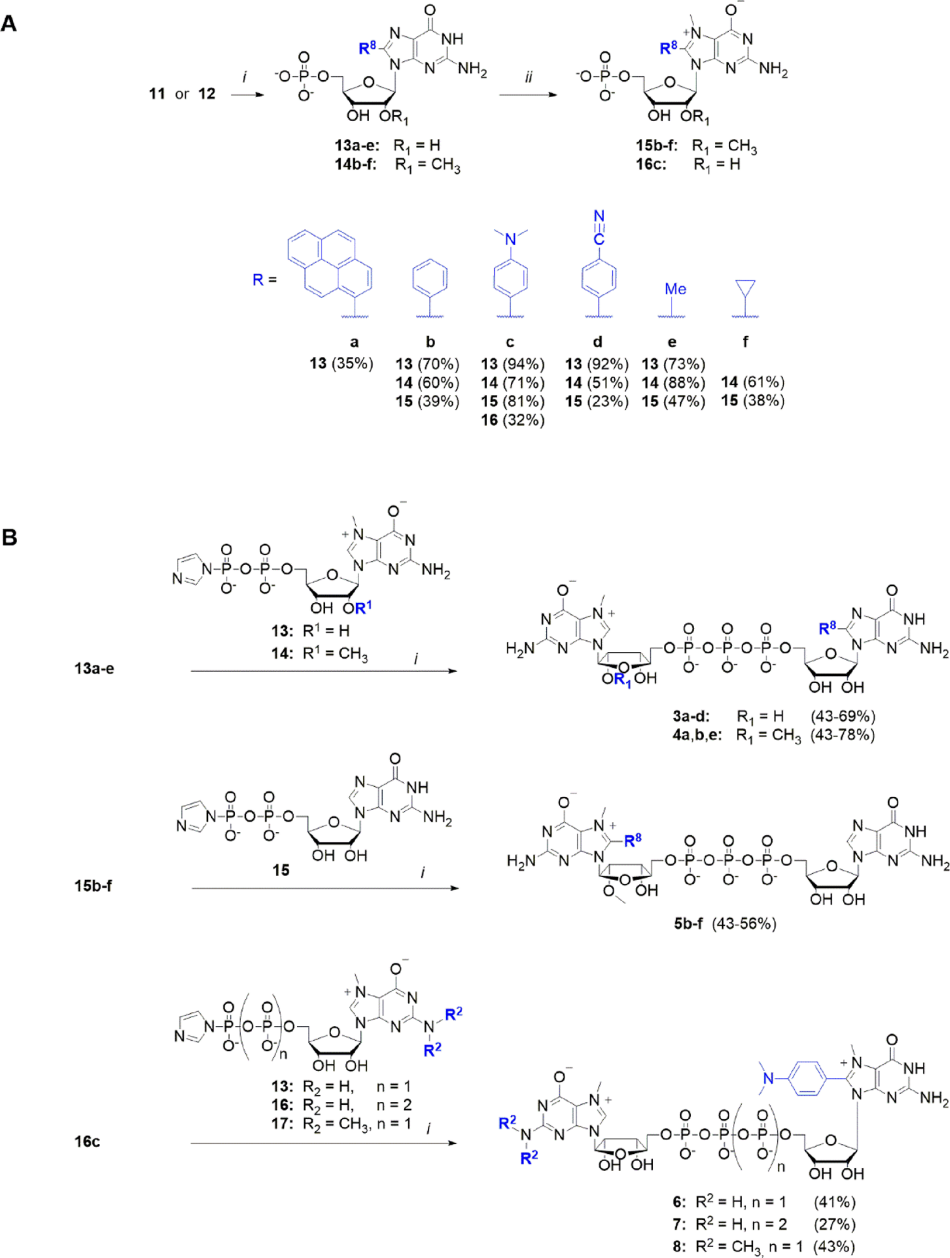
Synthesis of 8-Substituted Monophosphates and 8-Substituted Cap Analogs (A) Synthesis of 8-substituted monophosphates. Reagents and conditions: (i) Pd(OAc)_2_/TPPTS complex, Cs_2_CO_3_, R-B(OH)_2_, 90–95 °C, 15–60 min; (ii) MeI, DMSO/DMF (1:1). (B) Synthesis of 8-substituted cap analogs *via* phosphorimidazolide intermediates. Reagents and conditions: (i) ZnCl_2_, DMF, 24 h.

Having the 8-bromo-modified cap analog **1** in hand, we used Suzuki–Miyaura cross-coupling reaction to obtain six different compounds (**3a**–**f**, Scheme 1B). Recently, the Suzuki–Miyaura cross-coupling reaction was used for the postsynthetic modification of halogenated oligonucleotides,^17,18,33,34^ polypeptides,^35^ and proteins.^36^ However, to our knowledge, this reaction has not been reported for mRNA cap analogs. Although an aqueous version of the Suzuki–Miyaura reaction can be performed without protecting groups,^13^ the general problem with direct nucleotide modification is the presence of a triphosphate chain at elevated temperatures. Nonetheless, efficient coupling occurred at pH > 8, and the addition of a base stabilized the triphosphate moiety.^37^ An additional challenge in the case of cap analogs is the instability of N7-methylguanosine under alkaline conditions, and therefore, it is crucial to determine the optimal conditions for complete reaction. First, we studied the thermal stability of **1** at pH 7.0, 8.0, and 8.5 and 60–90 °C within 15 min (Figure S1, SI). Cap analog **1** was stable at pH 7.0 in the whole temperature range, but fast decomposition was observed at pH > 7.0 and above 70 °C. On the other hand, at pH < 8 and 60 °C, no product was observed in the further cross-coupling reaction with **1**. Therefore, we chose 70 °C and pH 8.0–8.5 as a compromise between substrate stability and product formation for the Suzuki–Miyaura reaction using phenylboronic acid as the starting material. The reaction product of phenylboronic acid with guanosine, 8-phenylguanosine (^8Ph^G), emitted strong fluorescence (λ_em_ = 400 nm),^34^ allowing easy monitoring of product formation by reverse-phase high-performance liquid chromatography (RP-HPLC) equipped with a UV–vis and fluorescence detector. First, we subjected **1** to Suzuki–Miyaura cross-coupling reaction using conditions published by the Hocek group^38^ for 8-bromo-5′-triphosphate. Pd(OAc)_2_ was used as the catalyst, Cs_2_CO_3_ was used as the base, and the temperature was >90 °C. However, imidazole ring-opening of N7-methylguanosine was observed under these conditions as indicated by liquid chromatography–mass spectrometry (LC–MS). Because the side reaction was faster under basic conditions, next we decreased the amount of Cs_2_CO_3_. When Cs_2_CO_3_ was reduced from 5 to 3 equiv and boronic acid was increased from 1.2 to 2 equiv (Figures S2 and S3, SI), cap analog **3b** (m^7^GpppG^8Ph^) containing 8-phenylguanosine was obtained with >95% conversion, as indicated by analytical RP-HPLC. This promising result prompted us to attempt the Suzuki–Miyaura cross-coupling of **1** under conditions similar to those of other aromatic and nonaromatic boronic acids (Figures S4–S14, SI). Unfortunately, ring-opening of m^7^G was observed in most of these cases (Figures S12 and S14, SI). In Suzuki–Miyaura reactions, the addition of a base is necessary to convert Pd(II) into the active Pd(0) catalyst,^39^ and the pH should be high during the reaction to ensure an adequate concentration of the active catalyst, whereas for our purpose, the pH should be kept as low as possible to avoid the m^7^G imidazole ring-opening side reaction. When the reaction was conducted at pH 7.6, no trace of product was detected by RP-HPLC, and at above pH 8.5, both product formation and ring-opening side reactions were observed. Because a pH change can also be caused by excess boronic acid, to maintain a constant pH, we performed the Suzuki–Miyaura reaction at pH ≤ 8.5 in NaHCO_3_ buffer. Under these conditions, cap analog **3b** was obtained in quantitative yield (RP-HPLC), and the reaction was complete within 15 min (Figures S6 and S7, SI). Considering that aromatic boronic acids have p*K*_a_ ≈ 9, we observed that substituted boronic acids, which are well soluble in water, reacted quickly and efficiently at only 2 equiv under Suzuki–Miyaura reaction conditions. When the boronic acid bears an electron-donating group (4-dimethylaminophenyl (DMAPh)) or an electron-withdrawing group (4-cyanophenyl (PhCN)), we used 10- or 20-fold molar excess of boronic acid and a higher palladium catalyst loading, respectively, because otherwise the reaction was very slow or did not occur at all (Figures S8–S11, SI). This is consistent with earlier reports^39^ that aryl boronic acids, which involve both steric effects and electron-withdrawing substituents, resulted in lower yields. For less reactive boronic acids with p*K*_a_ ≈ 10, such as methyl- (Me) and cyclopropyl- (cPr) boronic acid, three peaks were observed in the crude RP-HPLC chromatograms corresponding to unreacted **1**, the imidazole ring-opening byproduct, and the product, as confirmed by HPLC and MS (Figures S12–S14, SI). Even using 20 equiv of boronic acid and microwave irradiation did not produce the desired results, as we mainly observed the product of the imidazole ring-opening side reaction. This suggests that postsynthetic modification *via* the Suzuki–Miyaura method is not the best solution for less reactive boronic acids. Therefore, to obtain cap analogs modified with alkyl substituents at the C8-position of guanosine (G or Guo), we performed C8-modification at an earlier stage of synthesis followed by final dinucleotide production by forming a triphosphate bridge *via* standard P-imidazolide activation in the presence of ZnCl_2_. In other words, cap analogs bearing aromatic and aliphatic substituents at the C8-position were synthesized by carrying out the Suzuki–Miyaura reaction prior to the standard imidazolide chemistry (Scheme 2A,B). For this purpose, we modified nucleoside monophosphates according to a protocol recently published by Shaughnessy.^40^ The author applied a complex formed between Pd(OAc)_2_ and triphenylphosphine-3,3′,3″-trisulfonic acid trisodium salt (TPPTS) in the presence of an inorganic base. When **11** and **12** were treated with different boronic acids in 2- to 10-fold excess, cesium carbonate, and the Pd(OAc)_2_/TPPTS complex, we obtained nucleotides **13a**–**e** and **14b**–**f** in moderate to very good yields (35–95%). However, the less reactive methyl and cyclopropyl boronic acids required longer reaction times, higher temperatures, and 10-fold excess of boronic acids to generate products at acceptable yields. Under these conditions, all cross-coupling reactions afforded the targeted 8-substituted guanosine 5′-monophosphates, which were isolated in high yields after RP-HPLC purification. Next, derivatives **14b**–**f** and **13c** were treated with methyl iodide in a DMSO/DMF mixture (1:1, v/v) to afford the corresponding 8-substituted N7-methyl-5′-monophosphates **15b**–**f** and **16c**. Finally, the obtained compounds (**11**, **12**, **13a**–**13e**, and **15b**–**f**) were coupled with various di- or triphosphate imidazolides [m^7^GDP-Im (**17**), m^7,2^′^*O*^GDP-Im (**18**), GDP-Im (**19**), GTP-Im (**20)**, or m^2,2,7^GDP-Im (**21**)] in the presence of ZnCl_2_ as catalyst to give the corresponding 5′-cap analogs (**1**, **2**, **3a**–**d**, **4a**, **b**, **e**, **5b**–**f**, and **6**–**8**).

All synthesized compounds were characterized by ^1^H and ^31^P NMR spectroscopy, UV spectroscopy, and electrospray ionization (ESI) mass spectrometry (for details, see the Experimental Section and SI). Before further study, we characterized the chemical stability of all cap analogs at pH 3, 5, 6, 7, and 10. RP-HPLC analysis indicated that, in most cases, modification at the C8-position of G or m^7^G did not substantially affect the stability compared to the reference compounds (m^7^GpppG) (Figure S15, SI). Only **3c** (m^7^GpppG^8DMAPh^) displayed moderate decomposition at pH 5–7 (with max. 18% content of unidentified products formed after 5 h at pH 5). Therefore, all biophysical experiments were conducted at close to neutral pH and within 60 min to minimize the decomposition process.

### Photophysical Properties

**Figure 1:**
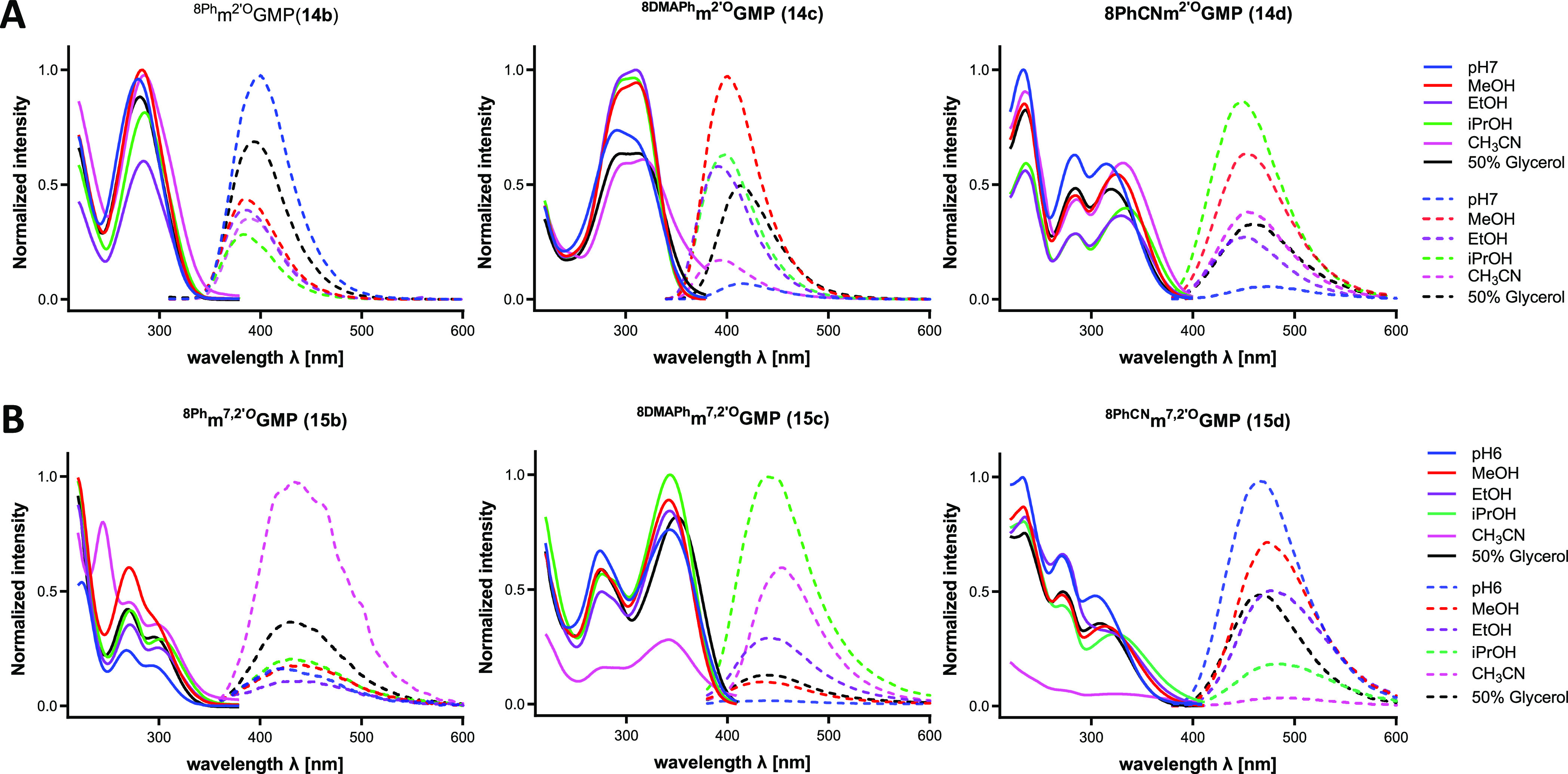
Absorption (solid line) and emission (dotted line) spectra of (A) **14b**–**d** and (B) **15b**–**d** in various solvents. The concentrations of all samples were 5 μM.

**Figure 2:**
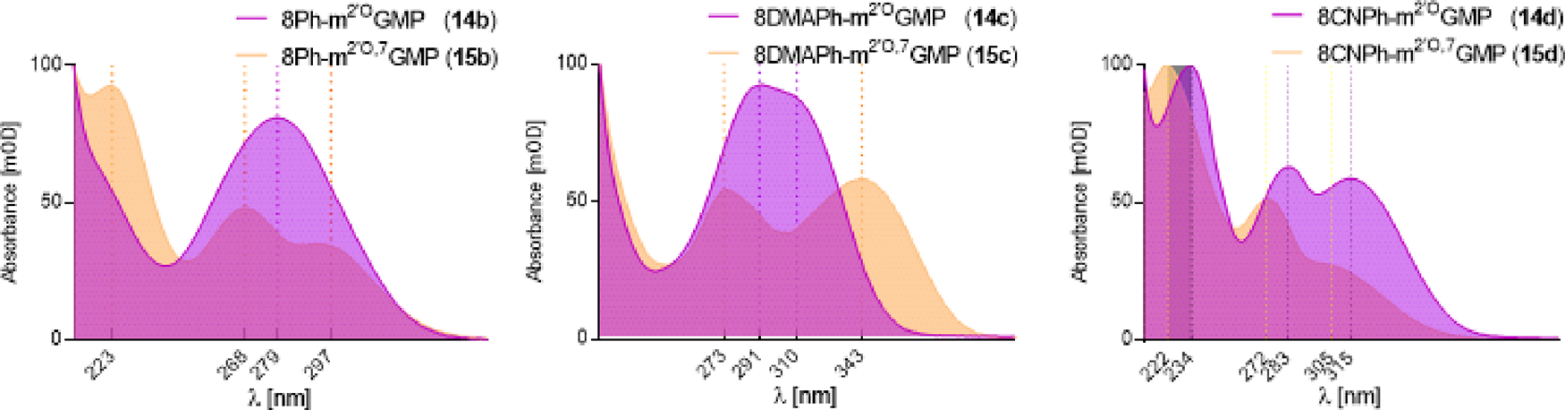
Absorption spectra of monophosphates (**14b**–**d**) and N7-monophosphates (**15b**–**d**) measured in phosphate buffer at pH 7 and 6, respectively. The concentrations of all samples were 5 μM.

The introduction of an aromatic substituent at the C8-position of the nucleic base results in strongly fluorescent nucleosides, and their fluorescence is sensitive to environmental conditions.^34,41^ These unique fluorescent properties of various C8-aryl modified adducts have been used recently as probes of DNA duplex formation or H-bonding.^33,34,42^ H-bonding, rigidity, and steric hindrance are among the many factors that can affect fluorescence. However, N7-methylation of guanine is known to significantly reduce the fluorescence quantum efficiency.^8,43^ To investigate this phenomenon, we studied the fluorescent properties of three analogs of m^2^′^*O*^GMP and m^2^′^*O*,7^GMP modified at the C8-position (Figures 1 and 2). Guanosine and 2′-*O*-Me-guanosine (m^2^′^*O*^G) monophosphates substituted at the C8-position with bromine (**11**, **12**), methyl (**13e**, **14e**), and cyclopropyl (**14f**) were not fluorescent and therefore excluded from further analysis. The shape of absorption and fluorescence spectra can be affected by solvent parameters such as polarity, polarizability, and dielectric constant. Different solvents can stabilize either the ground or excited state. To study whether new purine nucleobases with modifications at the C8-position induce significantly different spectral properties, the absorption and emission spectra of monophosphates (**14b**–**d**) and their N7-methylated analogs (**15b**–**d**) were measured in six solvents (aqueous buffer at pH 6 and 7, CH_3_CN, MeOH, EtOH, *i-*PrOH, and a 50% glycerol/water mixture; Figure 1 and Table S1, SI). Owing to solubility issues, we did not study less polar solvents. Introduction of aromatic substituents such as phenyl **(14b**, **15b**), 4-dimethylaminophenyl (**14c**, **15c**), 4-cyanophenyl (**14d**, **15d**), and extended π-conjugated systems causes a redshift of the excitation and emission maxima. These compounds possess a push–pull structure and exhibit unique spectral properties (except for the phenyl analog). This is also in agreement with Sproviero *et al*.^44^

The absorption properties of **14b**–**d** (^8Ph^m^2^′^*O*^GMP, ^8DMAPh^m^2^′^*O*^GMP, and ^8PhCN^m^2^′^*O*^GMP; in phosphate buffer at pH 7) and **15b**–**d** (^8Ph^m^7,2^′^*O*^GMP, ^8DMAPh^m^7,2^′^*O*^GMP, and ^8PhCN^m^7,2^′^*O*^GMP; in phosphate buffer at pH 6) are summarized in Table S2 (SI) and Figure 2. Briefly, **14b** exhibited a single absorption band at 279 nm, and an analogous compound with methyl group at the N7-position (**15b**) exhibited two major absorption peaks at 268 and 297 nm in aqueous buffer at pH 6. Whereas the absorption of **14b** changed slightly with solvent polarity, **15b** did not display any change at higher solvent polarity. Also, **14b** exhibited strong fluorescence emission at 400 nm in the aqueous solution, but that of **15b** was quenched in water and redshifted to 420 nm. Because of the inductive effect of substituents, **14c** and **14d** exhibited two π–π* transitions at 291/310 and 283/315 nm and weak emissions at 416 and 473 nm, respectively, which were quenched in aqueous solvents. Because of the electron-withdrawing and electron-donating effects of these substituents, the Stokes shifts varied from 125 nm for the 4-dimethylaminophenyl analog **14c** to 190 nm for the 4-cyanophenyl compound **14d** (Table S2, SI). Both compounds exhibited bathochromic shifts with increasing solvent polarity. This is usually due to solvation, which stabilizes the excited state π* that is more polar than the ground state.^44^

This phenomenon is often observed because of the charge transfer process, during which charge-separated species are formed to increase the dipole moment. In addition, increased fluorescence in viscous solvents was observed for **14c** and **14d**, which is in agreement with previously reported data for 8-PhCN-guanosine.^44^ For both compounds (being push–pull molecules), their fluorescence is quenched in aqueous solutions, which is likely caused by faster nonradiative decay due to H-bonding.^45^ However, a reverse hypsochromic shift with an increase in solvent polarity was observed for compounds **15c** and **15d**, suggesting that their ground state is more polar than the excited state π*. A similar situation was observed for thioflavin T (ThT),^46^ whose benzothiazole aniline backbone structurally resembles the N7-methylguanine moiety. The photophysical properties of ThT derivatives depend significantly on the ionic form of the molecules. Noncharged derivatives of ThT without a methyl group at the N3-position of the benzothiazole residue are characterized by intense fluorescence and behave as regular solvent viscosity-independent fluorophores.^47^ In contrast, ThT as a positively charged *N*-methyl benzothiazolium cation exhibits viscosity-dependent fluorescence and demonstrates fluorescent molecular rotor properties.^47^ Quantum chemical calculations of ThT confirmed that the TICT process plays an essential role in this molecule.^48,49^ Its excited singlet state switches from the fluorescent locally excited (LE) state to the nonfluorescent TICT state, and this process is responsible for quenching of ThT fluorescence in low-viscosity solvents. In viscous media, internal rotation (TICT formation) is blocked by steric hindrance, and a stronger fluorescence is observed simultaneously with the charge redistribution. This leads to a change in the dipole moment of the molecule.^49^ Therefore, we assume that a similar phenomenon occurs in **15c** and **15d**.

To verify whether the synthesized cap analogs bearing an aromatic substituent at the C8-position display molecular rotor properties, we conducted an experiment using various glycerol concentrations (Figures S16 and S17, SI). All tested analogs (**3a**-**3d**, **4a**-**b**, **5b**-**5d**, **6**, **7**, and **8**) exhibited viscosity-dependent fluorescence properties. The three compounds showing the greatest changes in their fluorescence intensity are ^8DMAPh^m^7,2^′^*O*^GpppG (**5c**), m^7^GpppG^8DMAPh^ (**3c**), and m^7,2^′^*O*^GpppG^8Ph^ (**4b**).The solution of **5c** in pure glycerol had a 263-fold higher fluorescence intensity than the aqueous solution at the same concentration, making this compound a good candidate for studying protein interactions.

**Figure 3:**
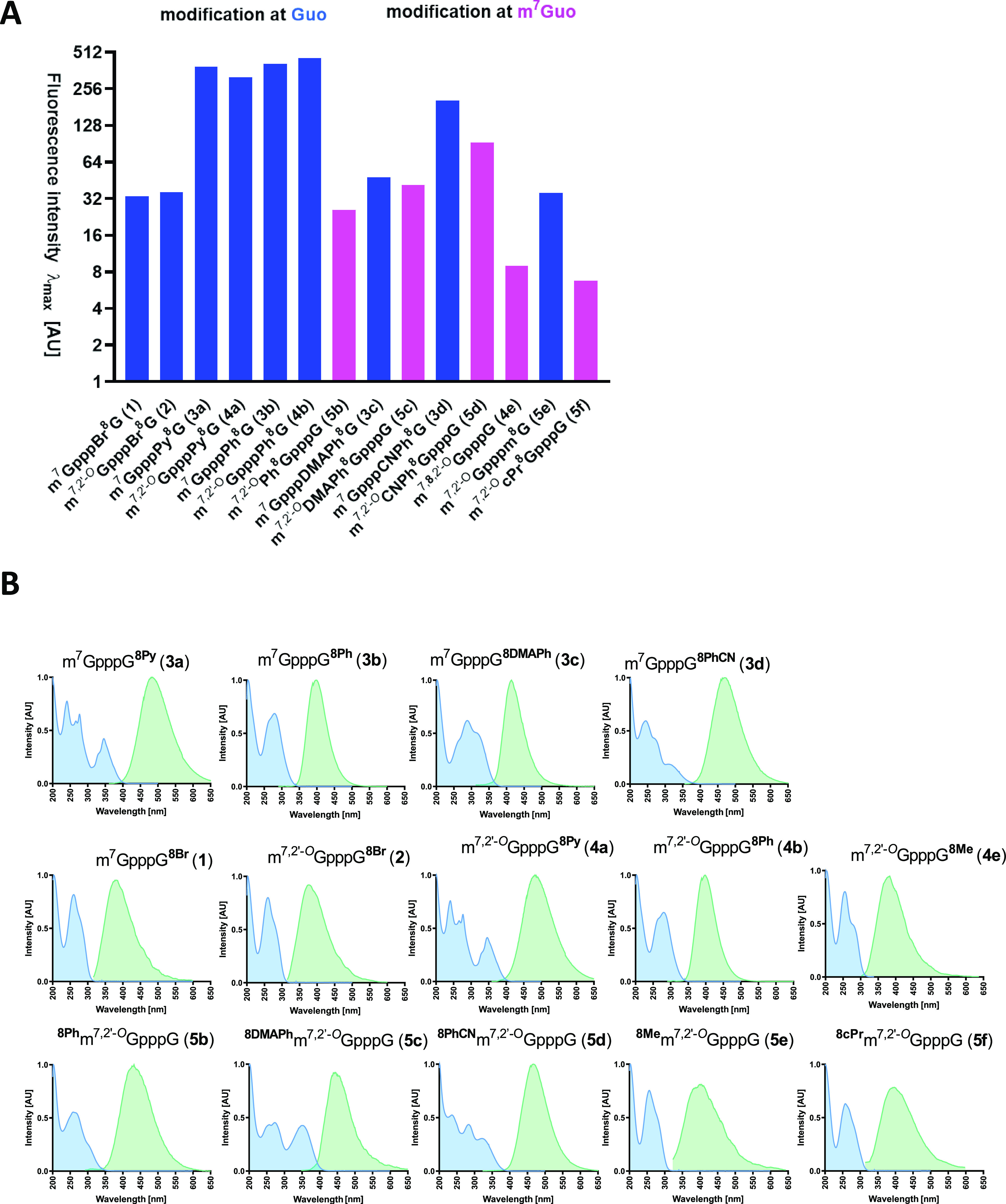
(A) Comparison of fluorescence intensity of C8-modified cap analogs depending on the site of modification. (B) Normalized absorption (blue) and emission (green) spectra of C8-modified cap analogs. The concentrations of all samples were 5 μM.

We also measured the spectra of dinucleotide cap analogs **1**–**5f** modified at the C8-position of either G or m^7^G under similar conditions and compared their spectral properties (Figure 3). All aromatic substituents enhanced the fluorescence quantum yields of the cap analogs in polar solvents in the order of Ph > Py > PhCN > DMAPh. However, as expected, the weakest fluorescence signals were observed for compounds modified with nonaromatic substituents (−Me, −Br, and −cPr). Similar to monophosphates, the fluorescence intensity is quenched by the presence of a methyl group at the N7-position of guanine. Good spectral features are advantageous in the biophysical studies with proteins. The most promising compounds for such studies are those with −PhCN and −DMAPh modifications owing to their characteristic bathochromic shift.

## Biophysical Studies

Modifications of the cap structure are most often introduced to alter the biological properties. How do changes in the cap structure translate into biological activity? A number of valuable clues have been provided by biophysical studies of proteins that are involved in cellular interactions with the cap. Therefore, we selected four proteins that recognize the RNA 5*′* caps in cells: a translation initiation factor (eIF4E), two enzymes associated with cap degradation (DcpS and Nudt16), and a protein transporting certain RNA variants from the cytoplasm to the cell nucleus (snurportin).

### Interaction with eIF4E

An interaction between the mRNA 5′ cap and eIF4E is required for translation initiation and subsequent protein biosynthesis.^50^ The specific recognition of the mRNA 5*′* cap by eIF4E is the rate-limiting step for translation initiation,^51^ and low translatability has been observed for mRNAs carrying cap analogs having lower affinity to eIF4E than natural caps.^52^ Hence, a high affinity to eIF4E is desired for many applications of synthetic cap analogs.

**Figure 4:**
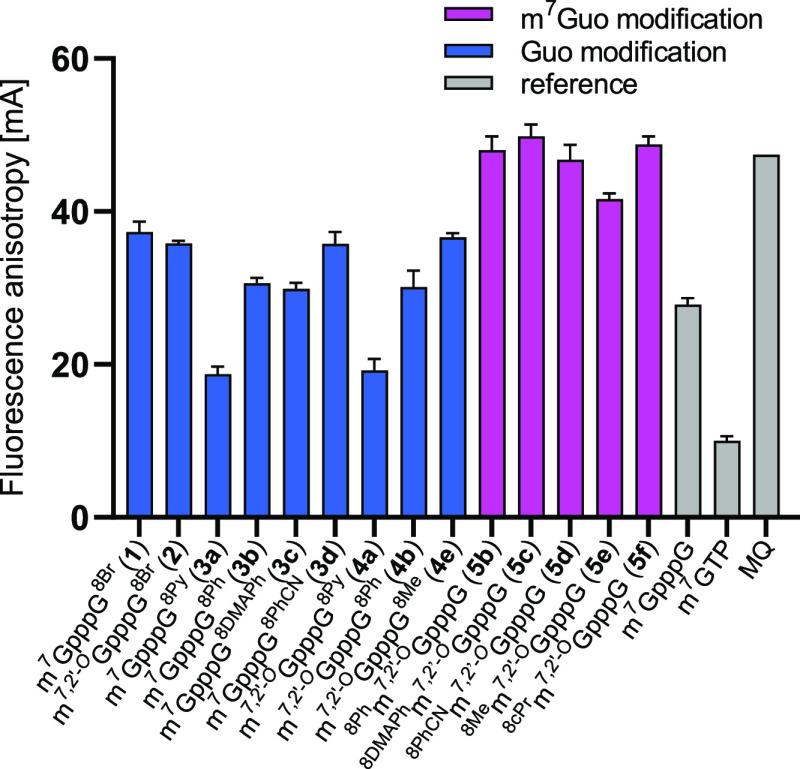
Screening of C8-modified cap analogs with eIF4E protein.

We investigated the relative affinities of modified dinucleotide cap analogs for eIF4E using the fluorescence anisotropy (FA) technique.^53^ The assay relies on replacing a fluorescent probe (the structure is presented in Figure S18) from the cap-binding site of eIF4E, which results in a decrease in fluorescence anisotropy of the sample. The results are shown in Figure 4. All cap analogs bearing modifications at the C8-position of m^7^G are characterized by low affinity to eIF4E. Even a relatively small substituent such as methyl group at the C8-position of m^7^G caused a notable decrease in affinity. On the other hand, C8-substitution at the second base (guanine) only minimally influenced eIF4E affinity compared to the native cap structure of m^7^GpppG. Among the studied compounds, analogs containing a bulky pyrene substituent (**3a**, **4a**) showed the highest affinity, which is somewhat surprising. This may be due to additional hydrophobic interactions of the pyrene residue with the eIF4E protein, which are not possible for smaller substituents. We also found that compounds **3a** and **4a** are better ligands for eIF4E than known unmodified ligand m^7^GpppG. The additional 2′-*O*-methyl group at the m^7^G side did not affect affinity in any case (see **1** and **2**, **3a** and **4a**, **3b** and **4b**).

### Translation Efficiency in HeLa Cells

**Figure 5:**
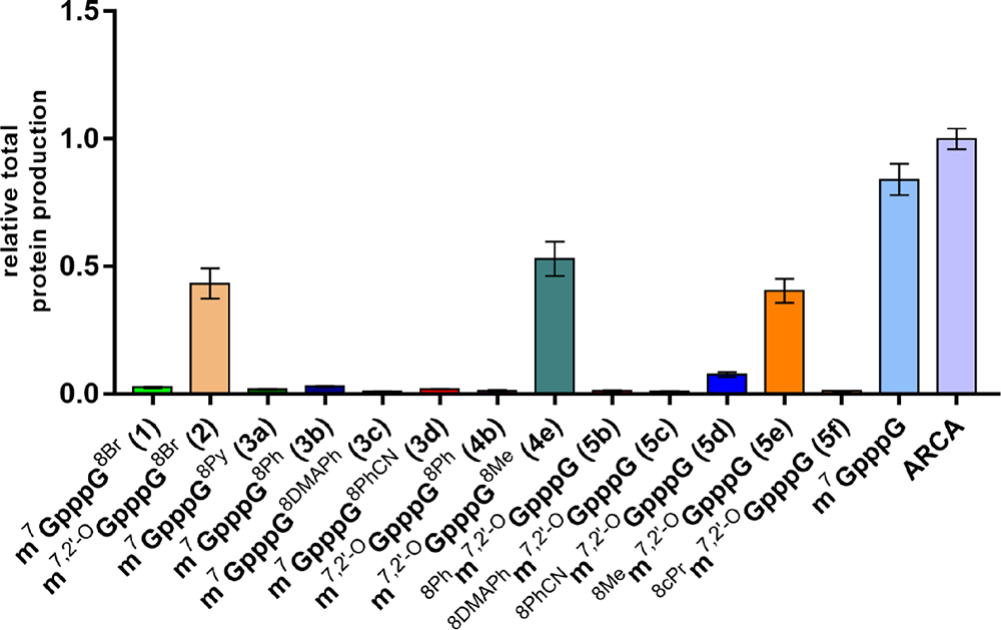
Translation efficiency of mRNAs carrying various C8-modified cap analogs at the 5′ end, evaluated as total protein expression (cumulative luminescence) in HeLa cells.

Next, we investigated the influence of C8-modifications on capping effectiveness during *in vitro* transcription (IVT) and protein production levels in HeLa cells using previously described methods.^7^ It should be noted that mRNA obtained by IVT (details of the synthesis of 5′ capped mRNA are described in the Experimental Section) was used directly in the translation experiment without removing the uncapped mRNA. Thus, we were evaluating properties of the reactants for obtaining mRNA and not the effect of modification itself on the level of translation. In other words, the yield of protein production in Figure 5 reflects the percentage of capping and effectiveness of translation.

HeLa cells were transfected with IVT mRNAs encoding firefly luciferase capped with C8-modified cap analogs. To account for variations in transfection efficiency between samples, mRNA encoding *Renilla* luciferase capped with ARCA was used as an internal control for each transfection. Activities of firefly and *Renilla* luciferases were measured using a dual-reporter assay at different time points post transfection. Firefly luciferase activity was normalized to *Renilla* luciferase activity and plotted as a function of time (Figure S19). The total protein production level was calculated for each mRNA based on the activity curve and is shown in Figure 5. The presence of substituents at the C8-position generally had a negative impact on the translational potential of mRNA, although the reasons may be different between analogs. For modifications within m^7^G, it is likely due to interference in the association with eIF4E, which reduces translation efficiency. In contrast, the low protein output for mRNAs capped with compounds carrying C8-substituted guanosine is most likely caused by less efficient incorporation of the cap analog into mRNA transcripts (the exact percentages of capped fraction in mRNA samples are given in Table 2). Only mRNAs capped with analogs possessing the smallest modification, namely, methyl group at either the m^7^G or G moiety, were translated at a reasonable level. However, the bulkier ARCA version of C8-bromo analog (**2**) was also expressed, whereas a similar compound without 2′-*O* modification (**1**) was not. This is consistent with studies showing that RNAs capped with ARCA analogs are translationally more active than non-ARCA capped ones because the latter are incorporated in both normal and reverse orientations.^31^ All these data indicate that modification of the C8-position of m^7^G unfortunately disrupts cap-eIF4E interaction and effective mRNA translation *in vivo*. However, this phenomenon may be useful for designing inhibitors of other cap-dependent proteins that do not interfere with translation.

**Table 2:** Susceptibility to DcpS and Nudt16 and Capping Efficiency in IVT

no.	compound	DcpS (% of hydrolysis after 30 min)	Nudt16 (% of hydrolysis after 60 min)	capping %^a^
	m^7,2^′^*O*^GpppG	n.d.	n.d.	67
	m^7^GpppG	hydrolyzed (69%)	hydrolyzed (79%)	
**1**	m^7^GpppG^8Br^	hydrolyzed (88%)	resistant	52
**2**	m^7,2^′^*O*^GpppG^8Br^	resistant	resistant	12
**3a**	m^7^GpppG^8Py^	hydrolyzed (98%)	hydrolyzed (36%)	30
**3b**	m^7^GpppG^8Ph^	hydrolyzed (47%)	resistant	45
**3c**	m^7^GpppG^8DMAPh^	hydrolyzed (71%)	resistant	30
**4a**	m^7,2^′^*O*^GpppG^8Py^	resistant	hydrolyzed (30%)	64
**4b**	m^7,2^′^*O*^GpppG^8Ph^	resistant	resistant	16
**4d**	m^7^GpppG^8PhCN^	hydrolyzed (25%)	resistant	20
**4e**	m^7,2^′^*O*^GpppG^8Me^	hydrolyzed (27%)	resistant	50
**5b**	^8Ph^m^7,2^′^*O*^GpppG	resistant	hydrolyzed (98%)	47
**5c**	^8DMAPh^m^7,2^′^*O*^GpppG	resistant	hydrolyzed (80%)	38
**5d**	^8PhCN^m^7,2^′^*O*^GpppG	resistant	hydrolyzed (62%)	46
**5e**	^8Me^m^7,2^′^*O*^GpppG	resistant	hydrolyzed (99%)	55
**5f**	^8cPr^m^7,2^′^*O*^GpppG	resistant	hydrolyzed (94%)	52

a Fraction of capped RNA

### Interactions with DcpS and Nudt16

DcpS and Nudt16 are pyrophosphatases involved in mRNA metabolism. DcpS belongs to the histidine triad (HIT) superfamily. It cleaves the cap between the γ and β phosphates, releasing N7-methylguanosine monophosphate (m^7^GMP) and guanosine diphosphate GDP.^54^ This protein has been identified as a molecular target for spinal muscular atrophy (SMA).^4^ A correlation between inhibition of DcpS activity and improvement of motor function has been observed in a mouse model.^55^ Nudt16 is a member of the Nudix family and hydrolyzes substrates composed of a nucleoside diphosphate linked to another moiety X.^56^ Being involved in the 5′ → 3′ degradation pathway, Nudt16 cleaves capped mRNAs or dinucleotide cap analogs between α and β phosphates, releasing m^7^GDP and monophosphate.^57^ Nudt16 displays activity toward a wide range of nucleotide substrates and shows various specificities.^58,59^ A recent study revealed that human Nudt16 (hNudt16) preferentially hydrolyzes unmethylated caps.^59^

We decided to use these two enzymes with different substrate specificities to characterize the C8-modified cap analogs. We first investigated the susceptibility of C8-modifed cap analogs to hydrolysis by hDcpS and Nudt16 (Figures S20 and S21, SI). The cap analogs (**1**, **2**, **3a**–**d**, **4a**–**b**, **4e**, **5b**–**f**) and the reference analog m^7^GpppG (20 μM) were incubated with 28 nM hDcpS for 30 min or 710 nM hNudt16 for 60 min. The percentage of the remaining substrate was determined by RP-HPLC analysis, and the susceptibility to hydrolysis is summarized in Table 2.

Cap analogs modified at the C8-position of m^7^G (**5b**–**f**) were completely resistant to hydrolysis by hDcpS, indicating that this modification effectively protects the cap structure from degradation. However, hydrolytic susceptibility depends not only on the C8-modification site but also on the presence/absence of 2′-*O*-Me (m^2^′^*O*^) modification. Whereas cap analogs with a single C8-modification at G (**3a**–**c**) were slowly hydrolyzed by DcpS, analogous compounds bearing also the 2′-*O*-Me group (**4a** and **5b**–**5c**) were less prone to hydrolysis.

**Figure 6:**
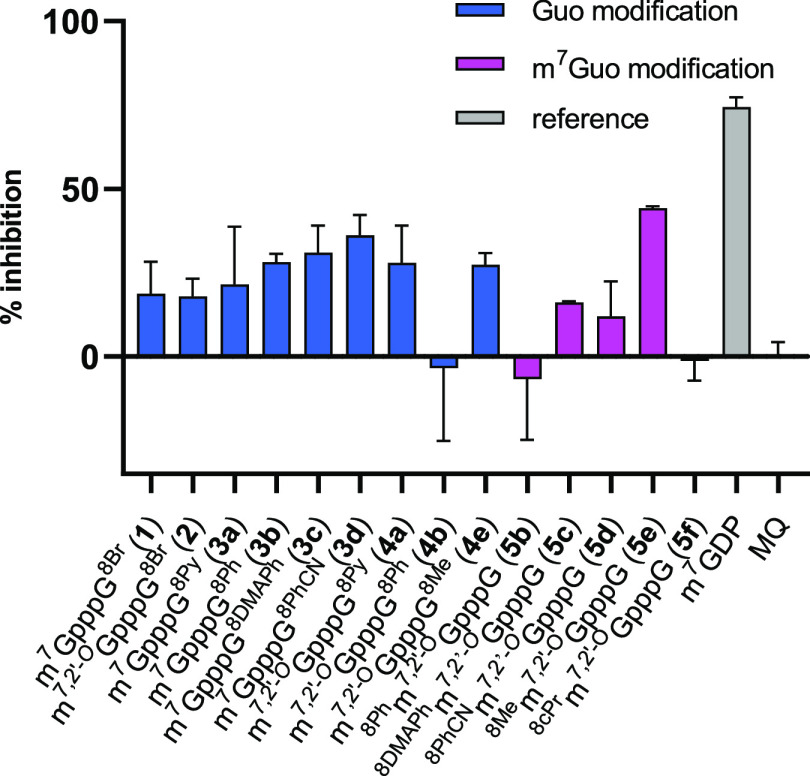
Screening of C8-modified cap analogs with DcpS.

Next, these compounds were tested as DcpS inhibitors using a fluorescence-based high-throughput screening (HTS) assay (Figure 6).^60^ None of the tested compounds showed superior inhibition to the reference compound m^7^GDP (77% inhibition). The best response was observed for **5e** carrying the 8-methyl group, but the inhibition (44%) was still 2-fold less potent than that of m^7^GDP.

In the case of Nudt16, all compounds possessing modifications at the C8-position of m^7^G were hydrolyzed. Relatively faster hydrolysis was observed for compounds with methyl and cyclopropyl substituents (**5e**, **5f**), and those with bulkier *p*-cyanophenyl or *p*-dimethylaminophenyl groups showed slower hydrolysis. On the other hand, substituents at the C8-position of guanosine made cap analogs resistant to hydrolysis, with the exception of **3a** and **4a** (C8-Br and C8-Pyr, respectively).

### Interaction with Snurportin

**Figure 7:**
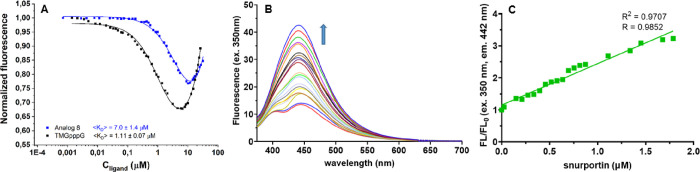
(A) Titration of 0.1 μM snurportin with compound **8** or unmodified TMGpppG. (B) Emission spectra of 1 μM compound **8** during titration with snurportin (from 0 to 2 μM). (C) Influence of snurportin concentration on the fluorescence intensity of compound **8.**

Snurportin is an adaptor protein that recognizes small nuclear RNA (snRNA) with m^2,2,7^Gppp (m_3_G cap, TMG cap) at its 5′ end and mediates its transport to the cell nucleus.^61^ Snurportin-TMG cap analog interactions are important for spliceosome formation and maturation of small nuclear ribonucleoprotein (snRNP).^62^ Hence, the interaction of compound **8** carrying the m^2,2,7^Gppp structural motif was investigated using snurportin. First, we used a fluorescence quenching titration (FQT) assay to assess how modification at the C8-position of the second nucleobase influences this interaction (Figure 7A). In this experiment, snurportin was titrated with increasing concentrations of **8**, and the intensity of snurportin emission at 345 nm was recorded after each addition. The data allowed us to calculate the binding affinity (*K*_d_) between snurportin and **8** (for details, see the Experimental Section).

The *K*_d_ value determined for m^2,2,7^Gppp^8DMAPh^m^7^G (**8**) and snurportin was 6-fold higher than that for natural m^2,2,7^GpppG (*K*_d_ = 1.1 μM),^63^ showing that modification of the second nucleoside destabilizes but does not completely disrupt the interaction with snurportin. Because modified dinucleotide **8** is specifically recognized by snurportin, it has potential applications as a molecular probe. Hence, next we performed the reverse experiment of titrating **8** with snurportin (Figure 7B,C) to test whether the changing emission from this compound can be used to probe interaction with the protein. The emission intensity of **8** at maximum (442 nm) gradually increased with higher snurportin concentration, reaching a 3-fold increase at 2 μM protein. This finding suggests that the ^8DMAPh^m^7^G motif may act as a synthetic equivalent of a fluorescent molecular rotor and be useful for ligand-specific protein sensing or quantification.

## Conclusions

In this work, we proposed two strategies for the modification of cap analogs via Suzuki–Miyaura cross-coupling at the C8-position of 7-methylguanosine or guanosine as the second nucleoside in the RNA cap structure. We proved that even fragile compounds such as the 7-metylguanosine cap may be efficiently postsynthetically modified by this method. We synthesized a series of dinucleotide cap analogs with various substituents at the C8-position of the first or second nucleoside. A total of 19 dinucleotide analogs were obtained and further evaluated for their biophysical and biochemical properties. Aromatic substituents such as phenyl, pyrene, or cyanophenyl confer stronger fluorescence properties when introduced on guanine (*e.g.*, **3a**, **3b**, **4b**, **4d**) rather than on N7-methylated guanine. Three of the compounds [m^7^GpppG^8DMAPh^ (**3c**), m^7,2^′^*O*^GpppG^8Ph^ (**4b**), and especially ^8DMAPh^m^7,2^′^*O*^GpppG (**5c**)] could serve as biological probes that are sensitive to the local viscosity of the molecular environment. We identified several modifications of guanine (**3b, 3c, 4b)** that were accepted by eIF4E with only slightly disturbed protein interaction. Compounds bearing both the methyl group at the N7-position of guanine and the 2′-*O*-Me modification (**5b**–**5f**) are resistant to DcpS hydrolysis. We also identified molecular motifs based on m^7^G that behave like molecular rotors and can be used to design molecular biosensors. These results indicate that the C8-position of 7-methylguanosine is extremely important in the recognition of cap structure by specific proteins, leading to translation inhibition and resistance to enzymes involved in degradation of the 5*′* end of mRNAs. This knowledge is valuable from the viewpoint of designing inhibitors related to 5*′* end mRNA metabolism.

## Experimental Section

2′-*O*-Me-guanosine (**9**) and guanosine 5′-monophosphate disodium salts (**10**) were purchased from BIOLOG Life Science Institute (Germany) and Carbosynth (UK), respectively. Boronic acids (methyl, cyclopropyl, phenyl, 4-(dimethylamino)phenyl, 4-cyanophenyl, and 1-pyrene), Pd(OAc)_2_, and triphenylphosphine-3,3′,3″-trisulfonic acid trisodium salt (TPPTS) were purchased from Sigma-Aldrich (US) and used without further purification. DMF and DMSO were dried over molecular sieves (3 Å) for at least 24 h before use. Triethylammonium salts of m^7^GMP, m^7^GDP, GDP-Im, and m^7^GDP-Im were obtained as described in the literature.^31^ m^2^′^*O*^GMP was prepared according to the literature.^30,31^ Sodium salts of the nucleotides were converted into triethylammonium salts using a Dowex 50WX8 ion-exchange resin.

### Chromatography

#### Ion-Exchange Chromatography

The synthesized nucleotides were purified by ion-exchange chromatography on a DEAE Sephadex A-25 (HCO_3_^–^ form) column. After loading the column with the reaction mixture and washing with deionized water, the products were eluted using different gradients of triethylammonium bicarbonate (TEAB) buffer in deionized water: 0–0.7 M for nucleoside monophosphates, 0–1.0 M for nucleoside diphosphates, and 0–1.2 M for nucleoside triphosphates. Fractions containing the desired product were collected together after RP-HPLC and spectrophotometric analysis at 260 nm. The nucleotide analogs were isolated as triethylammonium salts by evaporation under reduced pressure with repeated additions of 96% and then 99.8% ethanol.

#### Analytical and Preparative RP-HPLC

Analytical RP-HPLC was performed on an Agilent Technologies Series 1200 apparatus equipped with a Supelcosil LC-18 RP-T column (5 μm, 4.6 × 250 mm, flow rate 1.3 mL/min), Supelcosil LC-8 HPLC column (5 μm, 4.6 × 250 mm, flow rate 0.75 mL/min), or Phenomenex Gemini column (3 μm, 4.6 × 150 mm, flow rate 1.0 mL/min). The following gradient elution conditions were applied:

- Method A (Supelcosil C18 column): 15 min gradient of 0–100% methanol in 0.05 M ammonium acetate buffer at pH 5.9.
- Method B (Supelcosil C18 column): 7.5 min gradient of 0–100% methanol in 0.05 M ammonium acetate buffer at pH 5.9.
- Method C (Supelcosil C8 column): 15 min gradient of 0–50% acetonitrile in 0.05 M ammonium acetate buffer at pH 5.9.
- Method D (Gemini C18 column): 15 min gradient of 0–100% methanol in 0.05 M ammonium acetate buffer at pH 5.9.

Unless stated otherwise, the eluted compounds were detected using a UV–vis detector (254 nm) and a fluorescence detector (excitation wavelength 260 nm, emission wavelength 370 nm). The final C8-substituted monophosphates (**13a**–**e** and **15b**–**f**) were purified by preparative RP-HPLC using C18 cartridges (puriFlash, 20 g C18, 15 μm, flow rate 10 mL/min) on a Reveleris flash chromatography system equipped with a variable-wavelength UV and evaporative light scattering detector (ELSD). A linear gradient of acetonitrile in 0.05 M ammonium acetate (pH 5.9) was used as the mobile phase. Semipreparative RP-HPLC was carried out on an Agilent Technologies Series 1200 apparatus equipped with a Discovery RP Amide C16 column (21.2 × 250 mm, flow rate 5.0 mL/min) using a linear gradient (0–30%) of acetonitrile in 0.05 M ammonium acetate (pH 5.9) as the mobile phase. The eluted compounds were collected and subjected to repeated lyophilization. The pure products were isolated as ammonium salts.

### Nuclear Magnetic Resonance

^1^H and ^31^P NMR spectra were recorded at 25 or 45 °C on a Varian UNITY-plus at 399.94 and 161.90 MHz, respectively. ^1^H NMR chemical shifts were reported in ppm with the residual solvent peak as the internal standard. ^31^P NMR chemical shifts were reported using 20% phosphoric acid in D_2_O as the external standard. The chemical shifts (δ) are given in ppm, and the coupling constants (*J*) are given in Hz. Where applicable, structural assignments were made with additional information from gCOSY and gHSQC experiments. Assignments were as follows: s, singlet; bs, broad singlet; d, doublet; t, triplet; q, quartet; dd, a doublet of doublets; dt, a doublet of triplets; td, a triplet of doublets; and m, multiplet.

### High-Resolution Mass Spectrometry

Low-resolution mass spectra were measured using a Thermo Scientific LTQ OrbitrapVelos spectrometer (Thermo Fisher Scientific, Waltham, MA, USA). The structure and purity of the final compounds were confirmed by high-resolution mass spectrometry (Micromass QTOF MS 1) using the ESI ionization technique in the negative [MS ESI (−)] or positive ion mode [MS ESI (+)].

### UV–Vis and Fluorescence Measurement

UV absorption spectra were recorded using a Shimadzu UV-1800 spectrophotometer at 25 °C in 0.1 M phosphate buffer. The buffer pH was 7.0 for monophosphates and cap dinucleotides and 6.0 for N7-methyl monophosphates. Emission spectra were recorded at 25 °C using a Cary Eclipse (Agilent Technologies, Santa Clara, CA, USA) equipped with a xenon lamp under thermostatic conditions using a 10 × 4 mm quartz cuvette in various solvents and buffers.

### Chemical Synthesis

#### 8-Bromoguanosine 5′-Monophosphate, ^8Br^GMP (**11**)^64^

To a solution of GMP (**9**) (1 g, 2.45 mmol) in 0.5 M NaOAc buffer (100 mL, pH 4.0), saturated bromine water was added dropwise until the yellow color persisted. The reaction mixture was stirred at room temperature (RT) until the starting material was depleted as determined by RP-HPLC analysis. The solution was then washed with CHCl_3_ (3 × 50 mL) to remove excess bromine. The aqueous phase was concentrated to 50 mL using a rotary evaporator, and the crude product was purified by ion-exchange column chromatography using a linear gradient of 0.7 M TEAB buffer. The product was obtained as a triethylammonium salt. Yield 700 mg, 13,200 OD, 62%. For NMR analysis, a small quantity of the material was further purified by preparative RP-HPLC and isolated as an ammonium salt. Rt (A) = 5.88 min; ^1^H NMR (500 MHz, D_2_O) δ 5.96 (d, *J* = 6.1 Hz, 1H), 5.29 (t, *J* = 5.9 Hz, 1H,), 4.59 (dd, *J* = 5.8 Hz, 3.9 Hz, 1H), 4.25 (td, *J* = 5.5 Hz, 3.9 Hz, 1H), 4.11 (dt, *J* = 11.2 Hz, 5.6 Hz, 1H), 4.02 (dt, *J* = 11.4 Hz, 5.8 Hz, 1H); ^31^P NMR (202 MHz, D_2_O) δ 3.3 (t, *J* = 5.6 Hz, 1P); HRMS ESI (−) *m*/*z* [M – H]^−^, calcd for C_10_H_12_BrN_5_O_8_P^–^ 439.9612, 441.9592; found 439.9617, 441.9596.

#### 8-Bromo-2′-*O*-methylguanosine 5′-Monophosphate, ^8Br^m^2^′^*O*^GMP (**12**)

To a solution of 2′-*O*-Me-GMP triethylammonium salt (**10**) (500 mg, 0.86 mmol) in dry DMF (3 mL), NBS (370 mg, 2.07 mmol) was added, and the reaction mixture was stirred at RT using a magnetic stirrer. After depletion of the starting material as determined by RP-HPLC analysis (Figure S1, SI), the crude product was purified by ion-exchange column chromatography using a linear gradient of 0.7 M TEAB buffer. The product was obtained as a triethylammonium salt. Yield 670 mg, 12,250 OD, 77%. For NMR analysis, a small quantity of the material was further purified by preparative RP-HPLC and isolated as an ammonium salt. Rt (A) = 8.77 min; ^1^H NMR (400 MHz, D_2_O) δ = 6.03 (d, *J* = 6.0 Hz, 1H), 5.02 (dd, *J* = 6.0, 5.6 Hz, 1H), 4.76 (dd, *J* = 5.6 Hz, 3.9 Hz, 1H), 4.27 (q, *J* = 4.9 Hz, 4.2 Hz, 1H), 4.17 (m, 2H), 3.43 (s, 3H). ^31^P NMR (162 MHz, D_2_O) ^31^P^15^ δ = 0.24 (s, 1P); ^31^P NMR δ = 0.24 (t, 1P, *J* = 5.9 Hz); HRMS ESI (−) *m*/*z* [M – H]^−^, calcd for C_11_H_14_BrN_5_O_8_P^–^; exact mass: 453.9769; found: 453.9779, 455.9758.

### General Protocol for Suzuki–Miyaura Coupling Reaction

#### General Procedure A: Suzuki–Miyaura Coupling on Nucleotides with Nonaromatic Boronic Acids

Palladium acetate (3.36 mg, 0.015 mmol), TPPTS (42.6 mg, 0.075 mmol), sodium carbonate (146.7 mg, 0.45 mmol), ^8Br^GMP or ^8Br^m^2^′^*O*^GMP (0.15 mmol), and methyl or cyclopropylboronic acid (1.50 mmol, 10 equiv) were added to a degassed mixture of water/acetonitrile (2:1 v/v, 4.0 mL) and heated to 95 °C for 60 min under an argon atmosphere. Upon completion of reaction, the mixture was diluted with *ca*. 4.0 mL of water, and the pH was adjusted to 7 using 10% HCl. The crude products were purified by preparative RP-HPLC flash chromatography.

#### General Procedure B: Suzuki–Miyaura Coupling on Nucleotides with Aromatic Boronic Acids

Palladium acetate (3.36 mg, 0.015 mmol), TPPTS (42.6 mg, 0.075 mmol), sodium carbonate (146.7 mg, 0.45 mmol), ^8Br^GMP or ^8Br^m^2^′^-*O*^GMP (0.15 mmol), and boronic acid (1-pyrene, phenyl, 4-dimethylaminophenyl, or 4-cyanophenyl; 0.30 mmol, 2 equiv) were added to a degassed mixture of water/acetonitrile (2:1 v/v, 4.0 mL) and heated to 90 °C for 30 min under an argon atmosphere. Upon completion of reaction, the mixture was diluted with *ca*. 4.0 mL of water, and the pH was adjusted to 7 using 10% HCl. The crude products were purified using preparative RP-HPLC flash chromatography.

#### General Procedure C: Postsynthetic Suzuki–Miyaura Coupling

To prepare the Pd(OAc)_2_(TPPTS)_2_ complex as catalyst, Pd(OAc)_2_ (11.2 mg, 0.05 mmol) was added to TPPTS (56.8 mg, 0.1 mmol) dissolved in 1 mL of degassed water. The solution was stirred for 10 min at RT to obtain a dark red solution. The final stock solution (50 mM) was divided into 50 μL aliquots and kept at −20 °C. The catalyst solution was defrosted and used within 1 h. The reaction was performed in a 1.5 mL screw cap microcentrifuge Eppendorf tube. To a solution of m^7^GpppG^8Br^ (10 μL, 50 mM, 1 equiv) in 100 μL of degassed NaHCO_3_ buffer (100 mM, pH 8.0), boronic acid dissolved in DMSO (10 μL, 500 mM, 10 equiv) was added. [To dissolve (4-dimethylaminophenyl)boronic acid, 50 μL of NaHCO_3_ buffer (100 mM, pH 8.5) was added instead.] The prepared Pd(OAc)_2_(TPPTS)_2_ complex (5 μL, 50 mM, 0.5 equiv) was then added. The final reaction volume was 125 μL with a cap concentration of 4 mM. The mixture was placed on a preheated thermoblock at 90 °C for 15 min, and the products were analyzed by analytical RP-HPLC.

##### 8-(1-Pyrene)-guanosine 5′-Monophosphate, ^8Py^GMP (**13a**)

^8Py^GMP (**13a**) was obtained from ^8Br^GMP (**11**) (96 mg, 0.15 mmol) following general procedure A. Yield 42 mg, 35%. Rt (D) = 15.05 min; ^1^H NMR (500 MHz, methanol-*d*_4_, 318 K) δ 8.36–8.24 (m, 3H), 8.24–8.14 (m, 4H), 8.08 (q, *J* = 7.7 Hz, 2H), 5.53–5.31 (m, 2H), 4.44–4.26 (m, 2H, H-3’, H-5’), 4.07–4.00 (m, 1H, H-5”), 3.96 (bs, 1H, H-4’). ^31^P NMR (203 MHz, DMSO-*d*_6_, 318 K) δ −0.11. HRMS ESI (−) *m*/*z* [M – H]^−^, calcd for C_26_H_21_N_5_O_8_P^–^ 562.1133; found 562.1136.

##### 8-Phenylguanosine 5′-Monophosphate, ^8Ph^GMP (**13b**)^64^

^8Ph^GMP (**13b**) was obtained from ^8Br^GMP (**11**) (96 mg, 0.15 mmol) following general procedure A. Yield 70 mg, 70%. Rt (D) = 8.90 min; ^1^H NMR (500 MHz, D_2_O) δ 7.73–7.53 (m, 5H), 5.79 (d, *J* = 6.2 Hz, 1H), 5.28 (dd, *J* = 6.2 Hz, 5.7 Hz, 1H), 4.50 (dd, *J* = 5.7 Hz, 3.5 Hz, 1H), 4.23–4.12 (m, 3H); ^31^P NMR (202 MHz, D_2_O) δ −0.91 (t, *J* = 5.7 Hz, 1P); HRMS ESI (−) *m*/*z* [M – H]^−^, calcd for C_16_H_17_N_5_O_8_P^–^ 438.0820; found 438.0819.

##### 8-(4-Dimethylaminophenyl)guanosine 5′-Monophosphate, ^8DMAPh^GMP (**13c**)

^8DMAPh^GMP (**13c**) was obtained from ^8Br^GMP (**11**) (96 mg, 0.15 mmol) following general procedure A. Yield 100 mg, 94%. Rt (D) = 8.72 min; ^1^H NMR (500 MHz, D_2_O) δ 7.45 (d, *J* = 8.7 Hz, 2H), 6.93 (d, *J* = 8.7 Hz, 2H), 5.75 (d, *J* = 5.7 Hz, 1H), 5.20 (t, *J* = 5.7 Hz, 1H), 4.54 (m, 1H), 4.21 (m, 3H), 3.03 (s, 6H); ^31^P NMR (202 MHz, D_2_O) δ 1.39 (t, *J* = 5.6 Hz, 1P); HRMS ESI (−) *m*/*z* [M – H]^−^, calcd for C_18_H_22_N_6_O_8_P^–^ 481.1242; found 481.1243.

##### 8-(4-Cyanophenyl)-guanosine 5′-Monophosphate, ^8PhCN^GMP (**13d**)

^8PhCN^GMP (**13d**) was obtained from ^8Br^GMP (**11**) (96 mg, 0.15 mmol) following general procedure A. Yield 95 mg, 92%. Rt (D) = 9.87 min; ^1^H NMR (500 MHz, D_2_O) δ 7.84 (m, 2H, H_ar_), 7.71 (m, 2H, H_ar_), 5.72 (d, *J* = 6.0 Hz, 1H), 5.26 (dd, *J* = 6.0 Hz, 5.8 Hz, 1H), 4.54 (dd, *J* = 5.8 Hz, 3.7 Hz, 1H), 4.22 (m, 2H), 4.16 (m, 1H); ^31^P NMR (202 MHz, D_2_O) δ 1.34 (t, *J* = 5.6 Hz, 1P); HRMS ESI (−) *m*/*z* [M – H]^−^, calcd for C_17_H_16_N_6_O_8_P^–^ 463.0773; found 463.0776.

##### 8-Methylguanosine 5′-Monophosphate, ^8Me^GMP (**13e**)^65^

^8Me^GMP (**13e**) was obtained from ^8Br^GMP triethylammonium salt (**11**) (100 mg, 0.15 mmol) following general procedure A. Yield 66 mg, 73%. Rt (A) = 4.52 min; ^1^H NMR (400 MHz, deuterium oxide) δ 5.86 (d, *J* = 6.7 Hz, 1H), 5.13 (dd, *J* = 6.7 Hz, 5.8 Hz, 1H), 4.53 (dd, *J* = 5.8 Hz, 3.5 Hz, 1H), 4.27 (q, *J* = 4.5 Hz, 3.5 Hz, 1H), 4.15 (m, 2H), 2.54 (s, 3H); ^31^P NMR (202 MHz, D_2_O) δ 0.36 (t, *J* = 5.8 Hz, 1P); HRMS ESI (−) *m*/*z* [M – H]^−^, calcd for C_11_H_15_N_5_O_8_P^–^ 376.0669; found 376.0658.

##### 8-Phenyl-2′-*O*-methylguanosine 5′-Monophosphate, ^8Ph^m^2^′^*O*^GMP (**14b**)

^8Ph^m^2^′^*O*^GMP (**14b**) was obtained from ^8Br^m^2^′^*O*^GMP (**12**) (100 mg, 0.17 mmol) following general procedure A. Yield 68 mg, 60%. Rt (D) = 8.97 min; ^1^H NMR (500 MHz, D_2_O) δ 7.66 (m, 5H), 5.83 (d, *J* = 6.4 Hz, 1H), 5.01 (dd, *J* = 6.4 Hz, 5.6 Hz, 1H), 4.64 (dd, *J* = 5.6 Hz, 3.4 Hz, 1H), 4.19 (m, 3H), 3.30 (s, 3H); ^31^P NMR (202 MHz, D_2_O) δ 1.33 (t, *J* = 5.8 Hz, 1P). HRMS ESI (−) *m*/*z* [M – H]^−^, calcd for C_17_H_19_N_5_O_8_P^–^ 452.0977; found 452.0986.

##### 8-(4-Dimethylaminophenyl)-2′-*O*-methylguanosine 5′-Monophosphate, ^8DMAPh^m^2^′^*O*^GMP (**14c**)

^8DMAPh^m^2^′^*O*^GMP (**14c**) was obtained from ^8Br^m^2^′^*O*^GMP (**12**) (200 mg, 0.34 mmol) following general procedure A. Yield 154 mg, 71%. Rt (D) = 11.41 min; ^1^H NMR (500 MHz, D_2_O) δ 7.61 (d, *J* = 8.5 Hz, 2H), 7.13 (d, *J* = 8.5 Hz, 2H), 5.82 (d, *J* = 6.4 Hz, 1H), 5.01 (dd, *J* = 6.4 Hz, 5.6 Hz, 1H), 4.64 (dd, *J* = 5.6 Hz, 3.3 Hz, 2H), 4.23 (m, 2H), 4.16 (q, *J* = 6.7 Hz, 1H), 3.30 (s, 3H), 3.07 (s, 6H); ^31^P NMR (202 MHz, D_2_O) δ 1.38 (t, *J* = 5.8 Hz, 1P); HRMS ESI (−) *m*/*z* [M – H]^−^, calcd for C_19_H_24_N_6_O_8_P^–^ 495.1399; found 495.1408.

##### 8-(4-Cyanophenyl)-2′-*O*-methylguanosine 5′-Monophosphate, ^8PhCN^m^2^′^*O*^GMP (**14d**)

^8PhCN^m^2^′^-*O*^GMP (**14d**) was obtained from ^8Br^m^2^′^-*O*^GMP (**12**) (200 mg, 0.34 mmol) following general procedure A. Yield 120 mg, 51%. Rt (D) = 12.08 min; ^1^H NMR (500 MHz, D_2_O) δ 7.94 (d, *J* = 8.4 Hz, 2H), 7.84 (d, *J* = 8.4 Hz, 2H), 5.78 (d, *J* = 6.5 Hz, 1H), 5.06 (t, *J* = 6.5 Hz, 5.5 Hz, 1H), 4.66 (dd, *J* = 5.5 Hz, 3.3 Hz, 2H), 4.21 (m, 2H), 4.15 (m, 1H), 3.30 (s, 3H); ^31^P NMR (202 MHz, D_2_O) δ 1.60 (t, *J* = 5.7 Hz, 1P); HRMS ESI (−) *m*/*z* [M – H]^−^, calcd for C_18_H_18_N_6_O_8_P^–^ 477.0929; found 477.0939.

##### 8-Methyl-2′-*O*-methylguanosine 5′-Monophosphate, ^8Me^m^2^′^*O*^GMP (**14e**)

^8Me^m^2^′^*O*^GMP (**14e**) was obtained from ^8Br^m^2^′^*O*^GMP (**12**) (100 mg, 0.17 mmol) following general procedure A. Yield 59 mg, 88%. Rt (A) = 7.10 min; ^1^H NMR (500 MHz, D_2_O) δ 5.93 (d, *J* = 6.5 Hz, 1H), 4.89 (t, *J* = 6.5 Hz, 5.6 Hz, 1H), 4.71 (dd, *J* = 5.6 Hz, 3.6 Hz, 1H), 4.28 (q, *J* = 4.2 Hz, 1H), 4.16 (m, 2H), 3.41 (s, 3H), 2.59 (s, 3H); ^31^P NMR (202 MHz, D_2_O) δ 1.23 (t, *J* = 5.6 Hz, 1P); HRMS ESI (−) *m*/*z* [M – H]^−^, calcd for C_12_H_17_N_5_O_8_P^–^ 390.0820; found 390.0830.

##### 8-Cyclopropyl-2′-*O*-methylguanosine 5′-Monophosphate, ^8cPr^m^2^′^*O*^GMP (**14f**)

^8cPr^m^2^′^*O*^GMP (**14f**) was obtained from ^8-Br^m^2^′^-*O*^GMP (**12**) (200 mg, 0.17 mmol) following general procedure A. Yield 87 mg, 61%. Rt (A) = 9.13 min; ^1^H NMR (500 MHz, D_2_O) δ 6.20 (d, *J* = 6.7 Hz, 1H), 4.92 (m, 1H), 4.72 (dd, *J* = 5.6 Hz, 3.6 Hz, 1H), 4.29 (q, *J* = 3.8 Hz, 1H), 4.16 (m, 3H), 3.41 (s, 3H), 2.22 (td, *J* = 8.3 Hz, 4.2 Hz, 1H), 1.17 (dd, *J* = 8.3 Hz, 4.8 Hz, 2H), 1.10 (m, 1H), 1.01 (m, 1H); ^31^P NMR (202 MHz, D_2_O) δ 1.20 (t, *J* = 5.6 Hz, 1P); HRMS ESI (−) *m*/*z* [M – H]^−^, calcd for C_14_H_19_N_5_O_8_P^–^ 416.0977; found 416.0983.

### General Protocol for Methylation of 8-Substituted Monophosphates

The 8-substituted monophosphate analog (^**8R**^GMP or ^**8R**^m^2^′^*O*^GMP, TEA salt) was dissolved in a mixture of dry DMSO and DMF (1:1, v/v). After adding CH_3_I (8 equiv), the mixture was stirred at RT for 12–24 h until the starting material disappeared according to RP-HPLC analysis. The reaction was stopped by 10-fold dilution with water followed by triple extraction with diethyl ether. Then, approx. 0.5 mg of Na_2_S_2_O_5_ was added to the collected aqueous phase, and the pH was adjusted to 7 using NaHCO_3_ before purification on the DEAE Sephadex column. Ion-exchange purification afforded the triethylammonium salt ^8R^m^2^′^*O*,7^GMP (60–80%).

#### 2′-*O*,7-Dimethyl-8-phenyl)guanosine 5′-Monophosphate, ^8Ph^m^7,2^′^*O*^GMP (**15b**)

^8Ph^m^7,2^′^*O*^GMP (**15b**) was obtained from ^8Ph^m^2^′^*O*^GMP (**14b**) (60 mg, 0.12 mmol) following the general procedure. Yield 23 mg, 39%. Rt (D) = 10.12 min; ^1^H NMR (500 MHz, D_2_O) δ 7.85 (t, *J* = 7.4 Hz, 1H), 7.77 (t, *J* = 7.4 Hz, 2H), 7.72 (m, 2H), 5.68 (d, *J* = 5.7 Hz, 1H), 4.99 (dd, *J* = 5.7, 5.4 Hz, 1H), 4.69 (dd, *J* = 5.4 Hz, 4.1 Hz, 1H), 4.22–4.02 (m, 3H), 3.94 (s, 3H), 3.36 (s, 3H); ^31^P NMR (202 MHz, D_2_O) δ 2.02 (t, *J* = 5.5 Hz, 1P); HRMS ESI (−) *m*/*z* [M – H]^−^, calcd for C_18_H_21_N_5_O_8_P^–^ 466.1133; found 466.1141.

#### 2′-*O*,7-Dimethyl-8-(4-dimethylaminophenyl)guanosine 5′-Monophosphate, ^8DMAPh^m^7,2^′^*O*^GMP (**15c**)

^8DMAPh^m^7,2^′^*O*^GMP (**14c**) was obtained from ^8DMAPh^m^2^′^*O*^GMP (**14c**) (50 mg, 0.10 mmol) following the general procedure. Yield 42 mg, 81%. Rt (D) = 10.22 min; ^1^H NMR (500 MHz, DMSO-*d*_6_) δ 7.49 (d, *J* = 8.5 Hz, 2H), 6.93 (d, *J* = 8.5 Hz, 2H), 5.56 (d, *J* = 7.4 Hz, 1H), 4.94 (dd, *J* = 7.4 Hz, 4.0 Hz, 1H), 4.40 (d, *J* = 4.0 Hz, 1H), 4.36 (m, 1H), 4.11 (dd, *J* = 7.9 Hz, 3.8 Hz, 1H), 4.01 (m, 2H), 3.75 (s, 3H), 3.04 (s, 6H); ^31^P NMR (202 MHz, DMSO-*d*_6_) δ 0.45 (s, 1P); HRMS ESI (−) *m*/*z* [M – H]^−^, calcd for C_20_H_26_N_6_O_8_P^–^ 509.1555; found 509.1564.

#### 2′-*O*,7-Dimethyl-8-(4-cyanophenyl)guanosine 5′-Monophosphate, ^8PhCN^m^7,2^′^*O*^GMP (**15d**)

^8PhCN^m^7,2^′^*O*^GMP (**15d**) was obtained from ^8PhCN^m^2^′^*O*^GMP (**14d**) (50 mg, 0.10 mmol) following the general procedure. Yield 12 mg, 23%. Rt (D) = 7.07 min; ^1^H NMR (500 MHz, D_2_O) δ 8.14 (d, *J* = 8.8 Hz, 1H), 7.92 (bs, 2H), 5.63 (d, *J* = 5.9 Hz, 1H), 5.04 (dd, *J* = 5.9 Hz, 5.4 Hz, 1H), 4.70 (dd, *J* = 5.4 Hz, 3.8 Hz, 1H), 4.20 (m, 1H), 4.12 (m, 2H), 3.95 (s, 3H), 3.37 (s, 3H); ^31^P NMR (202 MHz, D_2_O) δ 1.72 (s, 1P); HRMS ESI (−) *m*/*z* [M – H]^−^, calcd for C_19_H_21_N_6_O_8_P^–^ 491.1086; found 491.1099.

#### (2′-*O*,7,8-Trimethyl)guanosine 5′-Monophosphate, m^7,2^′^*O*,8^GMP (**15e**)

m^7,2^′^*O*,8^GMP (**15e**) was obtained from ^8Me^m^2^′^*O*^GMP (**14e**) (50 mg, 0.13 mmol) following the general procedure. Yield 33 mg, 47%. Rt (A) = 5.93 min; ^1^H NMR (500 MHz, D_2_O) δ 6.08 (d, *J* = 6.1 Hz, 1H), 4.84 (dd, *J* = 6.1, 5.7 Hz, 1H), 4.76 (m, 1H), 4.30 (m, 1H), 4.09 (m, 2H), 4.05 (s, 3H), 3.43 (s, 3H), 2.82 (s, 3H); ^31^P NMR (202 MHz, D_2_O) δ 3.91 (m, 1P); HRMS ESI (−) *m*/*z* [M – H]^−^, calcd for C_13_H_19_N_5_O_8_P^–^ 404.0977; found 404.0985.

#### (2′-*O*,7-Dimethyl-8-cyclopropyl)guanosine 5′-Monophosphate, ^8cPr^m^7,2^′^*O*^GMP (**15f**)

^8cPr^m^7,2^′^*O*^GMP (**15f**) was obtained from ^8cPr^m^2^′^*O*^GMP (**14f**) (30 mg, 0.06 mmol) following the general procedure. Yield 11 mg, 38%. Rt (A) = 7.97 min; ^1^H NMR (500 MHz, D_2_O) δ 6.37 (d, *J* = 6.2 Hz, 1H), 5.10 (t, *J* = 6.2 Hz, 1H), 4.82–4.79 (m, 1H, overlapped with solvent signal), 4.33 (q, *J* = 6.2 Hz, 4.8 Hz, 1H), 4.18 (m, 2H), 4.13 (s, 3H), 3.44 (s, 3H), 2.10 (dt, *J* = 14.5 Hz, 9.5 Hz, 5.5 Hz, 1H), 1.49 (m, 2H), 1.18 (m, 2H); ^31^P NMR (202 MHz, D_2_O) δ 1.43 (t, *J* = 5.6 Hz, 1P); HRMS ESI (−) *m*/*z* [M – H]^−^, calcd for C_15_H_21_N_5_O_8_P^–^ 430.1133; found 430.1142.

#### 7-Methyl-8-(4-dimethylaminophenyl)guanosine 5′-Monophosphate, ^8DMAPh^m^7^GMP (**16c**)

^8DMAPh^m^7^GMP (**16c**) was obtained from ^8DMAPh^mGMP (**13c**) (260 mg, 0.38 mmol) following the general procedure. Yield 81.5 mg, 32%. Rt (D) = 9.41 min; ^1^H NMR (500 MHz, DMSO-*d*_6_) δ = 7.53 (d, *J* = 9.0 Hz, 2H), 7.04 (d, *J* = 9.0 Hz, 2H), 5.70 (d, *J* = 5.8 Hz, 1H), 5.29 (t, *J* = 5.8 Hz, 1H), 4.54 (dd, *J* = 5.8 Hz, 3.9 Hz, 1H), 4.19 (m, 1H), 4.12 (m, 2H), 3.93 (s, 3H), 3.07 (s, 6H); ^31^P NMR {1H BB} (162 MHz, Deuterium Oxide) δ = 1.14 (s, *1P*), ^31^P NMR δ = 1.14 (t, *J* = 5.7 Hz, 1P); HRMS ESI (−) *m*/*z* [M – H]^−^, calcd for C_19_H_24_N_6_O_8_P^–^ 495.1398; found 495.1400.

### General Procedure for Synthesizing 8-Substituted Cap Analogs via P-Imidazolides (**17**–**21**)

The following imidazolides were obtained using the Mukaiyama–Hashimoto procedure according to the literature:^66^ m^7^GDP-Im (**17**), m^2^′^-*O*,7^GDP-Im (**18**), GDP-Im (**19**), m^7^GTP-Im (**20**), and m^2,2,7^GDP-Im (**21**). The imidazolide (Na^+^ salt, 1.5 equiv) and suitable C8-substituted monophosphate (triethylammonium salt, 1 equiv) were suspended in anhydrous DMF (1.0 mL) followed by the addition of anhydrous ZnCl_2_ (95 mg, 0.7 mmol, 10 equiv). The reaction mixture was shaken vigorously until the reagents were dissolved. The reaction progress was monitored using RP-HPLC. After completion of the reaction (24 h), a Na_2_EDTA solution (237 mg, 0.7 mmol) was added to chelate zinc ions, and the pH was adjusted to 6 with solid NaHCO_3_. The products were purified using DEAE-Sephadex and isolated as triethylammonium salts. For NMR analysis and biological studies, a small sample was purified by semipreparative RP-HPLC and isolated as an ammonium salt.

#### P1-(7-Methyl-guanosin-5′-yl)-P3-(8-bromoguanosin-5′-yl) Triphosphate, m^7^GpppG^8Br^ (**1**)

m^7^GpppG^8Br^ (**1**) (4115 mOD, 0.18 mmol, 48%) was obtained from m^7^GDP-Im (**17**) (200 mg, 0.38 mmol) and ^8Br^GMP (**11**) (266 mg, 0.41 mmol) following the general procedure. RP-HPLC purification on a semipreparative RP-HPLC system gave the product as an ammonium salt. RP-HPLC: Rt (A) = 6.11 min; ^1^H NMR (500 MHz, D_2_O) δ 5.81 (d, *J* = 6.0 Hz, 1H), 5.79 (d, *J* = 3.9 Hz, 1H), 5.10 (dd, *J* = 6.0 Hz, 5.6 Hz, 1H), 4.54 (dd, *J* = 5.6 Hz, 3.9 Hz, 1H), 4.50 (dd, *J* = 5.0 Hz, 3.9 Hz, 1H), 4.42 (t, *J* = 5.0 Hz, 1H), 4.40–4.32 (m, 3H), 4.30–4.18 (m, 3H), 4.07 (s, 3H); ^31^P NMR (202 MHz, D_2_O) δ −10.63 to −10.39 (m, 2P), −22.21 (t, *J* = 19.4 Hz, 1P); HRMS ESI (−) *m*/*z* [M – H]^−^, calcd for C_21_H_27_BrN_10_O_18_P_3_^–^ [M – H]^−^ 878.9906, 880.9886; found 878.9920, 879.9917.

#### P1-(2′-*O*-7-Dimethyl-guanosin-5′-yl)-P3-(8-bromoguanosin-5′-yl) Triphosphate, m^2^′^*O*,7^GpppG^8Br^ (**2**)

m^2^′^*O*,7^GpppG^8Br^ (**2**) (2557 mOD, 0.11 mmol, 75%) was obtained from m^2^′^*O*,7^GDP-Im (**18**) (93 mg, 0.17 mmol) and ^8Br^GMP (**11**) (100 mg, 0.15 mmol) following the general procedure. RP-HPLC purification on a semipreparative RP-HPLC system gave the product as an ammonium salt. RP-HPLC: Rt (A) = 6.13 min; ^1^H NMR (500 MHz, deuterium oxide) δ 8.92 (s, 1H), 5.78 (m, 2H), 5.05 (t, *J* = 5.8 Hz, 1H), 4.50 (m, 2H), 4.38 (m, 2H), 4.25 (m, 4H), 4.16 (m, 1H), 4.08 (s, 3H), 3.55 (s, 3H); ^31^P NMR (202 MHz, deuterium oxide) δ −10.37 (m, 2P), −22.16 to −21.56 (m, 1P); HRMS ESI (−) *m*/*z* [M – H]^−^, calcd. *m*/*z* for C_22_H_29_BrN_10_O_18_P_3_^–^ [M – H]^−^ 893.0063, 895.0043; found 893.0068, 895.0046.

#### P1-(7-Methyl-guanosin-5′-yl)-P3-[8-(1-pyrene)guanosin-5′-yl] Triphosphate, m^7^GpppG^8Py^ (**3a**)

m^7^GpppG^8Py^ (**3a**) (410 mOD, 0.018 mmol, 57%) was obtained starting from m^7^GDP-Im (**17**) (19.5 mg, 0.035 mmol) and ^8Py^GMP (**13a**) (25 mg, 0.032 mmol) following the general procedure. Further purification on a semipreparative RP HPLC system gave the product as an ammonium salt. RP-HPLC: Rt (C) = 11.76 min; ^1^H NMR (500 MHz, deuterium oxide) δ 8.61 (s, 1H), 8.22–7.39 (m, 9H), 5.62 (s, 1H), 5.24 (s, 1H), 5.11–4.92 (m, 1H, overlapped with signal from HDO), 4.66–3.96 (m, 9H), 3.62–3.32 (m, 3H); ^31^P NMR (202 MHz, deuterium oxide) δ −10.12 to −10.76 (m, 2P), −21.80 to −22.16 (m, 1P); HRMS ESI (−) *m*/*z* [M – H]^−^, calcd for C_37_H_36_N_10_O_18_P_3_^–^ [M – H]^−^ 1001.1427; found 1001.1442.

#### P1-(7-Methyl-guanosin-5′-yl)-P3-(8-phenylguanosin-5′-yl) Triphosphate, m^7^GpppG^8Ph^ (**3b**)

m^7^GpppG^8Ph^ (**3b**) (530 mOD, 0.023 mmol, 47%) was obtained from m^7^GDP-Im (**17**) (27 mg, 0.051 mmol) and ^8Ph^GMP (**13b**) (30 mg, 0.05 mmol) following the general procedure. Further purification on a semipreparative RP-HPLC system gave the product as an ammonium salt. RP-HPLC: Rt (A) = 8.37 min; ^1^H NMR (500 MHz, deuterium oxide) δ 9.02 (s, 1H), 7.57 (m, 5H), 5.75 (d, *J* = 3.9 Hz, 1H), 5.72 (d, *J* = 6.1 Hz, 1H), 5.20 (dd, *J* = 6.1 Hz, 5.7 Hz, 1H), 4.48 (dd, *J* = 5.7 Hz, 3.5 Hz, 1H), 4.46 (dd, *J* = 4.9 Hz, 3.9 Hz, 1H), 4.39 (m, 3H), 4.24 (m, 4H), 4.07 (m, 3H); ^31^P NMR (202 MHz, deuterium oxide) δ −10.30 to −10.70 (m, 2P), −22.21 (t, *J* = 19.3 Hz, 1P); HRMS ESI (−) *m*/*z* [M – H]^−^, calcd for C_27_H_32_N_10_O_18_P_3_^–^ [M – H]^−^ 877.1114; found 877.1112.

#### P1-(7-Methyl-guanosin-5′-yl)-P3-[8-(4-dimethylaminophenyl)guanosin-5′-yl] Triphosphate, m^7^GpppG^8DMAPh^ (**3c**)

m^7^GpppG^8DMAPh^ (**3c**) (895 mOD, 0.04 mmol, 69%) was obtained from m^7^GDP-Im (**17**) (31 mg, 0.058 mmol) and ^8DMAPh^GMP (**13c**) (40 mg, 0.058 mmol) following the general procedure. Further purification on a semipreparative RP-HPLC system gave the product as an ammonium salt. RP-HPLC: Rt (A) = 11.07 min; ^1^H NMR (500 MHz, deuterium oxide) δ 8.97 (s, 1H), 7.35 (d, *J* = 8.4 Hz, 2H), 6.85 (d, *J* = 8.4 Hz, 2H), 5.71 (d, *J* = 5.1 Hz, 1H), 5.67 (d, *J* = 3.9 Hz, 1H), 5.11 (s, 1H), 4.56 (t, *J* = 5.1 Hz, 1H), 4.46–4.39 (m, 2H), 4.36 (t, *J* = 5.0 Hz, 1H), 4.32 (dt, *J* = 12.0 Hz, 3.4 Hz, 1H), 4.23 (m, 3H), 4.12 (m, 1H), 4.03 (s, 3H), 3.01 (s, 6H); ^31^P NMR (202 MHz, deuterium oxide) δ −10.20 to −10.70 (m, 2P), −22.19 (t, *J* = 19.4 Hz, 1P); HRMS ESI (−) *m*/*z* [M – H]^−^, calcd for C_29_H_37_N_11_O_18_P_3_^–^ [M – H]^−^ 920.1536; found 920.1532.

#### P1-(7-Methyl-guanosin-5′-yl)-P3-[8-(cyanophenyl)guanosin-5′-yl] Triphosphate, m^7^GpppG^8PhCN^ (**3d**)

m^7^GpppG^8PhCN^ (**3d**) (354 mOD, 0.016 mmol, 43%) was obtained from m^7^GDP-Im (**17**) (19.6 mg, 0.037 mmol) and ^8PhCN^GMP (**13d**) (25 mg, 0.037 mmol) following the general procedure. Further purification on a semipreparative RP-HPLC system gave the product as an ammonium salt. RP-HPLC: Rt (A) = 8.66 min; ^1^H NMR (500 MHz, deuterium oxide) δ 9.03 (s, 1H), 7.91 (d, *J* = 8.4 Hz, 2H), 7.73 (d, *J* = 8.4 Hz, 2H), 5.74 (d, *J* = 3.9 Hz, 1H), 5.68 (d, *J* = 6.0 Hz, 1H), 5.23 (t, *J* = 6.0 Hz, 1H), 4.52 (dd, *J* = 5.8 Hz, 3.9 Hz, 1H), 4.45 (t, *J* = 4.6 Hz, 4.0 Hz, 1H), 4.39 (m, 3H), 4.24 (m, 4H), 4.06 (s, 3H); ^31^P NMR (202 MHz, deuterium oxide) δ −10.32 to −10.67 (m, 2P), −22.18 (t, *J* = 19.3 Hz, 1P); HRMS ESI (−) *m*/*z* [M – H]^−^, calcd for C_28_H_31_N_11_O_18_P_3_^–^ [M – H]^−^ 902.1067; found 902.1071.

#### P1-(2′-*O*,7-Dimethyl-guanosin-5′-yl)-P3-[8-(1-pyrene)guanosin-5′-yl] Triphosphate, m^2^′^*O*,7^GpppG^8Py^ (**4a**)

m^2^′^*O*,7^GpppG^8Py^ (**4a**) (210 mOD, 0.009 mmol, 69%) was obtained from m^2^′^*O*,7^GDP-Im (**18**) (9.1 mg, 0.013 mmol) and ^8Py^GMP (**13a**) (10.0 mg, 0.013 mmol) following the general procedure. Further purification on a semipreparative RP-HPLC system gave the product as an ammonium salt. RP-HPLC: Rt (C) = 11.13 min; ^1^H NMR (500 MHz, deuterium oxide) δ 8.58 (s, 1H), 8.08 (d, *J* = 7.8 Hz, 1H), 7.97 (d, *J* = 7.8 Hz, 1H), 7.94–7.63 (m, 7H), 5.74 (s, 1H), 5.15 (s, 2H), 4.57 (s, 1H), 4.52–4.41 (m, 1H), 4.41–4.29 (m, 2H), 4.29–4.22 (m, 2H), 4.19 (dd, *J* = 11.0 Hz, 6.6, 1H), 4.12 (dd, 1H, *J* = 11.0 Hz, 6.6 Hz), 3.78 (s, 1H), 3.55 (s, 3H), 3.36 (s, 3H); ^31^P NMR (202 MHz, deuterium oxide) δ −10.26 (dt, *J* = 19.3 Hz, 7.1 Hz, 1P), −10.64 (d, *J* = 17.9 Hz, 1P), −22.16 (t, *J* = 19.3 Hz, 1P); HRMS ESI (−) *m*/*z* [M – H]^−^, calcd for C_38_H_38_N_10_O_18_P_3_^–^ [M – H]^−^ 1015.1584; found 1015.1582.

#### P1-(2′-*O*,7-Dimethyl-guanosin-5′-yl)-P3-(8-phenylguanosin-5′-yl) Triphosphate, m^2^′^*O*,7^GpppG^8Ph^ (**4b**)

m^2^′^*O*,7^GpppG^8Ph^ (**4b**) (545 mOD, 0.024 mmol, 78%) was obtained from m^2^′^*O*,7^GDP-Im (**18**) (17 mg, 0.031 mmol) and ^8Ph^GMP (**13b**) (20 mg, 0.031 mmol) following the general procedure. Further purification on a semipreparative RP-HPLC system gave the product as an ammonium salt. RP-HPLC: Rt (A) = 6.16 min; ^1^H NMR (500 MHz, deuterium oxide) δ 9.00 (s, 1H), 7.58 (m, 5H), 5.70 (d, *J* = 5.8 Hz, 1H), 5.69 (d, *J* = 2.8 Hz, 1H), 5.21 (t, *J* = 5.8 Hz, 1H), 4.48 (dd *J* = 5.8 Hz, 3.8 Hz, 1H), 4.46 (t, *J* = 5.8 Hz, 5.4 Hz, 1H), 4.44–4.38 (m, 2H), 4.26–4.18 (m, 5H), 4.08 (s, 3H), 3.50 (s, 3H); ^31^P NMR (202 MHz, deuterium oxide) δ −11.25 to −11.60 (m, 2P), −23.15 (t, *J* = 19.6 Hz, 1P); HRMS ESI (−) *m*/*z* [M – H]^−^, calcd for C_28_H_34_N_10_O_18_P_3_^–^ [M – H]^−^ 891.1271; found 891.1275.

#### P1-(2′-*O*,7-Dimethyl-guanosin-5′-yl)-P3-(8-methylguanosin-5′-yl) Triphosphate, m^2^′^*O*,7^GpppG^8Me^ (**4e**)

m^2^′^*O*,7^GpppG^8Me^ (**4e**) (370 mOD, 0.016 mmol, 43%) was obtained from m^2^′^*O*,7^GDP-Im (**18**) (21 mg, 0.038 mmol) and ^8Me^GMP (**13e**) (22 mg, 0.038 mmol) following the general procedure. Purification on a semipreparative RP-HPLC system gave the product as an ammonium salt. RP-HPLC: Rt (A) = 7.04 min; ^1^H NMR (500 MHz, deuterium oxide) δ 9.03 (s, 1H), 5.89 (d, *J* = 3.2 Hz, 1H), 5.74 (d, *J* = 6.5 Hz, 1H), 4.96 (t, *J* = 6.5 Hz, 6.0 Hz, 1H), 4.53 (dd, *J* = 6.0 Hz, 5.6 Hz, 1H), 4.50 (dd, *J* = 5.6 Hz, 3.4 Hz, 1H), 4.44–4.41 (m, 1H), 4.40–4.39 (m, 1H), 4.38–4.31 (m, 2H), 4.29–4.19 (m, 3H), 4.08 (s, 3H), 3.56 (s, 3H), 2.51 (s, 3H); ^31^P NMR (202 MHz, deuterium oxide) δ −10.50 to −10.66 (m, 2P), −22.24 (t, *J* = 19.4 Hz, 1P); HRMS ESI (−) *m*/*z* [M – H]^−^, calcd for C_23_H_32_N_10_O_18_P_3_^–^ [M – H]^−^ 829.1114; found 829.1102.

#### P1-[(2′-*O*,7-Dimethyl-8-phenyl)-guanosin-5′-yl]-P3-(guanosin-5′-yl) Triphosphate, ^8Ph^m^2^′^*O*,7^GpppG (**5b**)

^8Ph^m^2^′^*O*,7^GpppG (**5b**) (220 mOD, 0.009 mmol, 43%) was obtained starting from GDP-Im (**19**) (12.5 mg, 0.024 mmol) and ^8Ph^m^2^′^*O*,7^GMP (**15b**) (15 mg, 0.022 mmol) following the general procedure. Purification on a semipreparative RP-HPLC system gave the product as an ammonium salt. RP-HPLC: Rt (A) = 9.38 min; ^1^H NMR (500 MHz, deuterium oxide) δ 8.42 (s, 1H), 7.83 (m, 1H), 7.74 (m, 2H), 7.68 (m, 2H), 5.89 (d, *J* = 5.2 Hz, 1H), 5.62 (d, *J* = 5.8 Hz, 1H), 4.96 (dd, *J* = 6.2 Hz, 5.2 Hz, 1H), 4.72 (dd, *J* = 5.8 Hz, 3.3 Hz, 1H), 4.70 (dd, *J* = 5.6 Hz, 5.2 Hz, 1H), 4.50 (dd, *J* = 5.2 Hz, 3.3 Hz, 1H), 4.32 (m, 3H), 4.22 (m, 3H), 3.93 (s, 3H), 3.33 (s, 3H); ^31^P NMR (202 MHz, deuterium oxide) δ −10.30 to −10.70 (m, 2P), −22.25 (t, *J* = 19.6 Hz, 1P); HRMS ESI (−) *m*/*z* [M – H]^−^, calcd for C_28_H_34_N_10_O_18_P_3_^–^ [M – H]^−^ 891.1271; found 891.1288.

#### P1-[(2′-*O*,7-Dimethyl-8-(4-dimethylaminophenyl)-guanosin-5′-yl]-P3-(guanosin-5′-yl) Triphosphate, ^8DMAPh^m^2^′^*O*,7^GpppG (**5c**)

^8DMAPh^m^2^′^*O*,7^GpppG (**5c**) (375 mOD, 0.016 mmol, 43%) was obtained from GDP-Im (**19**) (21 mg, 0.04 mmol) and ^8DMAPh^m^2^′^*O*,7^GMP (**15c**) (446 mOD, 12.9 mg, 0.037 mmol) following the general procedure. Purification on a semipreparative RP-HPLC system gave the product as an ammonium salt. RP-HPLC: Rt (B) = 13.44 min; ^1^H NMR (500 MHz, deuterium oxide) δ 8.10 (bs, 1H), 7.43 (d, *J* = 8.6 Hz, 2H), 6.91 (d, *J* = 8.6 Hz, 2H), 5.72 (d, *J* = 6.3 Hz, 1H), 5.68 (d, *J* = 5.7 Hz, 1H), 5.00 (t, *J* = 5.7 Hz, 1H), 4.76 (dd, *J* = 5.7 Hz, 3.9 Hz, 1H, overlapped with solvent signal), 4.66 (dd, *J* = 6.3 Hz, 5.1 Hz, 1H), 4.43 (dd, *J* = 5.1 Hz, 3.1 Hz, 1H), 4.39 (m, 1H), 4.24 (m, 3H), 4.17 (m, 1H), 4.08 (m, 1H), 3.98 (s, 3H), 3.32 (s, 3H), 3.05 (s, 6H); ^31^P NMR (202 MHz, deuterium oxide) δ −10.22 to −10.70 (m, 2P), −22.00 to −22.58 (m, 1P); HRMS ESI (−) *m*/*z* [M – H]^−^, calcd for C_30_H_39_N_11_O_18_P_3_^–^ [M – H]^−^ 934.1693; found 934.1697.

#### P1-[(2′-*O*,7-Dimethyl-8-(4-cyanophenyl)-guanosin-5′-yl]-P3-(guanosin-5′-yl) Triphosphate, ^8PhCN^m^2^′^*O*,7^GpppG (**5d**)

^8PhCN^m^2^′^*O*,7^GpppG (**5d**) (333 mOD, 0.015 mmol, 56%) was obtained starting from GDP-Im (**19**) (14 mg, 0.027 mmol) and ^8PhCN^m^2^′^*O*,7^GMP (**15d**) (280 mOD, 15.6 mg, 0.023 mmol) following the general procedure. Purification on a semipreparative RP-HPLC system gave the product as an ammonium salt. RP-HPLC: Rt (B) = 8.28 min; ^1^H NMR (500 MHz, deuterium oxide) δ 8.14–8.10 (m, 2H), 8.07 (s, 1H), 7.98–7.84 (m, 2H), 5.83 (d, *J* = 6.2 Hz, 1H), 5.56 (d, *J* = 6.0 Hz, 1H), 5.04 (dd, *J* = 6.0 Hz, 5.2 Hz, 1H), 4.77–4.73 (m, 2H), 4.51 (dd, *J* = 5.2 Hz, 2.8 Hz, 1H), 4.37 (m, 2H), 4.25 (m, 4H), 3.94 (s, 3H), 3.35 (s, 3H); ^31^P NMR (202 MHz, deuterium oxide) δ −10.42 to −10.70 (m, 2P), −22.25 (t, *J* = 19.5 Hz, 1P); HRMS ESI (−) *m*/*z* [M – H]^−^, calcd for C_29_H_33_N_11_O_18_P_3_^–^ [M – H]^−^ 916.1223; found 916.1225.

#### P1-[(2′-*O*,7-Dimethyl-8-methyl)-guanosin-5′-yl]-P3-(guanosin-5′-yl) Triphosphate, ^8Me^m^2^′^*O*,7^GpppG (**5e**)

^8Me^m^2^′^*O*,7^GpppG (**5e**) (768 mOD, 0.034 mmol, 55%) was obtained from GDP-Im (**19**) (35.0 mg, 0.068 mmol) and ^8Me^m^2^′^*O*,7^GMP (**15e**) (744 mOD, 37.5 mg, 0.062 mmol) following the general procedure. Purification on a semipreparative RP-HPLC system gave the product as an ammonium salt. RP-HPLC: Rt (A) = 5.88 min; ^1^H NMR (500 MHz, deuterium oxide) δ 7.99 (s, 1H), 5.96 (d, *J* = 6.2 Hz, 1H), 5.80 (d, *J* = 5.6 Hz, 1H), 4.69 (m, 1H), 4.64 (m, 2H), 4.46 (m, 1H), 4.27 (m, 6H), 4.01 (s, 3H), 3.39 (s, 3H), 2.75 (s, 3H); ^31^P NMR (202 MHz, deuterium oxide) δ −9.72 to −10.71 (m, 2P), −20.60 to −21.65 (m, 1P); HRMS ESI (−) *m*/*z* [M – H]^−^, calcd for C_23_H_32_N_10_O_18_P_3_^–^ [M – H]^−^ 829.1114; found 829.1117.

#### P1-[(2′-*O*,7-Dimethyl-8-cyclopropyl)-guanosin-5′-yl]-P3-(guanosin-5′-yl) Triphosphate, ^8cPr^m^2^′^*O*,7^GpppG (**5f**)

^8cPr^m^2^′^*O*,7^GpppG (**5f**) (223 mOD, 0.01 mmol, 56%) was obtained from GDP-Im (**19**) (10 mg, 0.019 mmol) and ^8cPr^m^2^′^*O*,7^GMP (**15f**) (270 mOD, 11.3 mg, 0.018 mmol) following the general procedure. Purification on a semipreparative RP-HPLC system gave the product as an ammonium salt. RP-HPLC: Rt (A) = 7.28 min; ^1^H NMR (500 MHz, deuterium oxide) δ 8.05 (s, 1H), 6.30 (d, *J* = 6.7 Hz, 1H), 5.83 (d, *J* = 6.2 Hz, 1H), 5.05 (dd, *J* = 6.7 Hz, 5.2 Hz, 1H), 4.85–4.77 (m, 2H, overlapped with solvent signal), 4.51 (dd, *J* = 5.5 Hz, 2.8 Hz, 1H), 4.40 (dt, *J* = 10.8 Hz, 6.7 Hz, 1H), 4.33–4.26 (m, 3H), 4.25–4.19 (m, 2H), 4.09 (s, 3H), 3.41 (s, 3H), 2.09–2.02 (m, 1H), 1.51–1.44 (m, 2H), 1.20–1.12 (m, 2H); ^31^P NMR (202 MHz, deuterium oxide) δ −10.40 to −10.72 (m, 2P), −22.10 to −21.45 (m, 1P); HRMS ESI (−) *m*/*z* [M – H]^−^, calcd for C_25_H_34_N_10_O_18_P_3_^–^ [M – H]^−^ 855.1271; found 855.1274.

#### P1-[8-(4-Dimethylaminophenyl)-7-methylguanosine-5′-yl)-P3-(7-methylguanosine-5′-yl] Triphosphate, ^8DMAPh^m^7^Gpppm^7^G (**6**)

^8DMAPh^m^7^Gpppm^7^G (**6**) (65 mOD, 0.003 mmol, 41%) was obtained from m^7^GDP-Im (**17**) (3.8 mg, 0.007 mmol) and ^8DMAPh^m^7^GMP (**16c**) (5 mg, 0.007 mmol) following the general procedure. Purification on a semipreparative RP-HPLC system gave the product as an ammonium salt. RP-HPLC: Rt (A) = 12.44 min; ^1^H NMR (500 MHz, deuterium oxide) δ 7.49 (d, *J* = 8.6 Hz, 2H), 6.99 (d, *J* = 8.6 Hz, 2H), 5.93 (d, *J* = 4.2 Hz, 1H), 5.66 (d, *J* = 5.6 Hz, 1H), 5.26 (t, *J* = 5.6 Hz, 1H), 4.60–4.58 (m, 2H), 4.47 (t, *J* = 4.8 Hz, 1H), 4.39–4.32 (m, 2H), 4.23 (m, 4H), 4.12 (s, 3H), 3.99 (s, 3H), 3.07 (s, 6H); ^31^P NMR (202 MHz, deuterium oxide) δ −10.40 to −10.60 (m, 2P), −22.22 (t, *J* = 19.7 Hz, 1P); HRMS ESI (−) *m*/*z* [M – H]^−^, calcd for C_30_H_39_N_11_O_18_P_3_^–^ [M – H]^−^ 934.1693; found 934.1704.

#### P1-[8-(4-Dimethylaminophenyl)-7-methylguanosine-5′-yl)-P4-(7-methylguanosine-5′-yl] Tetraphosphate, ^8DMAPh^m^7^Gppppm^7^G (**7**)

^8DMAPh^m^7^Gppppm^7^G (**7**) (84 mOD, 0.004 mmol, 27%) was obtained starting from m^7^GTP-Im (**20**) (8.7 mg, 0.014 mmol) and ^8DMAPh^m^7^GMP (**16c**) (10 mg, 0.014 mmol) following the general procedure. Purification on a semipreparative RP-HPLC system gave the product as an ammonium salt. RP-HPLC: Rt (A) = 11.90 min; ^1^H NMR (500 MHz, deuterium oxide) δ 9.25 (s, 1H), 7.53 (d, *J* = 8.3 Hz, 2H), 7.04 (d, *J* = 8.3 Hz, 2H), 6.00 (d, *J* = 4.2 Hz, 1H), 5.68 (d, *J* = 5.9 Hz, 1H), 5.33 (t, *J* = 5.9 Hz, 1H), 4.69–4.65 (m, 2H), 4.52 (t, *J* = 4.7 Hz, 1H), 4.40–4.30 (m, 3H), 4.28–4.20 (m, 3H), 4.12 (s, 3H), 3.96 (s, 3H), 3.07 (s, 6H); ^31^P NMR (202 MHz, deuterium oxide) δ −10.30 to −10.60 (m, 2P), −22.15 to −22.15 (m, 2P); HRMS ESI (−) *m*/*z* [M – H]^−^, calcd for C_30_H_40_N_11_O_21_P_4_^–^ [M – H]^−^ 1014.1356; found 1014.1368.

#### P1-[8-(4-Dimethylaminophenyl)-7-methylguanosine-5′-yl)-P3-(2*N*,2*N*-*N*7-trimethyl-guanosine-5′-yl] Triphosphate, ^8DMAPh^m^7^Gpppm^2,2,7^G (**8**)

^8DMAPh^m^7^Gpppm^2,2,7^G (**8**) (160 mOD, 0.006 mmol, 43%) was obtained from m^2,2,7^GDP-Im (**21**) (8.0 mg, 0.014 mmol) and ^8DMAPh^m^7^GMP (**16c**) (10 mg, 0.014 mmol) following the general procedure. Purification on a semipreparative RP-HPLC system gave the product as an ammonium salt. RP-HPLC: Rt (A) = 13.45 min; ^1^H NMR (500 MHz, deuterium oxide) δ 7.47 (d, *J* = 8.6 Hz, 2H), 6.98 (d, *J* = 8.6 Hz, 2H), 5.96 (d, *J* = 4.1 Hz, 1H), 5.64 (d, *J* = 5.6 Hz, 1H), 5.24 (t, *J* = 5.6 Hz, 1H), 4.63–4.59 (m, 2H), 4.45 (t, *J* = 4.7 Hz, 1H), 4.35 (m, 3H), 4.22 (m, 3H), 4.11 (s, 3H), 4.00 (s, 3H), 3.15 (s, 6H), 3.07 (s, 6H); ^31^P NMR (202 MHz, deuterium oxide) δ −10.44 to −10.71 (m, 2P), −22.21 (t, *J* = 19.1 Hz, 1P); HRMS ESI (−) *m*/*z* [M – H]^−^, calcd for C_32_H_43_N_11_O_18_P_4_^–^ [M – H]^−^ 962.2006; found 962.2016.

## Biophysical Studies

### Thermal Stability of the m^7^GpppG^8Br^ Cap Analog at Various pHs

The thermal stability of compound **1** was tested in three 100 mM NaHCO_3_ buffers at pH 7, 8.0, and 8.5 (Figure S1, SI). A stock solution of **1** (50 mM) was prepared in deionized water, stored at −20 °C, and used for preparing working solutions (50 μM) in the described buffers. The solutions were incubated in a thermomixer at 60, 70, 80, and 90 °C for 15 min. The unmodified cap analog m^7^GpppG was used as a reference. RP-HPLC measurements (Method D, Experimental Section, Chromatography, Analytical and Preparative RP-HPLC) were performed using a Shimadzu RP HPLC system equipped with an autosampler. The cap analog m^7^GpppG^8Br^ at time 0 was used as a reference.

### Chemical Stability of Cap Analogs at Various pHs

The chemical stability of the 8-modified cap analogs (Figure S15*,* SI) was tested in the following buffers (pH 3–10): pH 3, 100 mM sodium citrate buffer; pH 5, 100 mM ammonium acetate buffer; pH 6, 100 mM potassium phosphate buffer; pH 7, 100 mM potassium phosphate buffer; and pH 10, 100 mM ammonium chloride buffer. Stock solutions of the compounds (5 mM) were prepared in deionized water, stored at −20 °C, and mixed with various buffers to prepare the working solutions (50 μM). After incubating the working solutions at 37 °C for 5 h, RP-HPLC analysis was performed using an Agilent RP HPLC system equipped with an autosampler.

### Photophysical Properties

Absorption spectra were recorded on a Shimadzu UV-1800 spectrophotometer, and fluorescence spectra were recorded on a Cary Eclipse (Agilent) spectrofluorometer in 0.1 M potassium phosphate buffer at 25 °C. The concentration of cap analogs was 5 μM.

#### Fluorescence Anisotropy

Fluorescence anisotropy was measured using a BioTek Synergy H1 microplate reader equipped with polarizing filters (excitation 485 ± 20 nm, emission 528 ± 20 nm). Black nonbinding 96-well plates were used for the experiments. To estimate the affinity of C8-modified cap analogs for eIF4E protein, a competition experiment was performed as described previously^53^ with minor modifications. For the screening experiment, 10 nM of the m^7^Gppp-triazol-(6)FAM (compound **1a** from ref (53), Figure S18) probe was used together with 100 nM of eIF4E and 500 nM of the tested compound. The reaction with deionized water instead of the compound served as a negative control. For the selected cap analogs, the EC_50_ parameter was determined using the same probe and protein concentrations, except for the ligand concentration. For each EC_50_, a 12-point serial dilution of the tested compound was used.

#### Changes in Fluorescence Intensity Due to eIF4E Binding

To verify whether the fluorescence intensity of C8-modified cap analogs changed upon binding to eIF4E, a solution of cap analog (500 nM) was incubated with increasing concentrations of eIF4E (0, 0.1, 0.2, 0.5, and 1 μM) in black 96-well plates for 10 min at 25 °C. The experiment was performed in a 2-[4-(2-hydroxyethyl)piperazin-1-yl]ethane-1-sulfonic acid (HEPES)/KOH (50 mM, pH 7.2) buffer containing 100 mM KCl, 0.5 mM EDTA, and 1 mM dithiothreitol (DTT). The excitation wavelength was 315 or 346 nm, and fluorescence was recorded at 400 or 480 nm using a BioTek Synergy H1 microplate reader.

For selected compounds, fluorescent titration experiments were performed to determine the affinity of the cap analogs to eIF4E_._ The experiments were performed in a quartz cuvette at 25 °C using a Cary Eclipse (Agilent) spectrofluorometer. To determine the *K*_d_ value, a solution of cap analog (100 nM of m^7^GpppG^8DMAPh^, 50 nM of m^7^GpppG^8Ph^, or 50 nM of m^7,2^′^*O*^GpppG^8Ph^) was thermostated for 10 min. After adding each 1 μL aliquot of protein solution, the fluorescence spectrum was recorded. The excitation/emission wavelengths were 315/415 nm for m^7^GpppG^8DMAPh^ and 315/391 nm for m^7^GpppG^8Ph^ and m^7,2^′^*O*^GpppG^8Ph^. Control experiments with m^7^GpppG^8DMAPh^ and bovine serum albumin(BSA) were performed under the same conditions.

#### Fluorescence Quenching Titration Assay with Snurportin

Titration experiments were performed in a standard buffer of 50 mM HEPES/NaOH (pH 7.2) containing 150 mM NaCl, 1 mM EDTA, and 2 mM DTT. The pH (±0.01) was measured independently at each temperature and ionic strength (SevenCompact pH meter S220, Mettler Toledo, Switzerland). The sample was thermostated at 19.8–20 °C, and the temperature was controlled with a thermocouple inside the cuvette (±0.2 °C). For the snurportin1–ligand association, an excitation wavelength of 280 nm (slit 10 nm, auto cutoff filter) and an emission wavelength of 345 nm (slit 10 nm, 290 nm cutoff filter) were applied with correction for the photomultiplier sensitivity. These conditions ensured that only emission from tryptophan residues in the protein was observed. The fluorescence intensity was monitored during continuous time synchronized titration (TST) at a single wavelength with an integration time of 30 s and a gap of 30 s for adding the ligand. The solution was slowly but sufficiently stirred magnetically to ensure mixing and constant temperature throughout the volume. During the gap, the UV xenon flash lamp was switched off to avoid photobleaching the sample. Titration was performed for 0.1 μM snurportin concentration under steady-state conditions provided by preincubation in the buffer. Aliquots of the ligand at increasing concentrations (1 μM to 1 mM) were added to 1400 μL of snurportin solution. Suitable data correction was applied when the final dilution was ≥2% (but always ≤4%).

#### Numerical Data Analysis of FQT Experiments

The following theoretical curve for the fluorescence intensity (*F*) as a function of ligand concentration [*L*] was used to fit the experimental data:
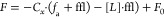
where the equilibrium concentration of the cap-snurportin1 complex *C_x_* is given by
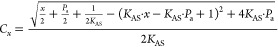


The fitted parameters are as follows: *K*_AS_ (association constant), *P*_a_ (concentration of the active protein), *f*_a_ (fluorescence efficiency of the active protein), ffl (fluorescence efficiency of the free cap analog in the solution), and *F*_0_ (initial fluorescence intensity). The last two parameters were independently verified by experiments. The total quenching is calculated as follows:
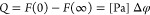


The fluorescence intensities (*F*) were corrected for the inner-filter effect. This effect was negligible for the specific cap analogs but could change the *K*_AS_ values by approximately twofold for the weakly interacting and strongly absorbing cap analogs. The final *K*_AS_ was calculated as the weighted average of three independent titration series. The results were consistent within 10%.

Regressions were performed using a nonlinear least-squares method *via* OriginPro 8.5.0 SR1 (Microcal Software Inc., USA).

#### Saturation Binding Experiment for ^8DMAPh^m^7^Gpppm^2,2,7^G (8) and Snurportin

A concentrated solution of compound **8** (1 μM) was prepared in HEPES buffer (50 mM HEPES, 150 mM NaCl, and 1 mM EDTA, pH 7.2). The emission spectrum of unbound ligand was recorded. Aliquots of 30 μM snurportin in HEPES buffer (50 mM HEPES, 150 mM NaCl, 0.5 mM EDTA, 2 mM DTT, and 10% glycerol, pH 7.5) were added to the ligand solution in the fluorescence cuvette and mixed using a magnetic stirrer. After adding each aliquot of protein solution and waiting for 30 s, the solution was excited at 350 nm, and the emission spectrum was recorded.

#### Enzymatic Degradation (Susceptibility to DcpS and Nudt16 Hydrolysis)

The enzymatic activity of DcpS was determined at 30 °C in 50 mM Tris/HCl (pH 7.6) containing 200 mM KCl, 0.5 mM EDTA, and 1 mM DTT. The hydrolytic activity of Nudt16 was assayed at 37 °C in 40 mM Tris (pH 7.9) containing 10 mM NaCl, 6 mM MgCl_2_, and 2 mM DTT. The reaction components were thermostated for 10 min, and then the enzyme was added to start the reaction. At specific times, 120 μL aliquots were taken out, and the enzyme therein was inactivated by heating at 98 °C for 2.5 min and measured by analytical HPLC (Agilent Technologies 1200 Series, Santa Clara, CA, USA). After sample injection, the HPLC column was eluted with a linear gradient of methanol (0–50%) in aqueous 0.1 M KH_2_PO_4_ over 15 min at a flow rate of 1.3 mL/min. The specific conditions are as follows: DcpS assay: 20 μM cap analog, 28 nM DcpS, sampled at 0, 5, 15, and 30 min and Nudt16 assay: 20 μM cap analog, 710 nM Nudt16, sampled at 0, 15, 30, and 60 min.

#### Determination of Inhibitory Properties Using HTS Fluorescence-Based Assay

The enzymatic assays were performed in 96-well black nonbinding assay plates. Enzymatic reactions were carried out in 50 mM Tris/HCl buffer (pH 7.6) containing 200 mM KCl, 0.5 mM EDTA, and 0.75 mg/mL BSA. The total volume of the reaction mixture was 200 μL. In the screening experiment, m^7^GMPF (60 μM) was used as the substrate and tested compound at 20 μM in the presence of 30 nM DcpS. The reaction components were preincubated for 10–15 min at 30 °C. After adding DcpS, the sample and enzyme were incubated at 30 °C for 55 min and then quenched using 100 μL of acetonitrile. Aliquots of 25 μL were transferred to a new plate. Freshly prepared fluorogenic probe solution (TBS-fluorescein, FTBS, 90 μL) was added to each well. The probe solution was freshly prepared before each experiment. To prepare, FTBS (3.0 mg, 5.36 μmol) was dissolved in 50 μL of ethyl acetate and diluted with 10 mL of a 9:1 (v/v) mixture of DMSO and aqueous Tris buffer (50 mM Tris/HCl, 0.5 mM EDTA, 200 mM KCl, pH 7.6) to a final FTBS concentration of 5.3 μM. The samples with fluorogenic probe were incubated for 60 min at 30 °C. Then, 100 μL of HEPES buffer (200 mM, pH 7.0) was added followed by a fluorescence readout (excitation at 480 nm and emission at 535 nm).

#### Fluorescence Intensity Change during Hydrolysis by DcpS

The experiment was performed in black 96-well plates at 25 °C in 50 mM Tris/HCl buffer containing 200 mM KCl, 0.5 mM EDTA, and 1 mM DTT (pH 7.6). The reaction components were thermostated for 10 min before enzyme addition (20 μM substrate and 28 nM DcpS). The fluorescence intensity was recorded every 1 min using a Biotek Synergy H1 microplate reader. The excitation and emission wavelengths were 280/400 nm for the Ph-modified compound, 288/414 nm for the DMAPh-modified cap analogs, 346/480 nm for the Py-modified compounds, and 315/466 nm for the PhCN-modified cap analogs.

#### *In Vitro* Transcription of mRNA and Determination of Capping Efficiency

mRNAs capped with C8-modified compounds and encoding firefly and *Renilla* luciferase were synthesized by *in vitro* transcription with SP6 or T7 polymerase. pJET_luc_128A and hRLuc-pRNA2(A)128, both digested with the restriction enzyme AarI (Thermo Fisher Scientific), were used as templates to obtain firefly and *Renilla* mRNA, respectively. In the typical *in vitro* transcription, we incubated 20 μL of RNA Pol buffer containing the following components: 1 U/μL SP6 polymerase or 1 U/μL T7 polymerase (New England BioLabs) to respectively synthesize firefly or *Renilla* mRNA, 1 U/μL RiboLock RNase inhibitor, 0.5 mM ATP/CTP/UTP, 0.125 mM GTP, 1.25 mM cap analog of interest, and 5 μg/μL digested plasmid as a template. The mRNA encoding *Renilla* luciferase was always capped with ARCA (m_2_^7,3^′^*O*^GpppG). Following 2 h of incubation, 1 U/μL DNase I (Ambion) was added, and incubation was continued for 30 min at 37 °C, after which EDTA was added to reach a final concentration of 25 μM. The obtained mRNAs were purified using NucleoSpin RNA Clean-up XS (Macherey-Nagel). The quality of the transcripts was checked on a native 1.2% agarose gel, and the concentration was determined by spectrophotometry. The capping efficiencies of *in vitro* synthesized RNAs achievable with the new analogs were determined as described previously.^10a^

#### Measurement of Translation Efficiency

To measure translation efficiency in cell culture, mRNAs encoding firefly and *Renilla* luciferase were synthesized by *in vitro* transcription as described above. HeLa cells were grown at 5% CO_2_ and 37 °C in Dulbecco’s modified Eagle medium (Gibco) supplemented with GlutaMAX (Gibco), 10% fetal bovine serum (Sigma), and 1% penicillin/streptomycin (Gibco). In a typical experiment, 24 h before transfection, 10^4^ grown cells were seeded in 100 μL of medium without antibiotics in each well of a 96-well plate. Then, cells in each well were transfected for 1 h using 0.3 μL Lipofectamine MessengerMAX Transfection Reagent (Invitrogen), 0.1 μg mRNA, and 10 μL Opti-Mem (Gibco). The mRNA used for transfection was a mixture of mRNA encoding firefly luciferase (70 ng) with a cap analog of interest at the 5′ end and mRNA encoding *Renilla* luciferase (30 ng) capped with m_2_^7,3^′^*O*^GpppG as a transfection efficiency control. The transfected cells were washed with phosphate-buffered saline, supplemented with fresh medium without antibiotics, and grown for the indicated times. Cell lysis was performed using the Dual-Luciferase Reporter Assay System (Promega), and activities of firefly and *Renilla* luciferase were measured sequentially using a Synergy H1 microplate reader (Biotek). The obtained firefly luminescence was normalized as described previously^7^ to account for transfection efficiency according to the formula *F*/*R*·*R*_AVG_ (*F* = firefly luminescence measured for the chosen well, *R* = *Renilla* luminescence measured for the same well as *F*, *R*_AVG_ = average *Renilla* luminescence measured for all transfections at the indicated time point). After plotting the normalized firefly luminescence as a function of time, the total protein expression was calculated as the integrated area under the line segments.

## Data Availability

The data underlying this study are available in the published article and its Supporting Information.

## Supporting Information Available

Copies of ^1^H, ^31^P NMR, ESI HRMS, UV/VIS/FLU spectra, and RP-HPLC chromatograms (PDF)
