# Supplementary material for: Synthesis and Evaluation of Diguanosine Cap Analogs Modified at the C8-Position by Suzuki–Miyaura Cross-Coupling: Discovery of 7-Methylguanosine-Based Molecular Rotors
Source: J Org Chem. 2023 May 20;88(11):6827–46. doi: 10.1021/acs.joc.3c00126 (PMC10242767; doi:10.1021/acs.joc.3c00126)
Supplement: Supplementary file 1 — jo3c00126_si_001.pdf [file jo3c00126_si_001.pdf]

Synthesis and Evaluation of Diguanosine Cap Analogs Modified at the C8-Position by Suzuki-Miyaura Cross-Coupling: Discovery of 7-Methylguanosine-Based Molecular Rotors

Blazej A. Wojtczak[1†], Marcelina Bednarczyk[1,2†], Pawel J. Sikorski[1], Anna Wojtczak[2], Piotr Surynt[1,2], Joanna Kowalska[2], Jacek Jemielity[1,*]

[1] Centre of New Technologies, University of Warsaw; S. Banacha 2c, 02-097 Warsaw, Poland

[2] Faculty of Physics, University of Warsaw; L. Pasteura 5, 02-093, Warsaw, Poland

***** Correspondence: j.jemielity@cent.uw.edu.pl; Tel.: +48-22-55-43-774

† These authors contributed equally

Supporting Info

Table of contents

1. Chemical synthesis (Scheme S1, Figure S1-S15)...S2

2. Photophysical properties (Figure S16-S17)...S17

3. Biophysical studies (Figure S18-S20)...S18

4. Tables...S21

5. Compounds characterization (HPLC profiles, NMR and HRMS spectra) S24

1. Chemical synthesis (Scheme S1, Figure S1-S15)

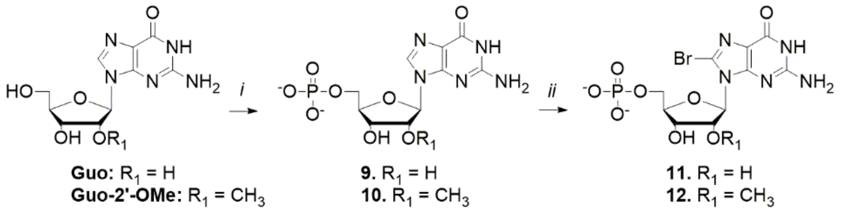

Scheme S1. Synthesis of 8-bromoguanosine 5′-monophosphates (**11**, **12**). Reagents and conditions: (i) $POCl_3$, $PO(OMe)_3$, 0 °C, 3h; ii) Br_2 (aq), NaOAc, pH 4.0 or NBS/DMF.

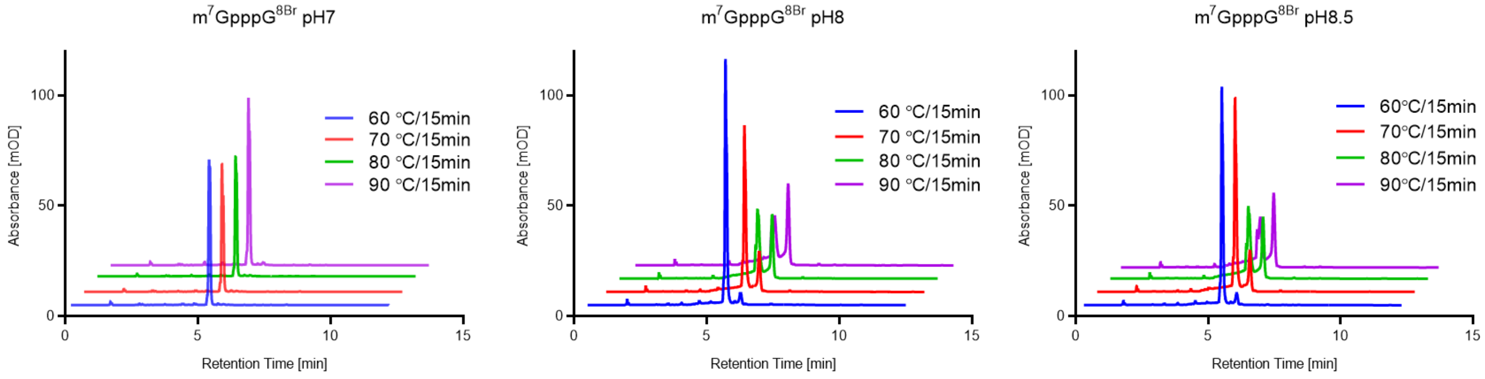

Figure S1. Stability of cap analog m^{7}GpppG^{8Br} (**1**) at various pH and temperatures.

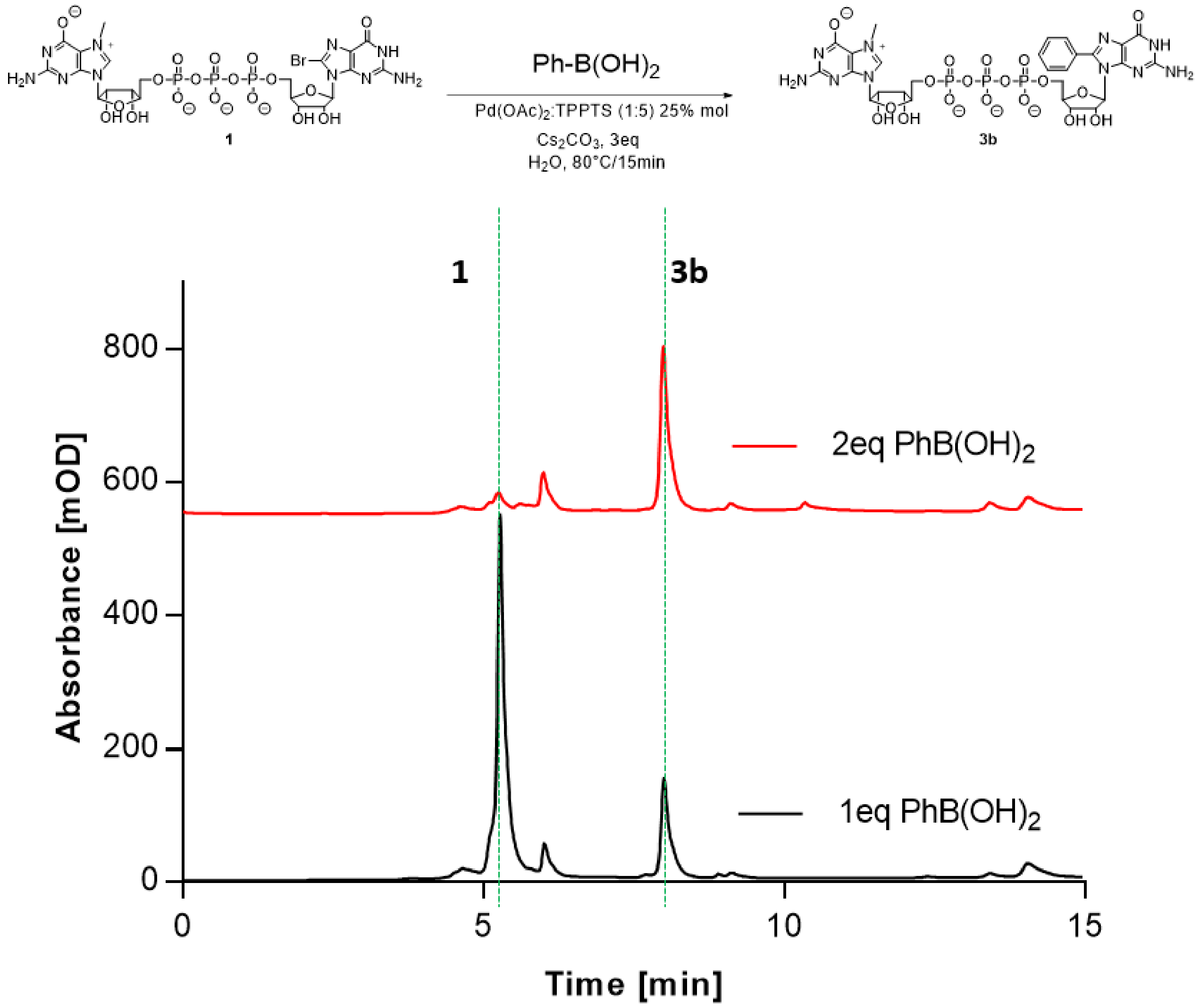

Figure S2. (C18) RP-HPLC reaction profiles of m^{7}GpppG^{8Ph} (**3b**) synthesis depending on the amount of phenylboronic acid used in the reaction.

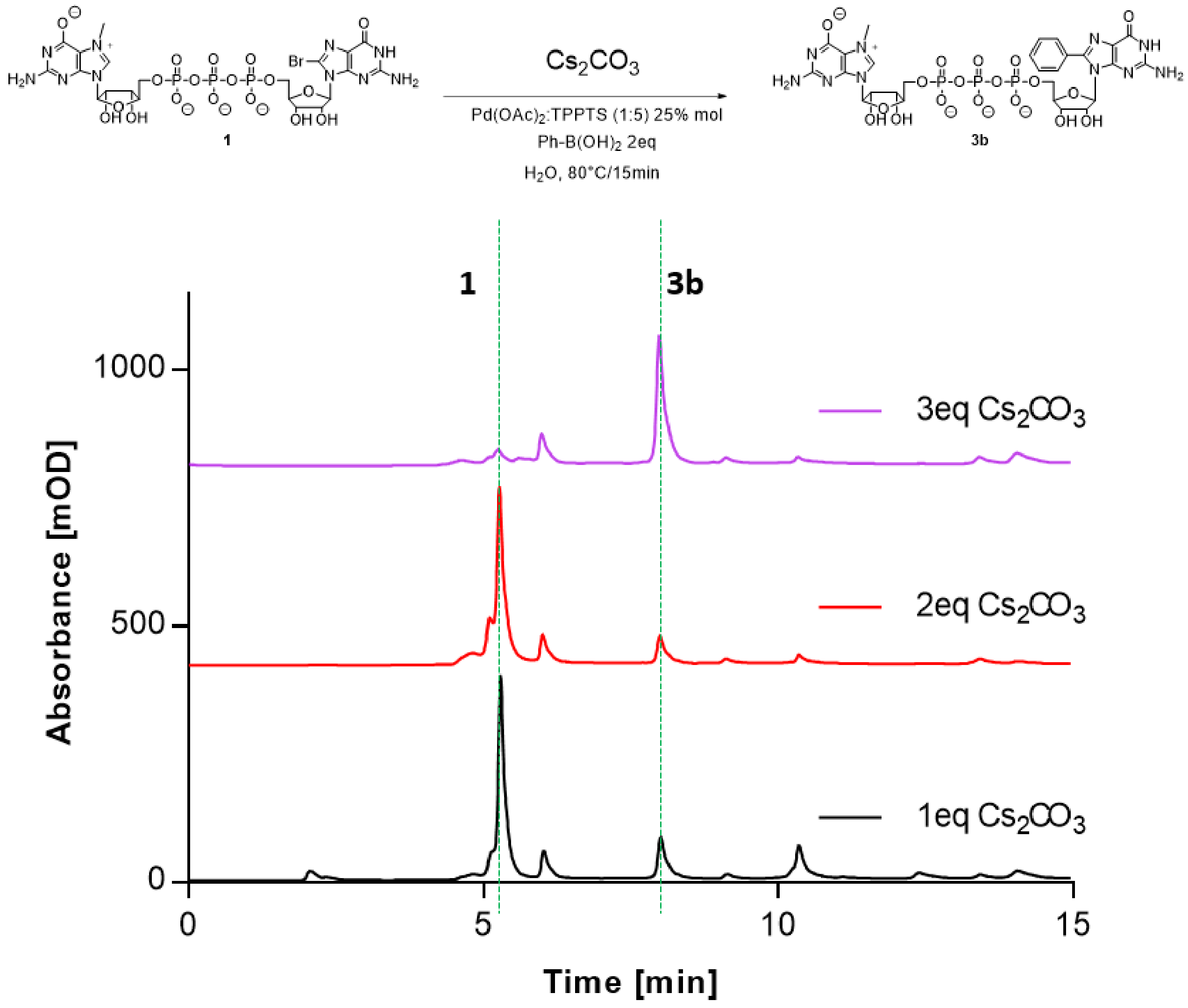

Figure S3. (C18) RP-HPLC reaction profiles of m^7GpppG^{8Ph} (**3b**) synthesis depending on amount of Cs_2CO_3 used in the reaction.

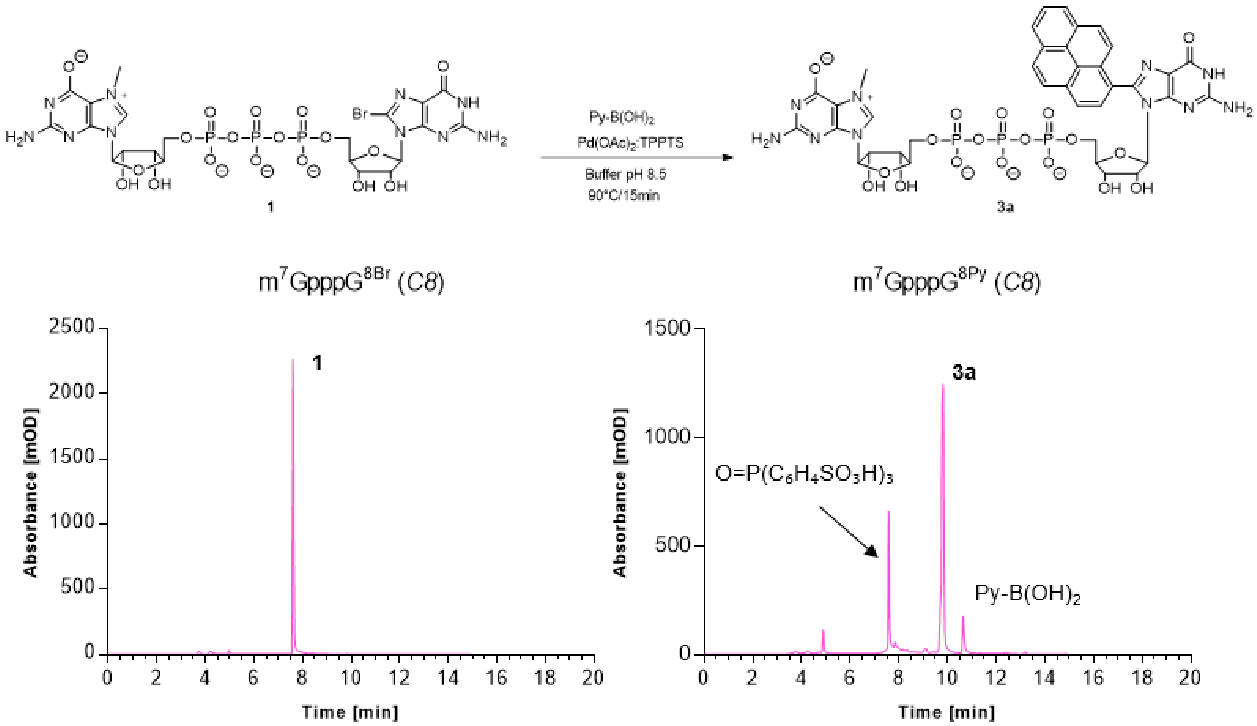

Figure S4. (C8) RP-HPLC reaction profile of post-synthetic modification of cap analog m^{7}GpppG^{8Br} (**1**) via palladium catalyzed Suzuki-Miyaura cross coupling reaction.

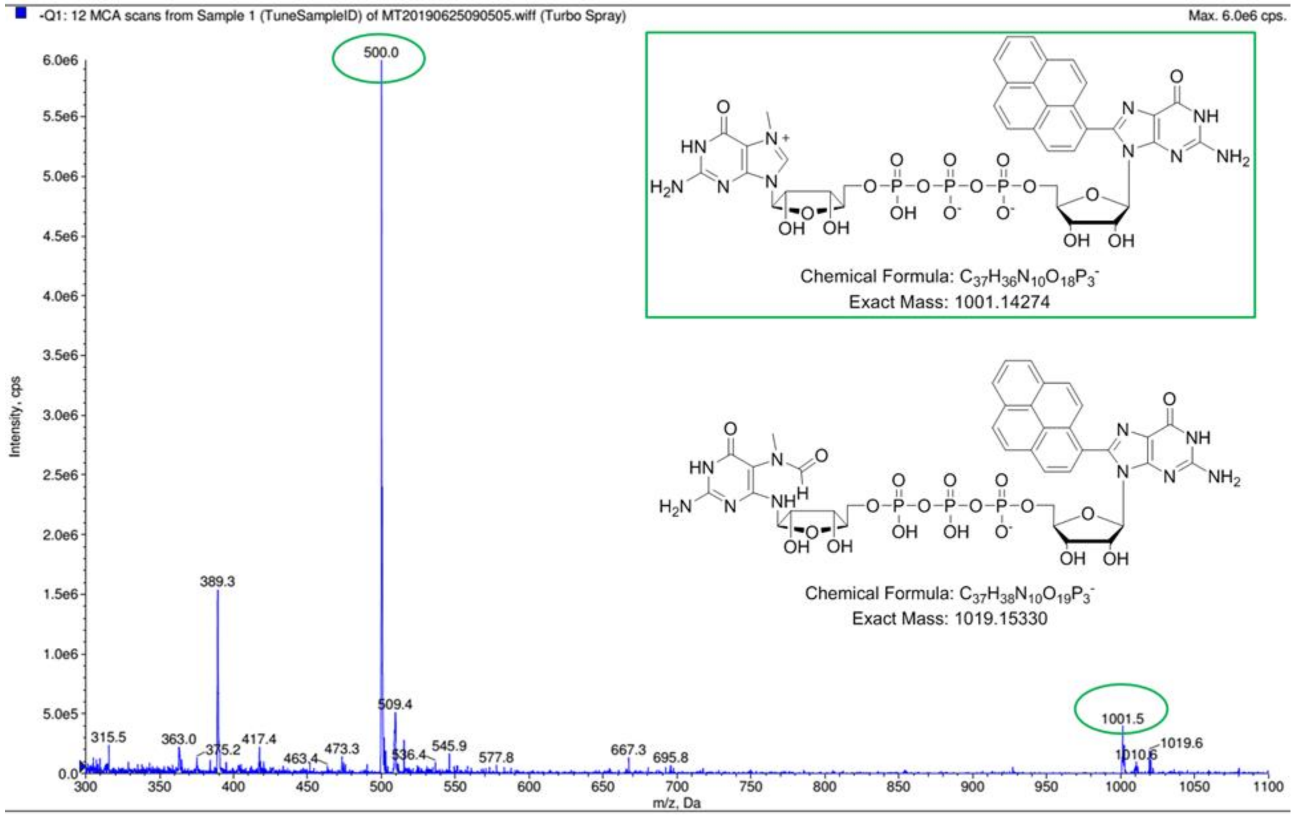

Figure S5. ESI MS of signal (**3a**), t_R = 9.8 min. (mixture of product **3a** and ring opening side product).

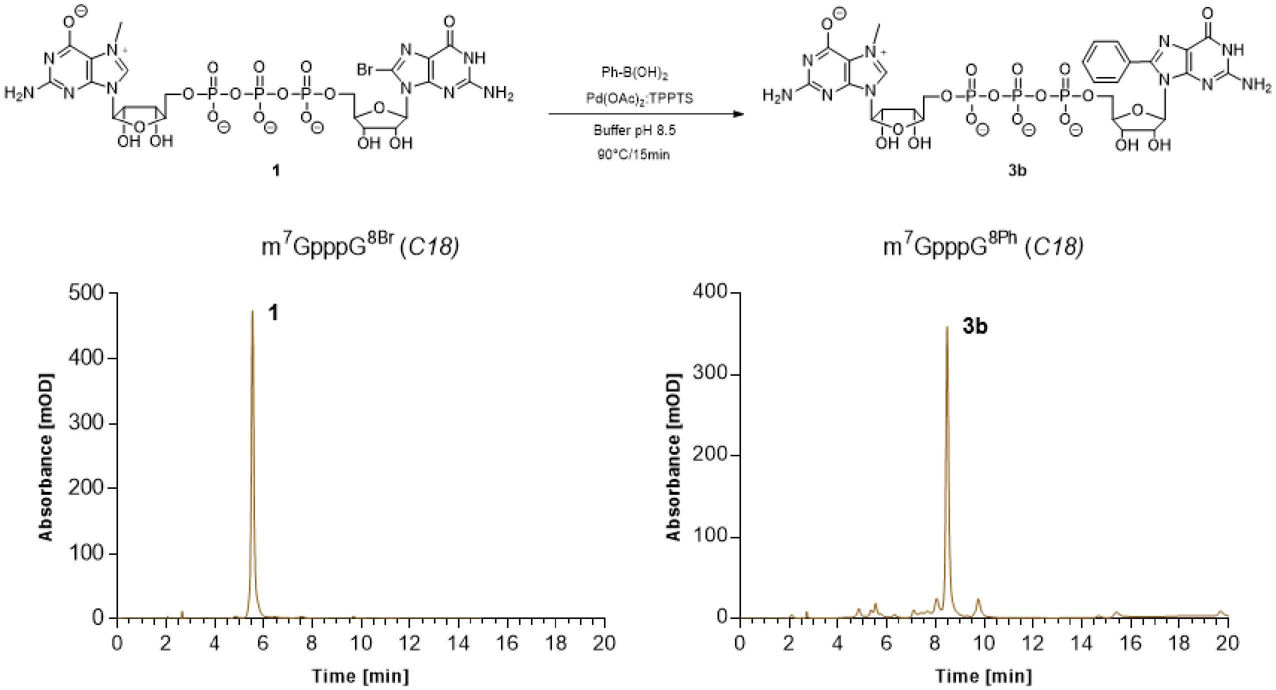

Figure S6. (C18) RP-HPLC reaction profile of post-synthetic modification of cap analog m^{7}GpppG^{8Br} (**1**) via palladium catalyzed Suzuki-Miyaura cross coupling reaction.

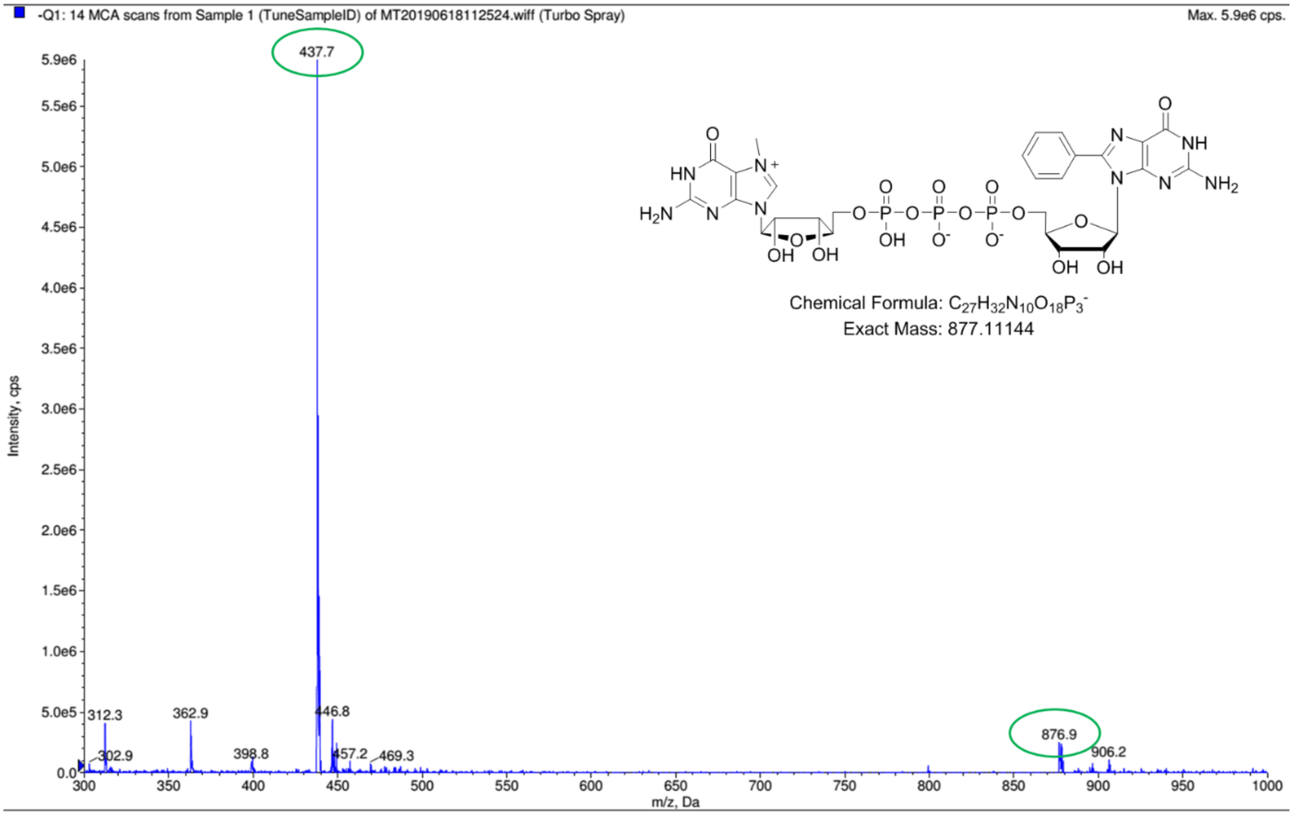

Figure S7. ESI MS of signal (**3b**), t_R = 8.3 min.

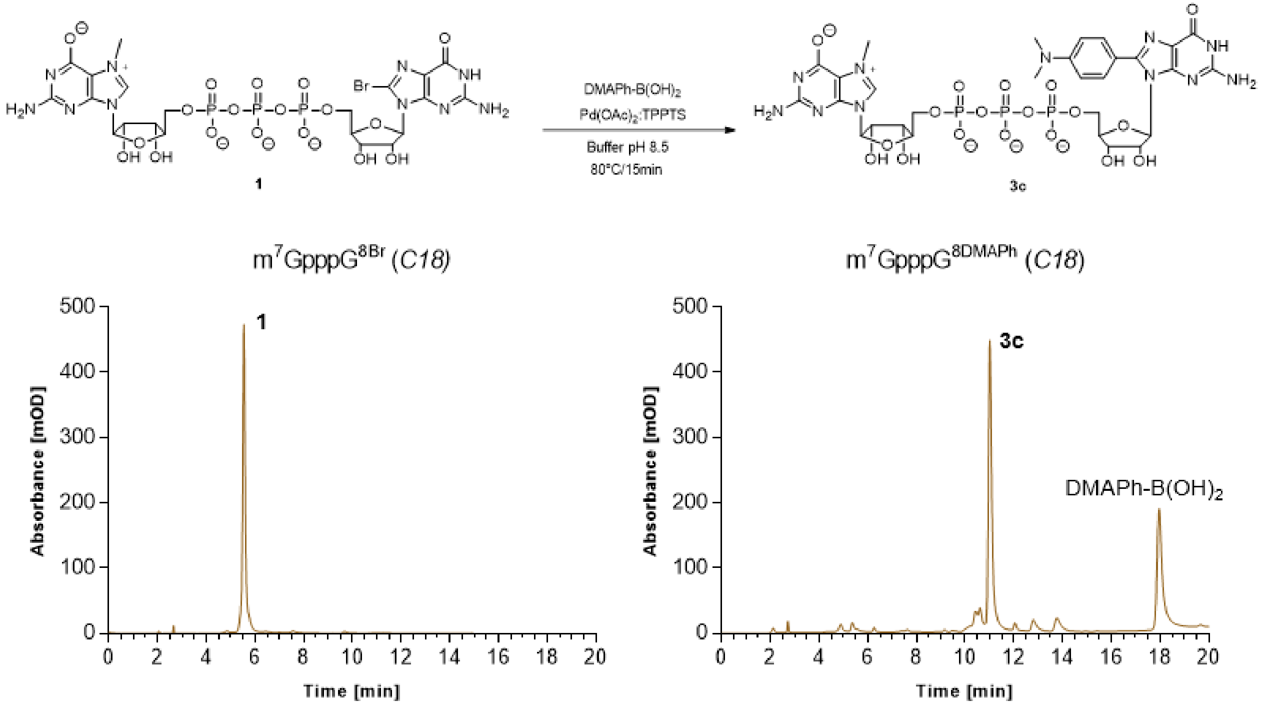

Figure S8. (C18) RP-HPLC reaction profile of post-synthetic modification of cap analog m^{7}GpppG^{8Br} (**1**) via palladium catalyzed Suzuki-Miyaura cross coupling reaction.

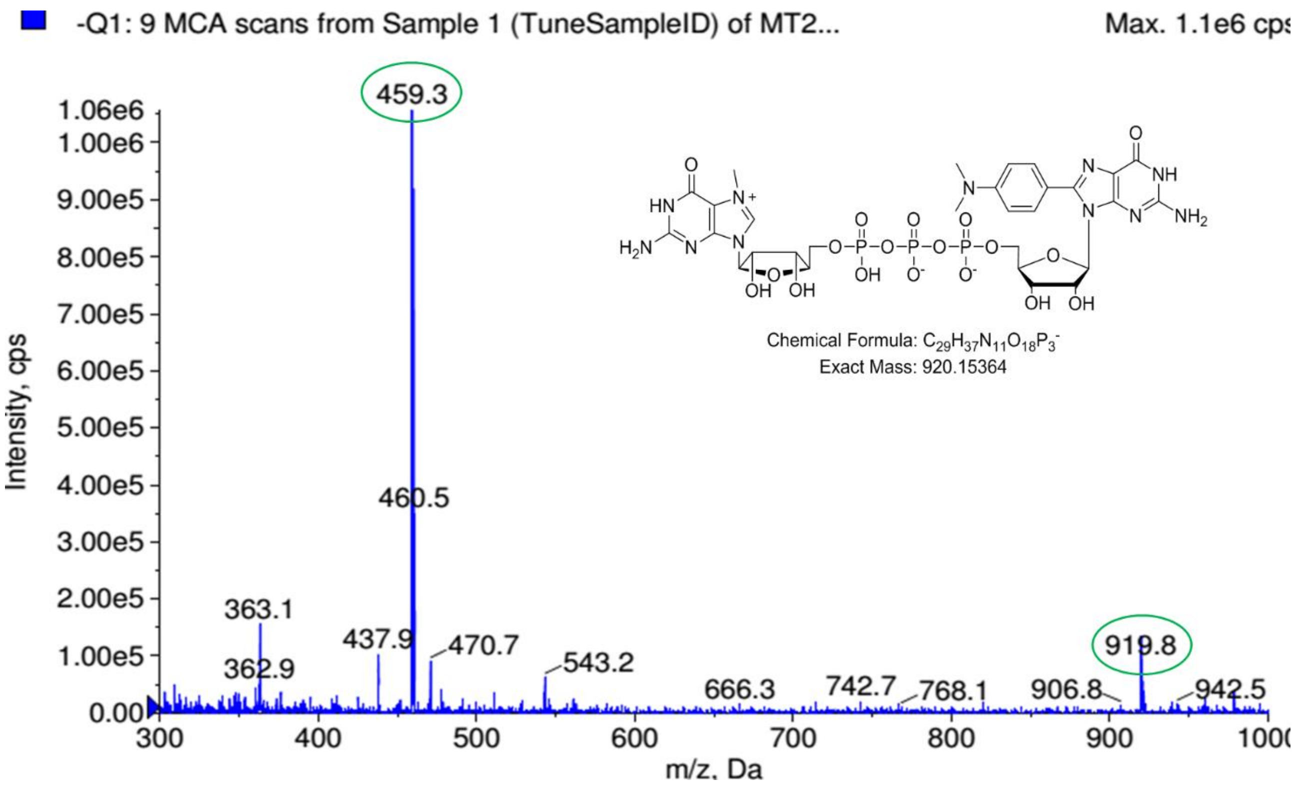

Figure S9. ESI MS of signal (**3c**), t_R = 11.2 min

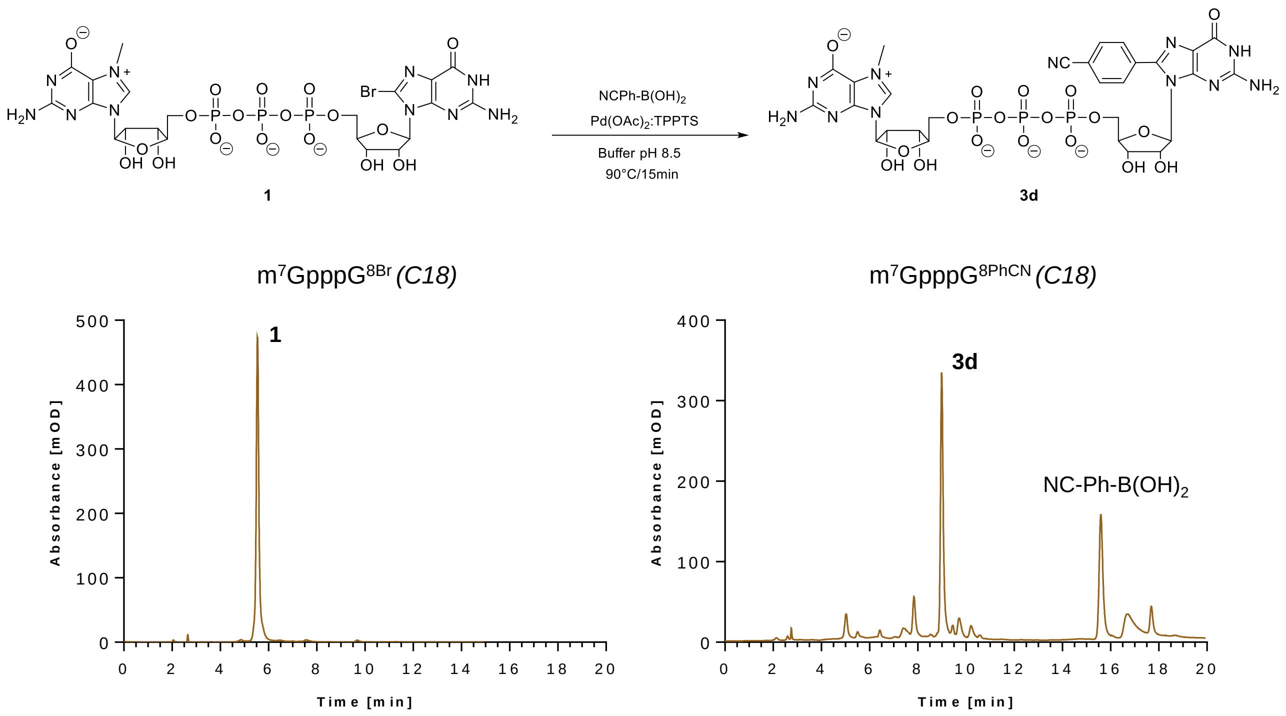

Figure S10. (C18) RP-HPLC reaction profile of post-synthetic modification of cap analog m^{7}GpppG^{8Br} (**1**) via palladium catalyzed Suzuki-Miyaura cross coupling reaction.

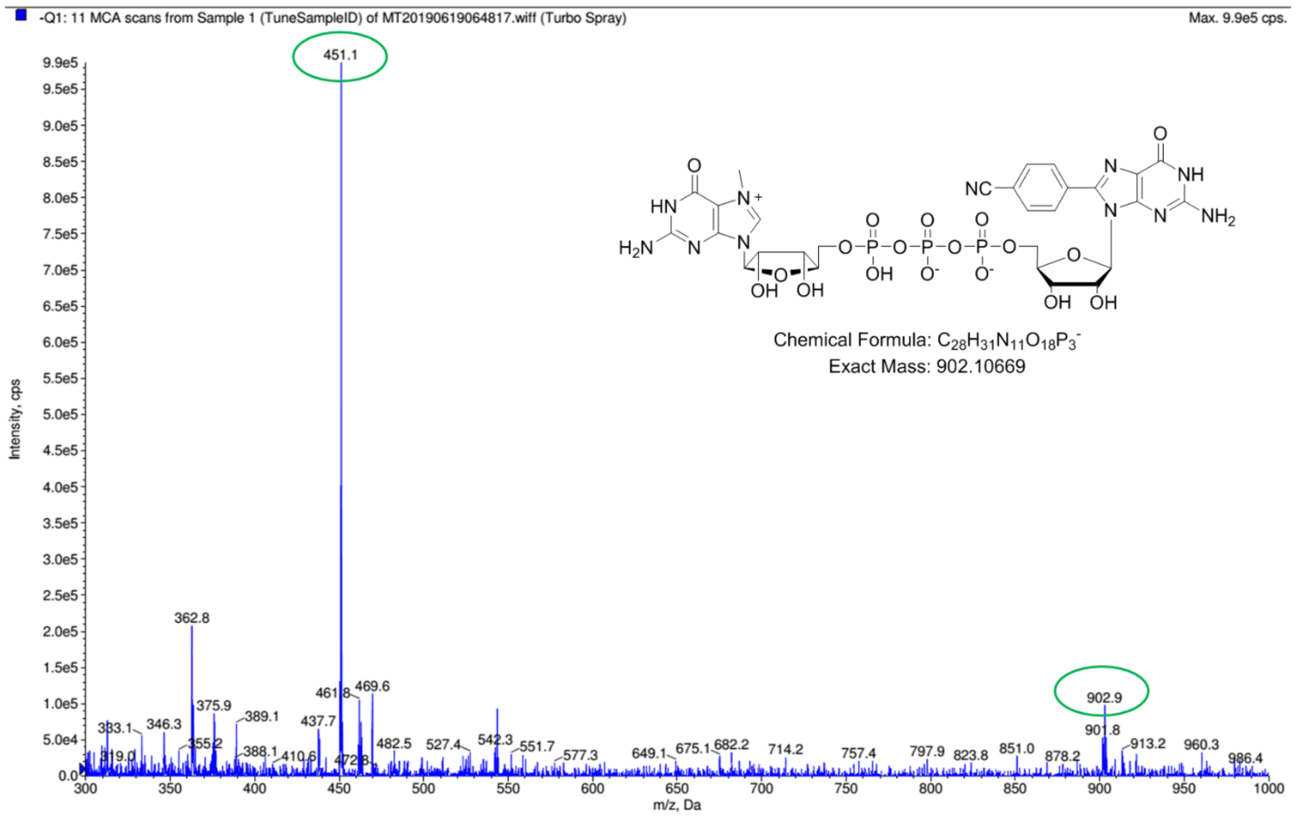

Figure S11. ESI MS of signal (**3d**), t_R = 9.1 min.

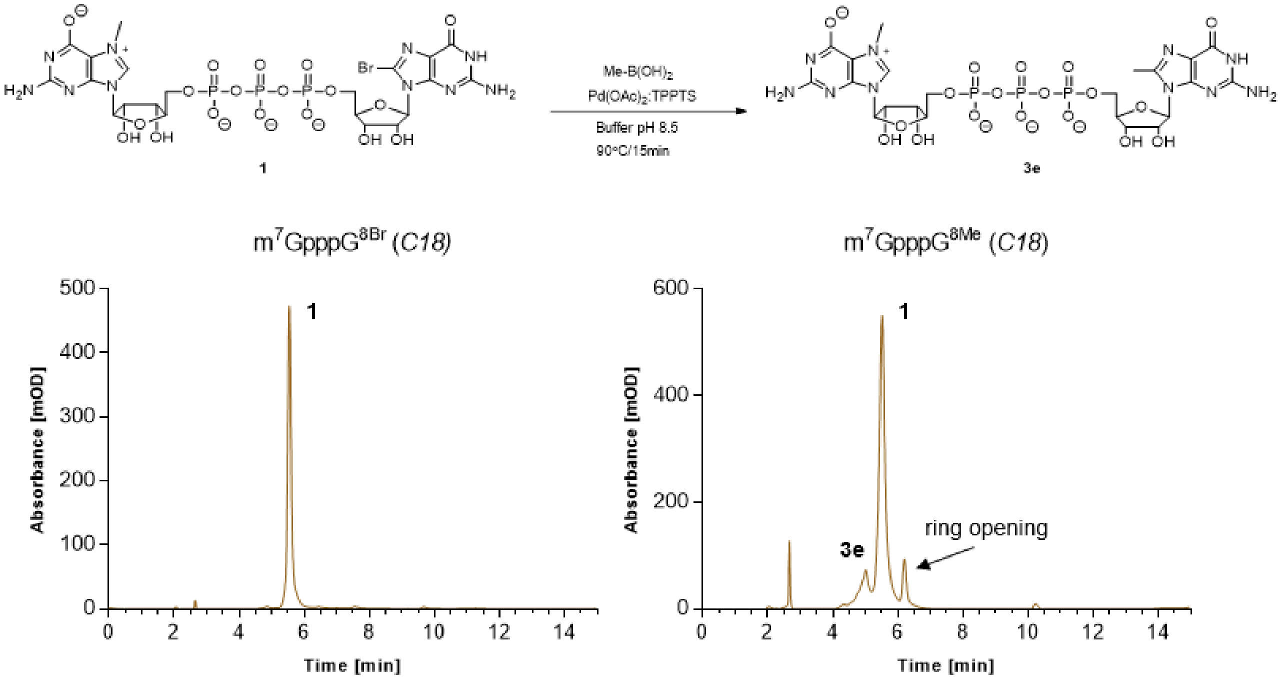

Figure S12. (C18) RP-HPLC reaction profile of post-synthetic modification of cap analog m^{7}GpppG^{8Br} (**1**) via palladium catalyzed Suzuki-Miyaura cross coupling reaction.

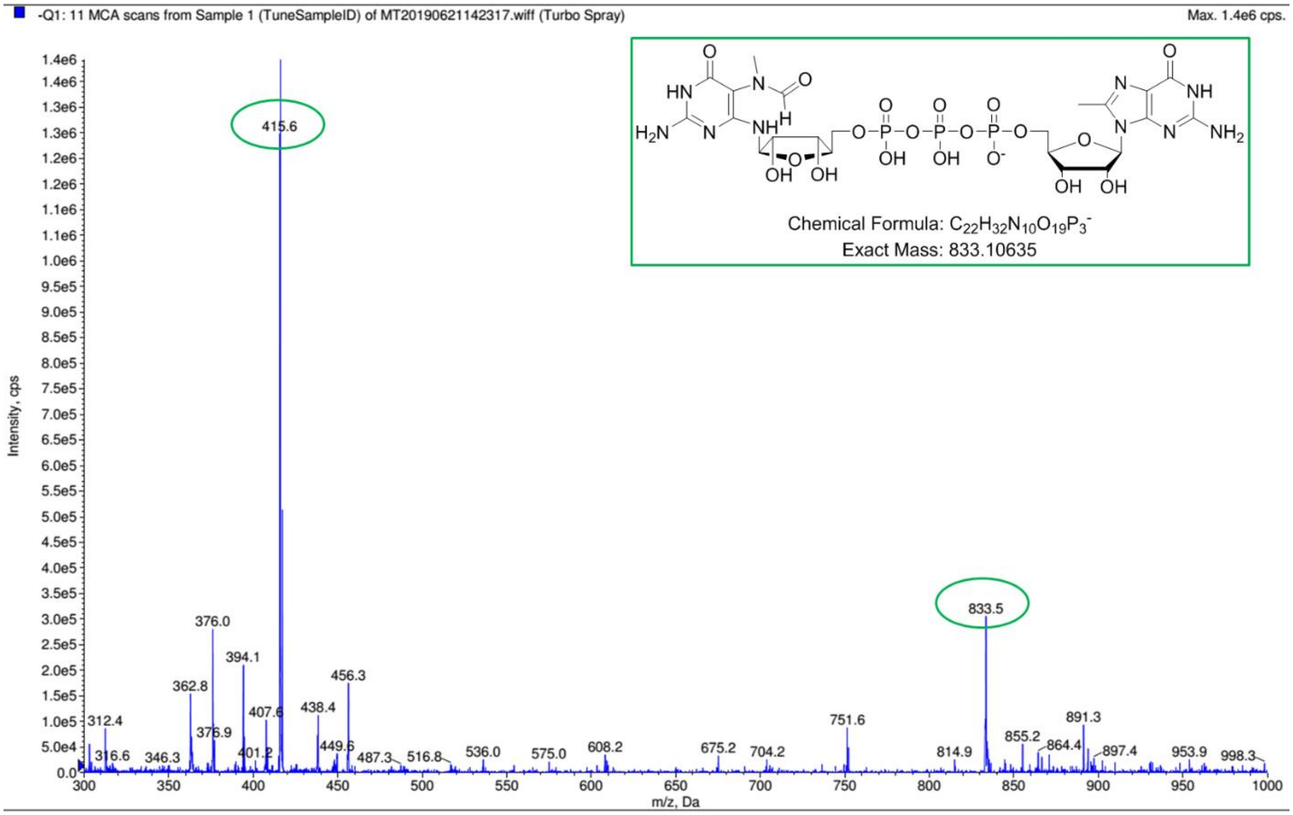

Figure S13. ESI MS of ring opening side product, t_R = 6.1 min.

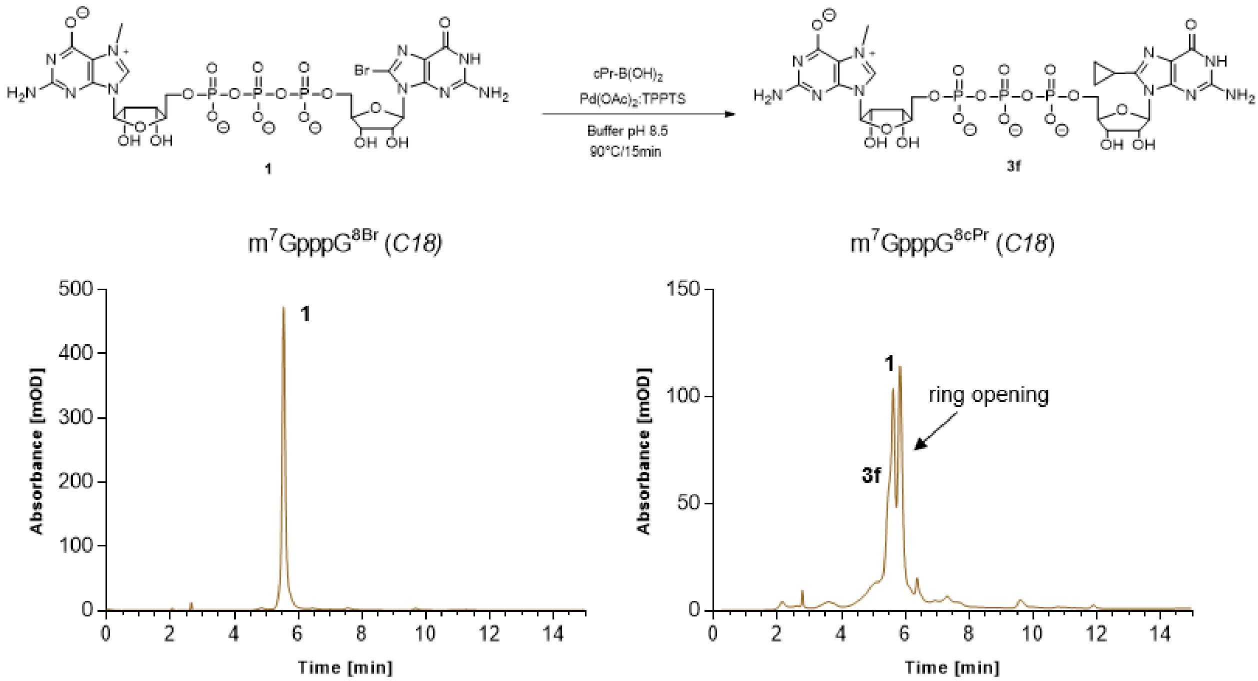

Figure S14. (C18) RP-HPLC reaction profile of post-synthetic modification of cap analog m^{7}GpppG^{8Br} (**1**) via palladium catalyzed Suzuki-Miyaura cross coupling reaction.

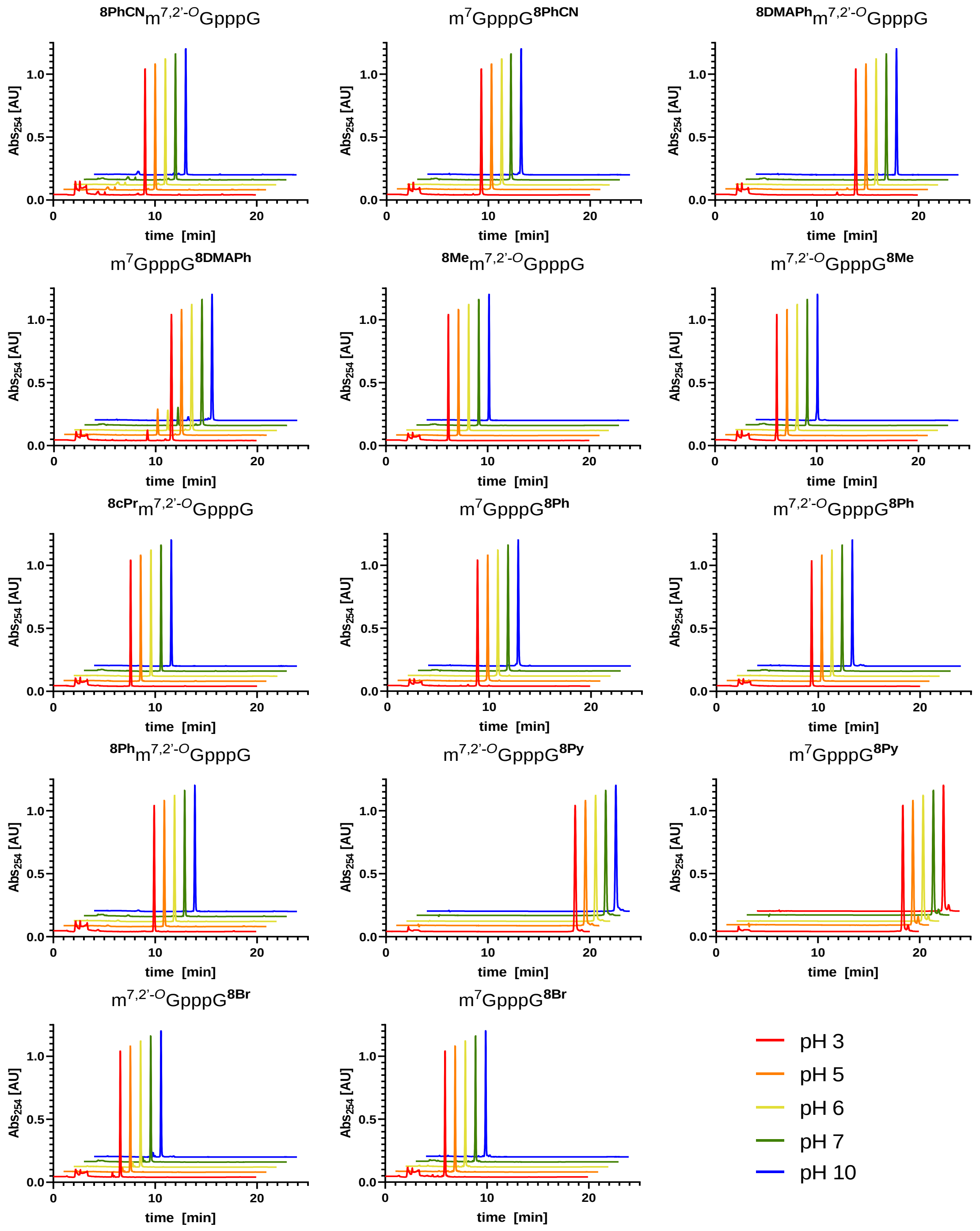

Figure S15. Analysis of chemical stability of 8-modified cap analogs using RP HPLC. Compounds at 50 μM concentration were incubated at various pH (3, 5, 6, 7, and 10) for 5h at 37 °C min followed by RP HPLC analysis as described in the experimental section. Buffers: pH 3: 100 mM sodium citrate buffer; pH 5: 100 mM ammonium acetate buffer; pH 6: 100 mM potassium phosphate buffer, pH 7: 100 mM potassium phosphate buffer, pH 10: 100 mM ammonium chloride buffer.

2. Photophysical properties (Figure S16-S17)

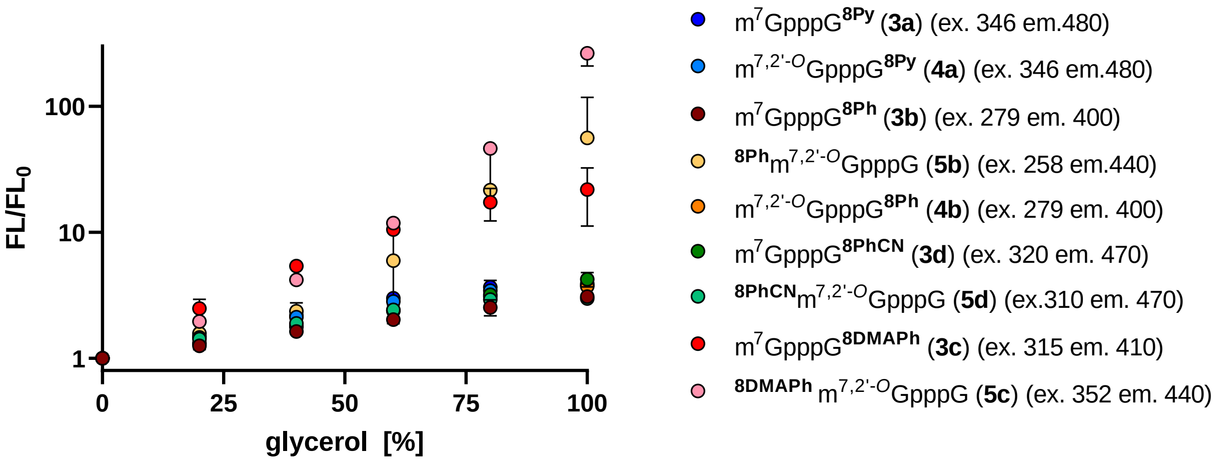

Figure S16. Fluorescence intensity relationship of C8-modified cap analogs at increasing glycerol concentration.

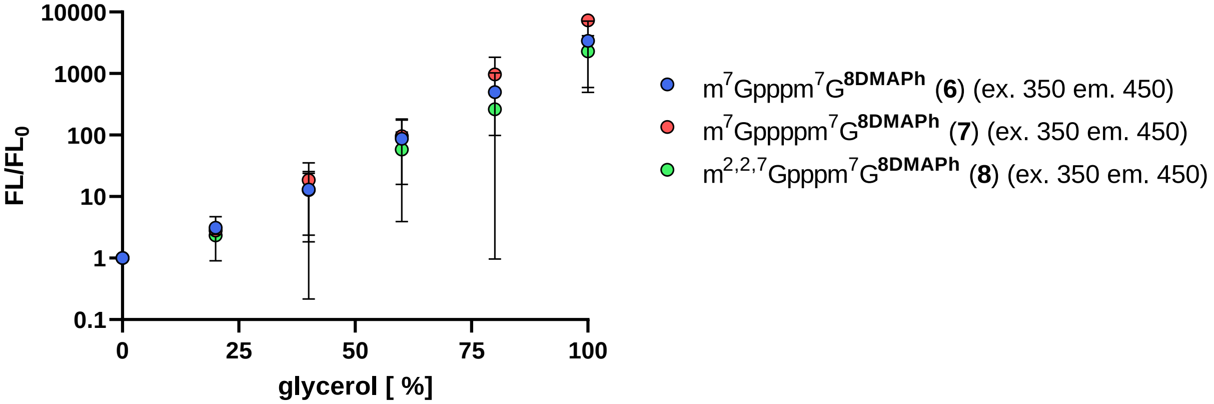

Figure S17. Fluorescence intensity relationship of two-headed C8-modified cap analogs at increasing glycerol concentration.

3. Biophysical studies (Figure S18-S20)

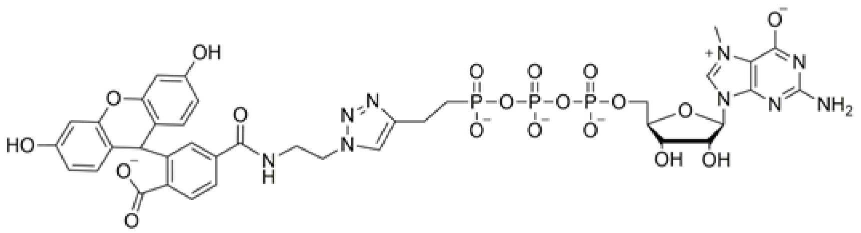

Figure S18. Structure of the probe m^{7}Gppp-triazol-(6)FAM used in fluorescence anisotropy experiments to study interactions with eIF4E protein.

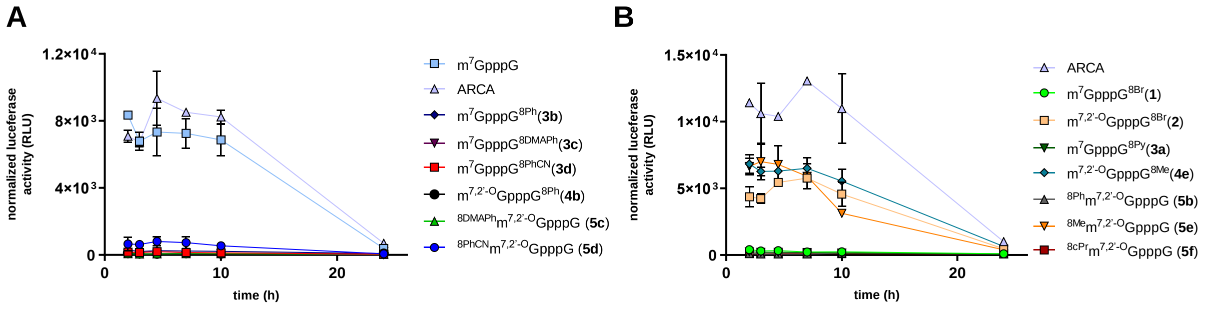

Figure S19. Translation efficiency of mRNAs carrying various C8 modified cap analogs at the 5’ end. A-B. Firefly and Renilla (for ARCA) luciferase activity in the supernatant of HeLa cells measured after 2, 3, 4.5, 7, 10 and 24 hours after transfection.

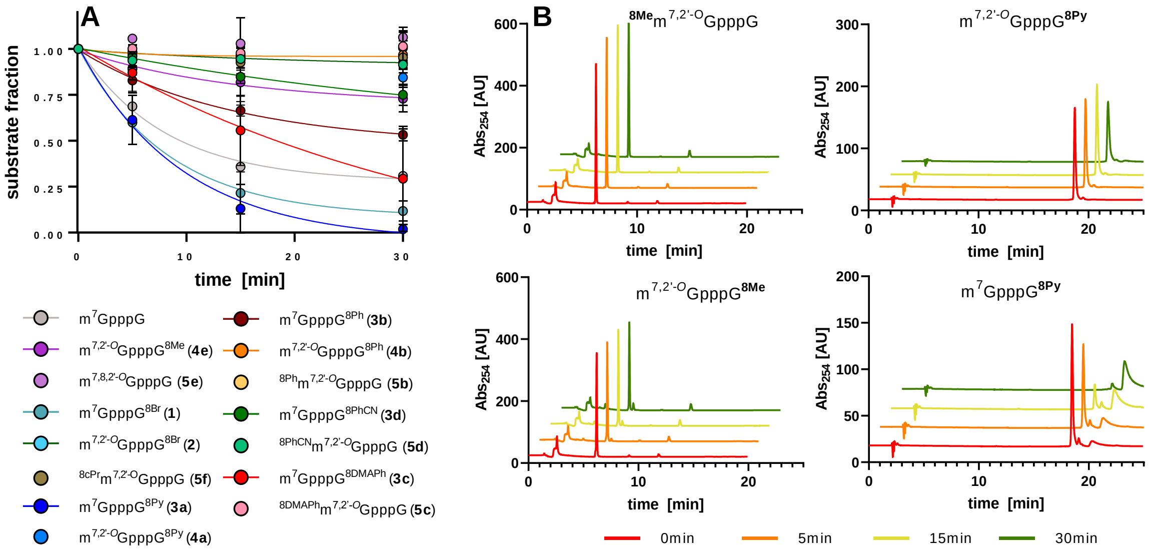

Figure S20. A) Susceptibility to DcpS hydrolysis; B) Representative RP HPLC profiles after 0, 5, 15, and 30 min of incubation are shown for: ^{8Me}m$^{7,2'O}$GpppG, m$^{7,2'O}$GpppG^{8Py}, m$^{7,2'O}$GpppG^{8Me}, m^{7}GpppG^{8Py}.

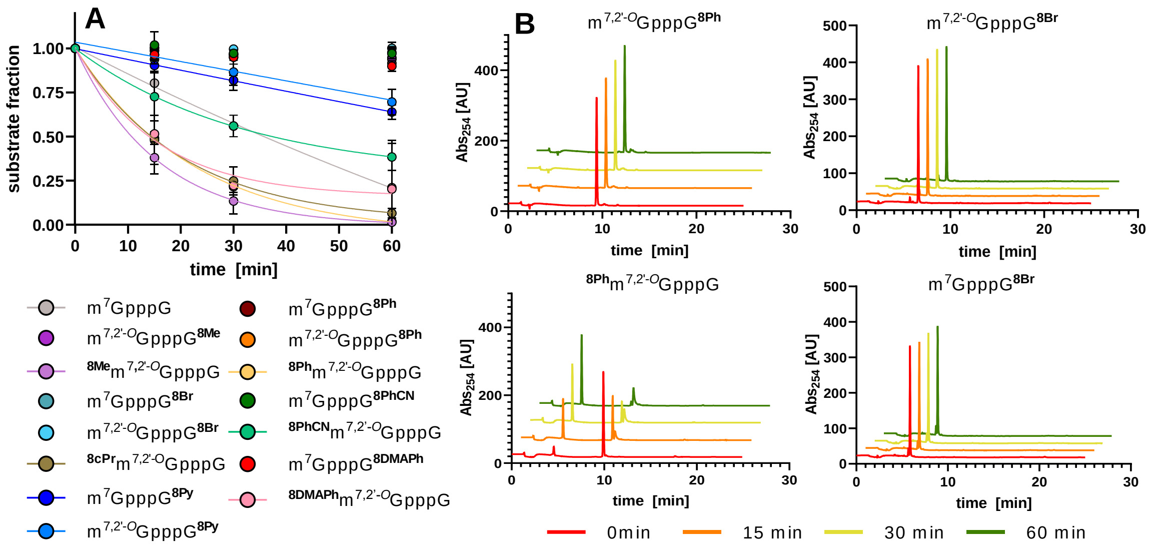

Figure S21. A) Susceptibility to Nudt16 hydrolysis; B) Representative RP HPLC profiles after 0, 15, 30 and 60 min of incubation are shown for: $^{m7,2'O}$GpppG^{8Ph}, $^{m7,2'O}$GpppG^{8Br}, ^{8Ph}m$^{7,2'O}$GpppG, m^{7}GpppG^{8Br}.

4. Tables

Table S1. Photophysical data of 8-substituted monophosphates (**14b-14d** and **15b-15d**) in different solvents.

	**14b**		**15b**	
**Solvent**	**$^{8Ph}m^{2'O}GMP$ λ_{abs} [nm]**	**$^{8Ph}m^{2'O}GMP$ (λ_{ex} 279 nm) λ_{em} [nm]**	**$^{8Ph}m^{7,2'O}GMP$ λ_{abs} [nm]**	**$^{8Ph}m^{7,2'O}GMP$ (λ_{ex} 269 nm) λ_{em} [nm]**
Buffer pH 7	279	400	225/269/297*	420*
MeOH	283	385	271/299	442
EtOH	286	383	272/300	441
iPrOH	284	383	273/302	433
CH_3CN	285	390	245/271/299	434
50 % Glycerol	281	394	270/297	430

	**14c**		**15c**	
**Solvent**	**$^{8DMAPh}m^{2'O}GMP$ λ_{abs} [nm]**	**$^{8DMAPh}m^{2'O}GMP$ (λ_{ex} 291 nm) λ_{em} [nm]**	**$^{8DMAPh}m^{7,2'O}GMP$ λ_{abs} [nm]**	**$^{8DMAPh}m^{7,2'O}GMP$ (λ_{ex} 343 nm) λ_{em} [nm]**
pH 7	291	416	274/343*	440*
MeOH	312	400	275/343	437
EtOH	309	399	275/342	443
iPrOH	310	391	276/342	441
CH_3CN	311	395	278/342	453
50 % Glycerol	295/315	414	276/351	440

Table S1. Continued. Photophysical data of 8-substituted monophosphates (**14b-14d** and **15b-15d**) in different solvents.

	**14d**		**15d**	
**Solvent**	**$^{8CNPh}m^{2'O}GMP$ λ_{abs} [nm]**	**$^{8CNPh}m^{2'O}GMP$ (λ_{ex} 284 nm) λ_{em} [nm]**	**$^{8CNPh}m^{7,2'O}GMP$ λ_{abs} [nm]**	**$^{8CNPh}m^{7,2'O}GMP$ (λ_{ex} 271 nm) λ_{em} [nm]**
Buffer pH 7	233/284/316	473	234/271/305*	467*
MeOH	234/285/326	452	234/271/314	473
EtOH	236/286/330	451	235/272/322	478
iPrOH	236/286/336	449	235/272/326	488
CH_3CN	236/286/332	453	271/326	487
50 % Glycerol	235/285/320	460	235/273/310	466

(*) Spectra were recorded in buffer pH 6.0

Table S2. Photophysical data of 8-substituted monophosphates (**14b-d, 15b-d**) and cap analogs (**3a-d, 4a, 4b, 4d, 5b, 5c, 6-8**)

No	Compound	$\lambda_{abs,max}$ [nm]	λ_{ex} [nm]	λ_{em} [nm]
**3a**	$m^{7}GpppG^{8Py}$	240	346	484
**3b**	$m^{7}GpppG^{8Ph}$	279	279	398
**3c**	$m^{7}GpppG^{8DMAPh}$	288	288	414
**3d**	$m^{7}GpppG^{8CNPh}$	283, 319	283, 319	466
**4a**	$m^{7,2'-O}GpppG^{8Py}$	240	346	484
**4b**	$m^{7,2'-O}GpppG^{8Ph}$	280	280	398
**4d**	$^{8PhCN}m^{7,2'-O}GpppG$	245, 310	245, 310	472
**5b**	$^{8Ph}m^{7,2'-O}GpppG$	258	258	432
**5c**	$^{8DMAPh}m^{7,2'-O}GpppG$	280, 352	352	444
**6**	$^{8DMAPh}m^{7}Gpppm^{7}G$	x	x	x
**7**	$^{8DMAPh}m^{7}Gppppm^{7}G$	x	x	x
**8**	$m^{2,2,7}Gpppm^{7}G^{8DMAPh}$	x	x	x
**14b**	$^{8Ph}m^{2'-O}GMP$	279	279	400
**14c**	$^{8DMAPh}m^{2'-O}GMP$	291	291	416
**14d**	$^{8PhCN}m^{2'-O}GMP$	233, 284, 316	284	473
**15b**	$^{8Ph}m^{7,2'-O}GMP$*	225, 269, 297	269	420
**15c**	$^{8DMAPh}m^{7,2'-O}GMP$*	274, 343	270, 340	410
**15d**	$^{8PhCN}m^{7,2'-O}GMP$*	234, 271, 305	271	467

(*) Spectra were recorded in buffer pH 6.0

5. **Compounds characterization (HPLC profiles, NMR and HRMS spectra)**

[8-Br]GMP, 8-Bromoguanosine 5'-monophosphate (11)

Structure

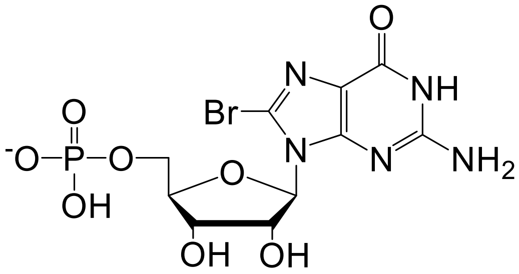

RP-HPLC profile

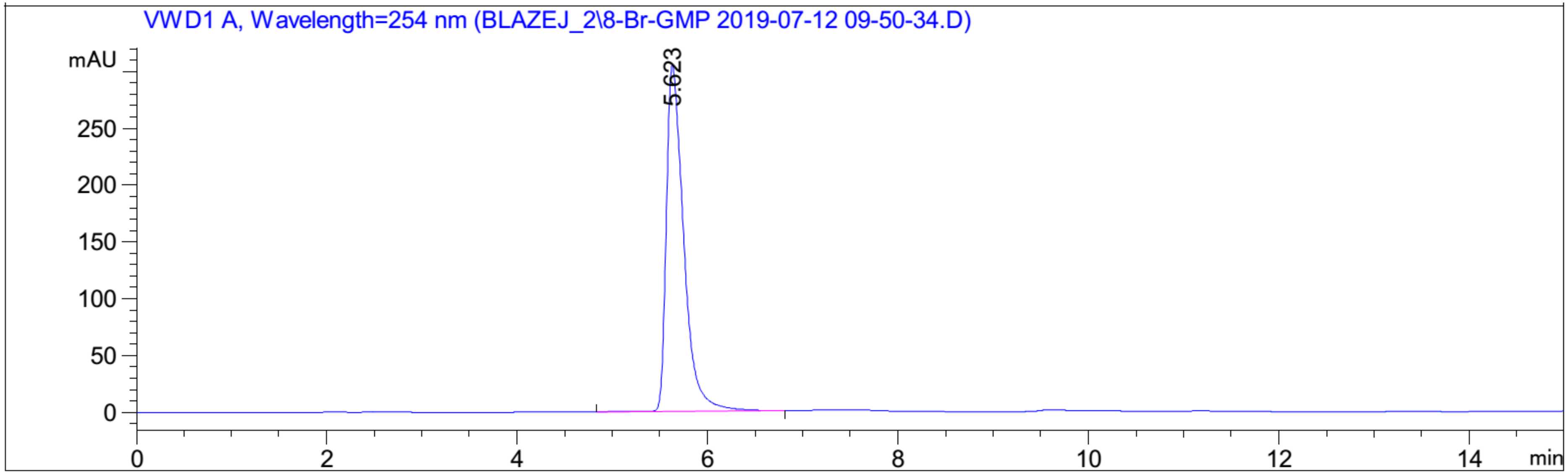

^{1}H NMR

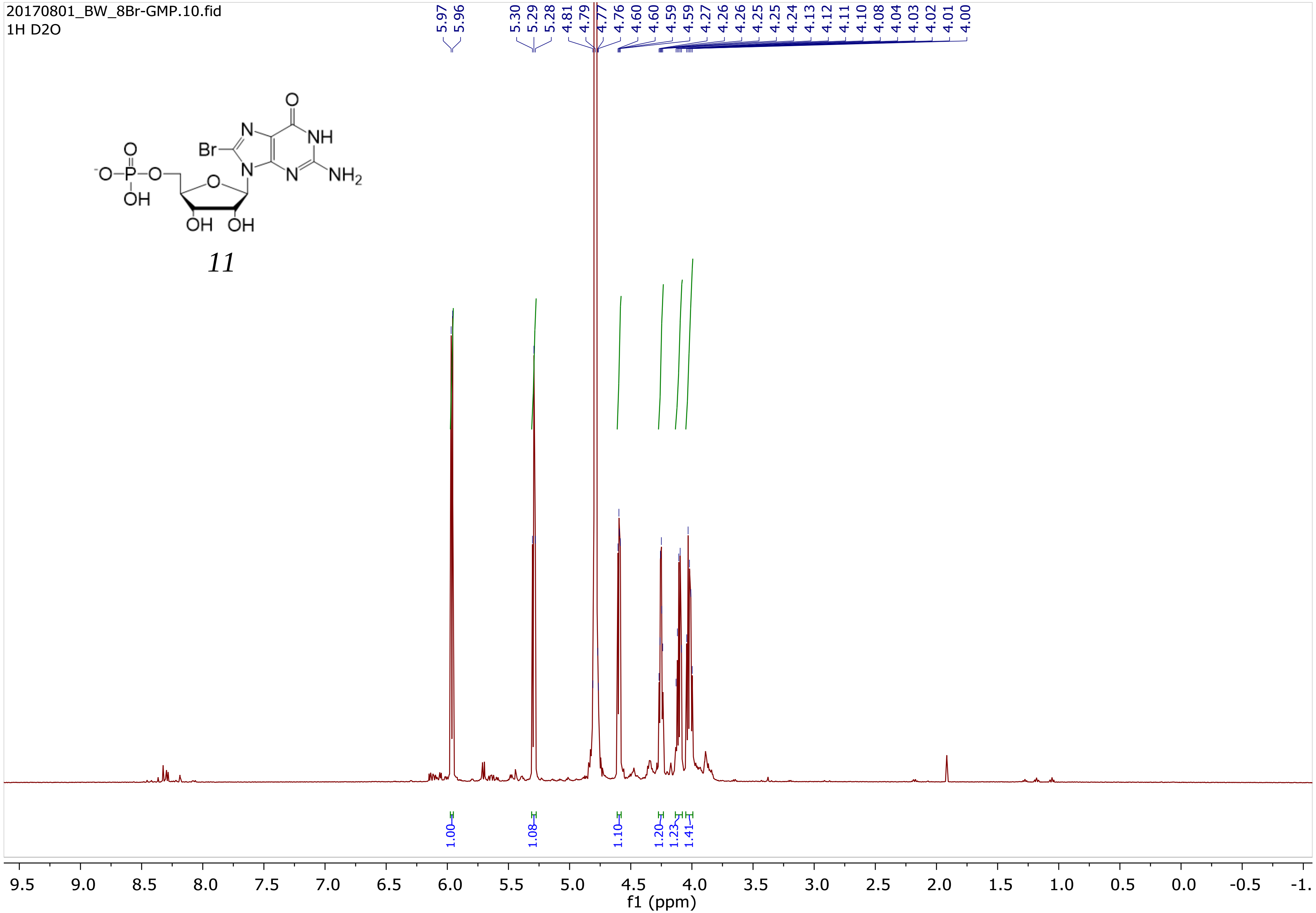

20170801_BW_8Br-GMP.10.fid
1H D2O
5.97
5.96
5.30
5.29
5.28
4.81
4.79
4.77
4.76
4.60
4.60
4.59
4.59
4.27
4.26
4.26
4.25
4.25
4.24
4.13
4.12
4.11
4.10
4.08
4.04
4.03
4.02
4.01
4.00
11
1.00
1.08
1.10
1.20
1.23
1.41
9.5 9.0 8.5 8.0 7.5 7.0 6.5 6.0 5.5 5.0 4.5 4.0 3.5 3.0 2.5 2.0 1.5 1.0 0.5 0.0 -0.5 -1.
f1 (ppm)

^{1}H-^{1}H COSY NMR

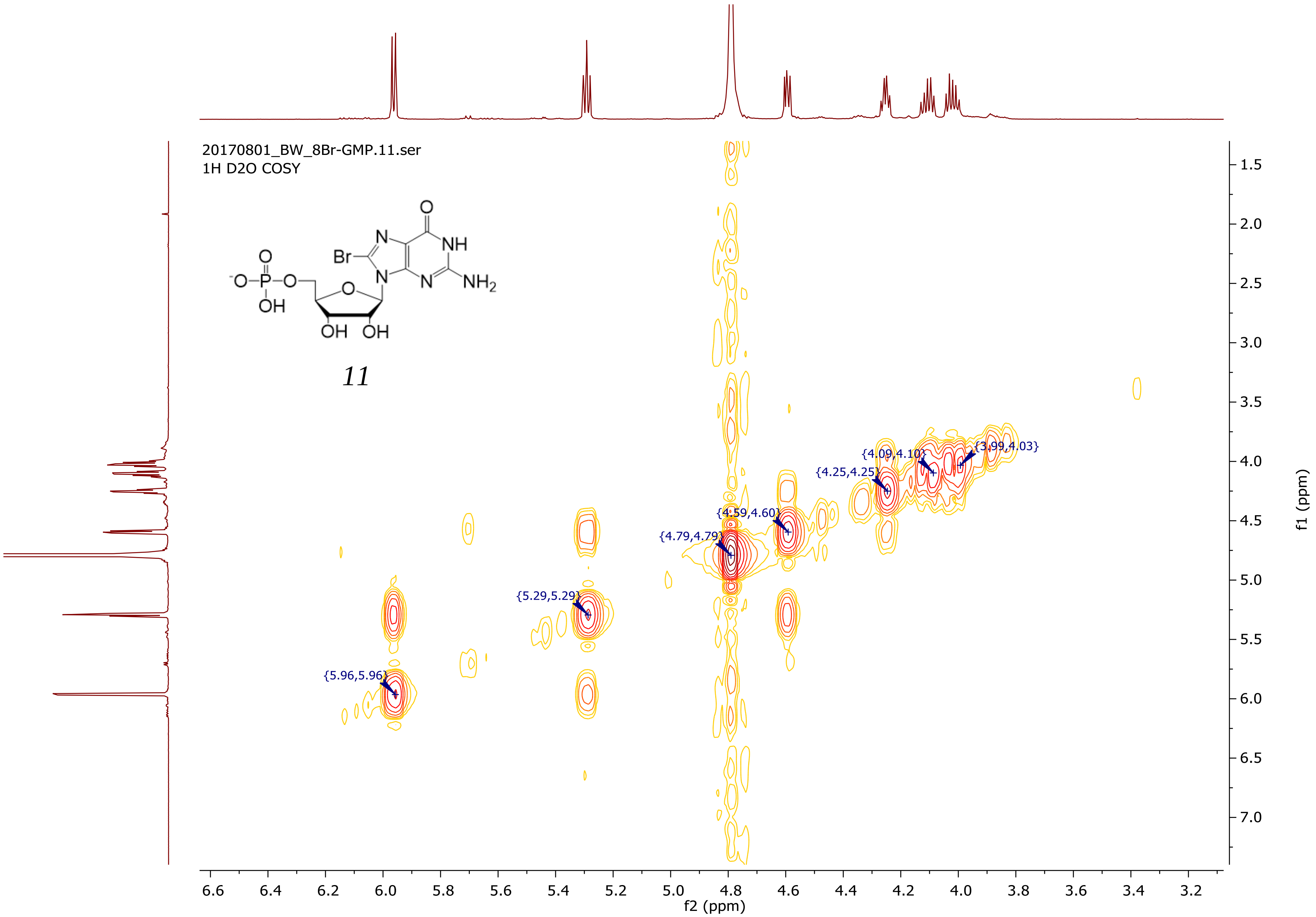

^{31}P NMR

20170801_BW_8Br-GMP.12.fid
31P D2O

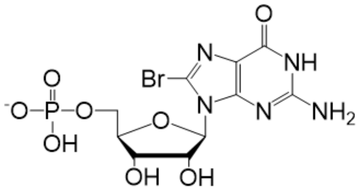

11

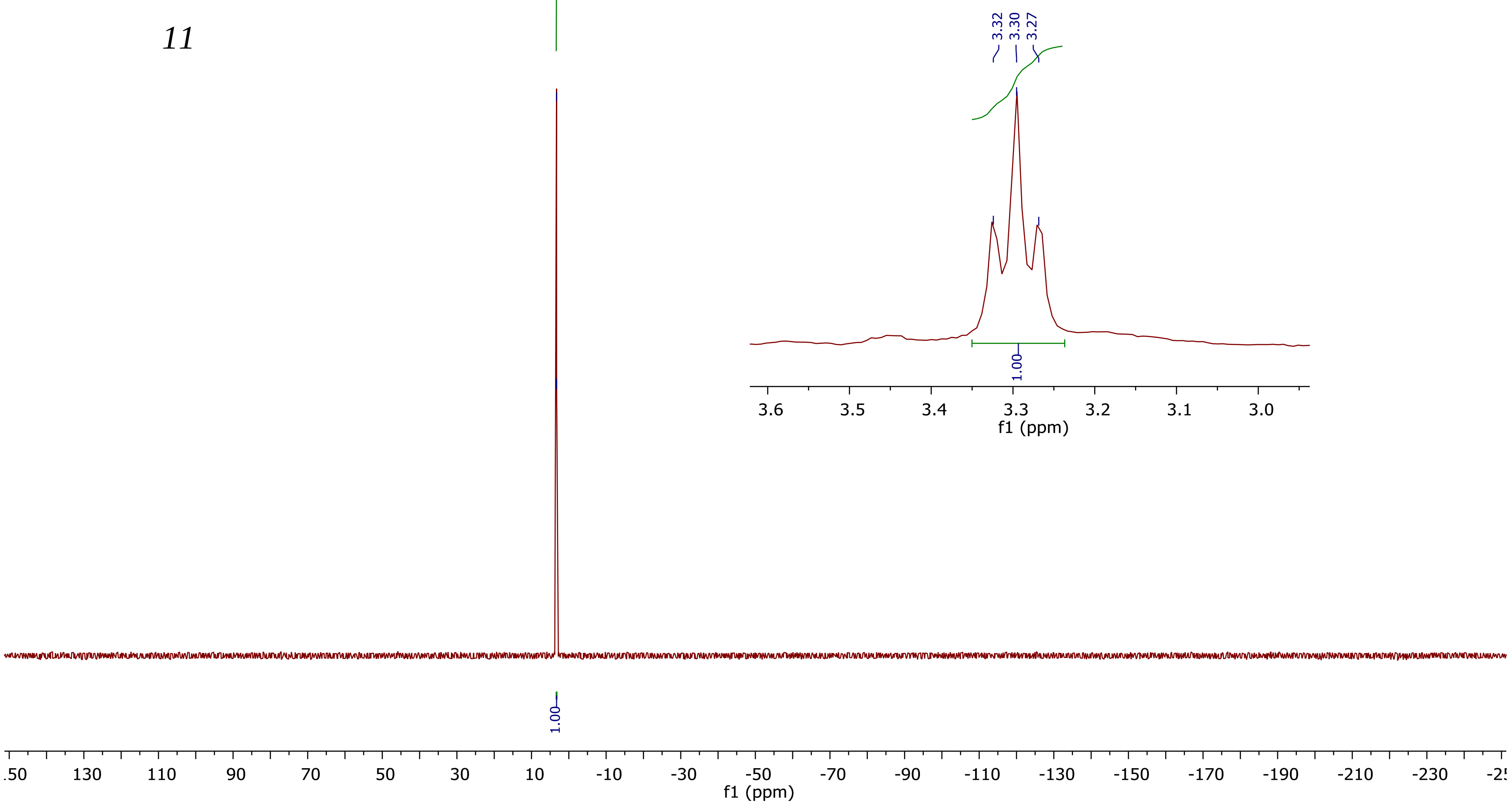

HRMS

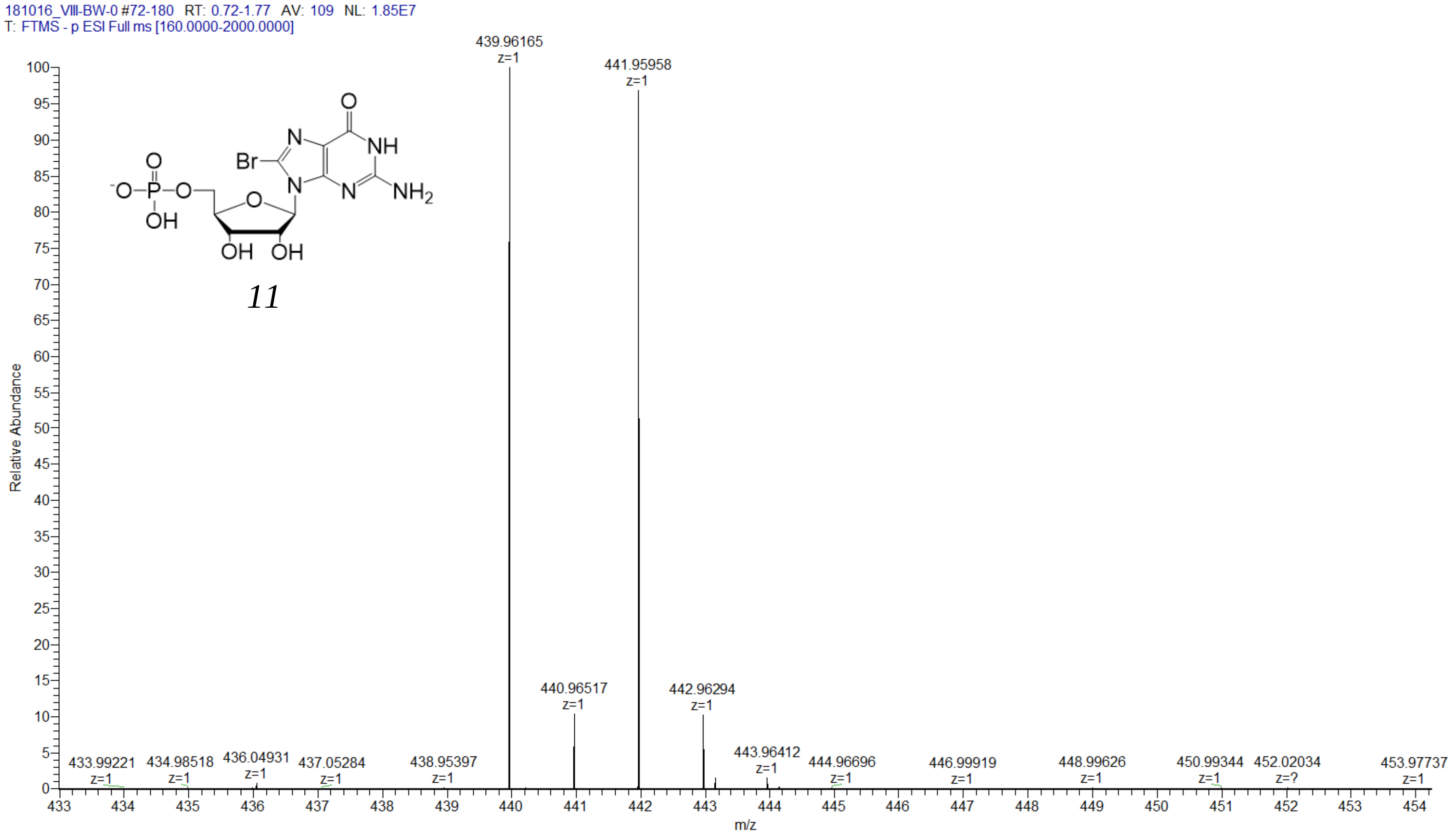

Summary

Rt (A) = 5.6 min; ^{1}H NMR (500 MHz, D_2O) δ 5.96 (d, *J* = 6.1 Hz, 1H), 5.29 (t, *J* = 5.9 Hz, 1H,), 4.59 (dd, *J* = 5.8 Hz, 3.9 Hz, 1H), 4.25 (td, *J* = 5.5 Hz, 3.9 Hz, 1H), 4.11 (dt, *J* = 11.2 Hz, 5.6 Hz, 1H), 4.02 (dt, *J* = 11.4 Hz, 5.8 Hz, 1H); ^{31}P NMR (202 MHz, D_2O) δ 3.3 (t, *J* = 5.6 Hz, 1P); HRMS ESI (-) m/z [M-H]$^-$, calcd for $C_{10}H_{12}BrN_5O_8P^-$ 439.9612, 441.9592; found 439.9617, 441.9596.

$^{8\text{-}Br}m^{2'O}$GMP, 8-Bromo-2'-O-methylguanosine-5'-monophosphate (12)

Structure

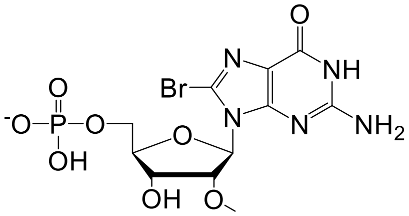

RP-HPLC profile

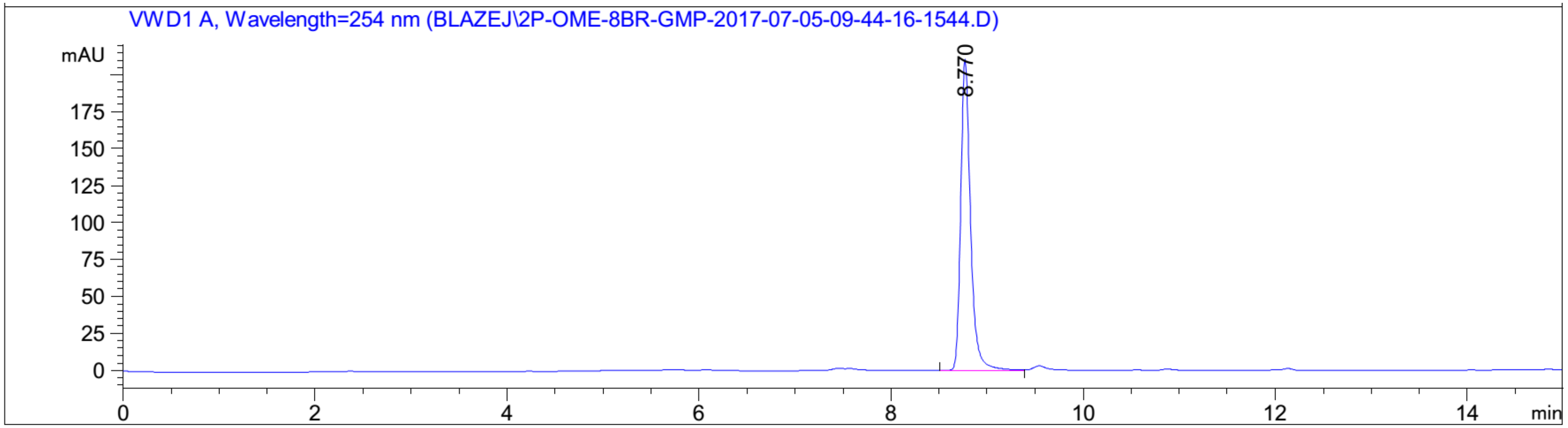

^{1}H NMR

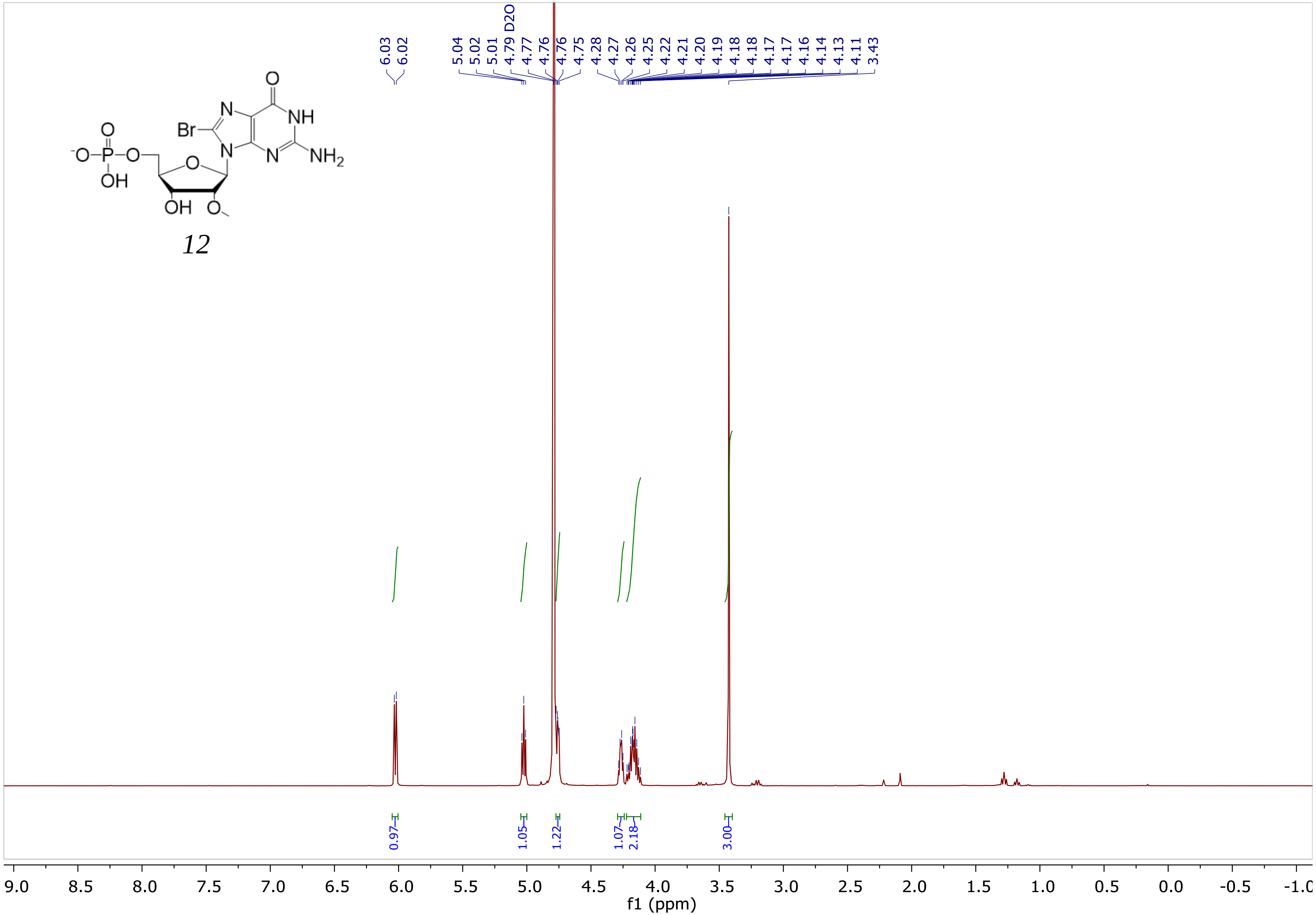

6.03
6.02
5.04
5.02
5.01
4.79 D2O
4.77
4.76
4.76
4.75
4.28
4.27
4.26
4.25
4.22
4.21
4.20
4.19
4.18
4.18
4.17
4.17
4.16
4.14
4.13
4.11
3.43
Br
N
NH
NH2
O
OH
0.97
1.05
1.22
1.07
2.18
3.00
9.0
8.5
8.0
7.5
7.0
6.5
6.0
5.5
5.0
4.5
4.0
3.5
3.0
2.5
2.0
1.5
1.0
0.5
0.0
-0.5
-1.0
f1 (ppm)

12

^{1}H-^{1}H COSY NMR

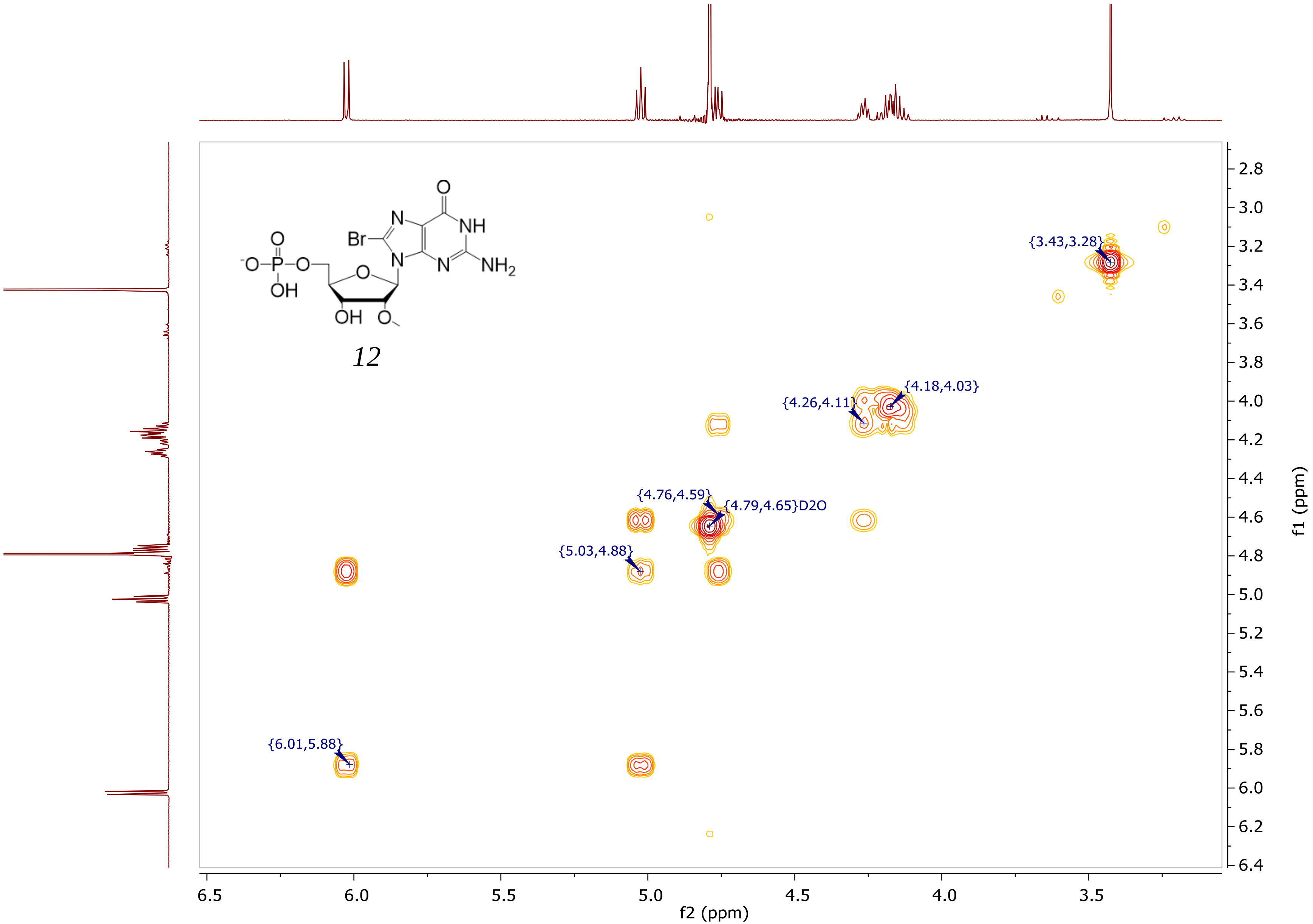

^{31}P NMR

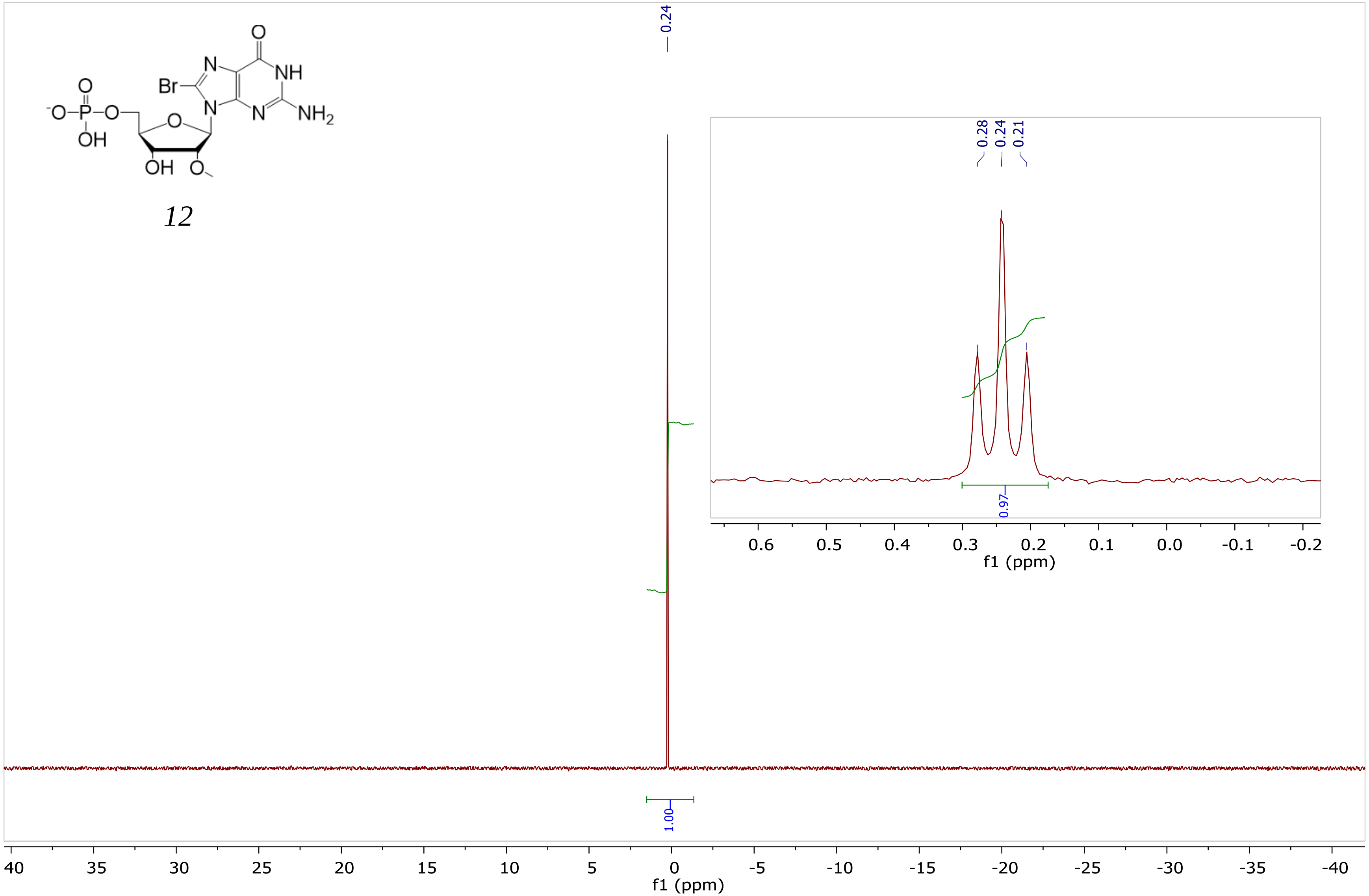

12
0.24
1.00
0.28
0.24
0.21
0.97
40
35
30
25
20
15
10
5
0
-5
-10
-15
-20
-25
-30
-35
-40
f1 (ppm)
0.6
0.5
0.4
0.3
0.2
0.1
0.0
-0.1
-0.2
f1 (ppm)

HRMS

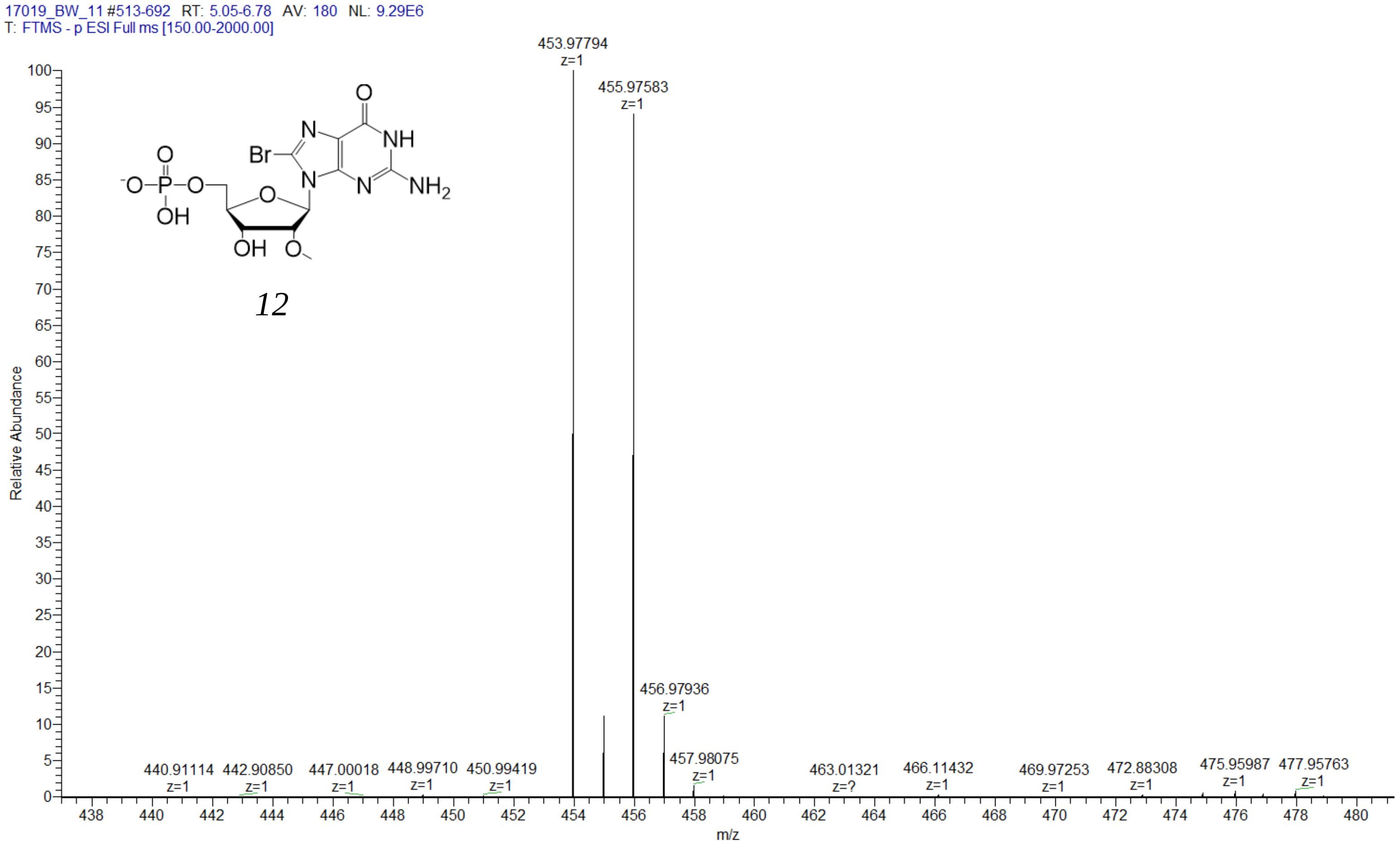

Summary

Rt (A) = 8.77 min; ^{1}H NMR (400 MHz, D_2O) δ = 6.03 (d, *J*=6.0 Hz, 1H), 5.02 (dd, *J*=6.0, 5.6 Hz, 1H), 4.76 (dd, *J*=5.6 Hz, 3.9 Hz, 1H), 4.27 (q, *J*=4.9 Hz, 4.2 Hz, 1H), 4.17 (m, 2H), 3.43 (s, 3H). ^{31}P NMR (162 MHz, D_2O) ^{31}P ^{15} δ = 0.24 (s, 1P); ^{31}P NMR δ = 0.24 (t, 1P, J= 5.9 Hz); HRMS ESI (-) m/z [M-H]$^-$, calcd for $C_{11}H_{14}BrN_5O_8P^-$; Exact Mass: 453.9769; found: 453.9779, 455.9758.

8-PyGMP (13a)

Structure

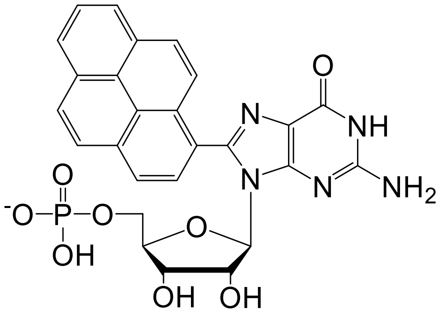

RP-HPLC profile

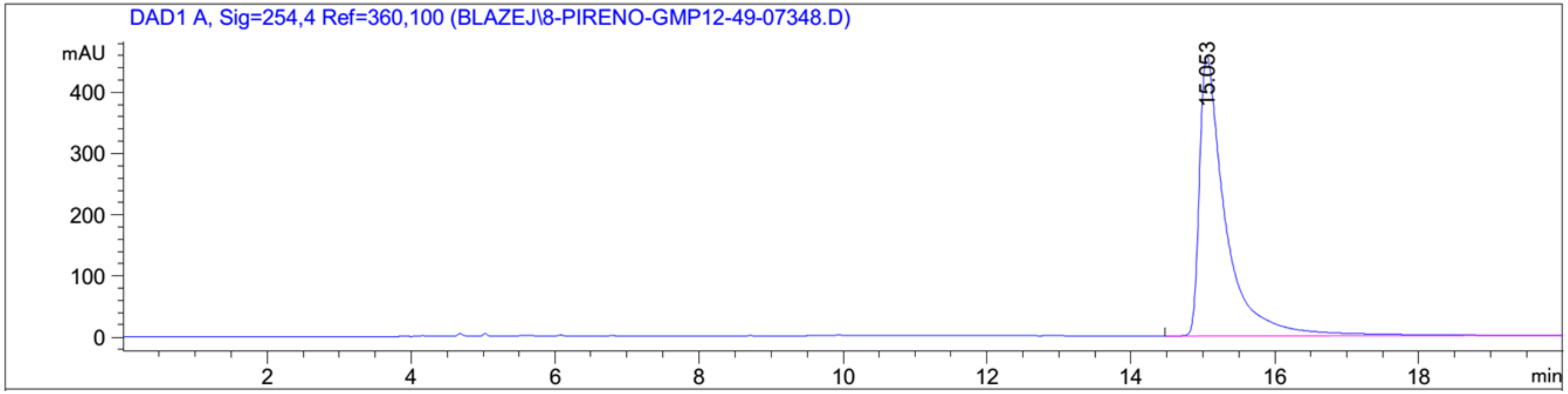

^{1}H NMR *(CD$_3$OD)*

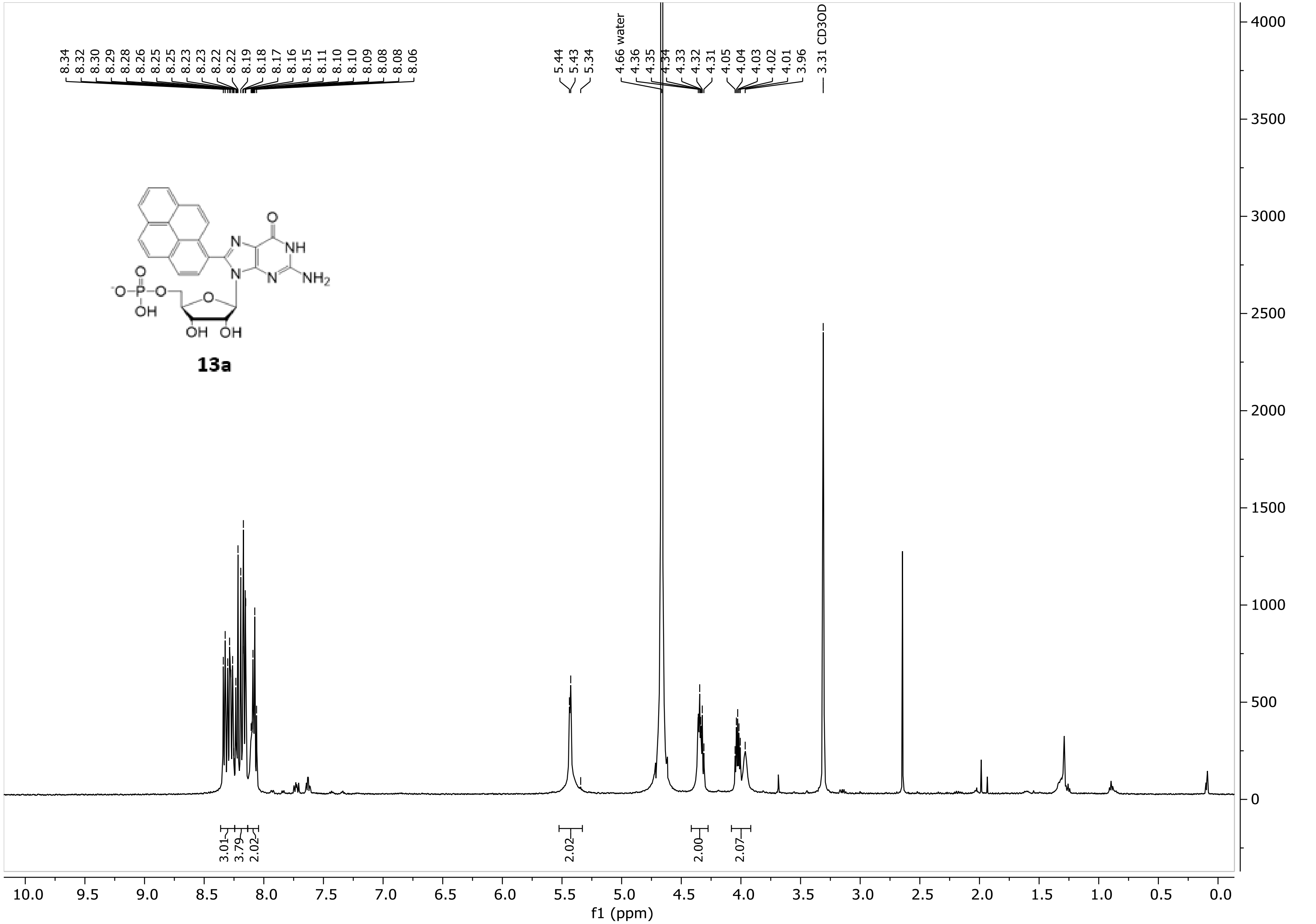

8.34 8.32 8.30 8.29 8.28 8.26 8.25 8.25 8.23 8.23 8.22 8.22 8.19 8.18 8.18 8.17 8.16 8.15 8.11 8.10 8.10 8.09 8.08 8.08 8.06
5.44 5.43 5.34
4.66 water 4.36 4.35 4.34 4.33 4.32 4.31 4.05 4.04 4.03 4.02 4.01 3.96
3.31 CD3OD
13a
3.01 3.79 2.02 2.02 2.00 2.07
f1 (ppm)

^{1}H-^{1}H COSY NMR (CD_3OD)

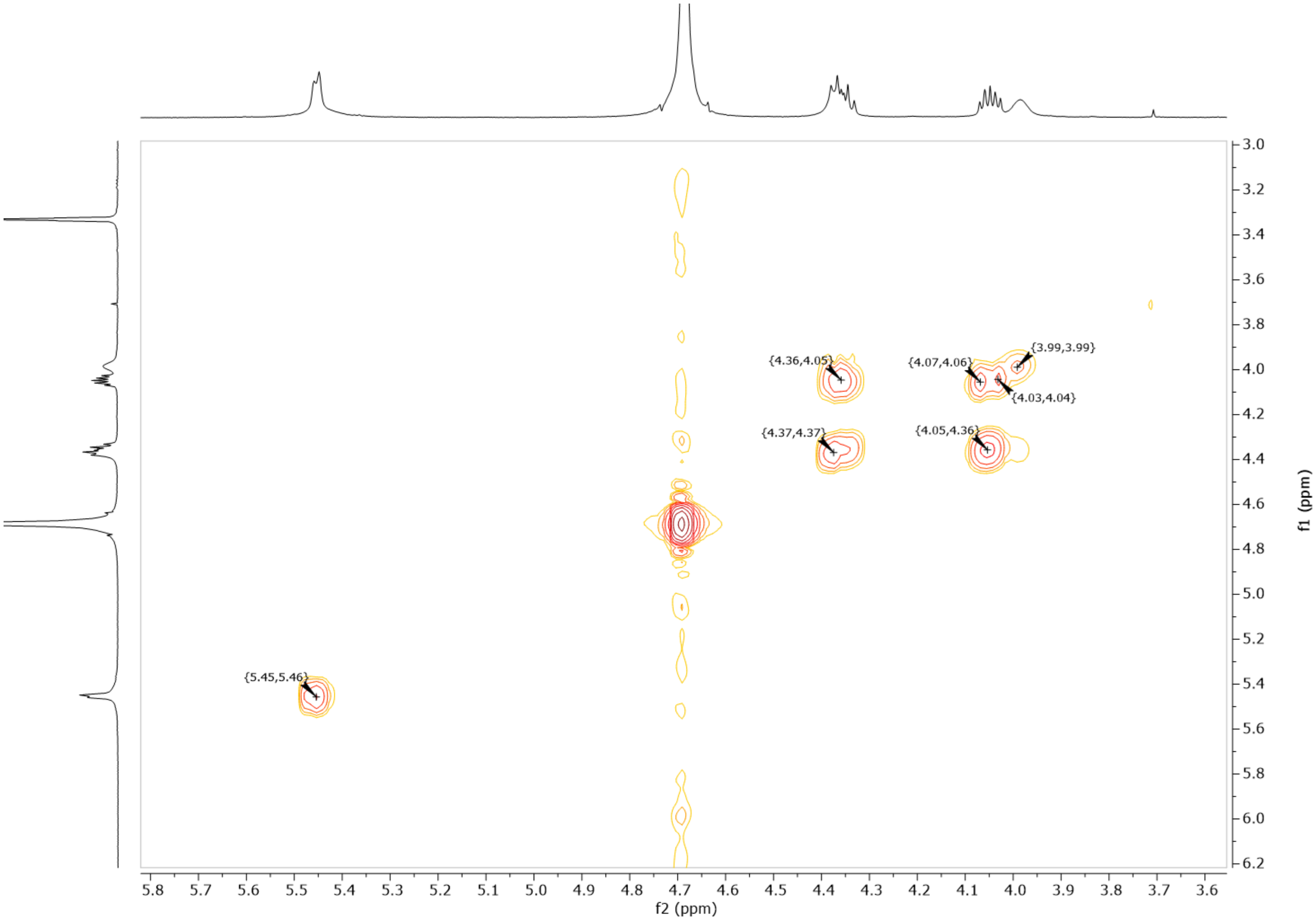

^{31}P NMR (DMSO-d6)

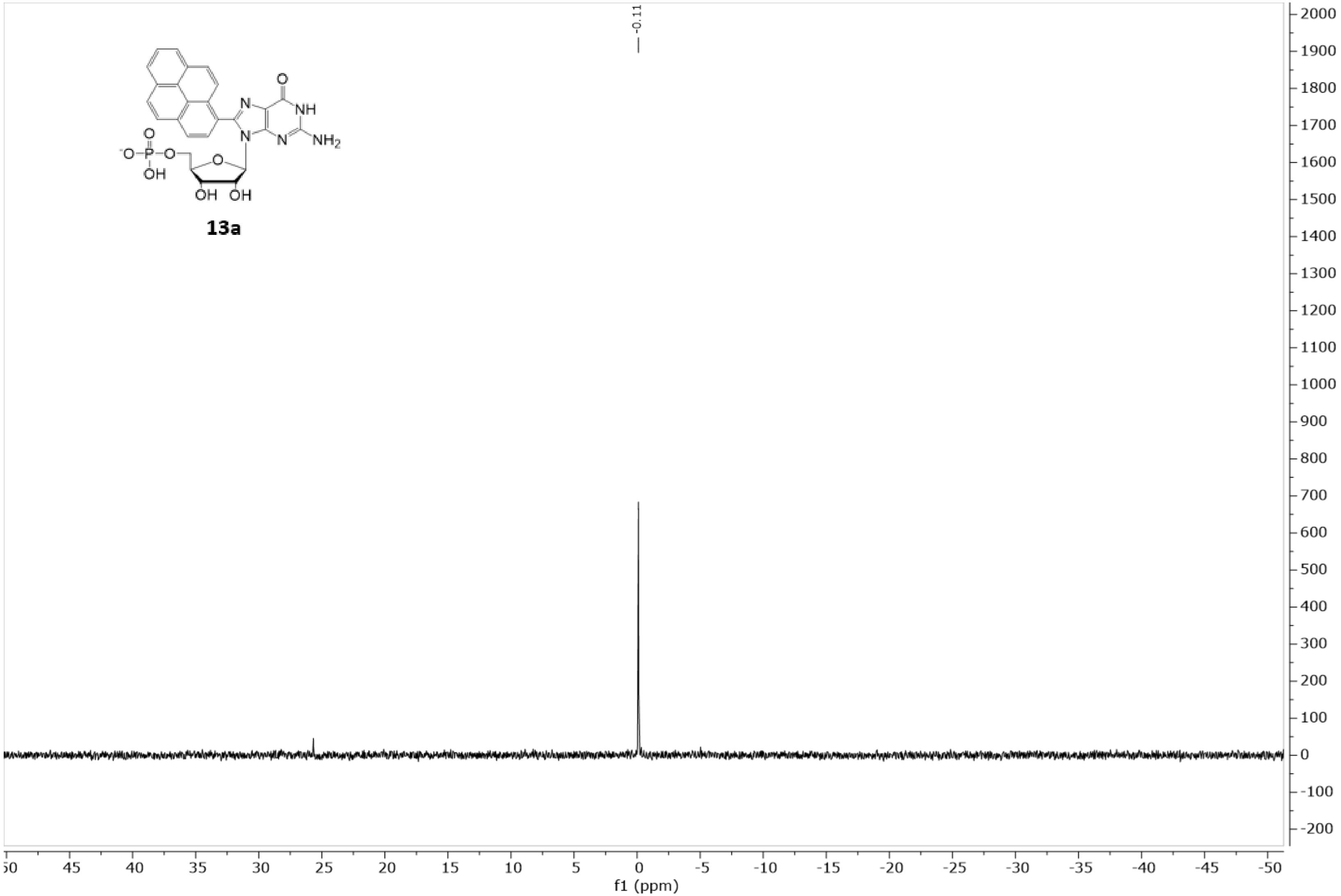

HSQC NMR *(CD$_3$OD)*

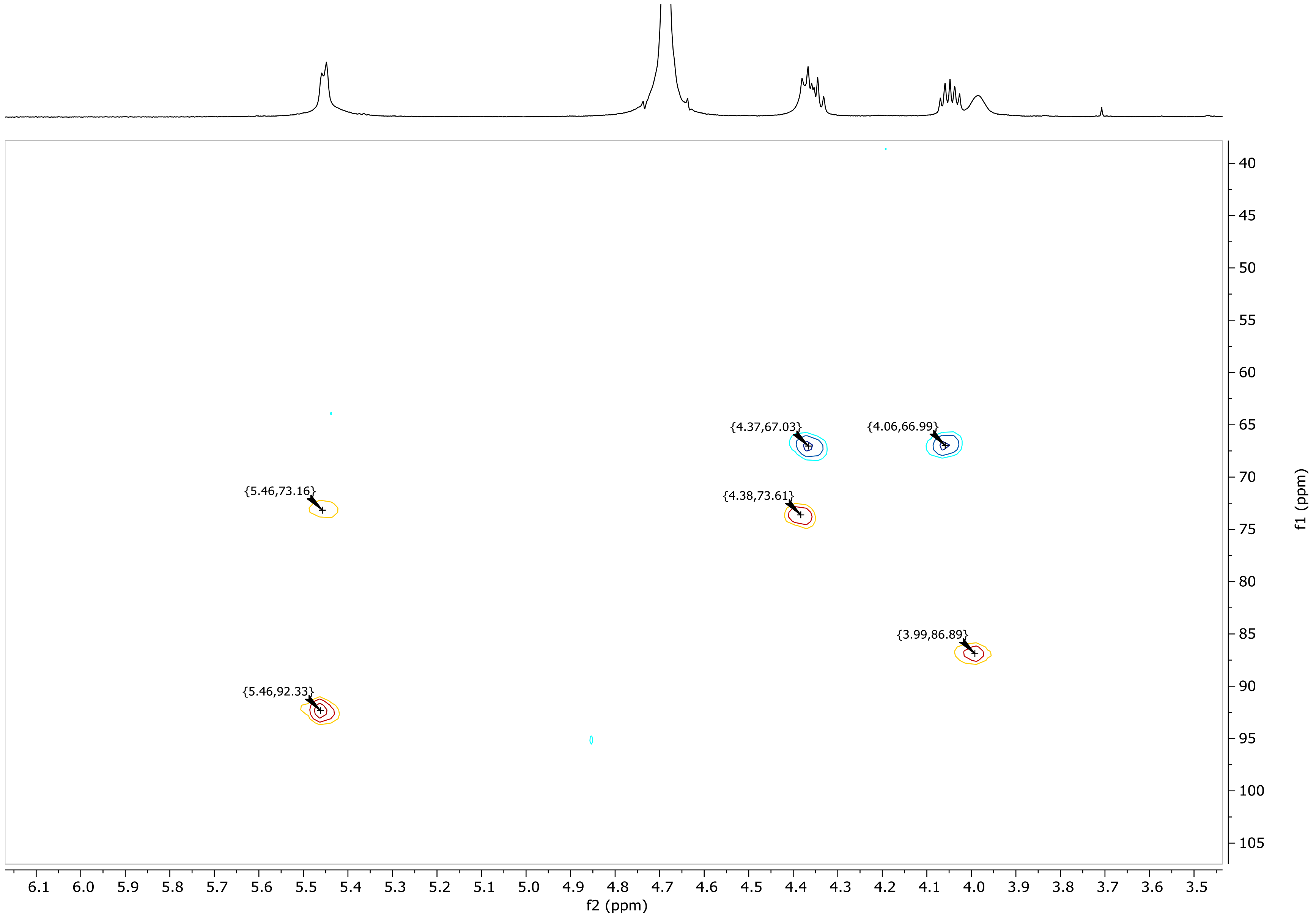

HRMS

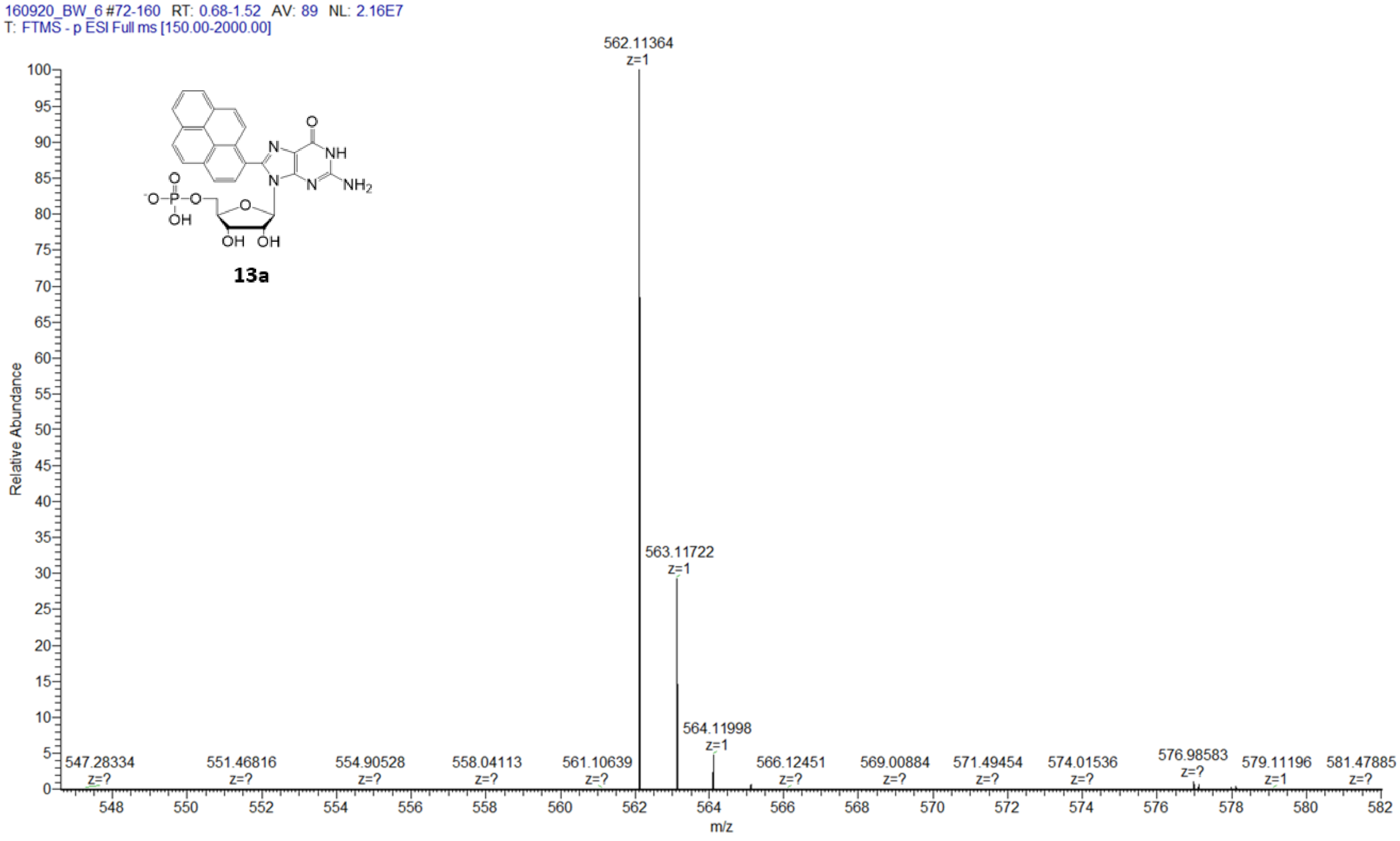

Summary

Rt (D) = 15.05 min; ^{1}H NMR (500 MHz, Methanol-d_4, 318 K) δ 8.36–8.24 (m, 3H), 8.24–8.14 (m, 4H), 8.08 (q, J = 7.7 Hz, 2H), 5.53–5.31 (m, 2H), 4.44–4.26 (m, 2H, H-3’, H-5’), 4.07–4.00 (m, 1H, H-5’’), 3.96 (bs, 1H, H-4’). ^{31}P NMR (203 MHz, DMSO-*d6*, 318 K) δ -0.11. HRMS ESI (-) m/z [M-H]$^-$, calcd for $C_{26}H_{21}N_5O_8P^-$ 562.1133; found 562.1136.

$^{8\text{-}Ph}$GMP (13b)

Structure

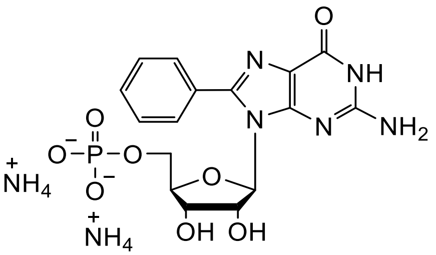

RP-HPLC profile

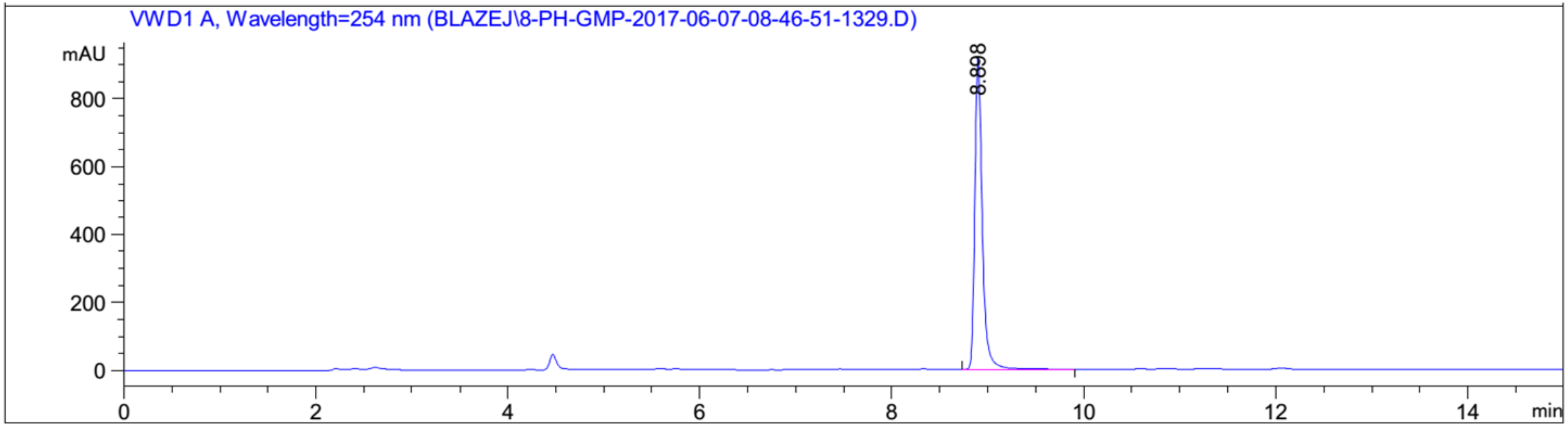
VWD1 A, Wavelength=254 nm (BLAZEJ\8-PH-GMP-2017-06-07-08-46-51-1329.D)
mAU
800
600
400
200
0
8.898
0
2
4
6
8
10
12
14
min

^{1}H NMR

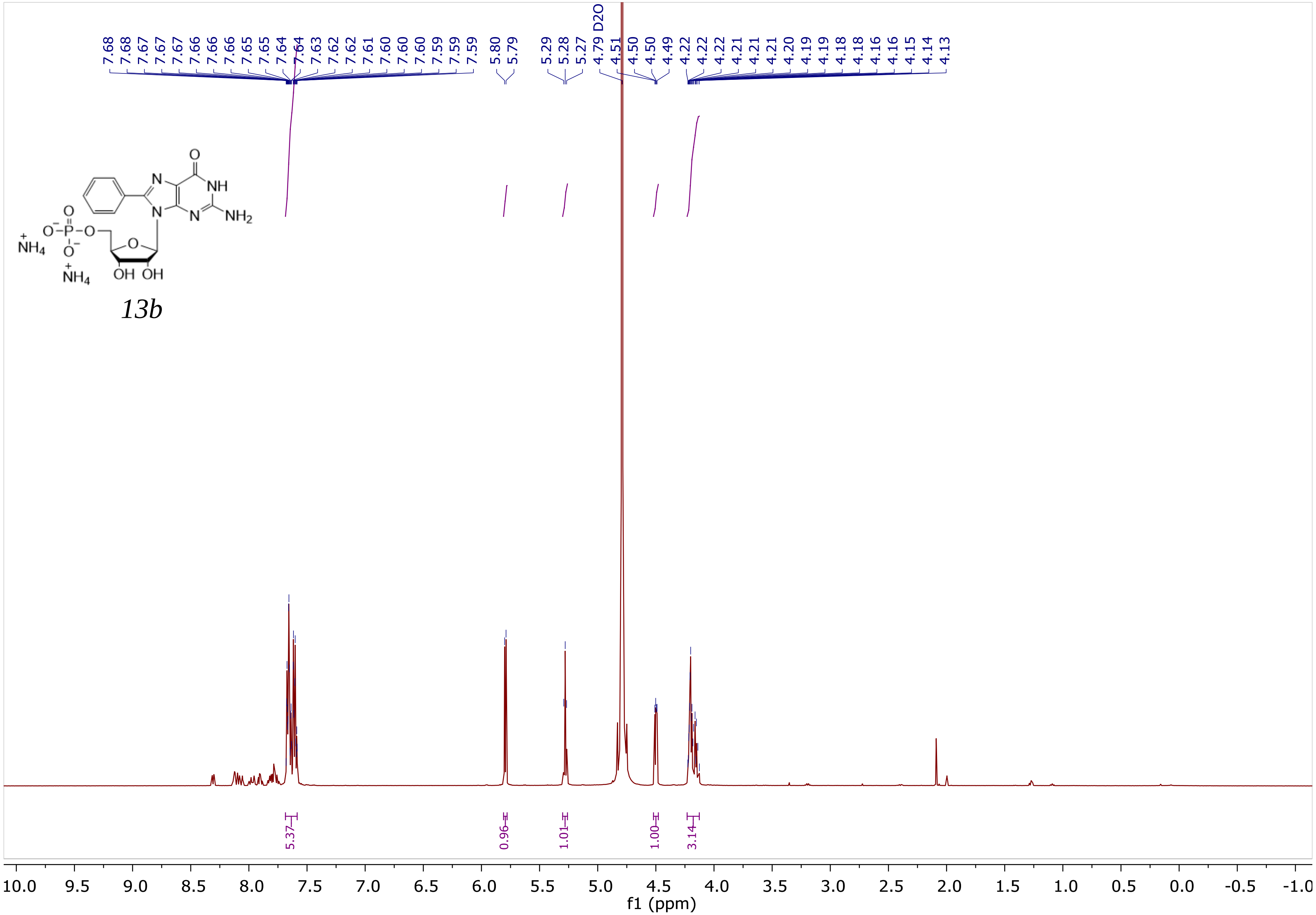

^{1}H-^{1}H COSY NMR

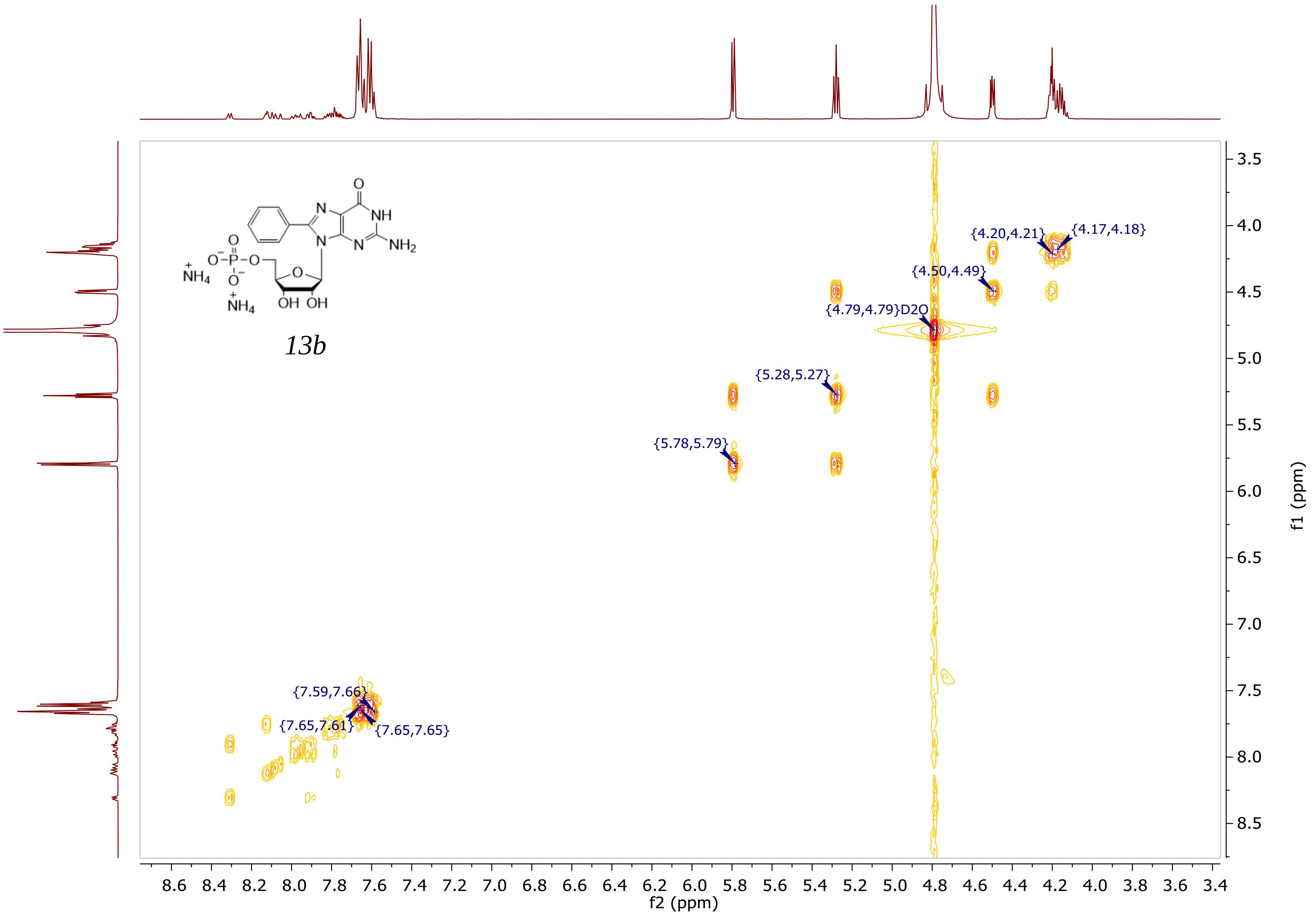
13b
{4.20,4.21}
{4.17,4.18}
{4.50,4.49}
{4.79,4.79}D2O
{5.28,5.27}
{5.78,5.79}
{7.59,7.66}
{7.65,7.61}
{7.65,7.65}
f2 (ppm)
f1 (ppm)

^{31}P NMR

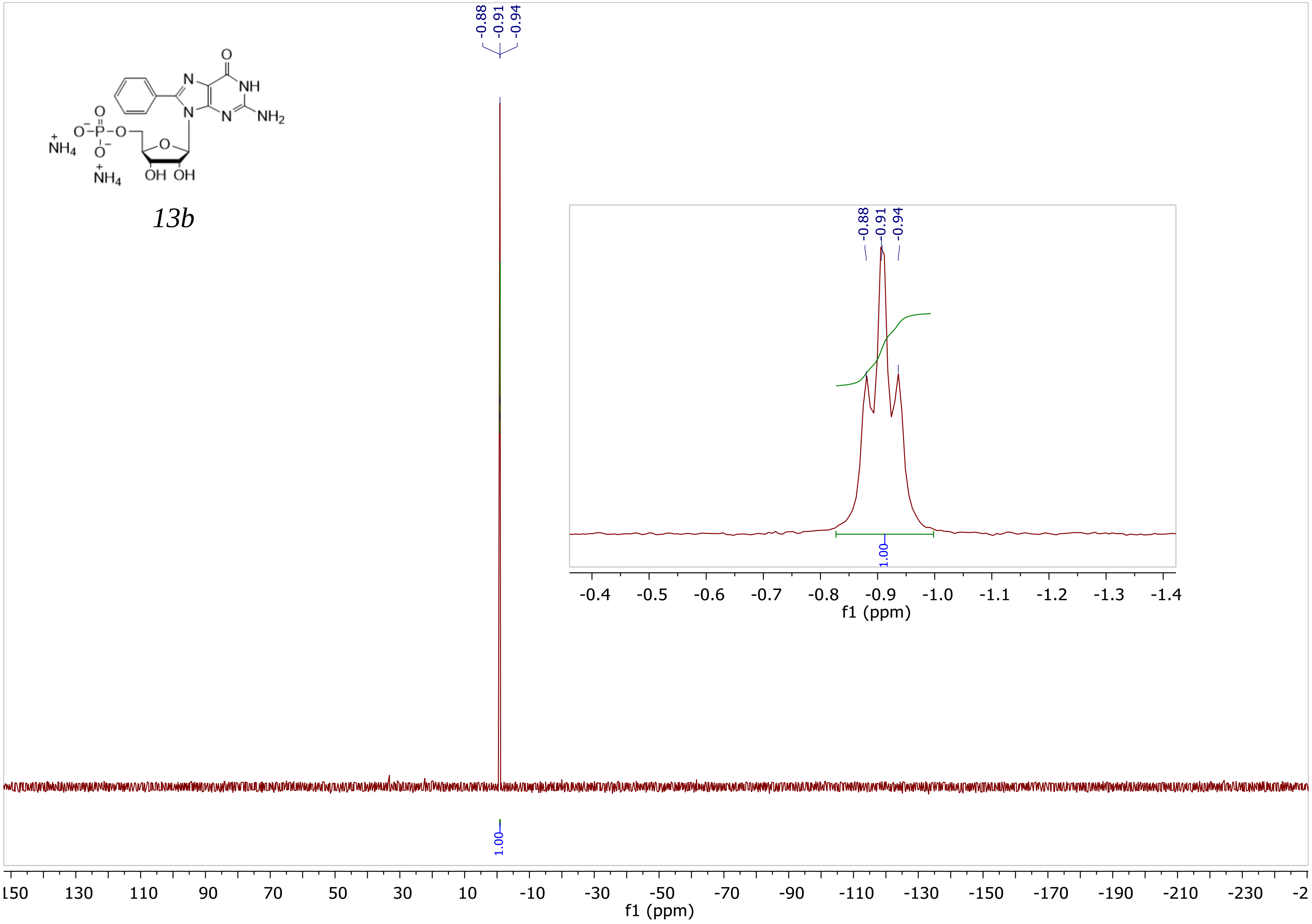
O
N
NH
NH2
N
N
O
P
O
O
NH4
O
NH4
OH OH
13b
-0.88
-0.91
-0.94
1.00
f1 (ppm)
150 130 110 90 70 50 30 10 -10 -30 -50 -70 -90 -110 -130 -150 -170 -190 -210 -230
-0.4 -0.5 -0.6 -0.7 -0.8 -0.9 -1.0 -1.1 -1.2 -1.3 -1.4

HRMS

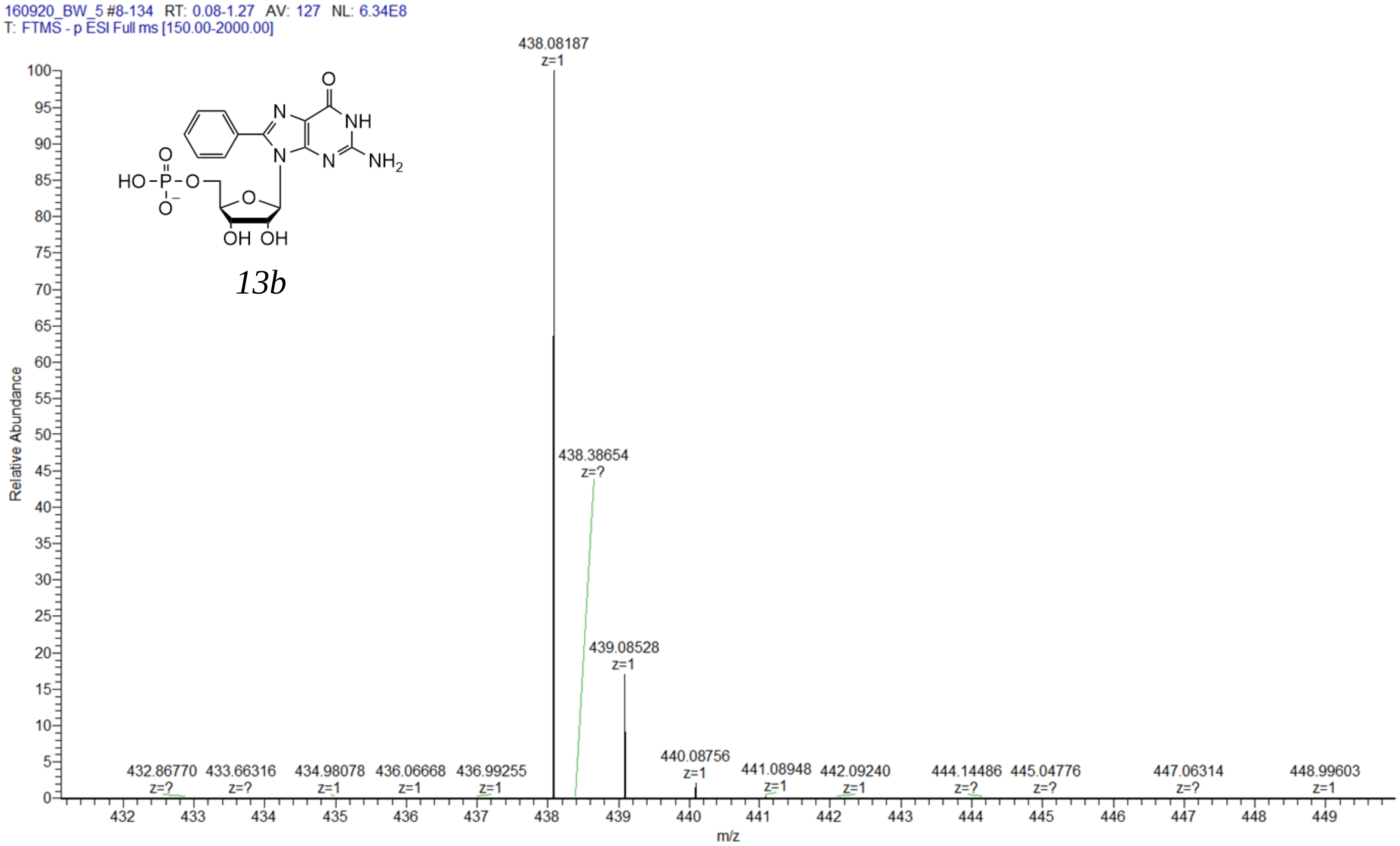

Summary

Rt (D) = 8.90 min; ^{1}H NMR (500 MHz, D_2O) δ 7.73-7.53 (m, 5H), 5.79 (d, *J* = 6.2 Hz, 1H), 5.28 (dd, *J* = 6.2 Hz, 5.7 Hz, 1H), 4.50 (dd, *J* = 5.7 Hz, 3.5 Hz, 1H), 4.23-4.12 (m, 3H); ^{31}P NMR (202 MHz, D_2O) δ –0.91 (t, *J* = 5.7 Hz, 1P); HRMS ESI (-) m/z [M-H]$^-$, calcd for $C_{16}H_{17}N_5O_8P^-$ 438.0820; found 438.0819.

^{8DMAPh}*GMP (13c)*

Structures

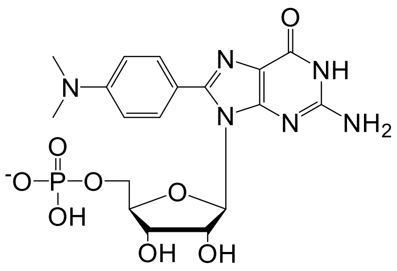

RP-HPLC profile

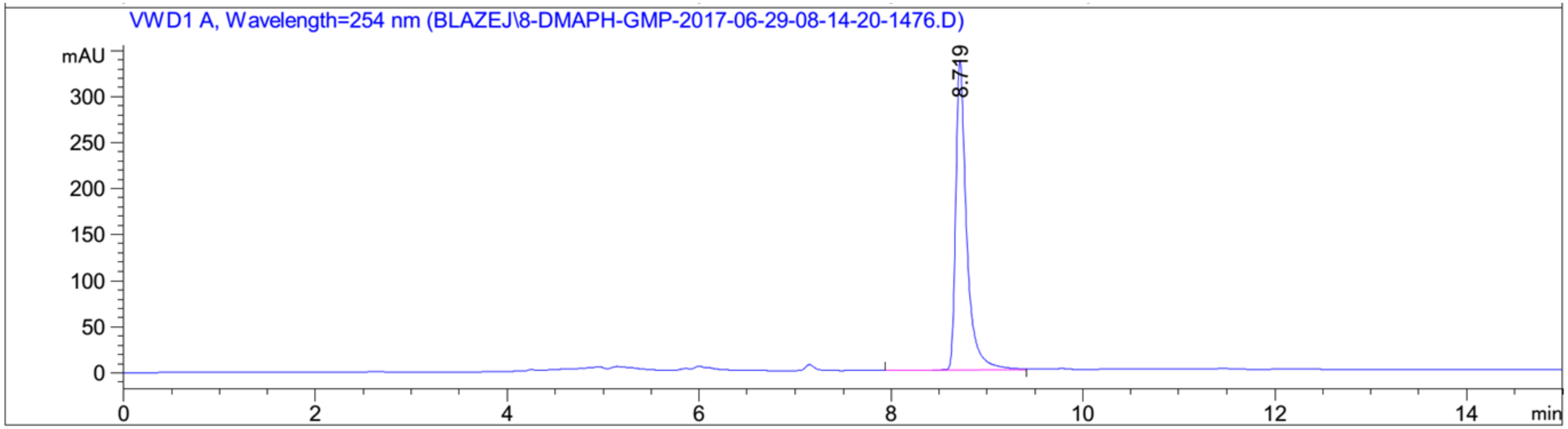

VWD1 A, Wavelength=254 nm (BLAZEJ\8-DMAPH-GMP-2017-06-29-08-14-20-1476.D)
mAU
300
250
200
150
100
50
0
8.719
0
2
4
6
8
10
12
14
min

^{1}H NMR

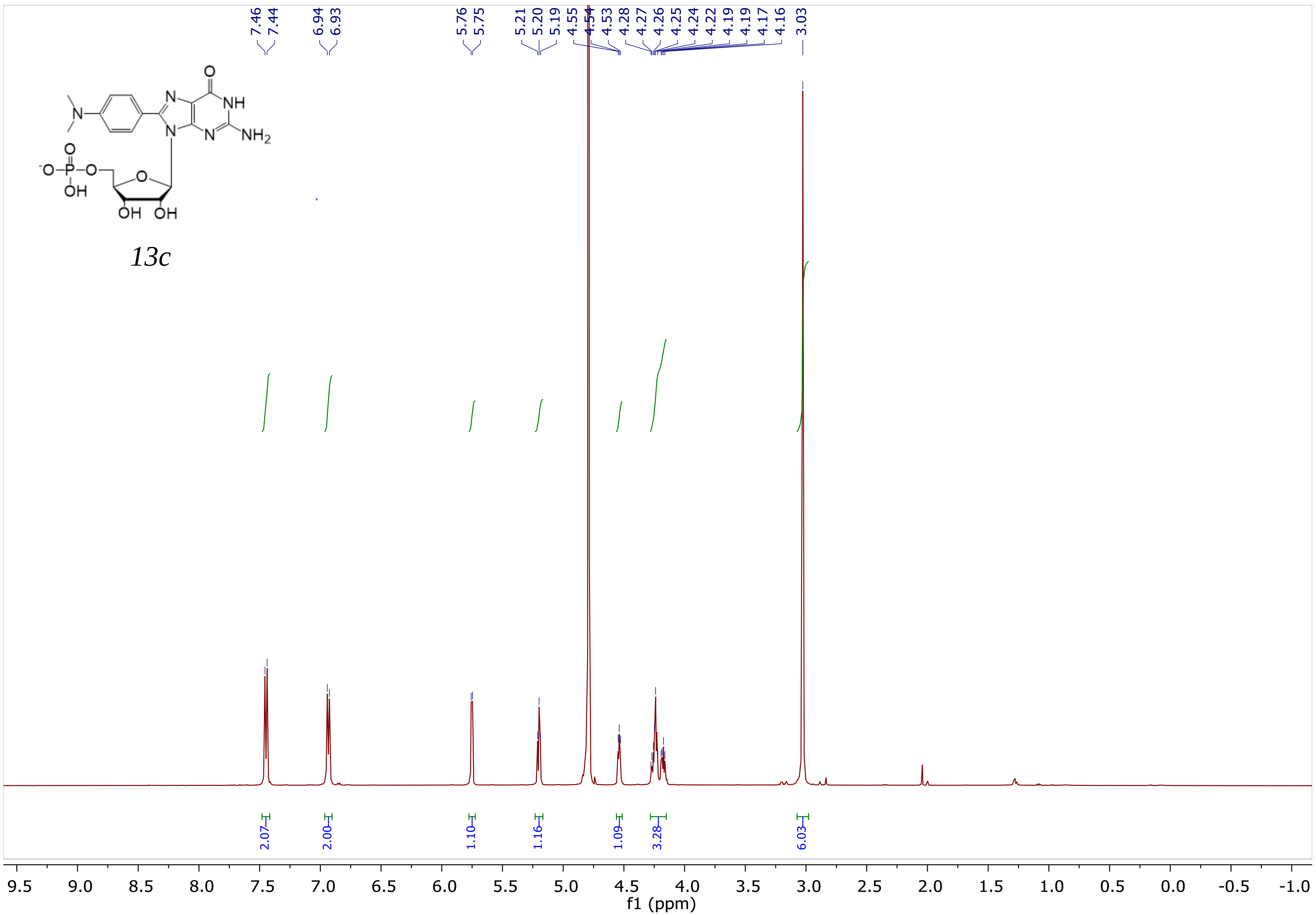
7.46
7.44
6.94
6.93
5.76
5.75
5.21
5.20
5.19
4.55
4.54
4.53
4.28
4.27
4.26
4.25
4.24
4.22
4.19
4.19
4.17
4.16
3.03
13c
2.07
2.00
1.10
1.16
1.09
3.28
6.03
9.5
9.0
8.5
8.0
7.5
7.0
6.5
6.0
5.5
5.0
4.5
4.0
3.5
3.0
2.5
2.0
1.5
1.0
0.5
0.0
-0.5
-1.0
f1 (ppm)

^{31}P NMR

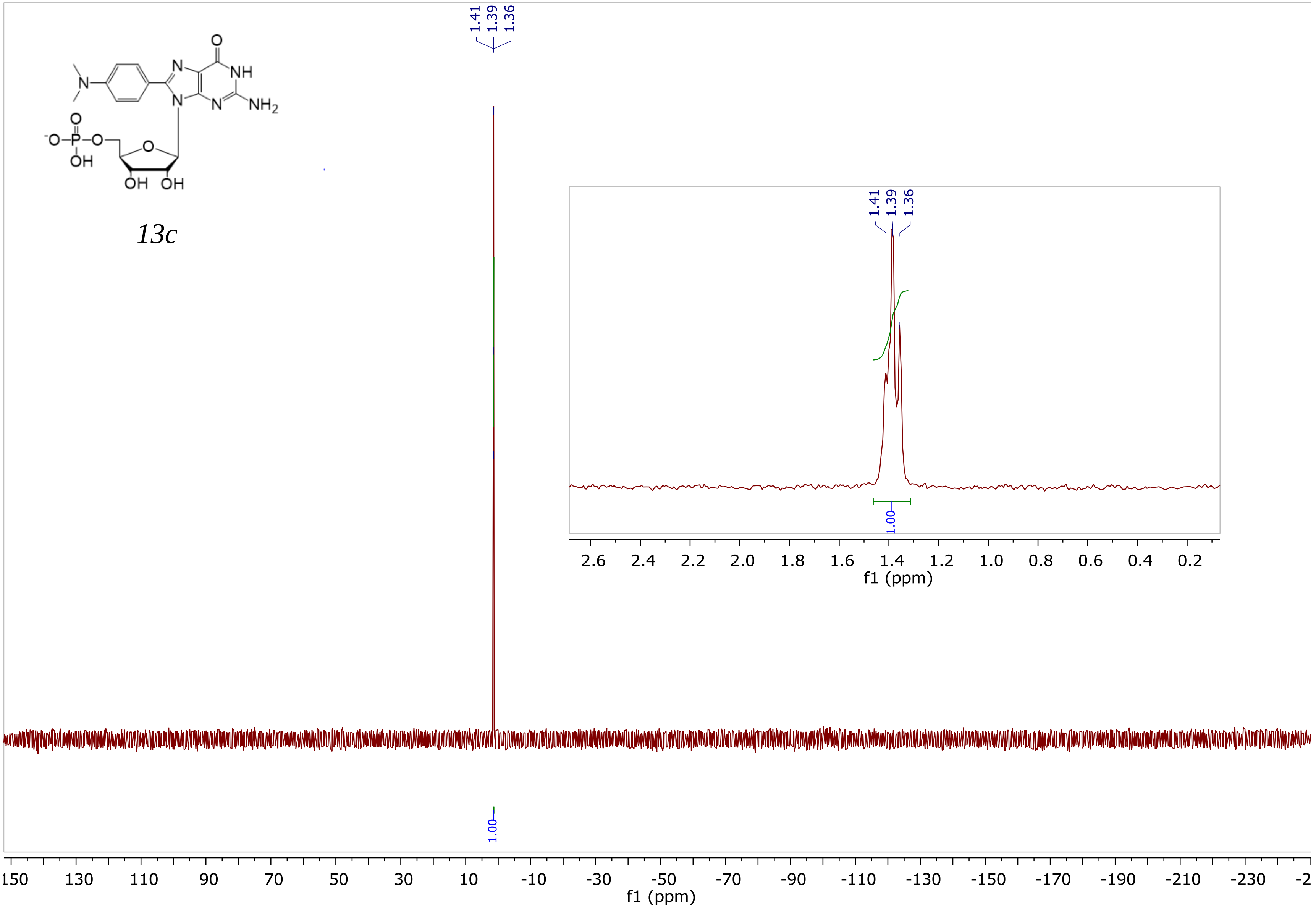

HRMS

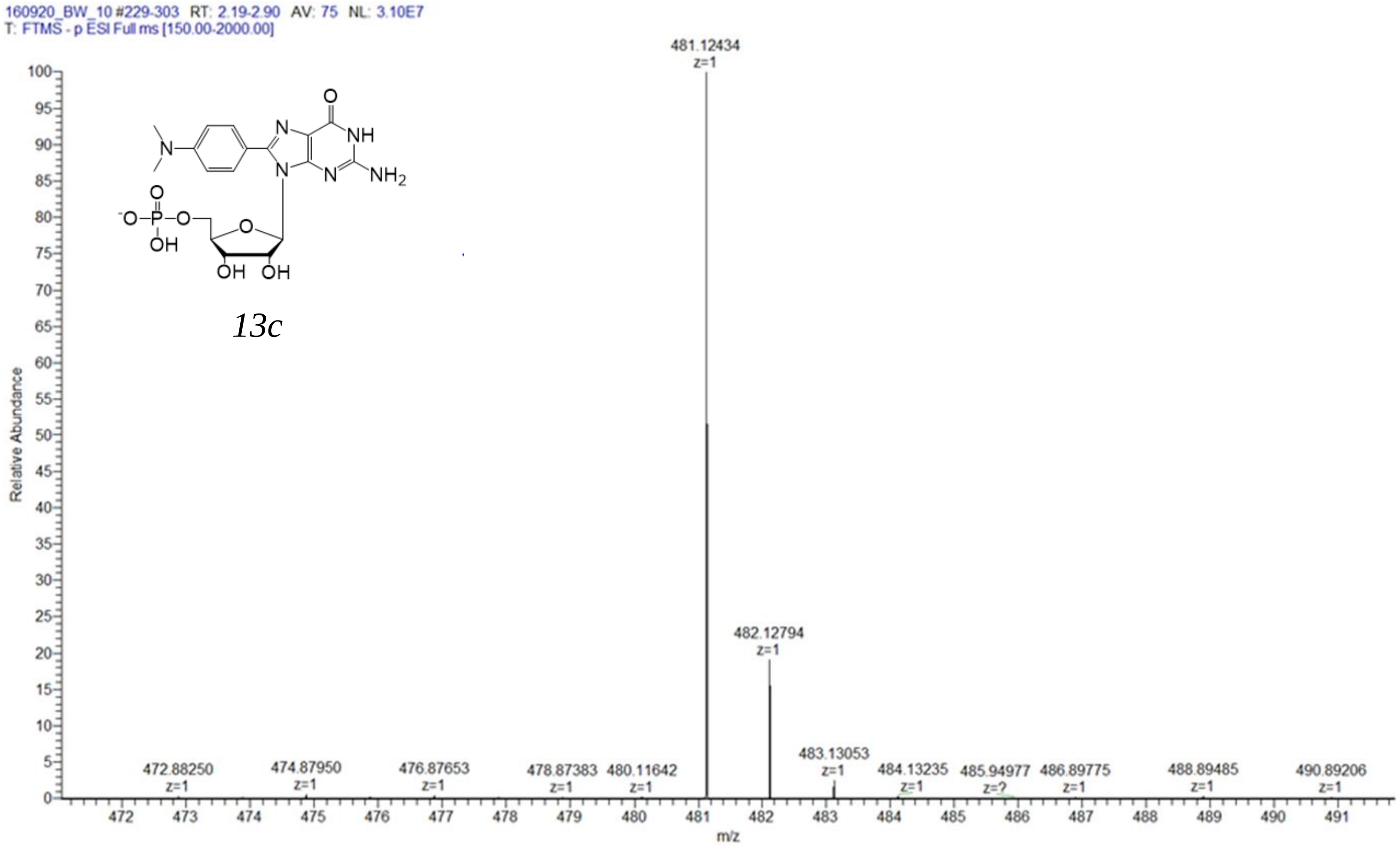

Summary

Rt (D) = 8.72 min; ^{1}H NMR (500 MHz, D_2O) δ 7.45 (d, *J* = 8.7 Hz, 2H), 6.93 (d, *J* = 8.7 Hz, 2H), 5.75 (d, *J* = 5.7 Hz, 1H), 5.20 (t, *J* = 5.7 Hz, 1H), 4.54 (m, 1H), 4.21 (m, 3H), 3.03 (s, 6H); ^{31}P NMR (202 MHz, D_2O) δ 1.39 (t, *J* = 5.6 Hz, 1P); HRMS ESI (-) m/z [M-H]$^-$, calcd for $C_{18}H_{22}N_6O_8P^-$ 481.1242; found 481.1243.

$^{8\text{-}CNPh}$GMP (13d)

Structure

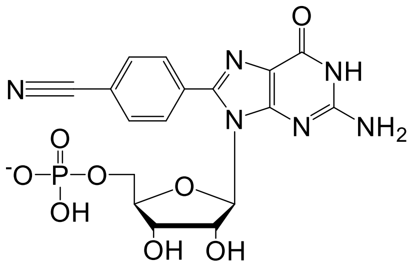

RP-HPLC profile

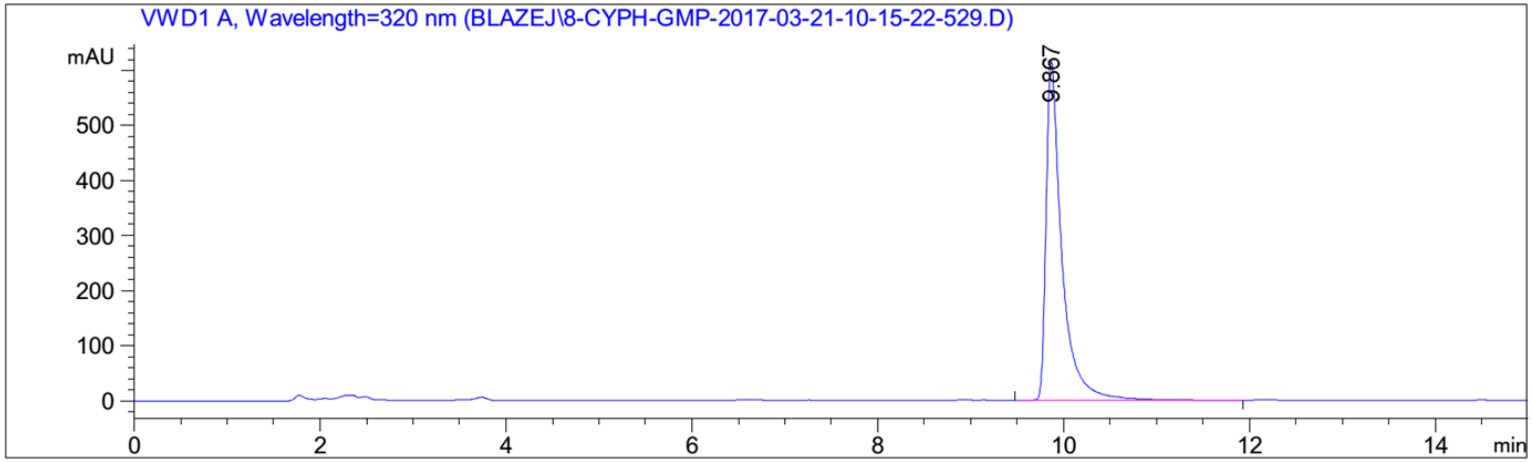

^1H NMR

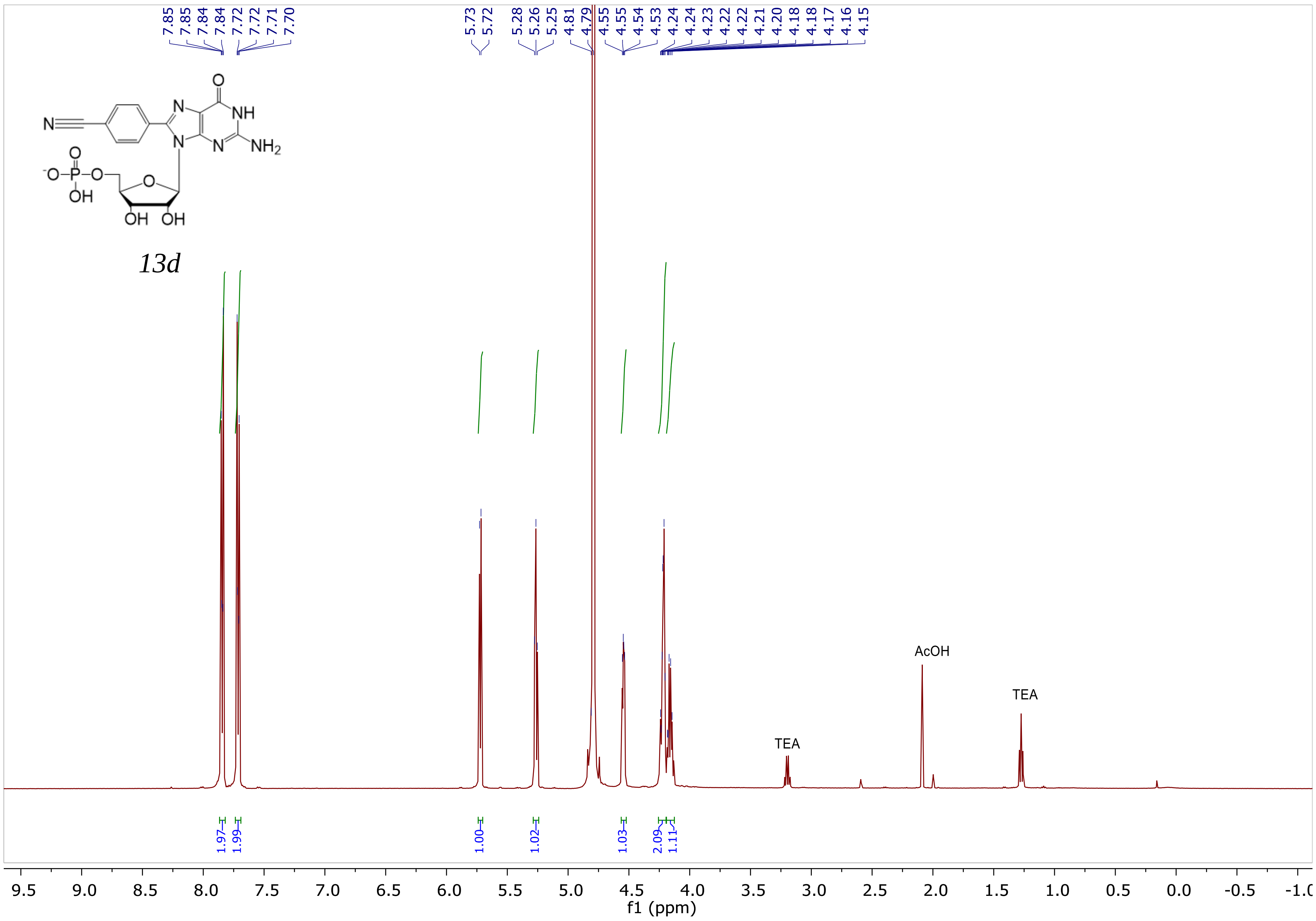
7.85 7.85 7.84 7.84 7.72 7.72 7.71 7.70
5.73 5.72 5.28 5.26 5.25 4.81 4.79 4.55 4.55 4.54 4.53 4.24 4.24 4.23 4.22 4.22 4.21 4.20 4.18 4.18 4.17 4.16 4.15
13d
TEA
AcOH
TEA
1.97 1.99 1.00 1.02 1.03 2.09 1.11
f1 (ppm)

^{31}P NMR

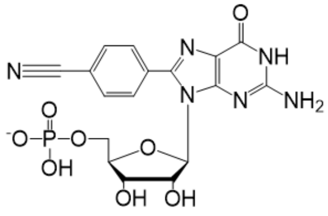

13d

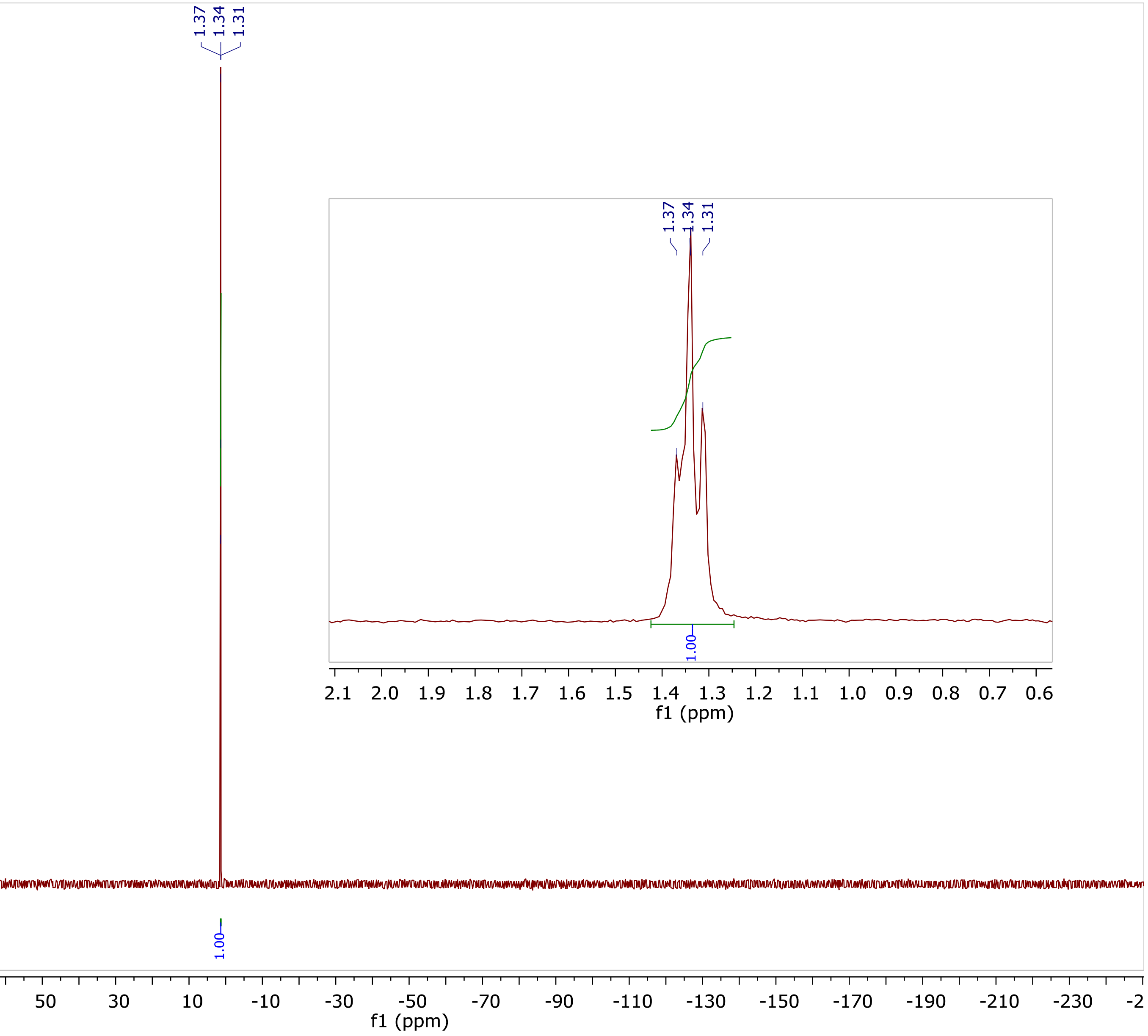
1.37
1.34
1.31
1.00
2.1 2.0 1.9 1.8 1.7 1.6 1.5 1.4 1.3 1.2 1.1 1.0 0.9 0.8 0.7 0.6
f1 (ppm)
150 130 110 90 70 50 30 10 -10 -30 -50 -70 -90 -110 -130 -150 -170 -190 -210 -230 -2
f1 (ppm)

HRMS

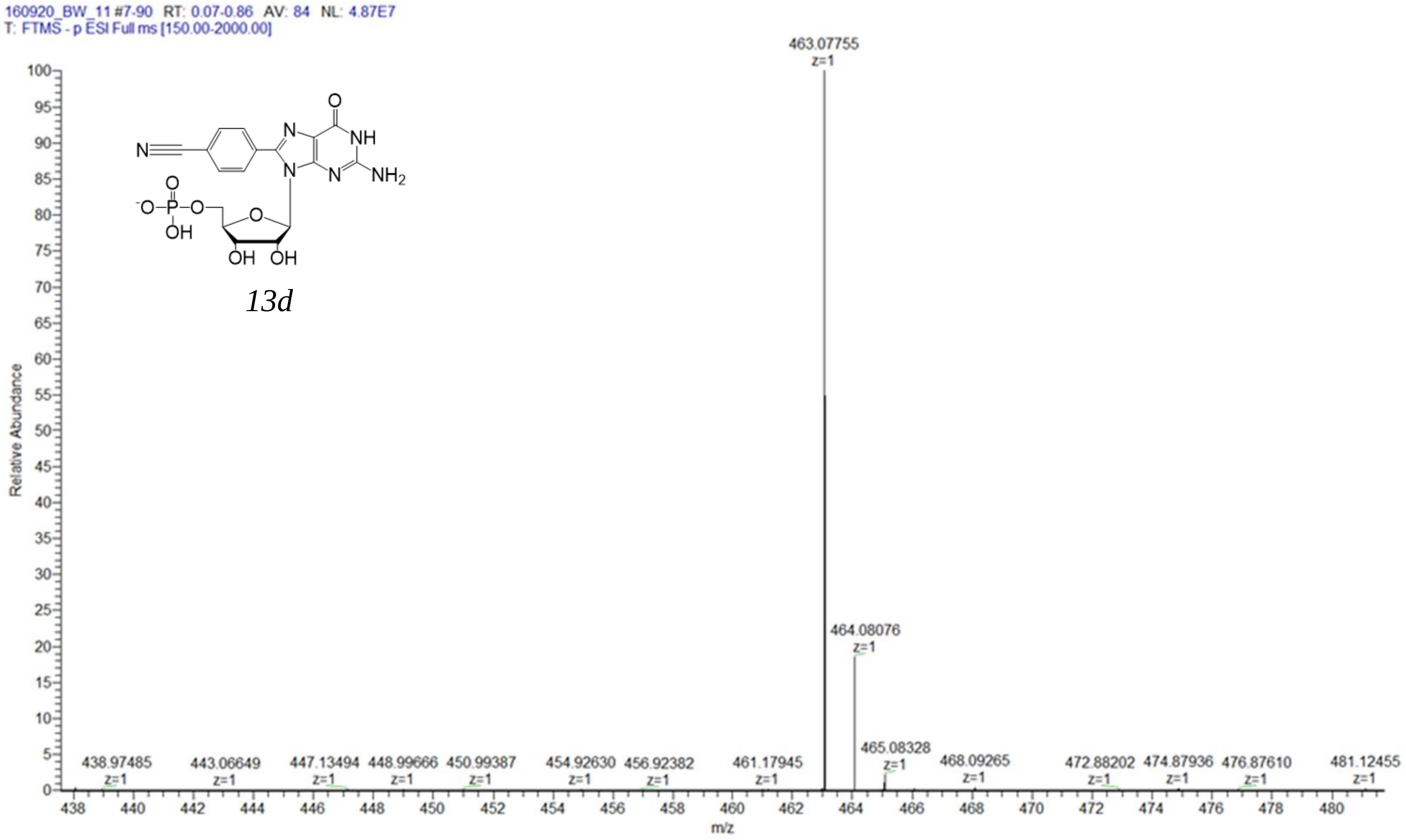

13d

Summary

Rt (D) = 9.87 min; ^{1}H NMR (500 MHz, D_2O) δ 7.84 (m, 2H, H_{ar}), 7.71 (m, 2H, H_{ar}), 5.72 (d, J = 6.0 Hz, 1H), 5.26 (dd, J = 6.0 Hz, 5.8 Hz, 1H), 4.54 (dd, J = 5.8 Hz, 3.7 Hz, 1H), 4.22 (m, 2H), 4.16 (m, 1H); ^{31}P NMR (202 MHz, D_2O) δ 1.34 (t, J = 5.6 Hz, 1P); HRMS ESI (-) m/z [M-H]$^-$, calcd for $C_{17}H_{16}N_6O_8P^-$ 463.0773; found 463.0776.

$^{8\text{-Me}}$GMP (13e)

Structure

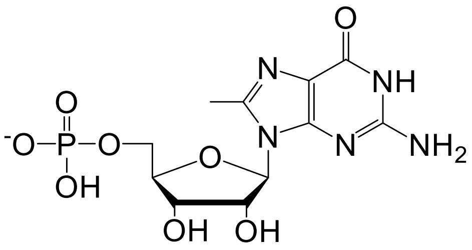

RP-HPLC profile

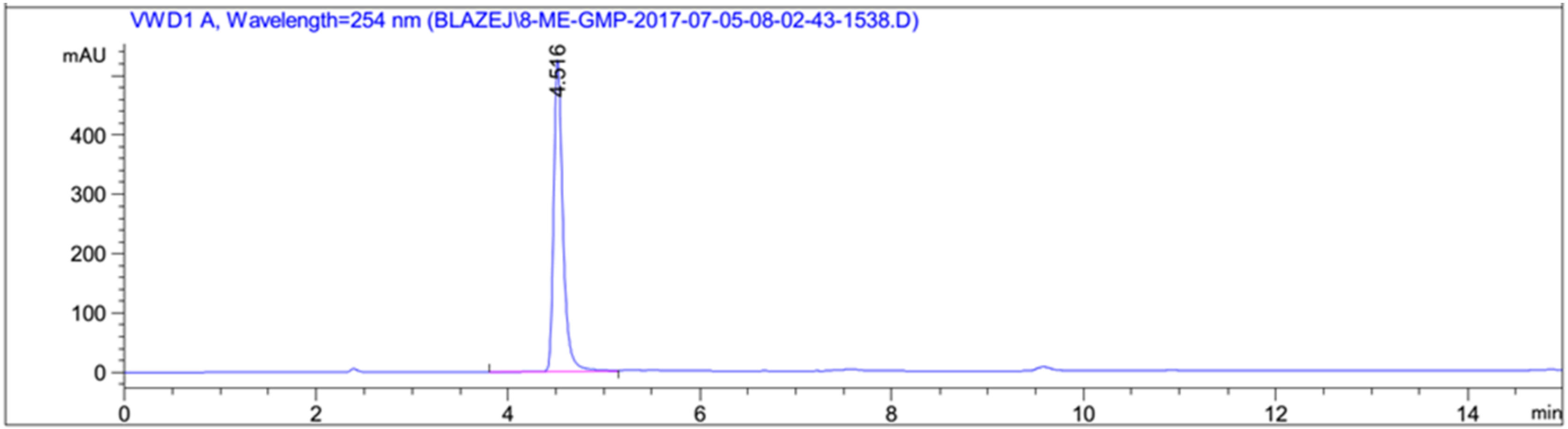

^{1}H NMR

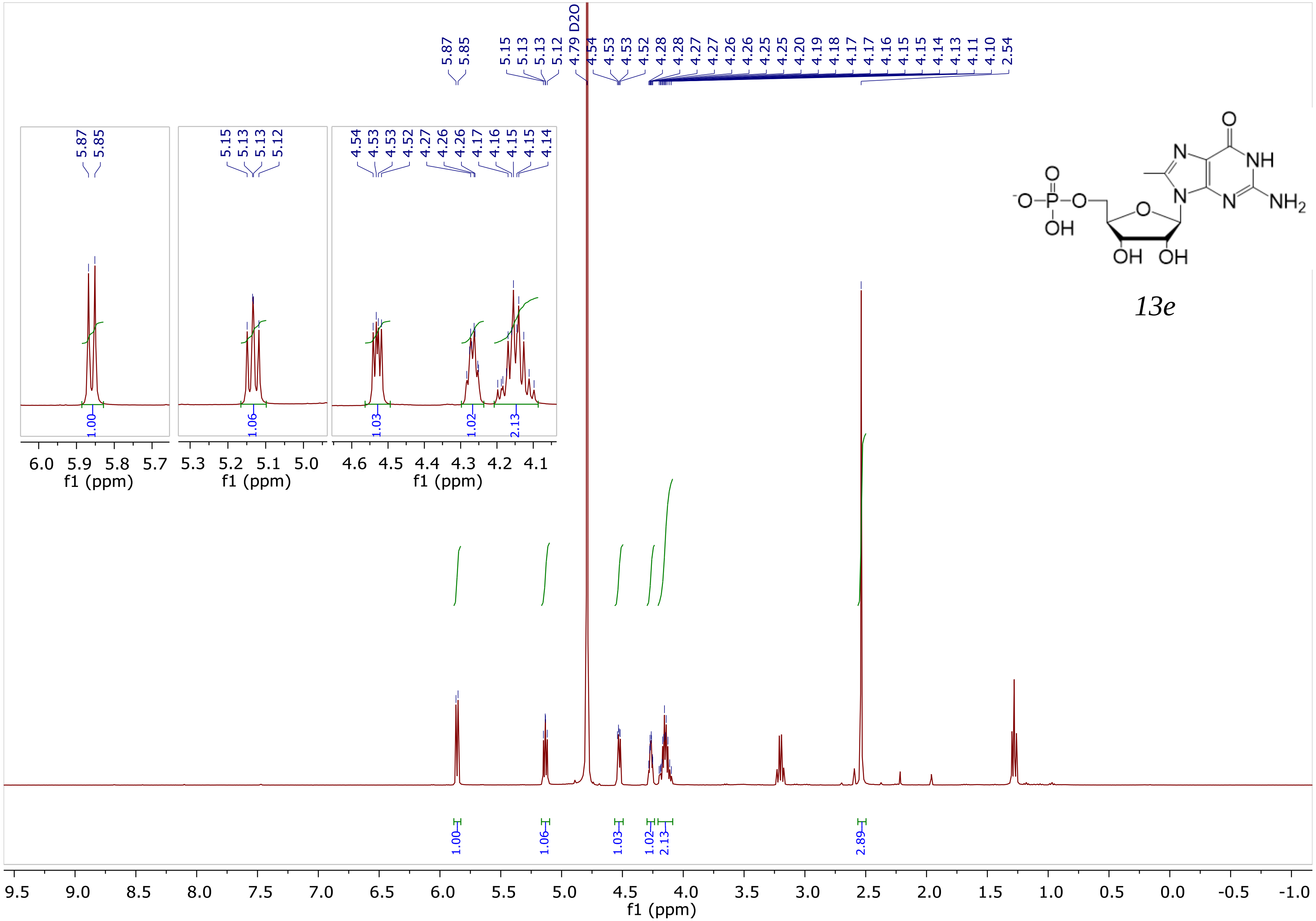
5.87
5.85
5.15
5.13
5.13
5.12
4.79 D2O
4.54
4.53
4.53
4.52
4.28
4.28
4.27
4.27
4.26
4.26
4.25
4.25
4.20
4.19
4.18
4.17
4.17
4.16
4.15
4.15
4.14
4.13
4.11
4.10
2.54
1.00
1.06
1.03
1.02
2.13
2.89
f1 (ppm)

13e

^{31}P NMR

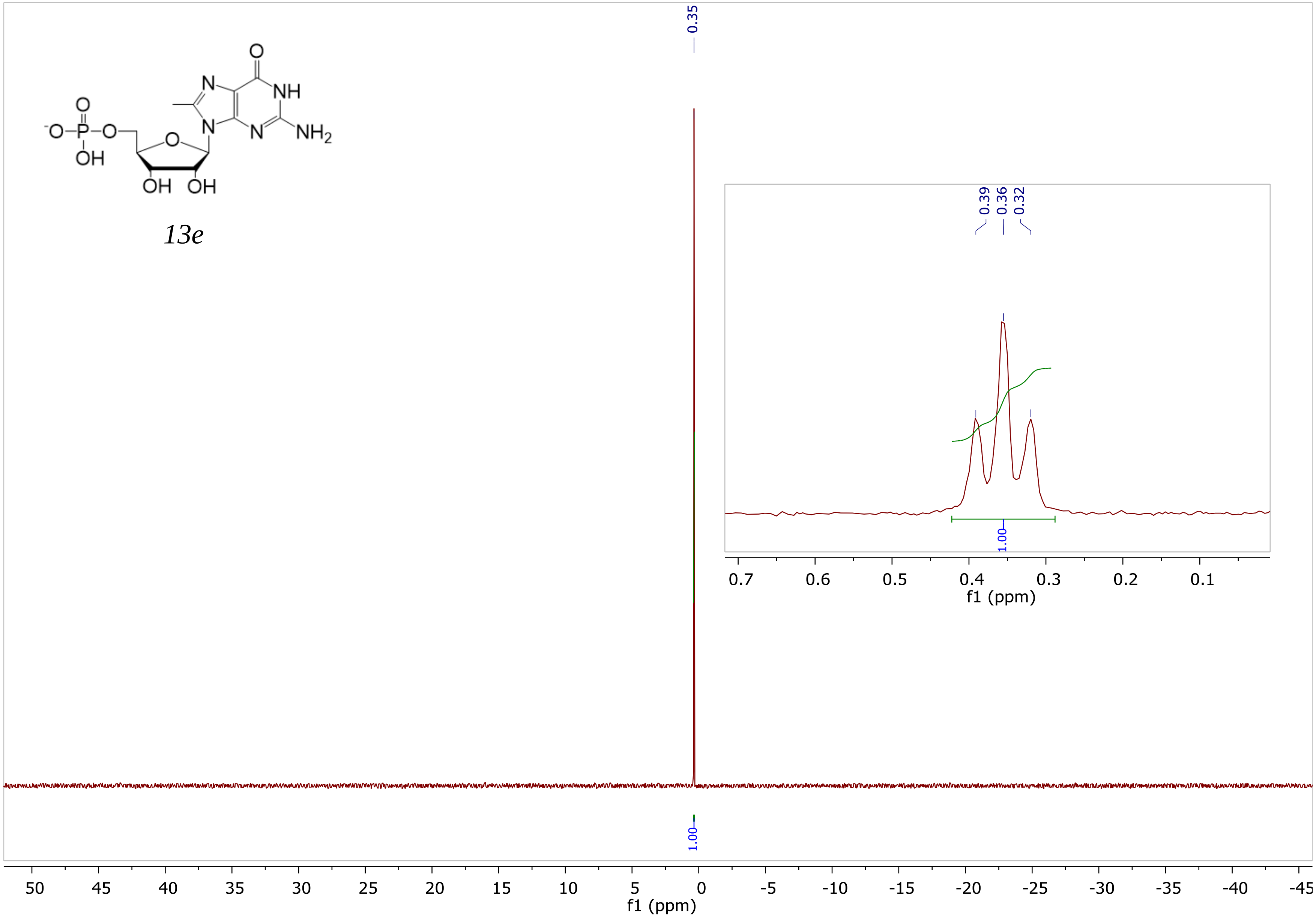

13e

0.35
0.39
0.36
0.32
1.00
0.7
0.6
0.5
0.4
0.3
0.2
0.1
f1 (ppm)
1.00
50
45
40
35
30
25
20
15
10
5
0
-5
-10
-15
-20
-25
-30
-35
-40
-45
f1 (ppm)

HRMS

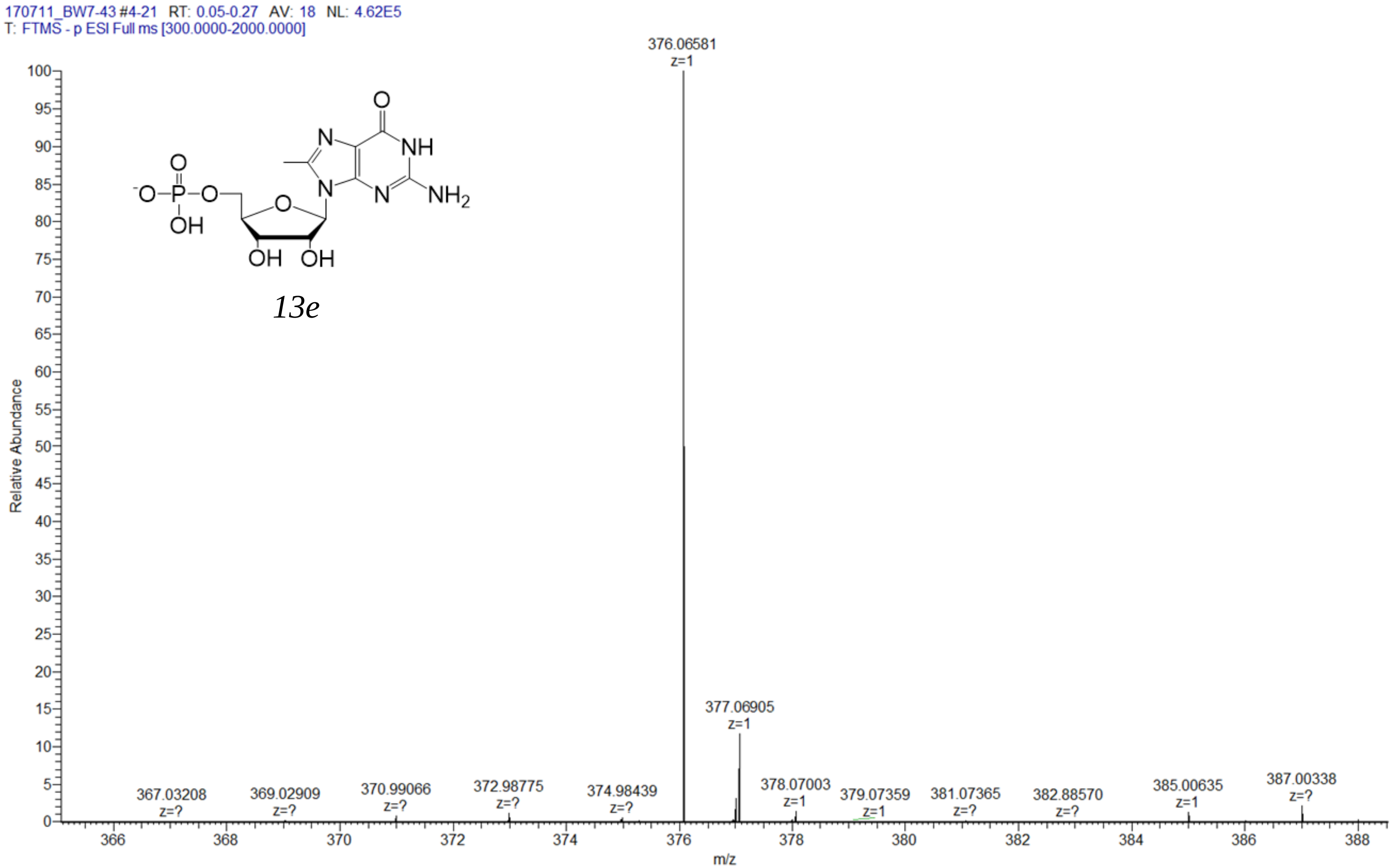

Summary

Rt (A) = 4.52 min; ^{1}H NMR (400 MHz, deuterium oxide) δ 5.86 (d, *J* = 6.7 Hz, 1H), 5.13 (dd, *J* = 6.7 Hz, 5.8 Hz, 1H), 4.53 (dd, *J* = 5.8 Hz, 3.5 Hz, 1H), 4.27 (q, *J* = 4.5 Hz, 3.5 Hz, 1H), 4.15 (m, 2H), 2.54 (s, 3H); ^{31}P NMR (202 MHz, D_2O) δ 0.36 (t, *J* = 5.8 Hz, 1P); HRMS ESI (-) m/z [M-H]$^-$, calcd for $C_{11}H_{15}N_5O_8P^-$ 376.0669; found 376.0658.

$^{8Ph}m^{2'O}$GMP (14b)

Structure

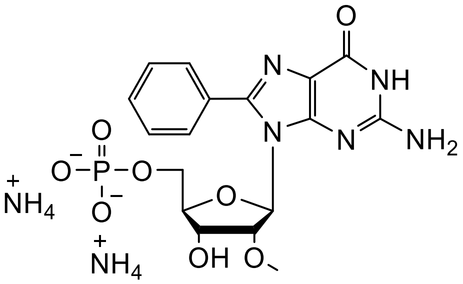

RP-HPLC profile

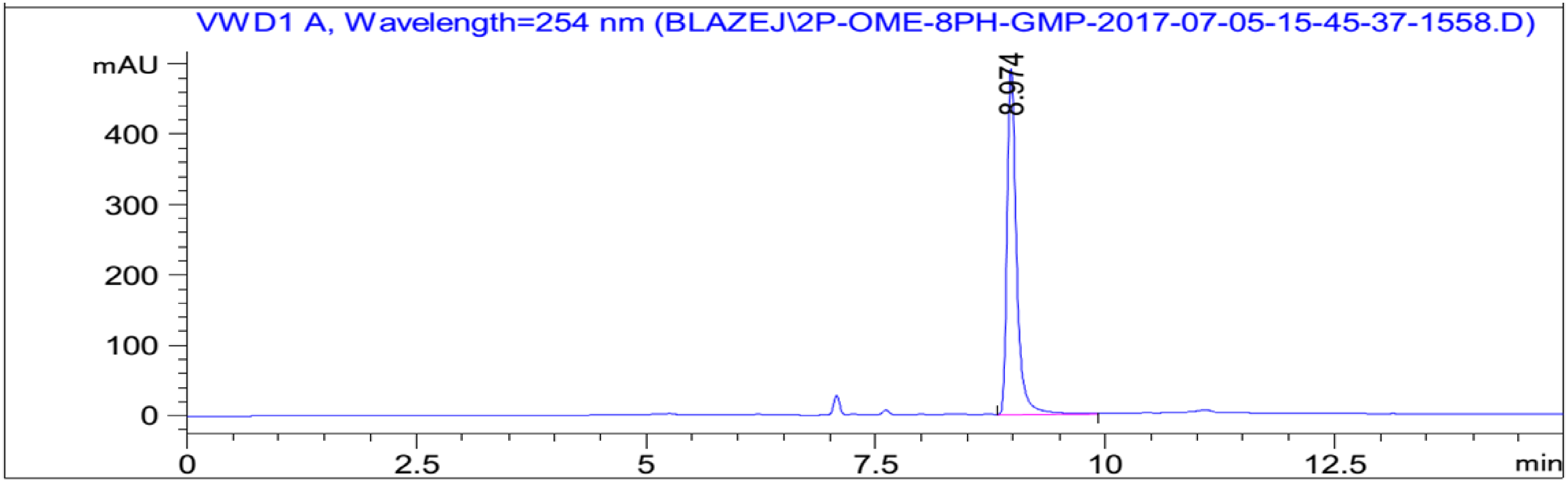

^{1}H NMR

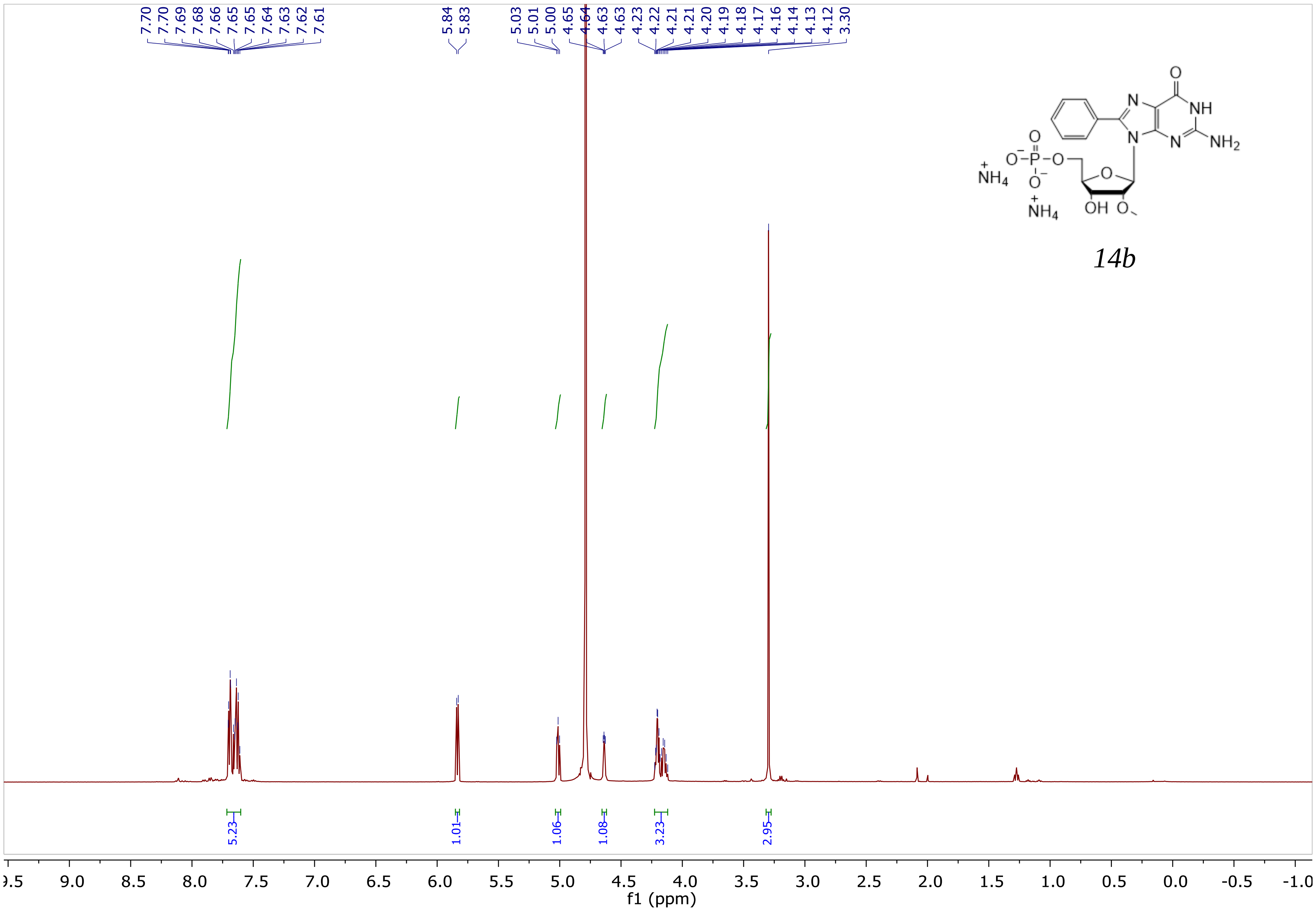

7.70 7.70 7.69 7.68 7.66 7.65 7.65 7.64 7.63 7.62 7.61
5.84 5.83
5.03 5.01 5.00 4.65 4.64 4.63 4.63 4.23 4.22 4.21 4.21 4.20 4.19 4.18 4.17 4.16 4.14 4.13 4.12 3.30
5.23
1.01
1.06
1.08
3.23
2.95
9.5 9.0 8.5 8.0 7.5 7.0 6.5 6.0 5.5 5.0 4.5 4.0 3.5 3.0 2.5 2.0 1.5 1.0 0.5 0.0 -0.5 -1.0
f1 (ppm)

14b

^{1}H-^{1}H COSY NMR

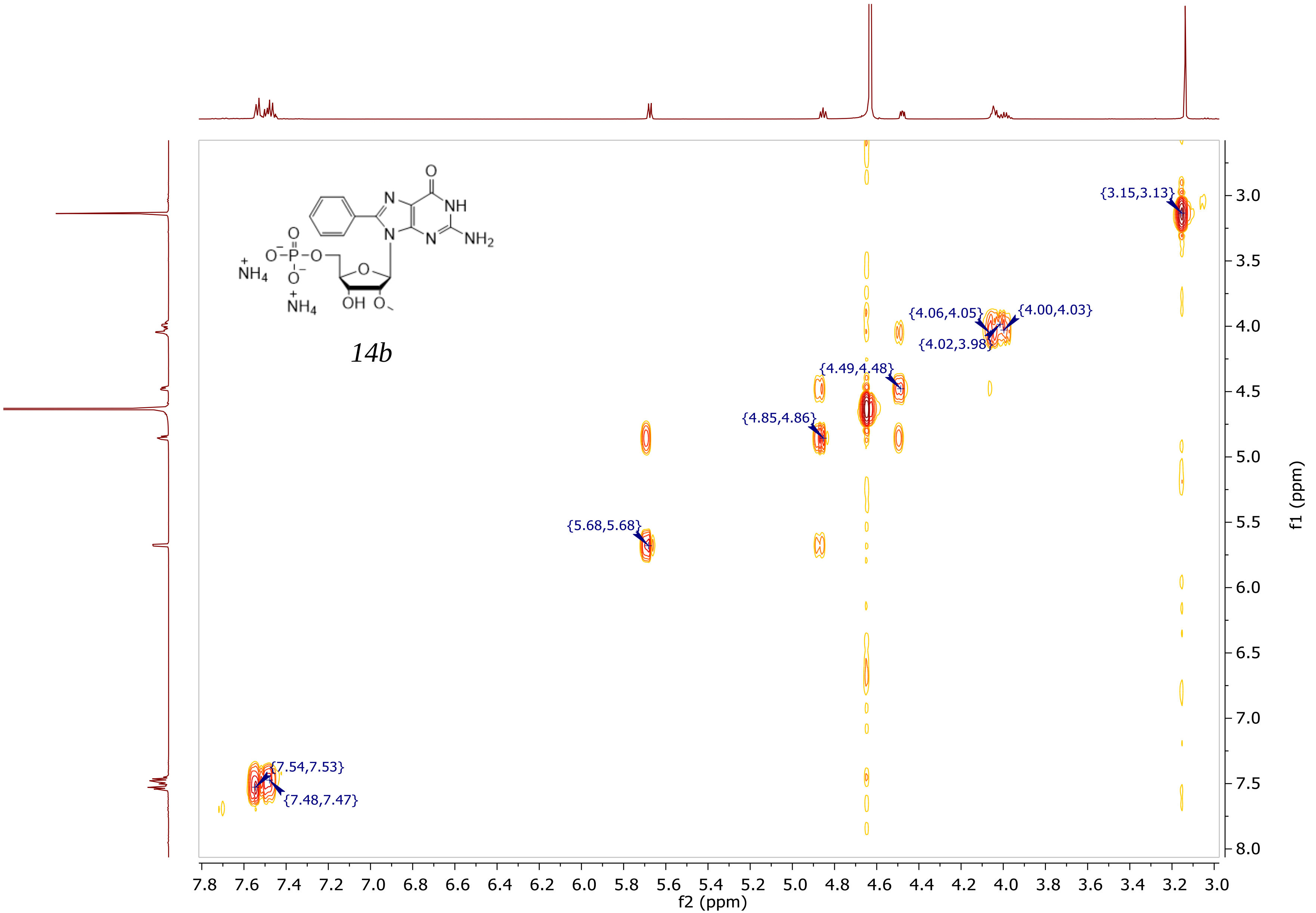

^{31}P NMR

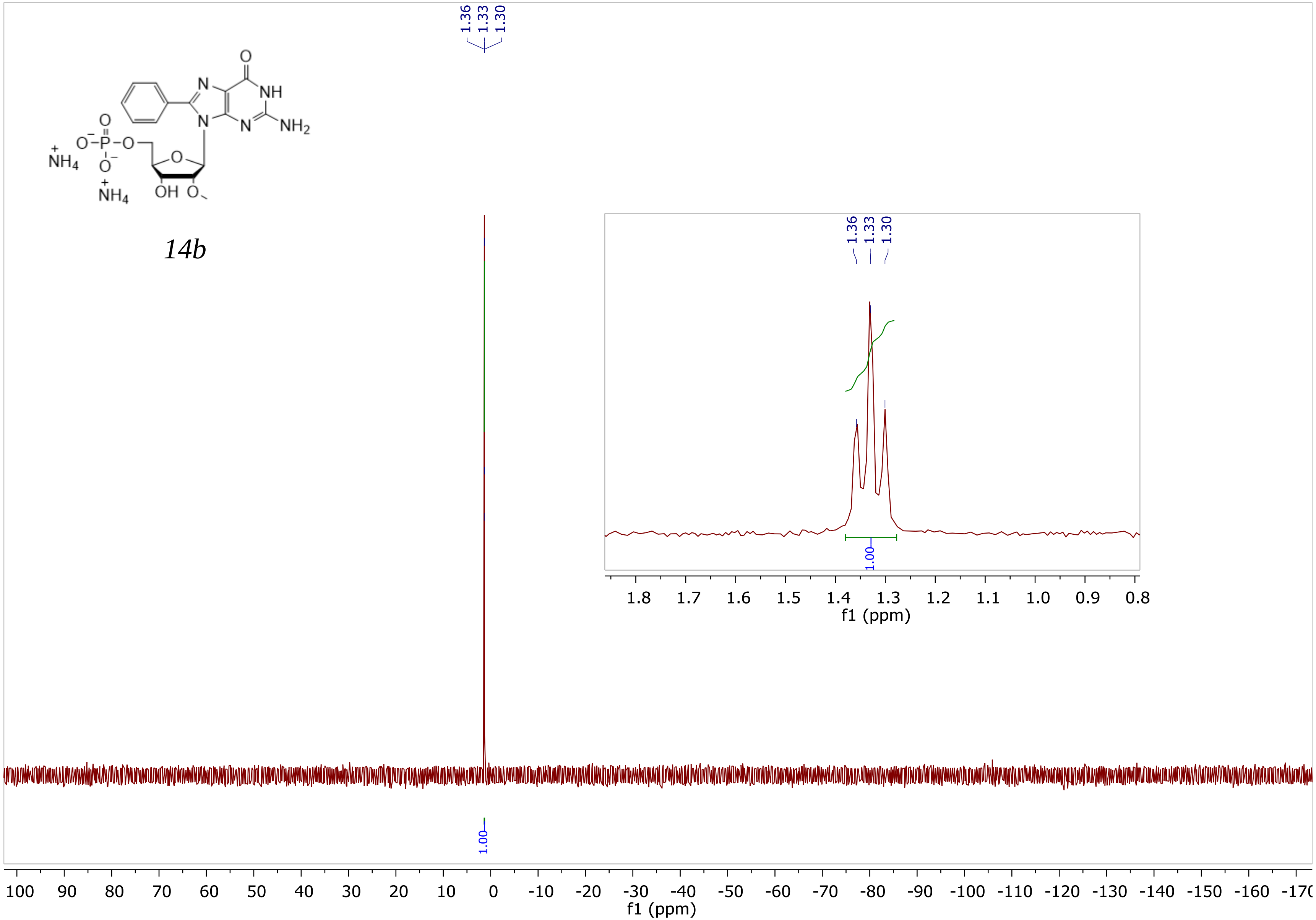

HRMS

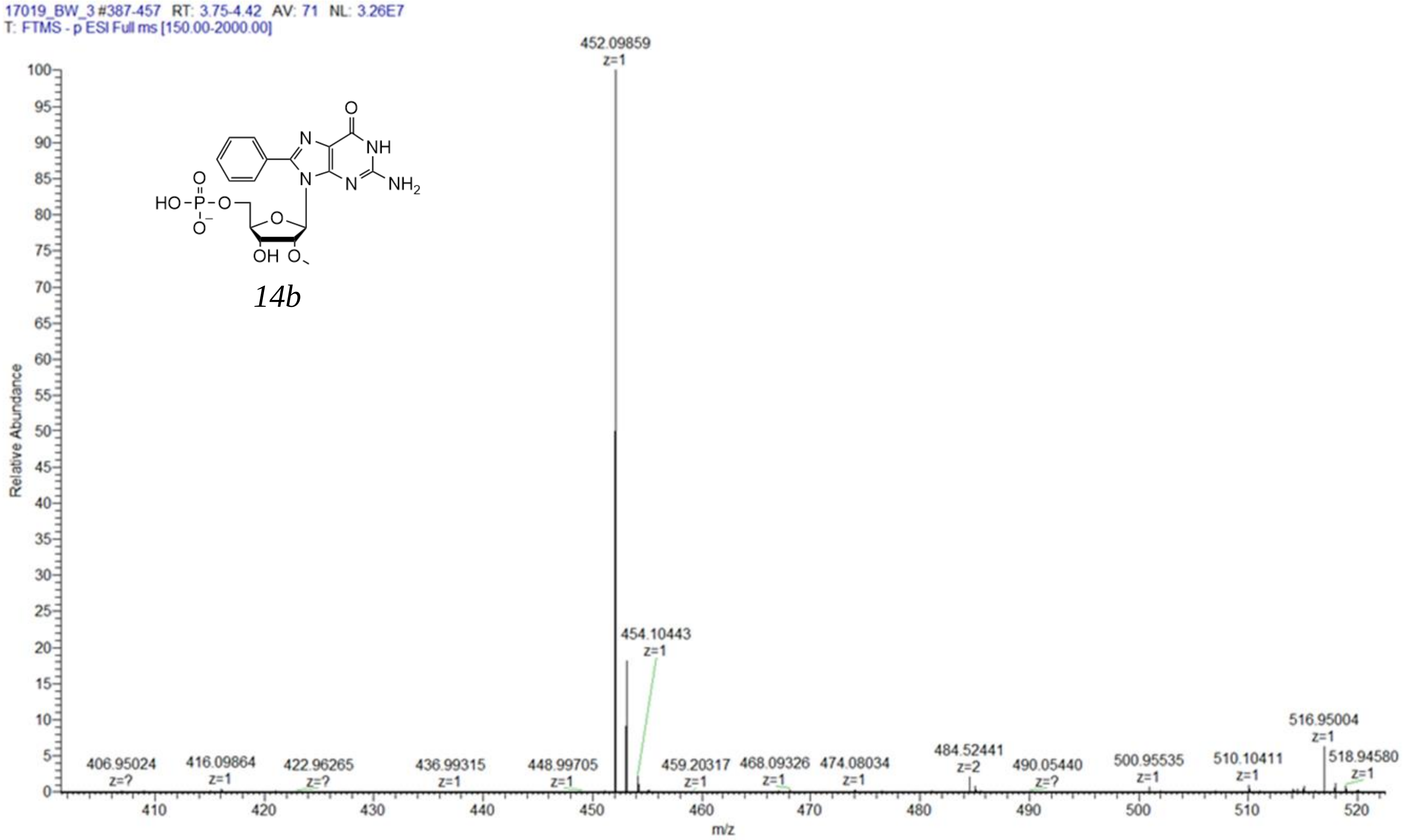

Summary

Rt (D) = 8.97 min; ^{1}H NMR (500 MHz, D_2O) δ 7.66 (m, 5H), 5.83 (d, *J* = 6.4 Hz, 1H), 5.01 (dd, *J* = 6.4 Hz, 5.6 Hz, 1H), 4.64 (dd, *J* = 5.6 Hz, 3.4 Hz, 1H), 4.19 (m, 3H), 3.30 (s, 3H); ^{31}P NMR (202 MHz, D_2O) δ 1.33 (t, *J* = 5.8 Hz, 1P). HRMS ESI (-) m/z [M-H]$^-$, calcd for $C_{17}H_{19}N_5O_8P^-$ 452.0977; found 452.0986.

$^{8\text{-DMAPh}}m^{2'O}GMP$ (14c)

Structure

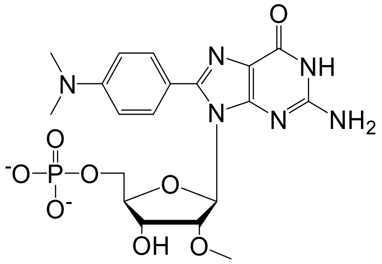

RP-HPLC profile

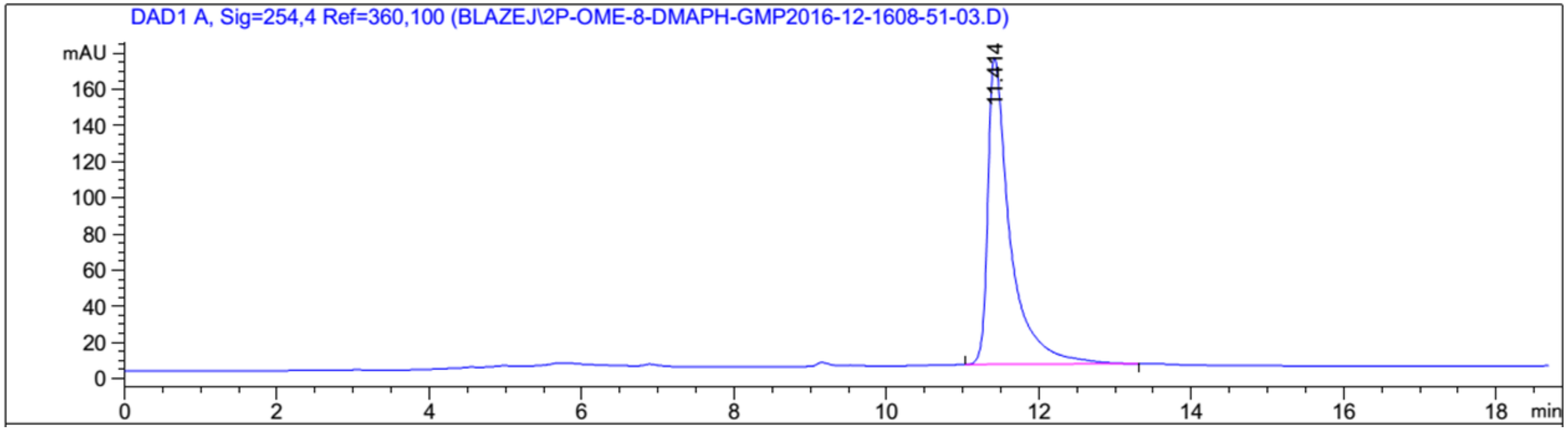

^{1}H NMR

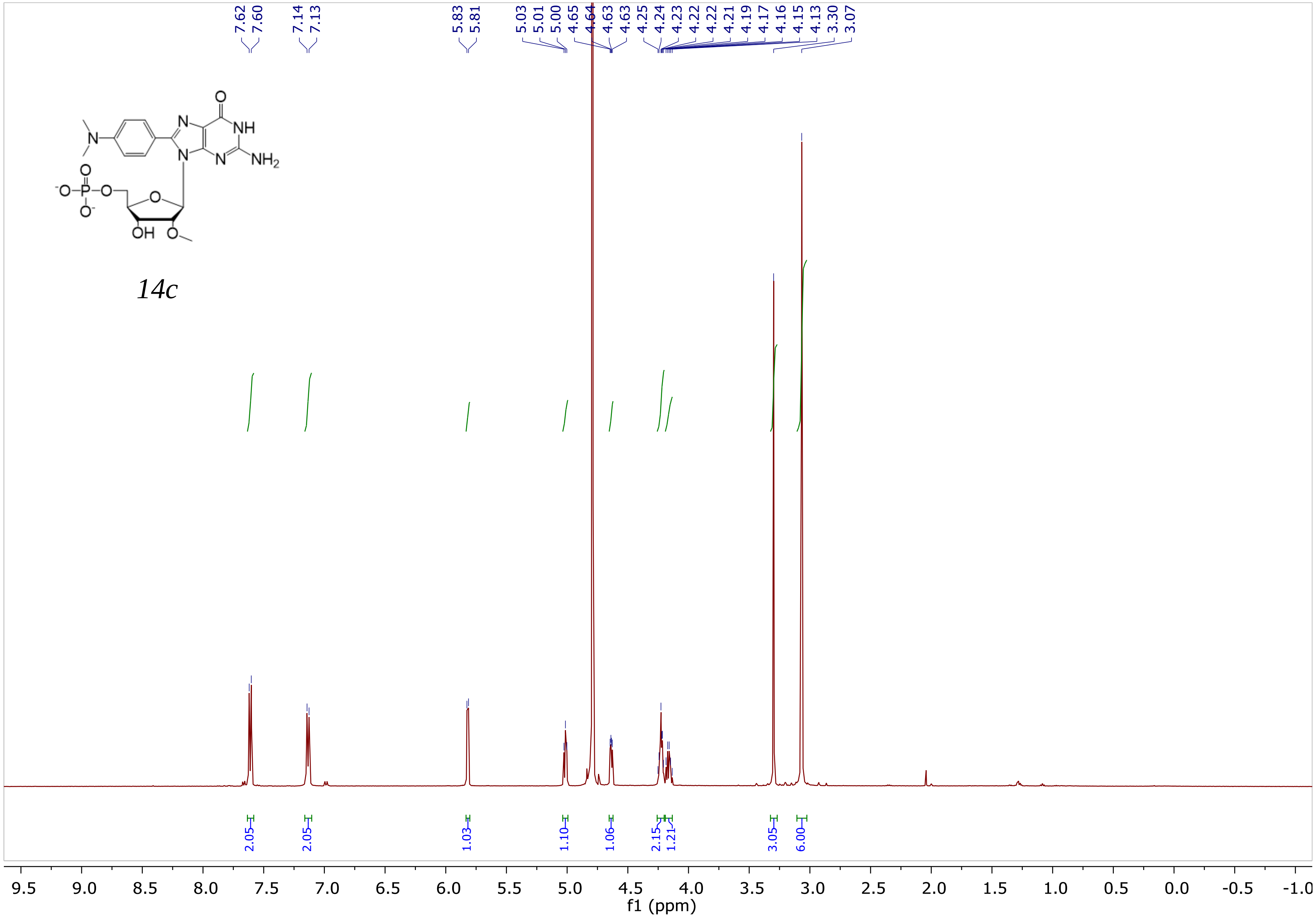
NH
NH2
N
O
OH
7.62
7.60
7.14
7.13
5.83
5.81
5.03
5.01
5.00
4.65
4.64
4.63
4.63
4.25
4.24
4.23
4.22
4.22
4.21
4.19
4.17
4.16
4.15
4.13
3.30
3.07
2.05
2.05
1.03
1.10
1.06
2.15
1.21
3.05
6.00
9.5
9.0
8.5
8.0
7.5
7.0
6.5
6.0
5.5
5.0
4.5
4.0
3.5
3.0
2.5
2.0
1.5
1.0
0.5
0.0
-0.5
-1.0
f1 (ppm)

14c

^1H-^1H COSY NMR

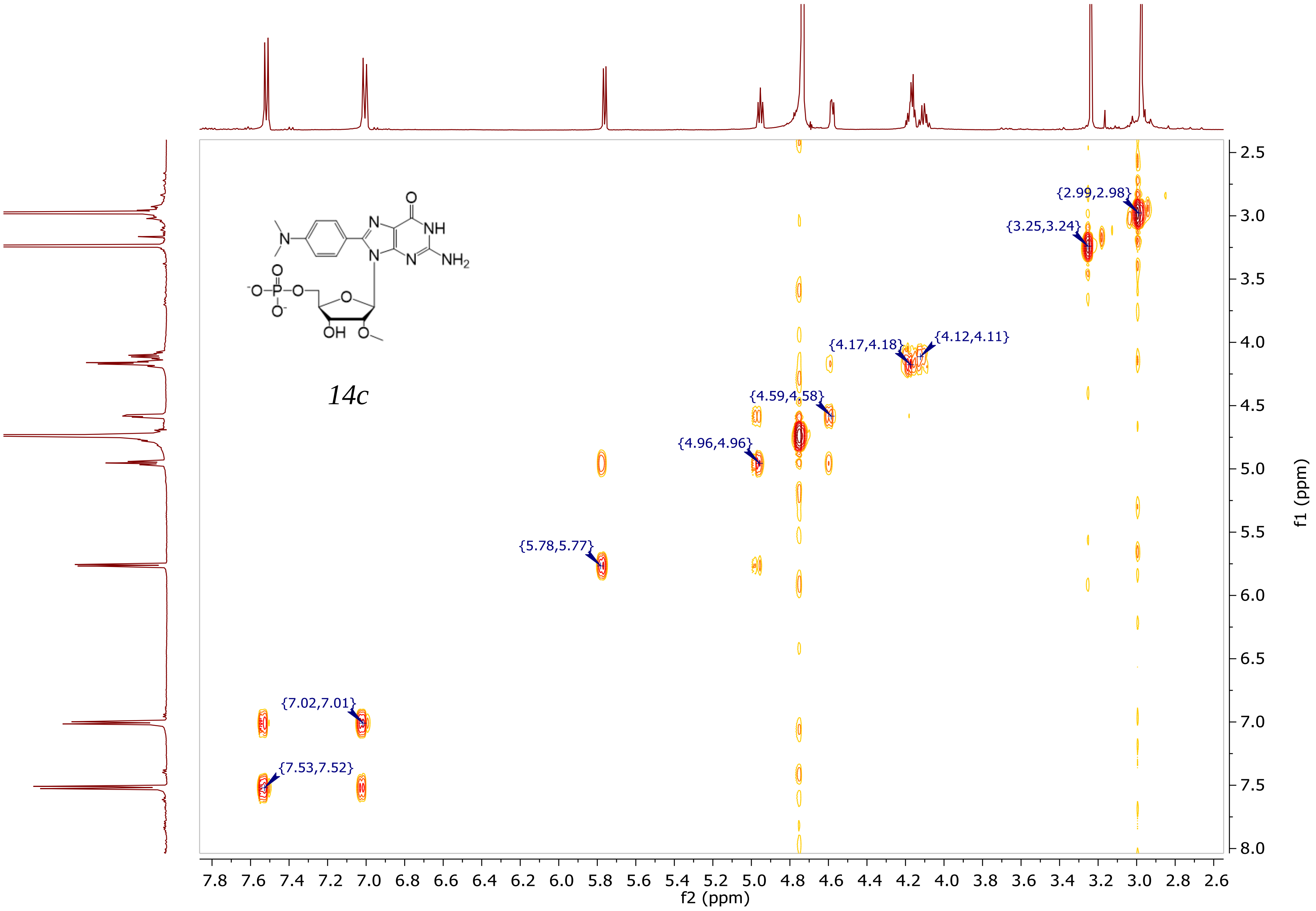

31P NMR

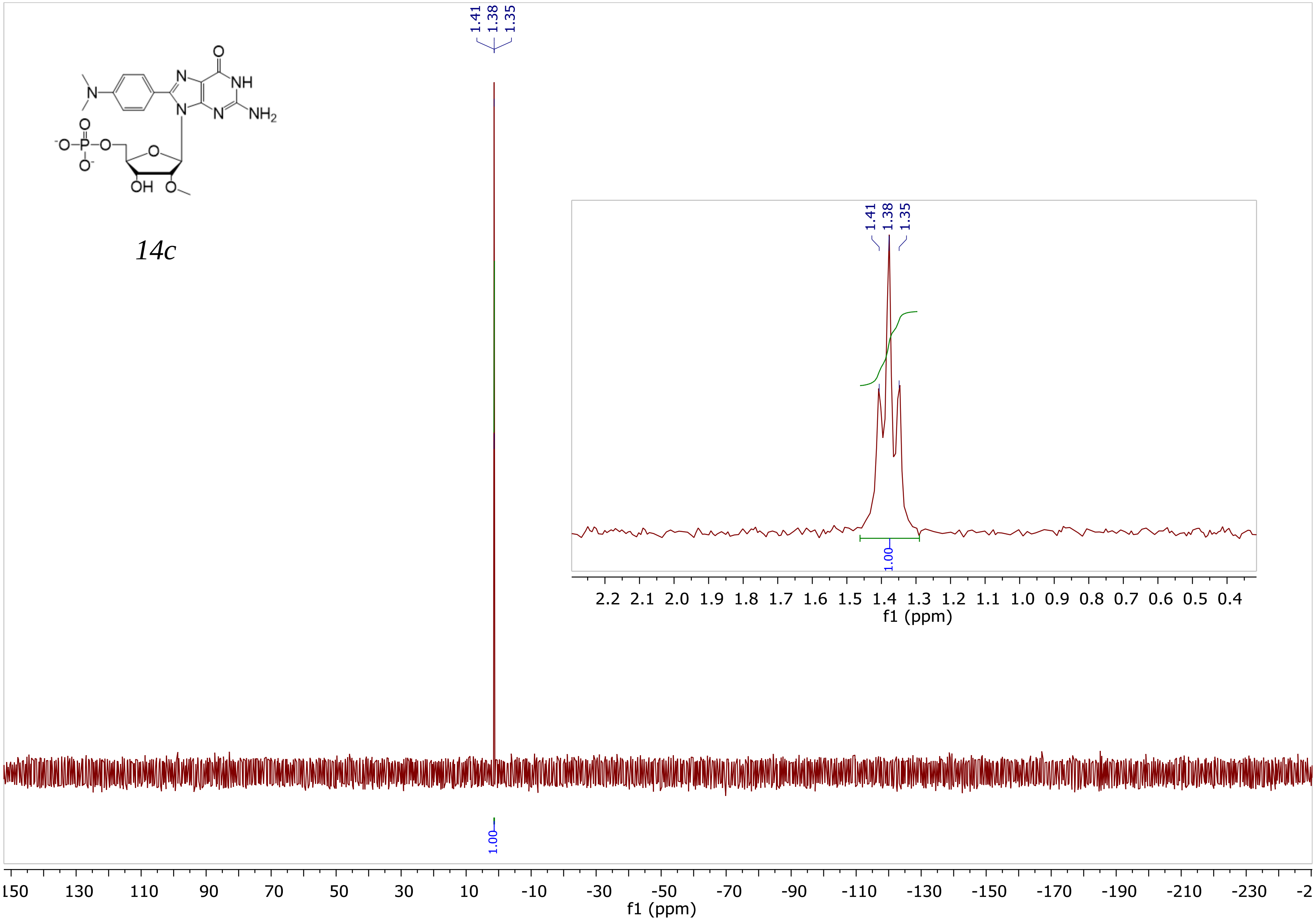

14c

HRMS

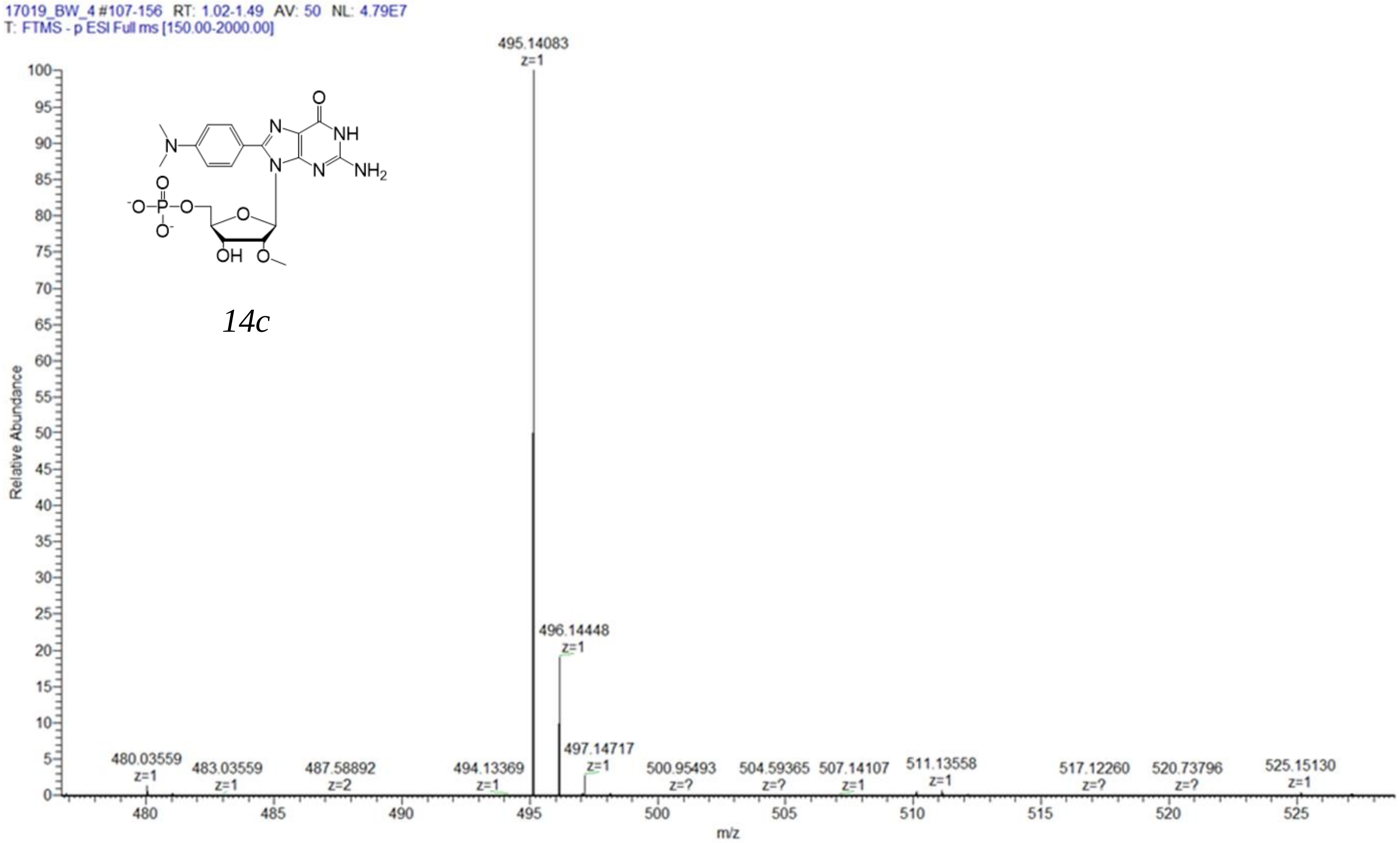

Summary

Rt (D) = 11.41 min; ^{1}H NMR (500 MHz, D_2O) δ 7.61 (d, *J* = 8.5 Hz, 2H), 7.13 (d, *J* = 8.5 Hz, 2H), 5.82 (d, *J* = 6.4 Hz, 1H), 5.01 (dd, *J* = 6.4 Hz, 5.6 Hz, 1H), 4.64 (dd, *J* = 5.6 Hz, 3.3 Hz, 2H), 4.23 (m, 2H), 4.16 (q, *J* = 6.7 Hz, 1H), 3.30 (s, 3H), 3.07 (s, 6H); ^{31}P NMR (202 MHz, D_2O) δ 1.38 (t, *J* = 5.8 Hz, 1P); HRMS ESI (-) m/z [M-H]$^-$, calcd for $C_{19}H_{24}N_6O_8P^-$ 495.1399; found 495.1408.

$^{8PhCN}m^{2'O}$GMP (14d)

Structure

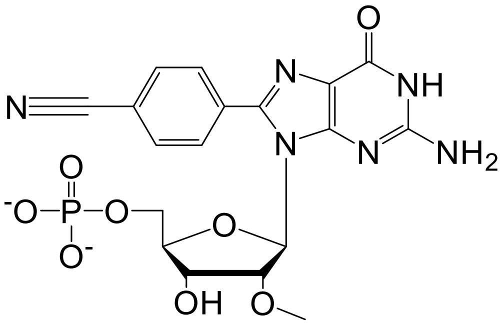

RP-HPLC profile

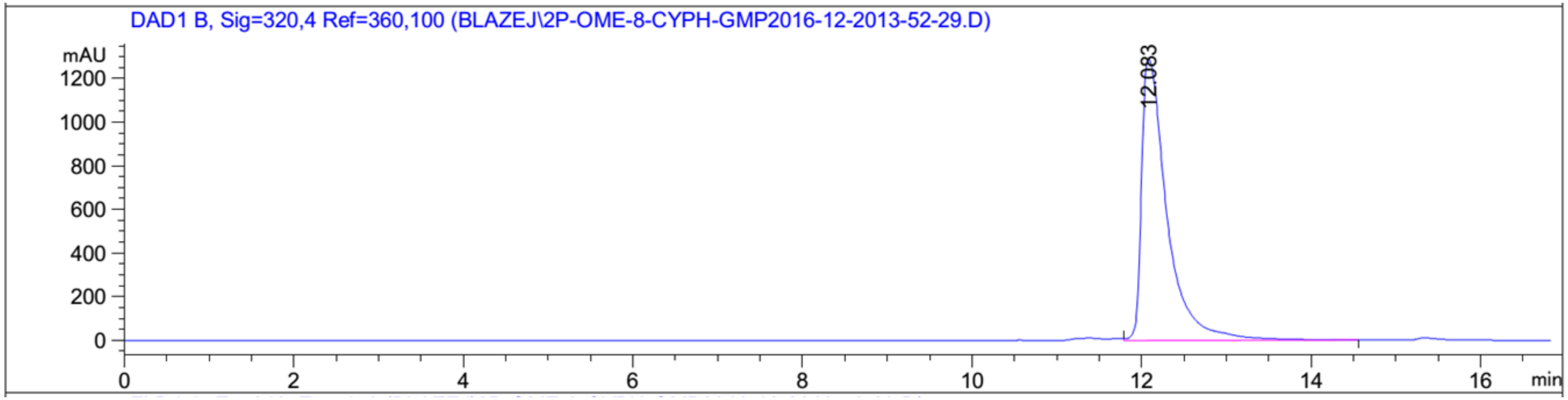

^{1}H NMR

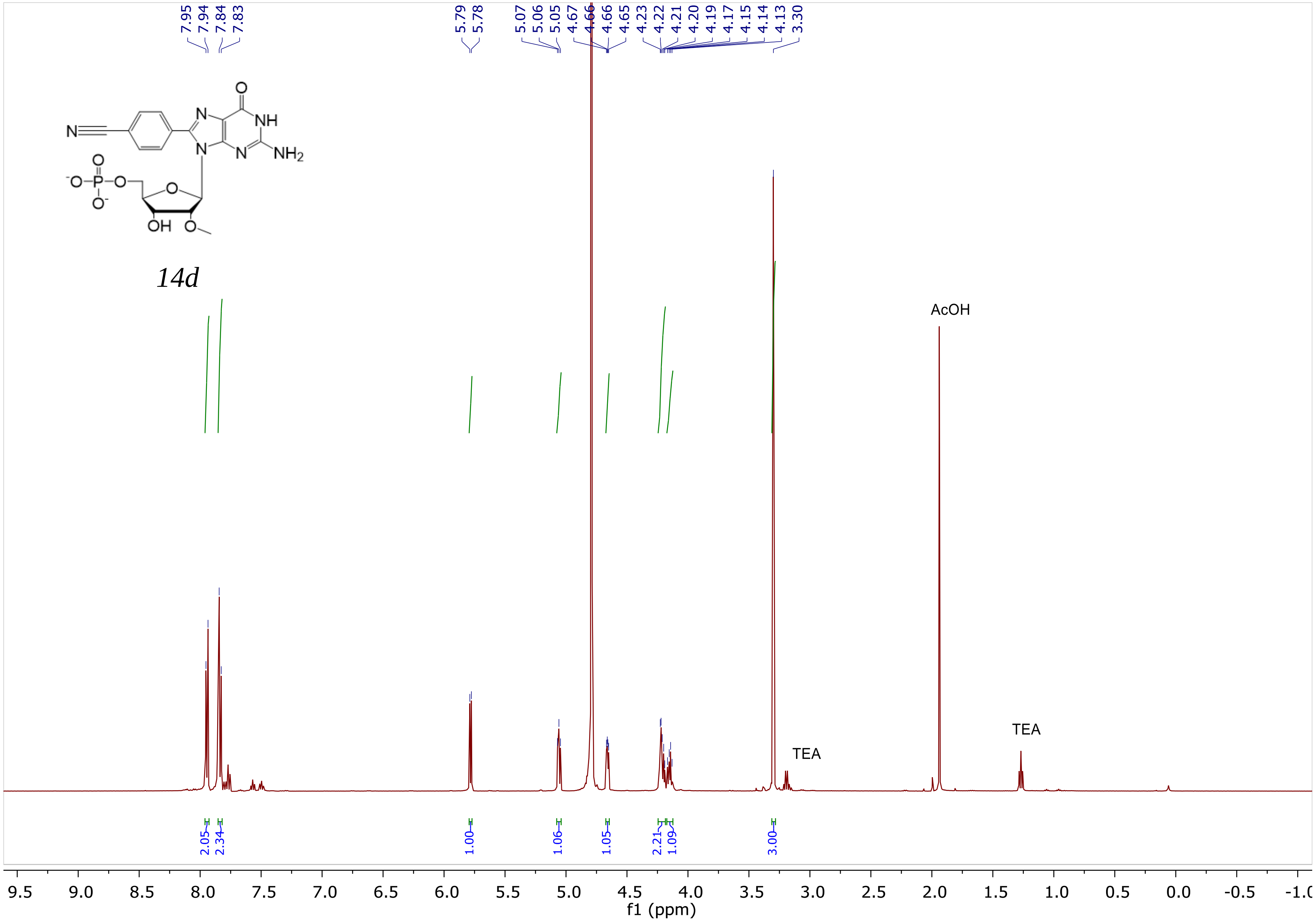

^{1}H-^{1}H COSY NMR

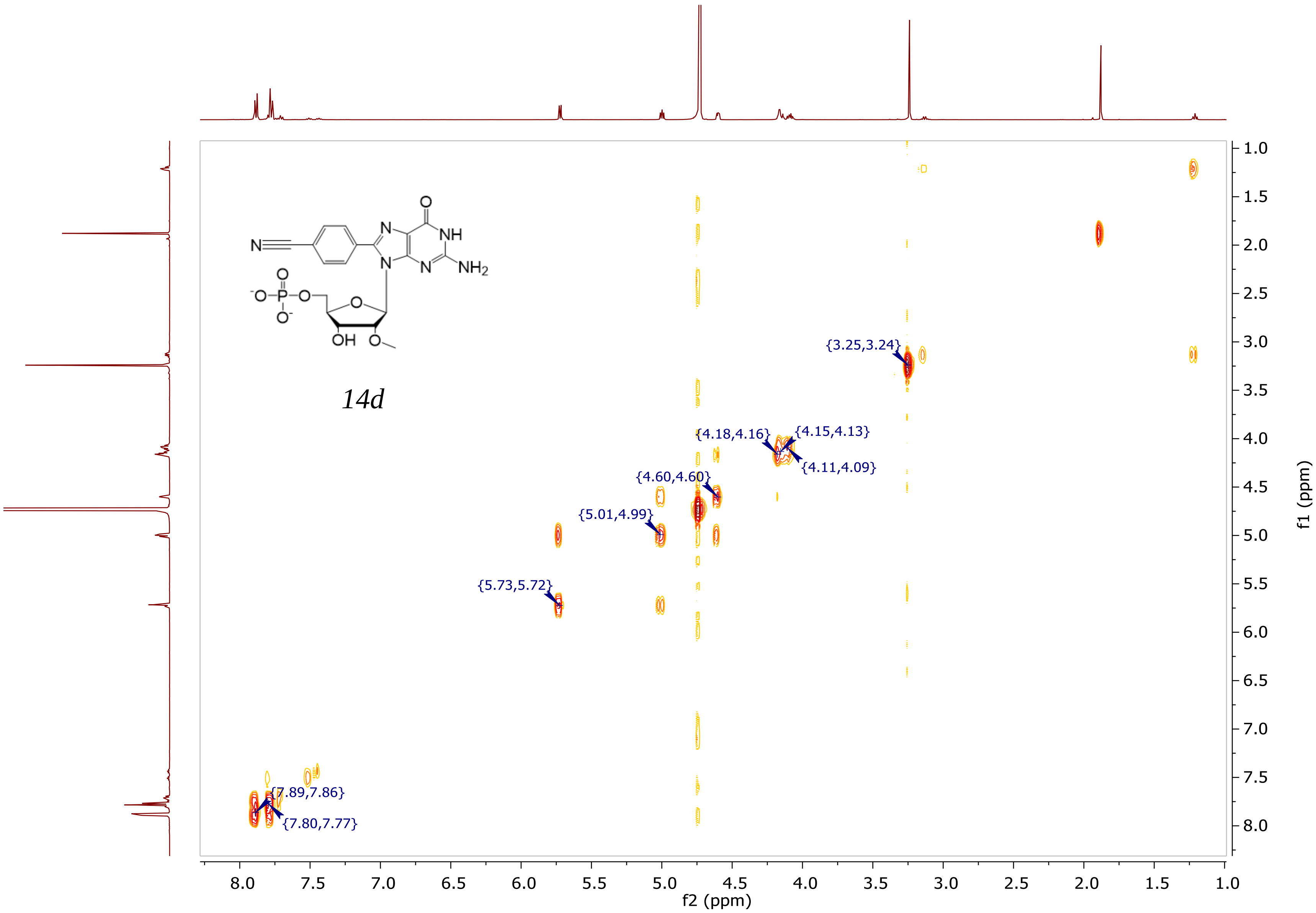
14d
{3.25,3.24}
{4.18,4.16}
{4.15,4.13}
{4.11,4.09}
{4.60,4.60}
{5.01,4.99}
{5.73,5.72}
{7.89,7.86}
{7.80,7.77}
f2 (ppm)
f1 (ppm)

^{31}P NMR

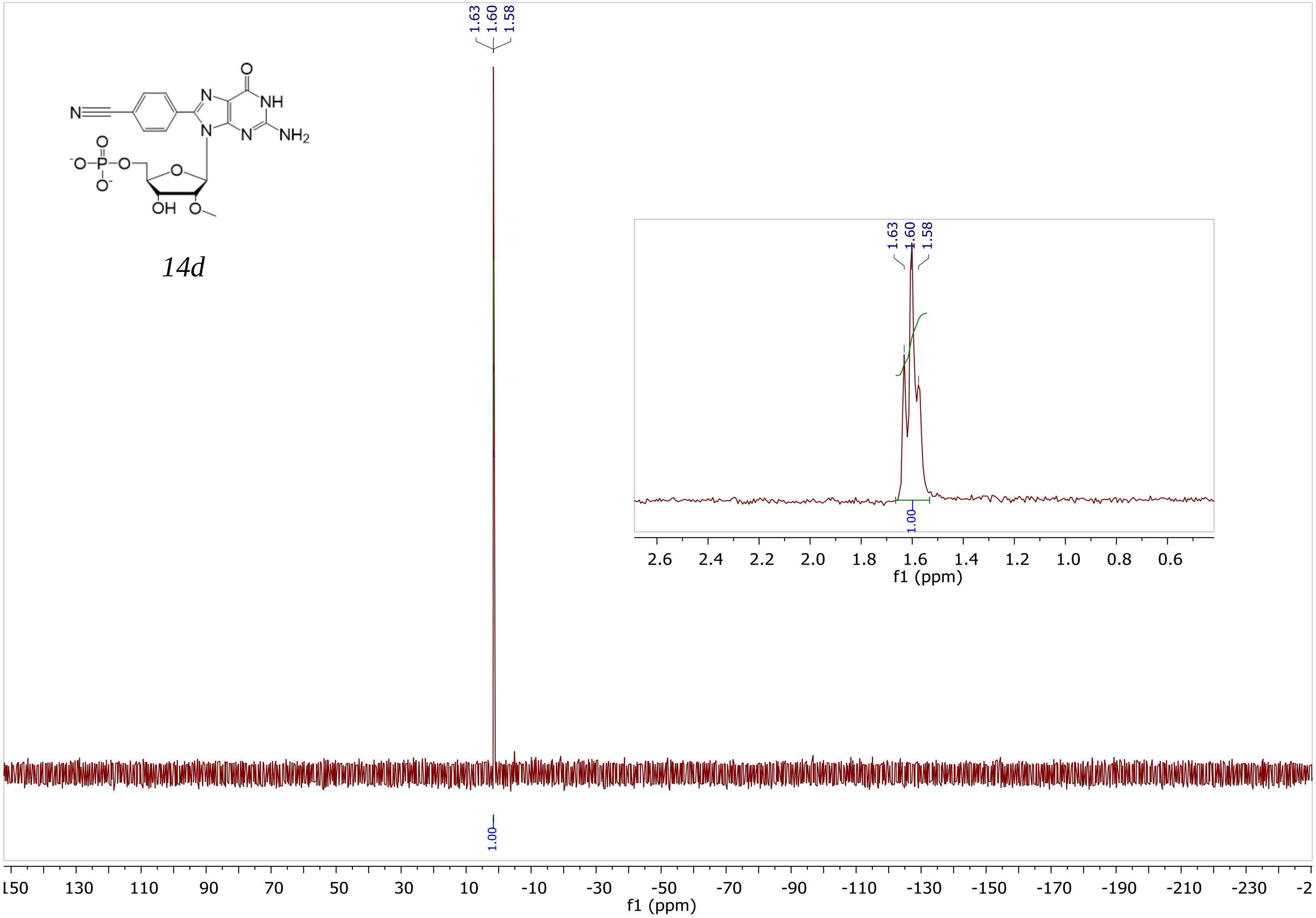

1.63
1.60
1.58
N
NH
NH2
O
P
OH
1.00
2.6
2.4
2.2
2.0
1.8
1.6
1.4
1.2
1.0
0.8
0.6
f1 (ppm)
130
110
90
70
50
30
10
-10
-30
-50
-70
-90
-110
-130
-150
-170
-190
-210
-230

14d

HRMS

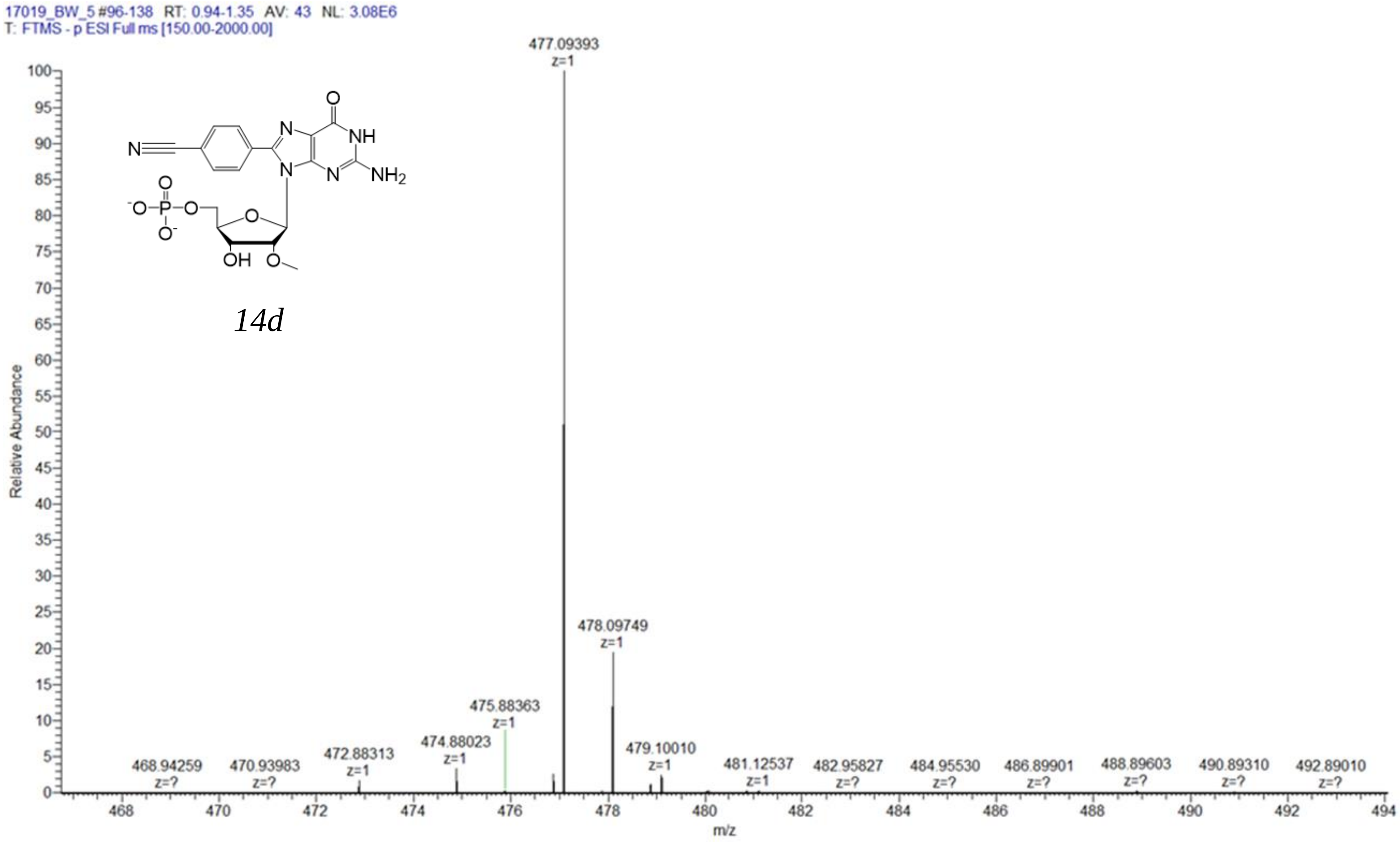

14d

Summary

Rt (D) = 12.08 min; ^{1}H NMR (500 MHz, D_2O) δ 7.94 (d, *J* = 8.4 Hz, 2H), 7.84 (d, *J* = 8.4 Hz, 2H), 5.78 (d, *J* = 6.5 Hz, 1H), 5.06 (t, *J* = 6.5 Hz, 5.5 Hz, 1H), 4.66 (dd, *J* = 5.5 Hz, 3.3 Hz, 2H), 4.21 (m, 2H), 4.15 (m, 1H), 3.30 (s, 3H); ^{31}P NMR (202 MHz, D_2O) δ 1.60 (t, *J* = 5.7 Hz, 1P); HRMS ESI (-) m/z [M-H]$^-$, calcd for $C_{18}H_{18}N_6O_8P^-$ 477.0929; found 477.0939.

$^{8Me}m^{2'O}$GMP (14e)

Structure

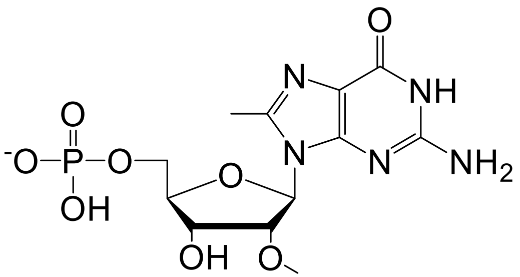

RP-HPLC profile

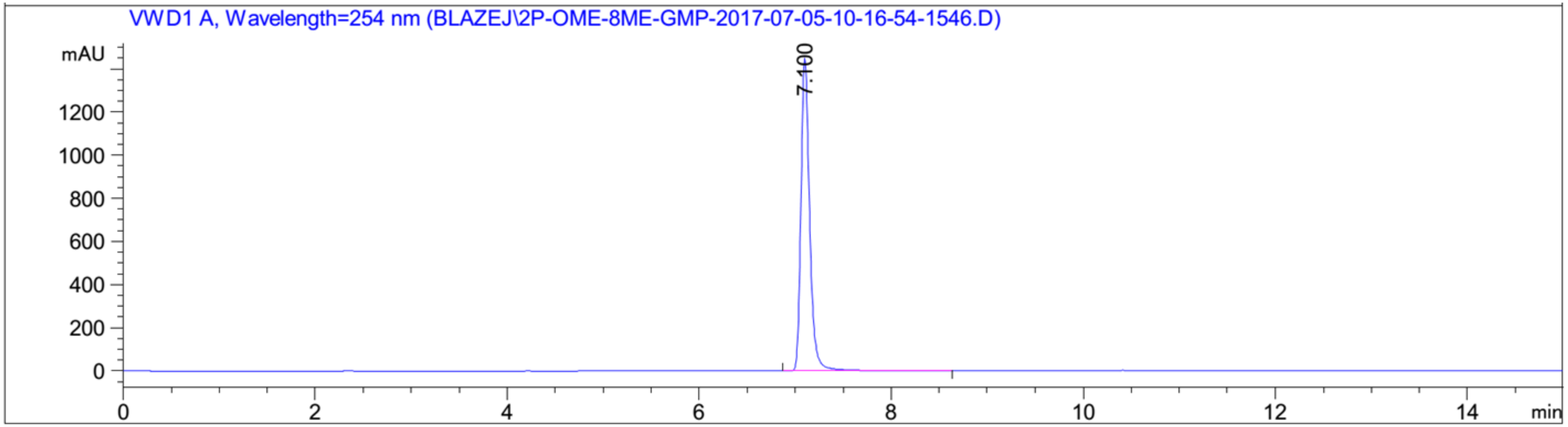

^{1}H NMR

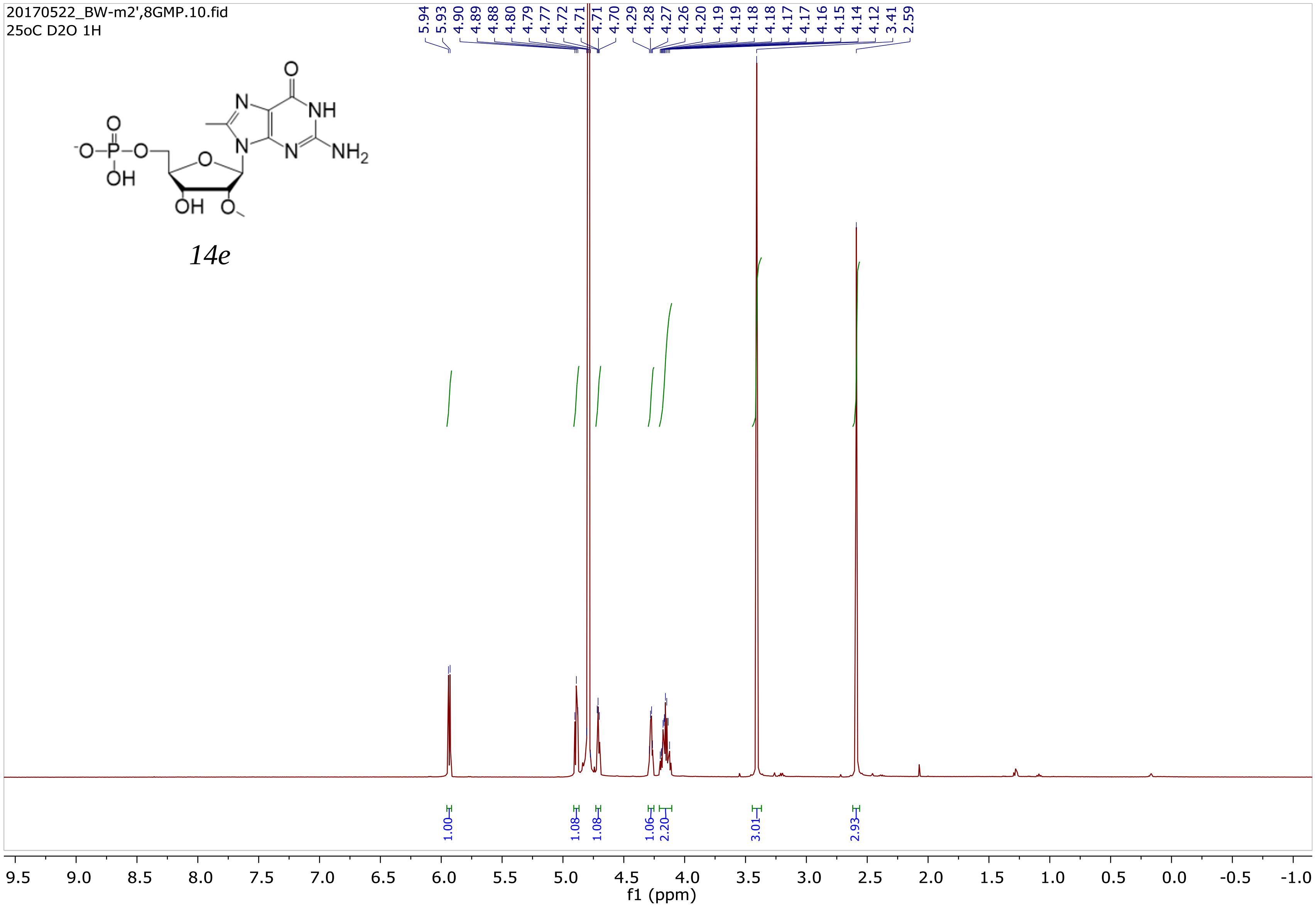

20170522_BW-m2',8GMP.10.fid
25oC D2O 1H
14e
5.94 5.93 4.90 4.89 4.88 4.80 4.79 4.77 4.72 4.71 4.71 4.70 4.29 4.28 4.27 4.26 4.20 4.19 4.19 4.18 4.18 4.17 4.17 4.16 4.15 4.14 4.12 3.41 2.59
1.00 1.08 1.08 1.06 2.20 3.01 2.93
9.5 9.0 8.5 8.0 7.5 7.0 6.5 6.0 5.5 5.0 4.5 4.0 3.5 3.0 2.5 2.0 1.5 1.0 0.5 0.0 -0.5 -1.0
f1 (ppm)

^{31}P NMR

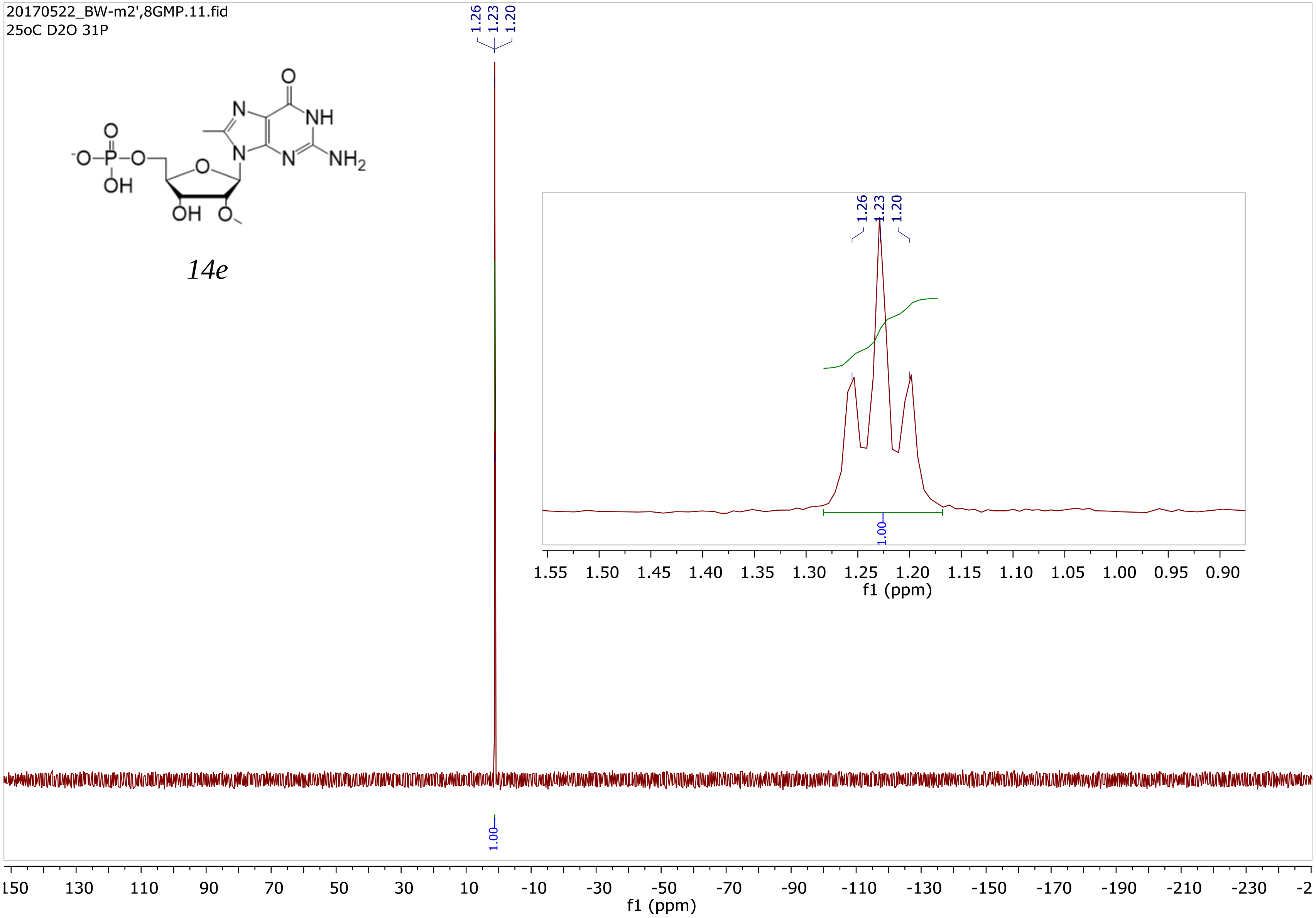
20170522_BW-m2',8GMP.11.fid
25oC D2O 31P
1.26
1.23
1.20
1.00
1.55
1.50
1.45
1.40
1.35
1.30
1.25
1.20
1.15
1.10
1.05
1.00
0.95
0.90
f1 (ppm)
130
110
90
70
50
30
10
-10
-30
-50
-70
-90
-110
-130
-150
-170
-190
-210
-230
f1 (ppm)

14e

HRMS

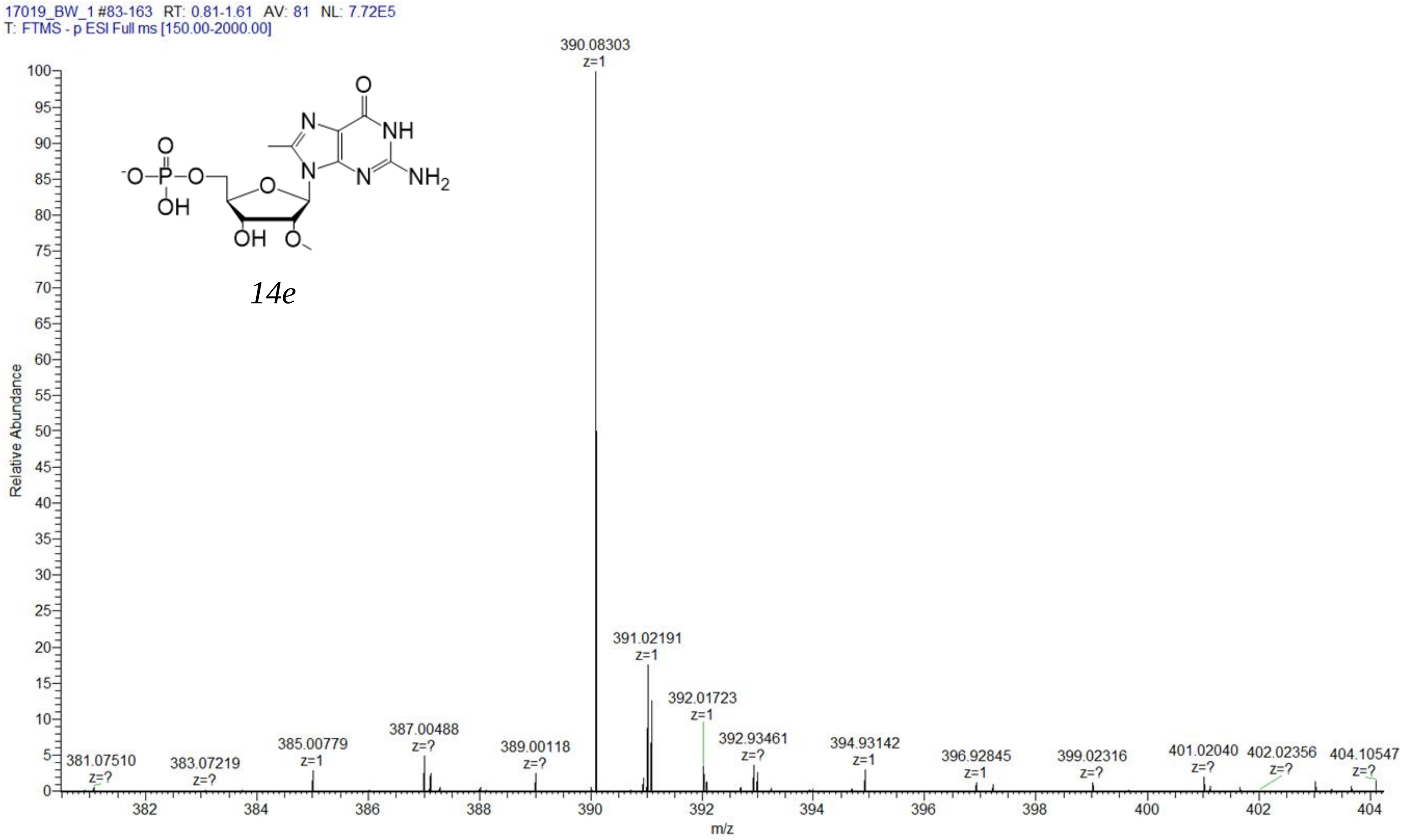

Summary

Rt (A) = 7.10 min; ^{1}H NMR (500 MHz, D_2O) δ 5.93 (d, *J* = 6.5 Hz, 1H), 4.89 (t, *J* = 6.5 Hz, 5.6 Hz, 1H), 4.71 (dd, *J* = 5.6 Hz, 3.6 Hz, 1H), 4.28 (q, *J* = 4.2 Hz, 1H), 4.16 (m, 2H), 3.41 (s, 3H), 2.59 (s, 3H); ^{31}P NMR (202 MHz, D_2O) δ 1.23 (t, *J* = 5.6 Hz, 1P); HRMS ESI (-) m/z [M-H]$^-$, calcd for $C_{12}H_{17}N_5O_8P^-$ 390.0820; found 390.0830.

$^{8\text{-}cPr}m^{2'O}$GMP (14f)

Structure

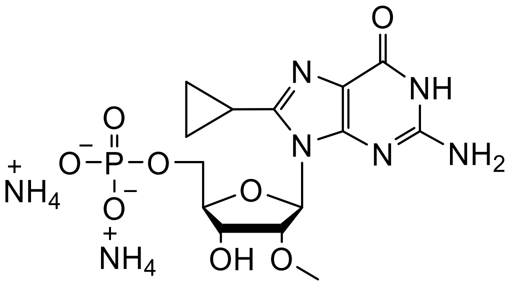

RP-HPLC profile

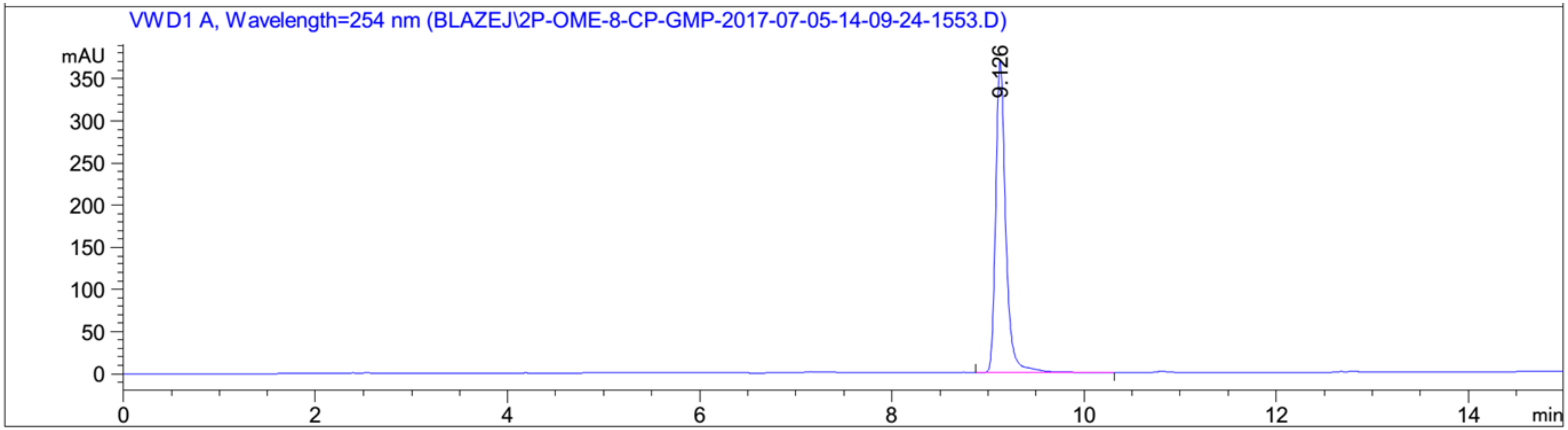

^{1}H NMR

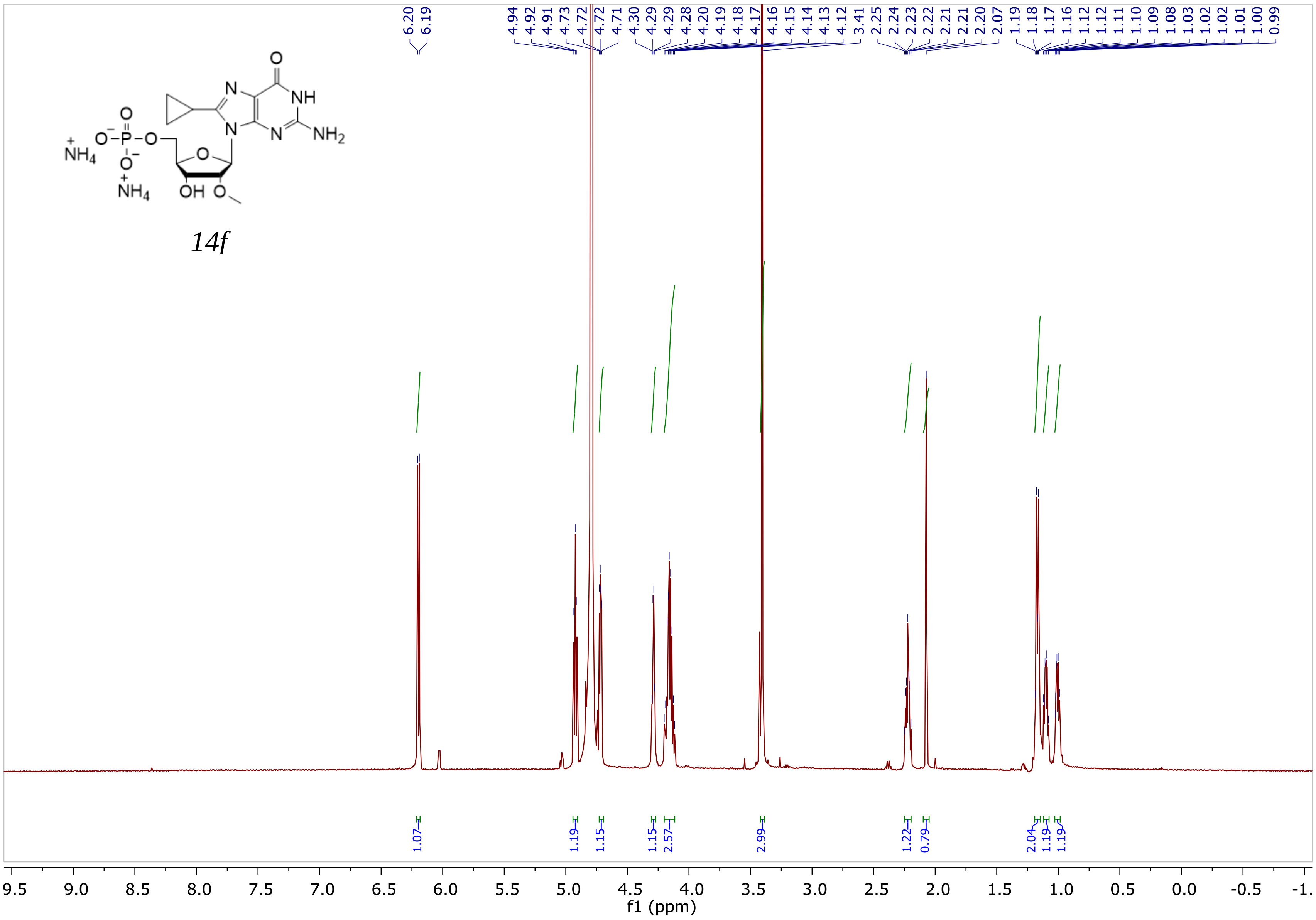

6.20
6.19
4.94
4.92
4.91
4.73
4.72
4.72
4.71
4.30
4.29
4.29
4.28
4.20
4.19
4.18
4.17
4.16
4.15
4.14
4.13
4.12
3.41
2.25
2.24
2.23
2.22
2.21
2.21
2.20
2.07
1.19
1.18
1.17
1.16
1.12
1.12
1.11
1.10
1.09
1.08
1.03
1.02
1.02
1.01
1.00
0.99
1.07
1.19
1.15
1.15
2.57
2.99
1.22
0.79
2.04
1.19
1.19
f1 (ppm)

14f

^{31}P NMR

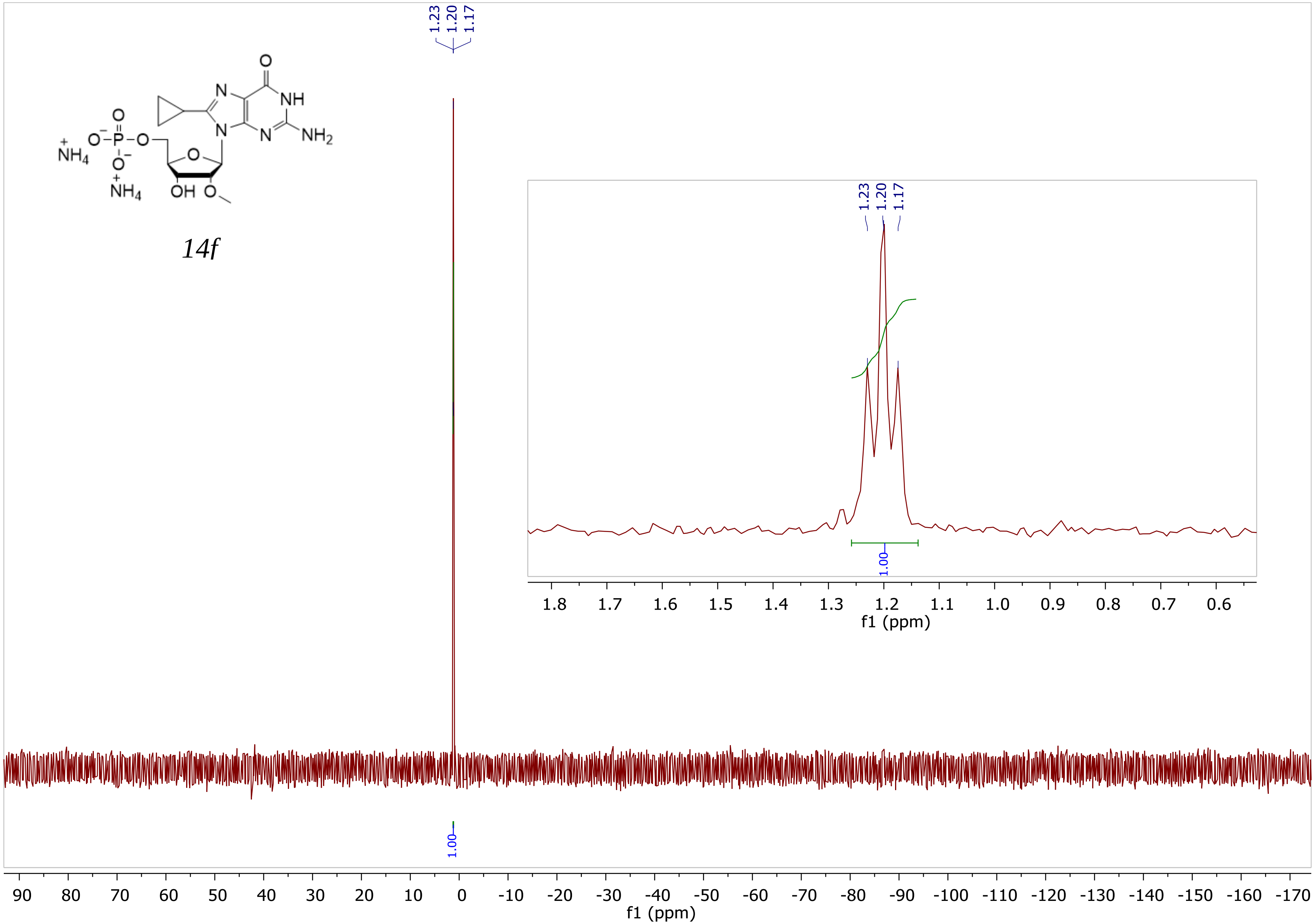

14f
1.23
1.20
1.17
1.00
1.8
1.7
1.6
1.5
1.4
1.3
1.2
1.1
1.0
0.9
0.8
0.7
0.6
f1 (ppm)
90
80
70
60
50
40
30
20
10
0
-10
-20
-30
-40
-50
-60
-70
-80
-90
-100
-110
-120
-130
-140
-150
-160
-170

HRMS

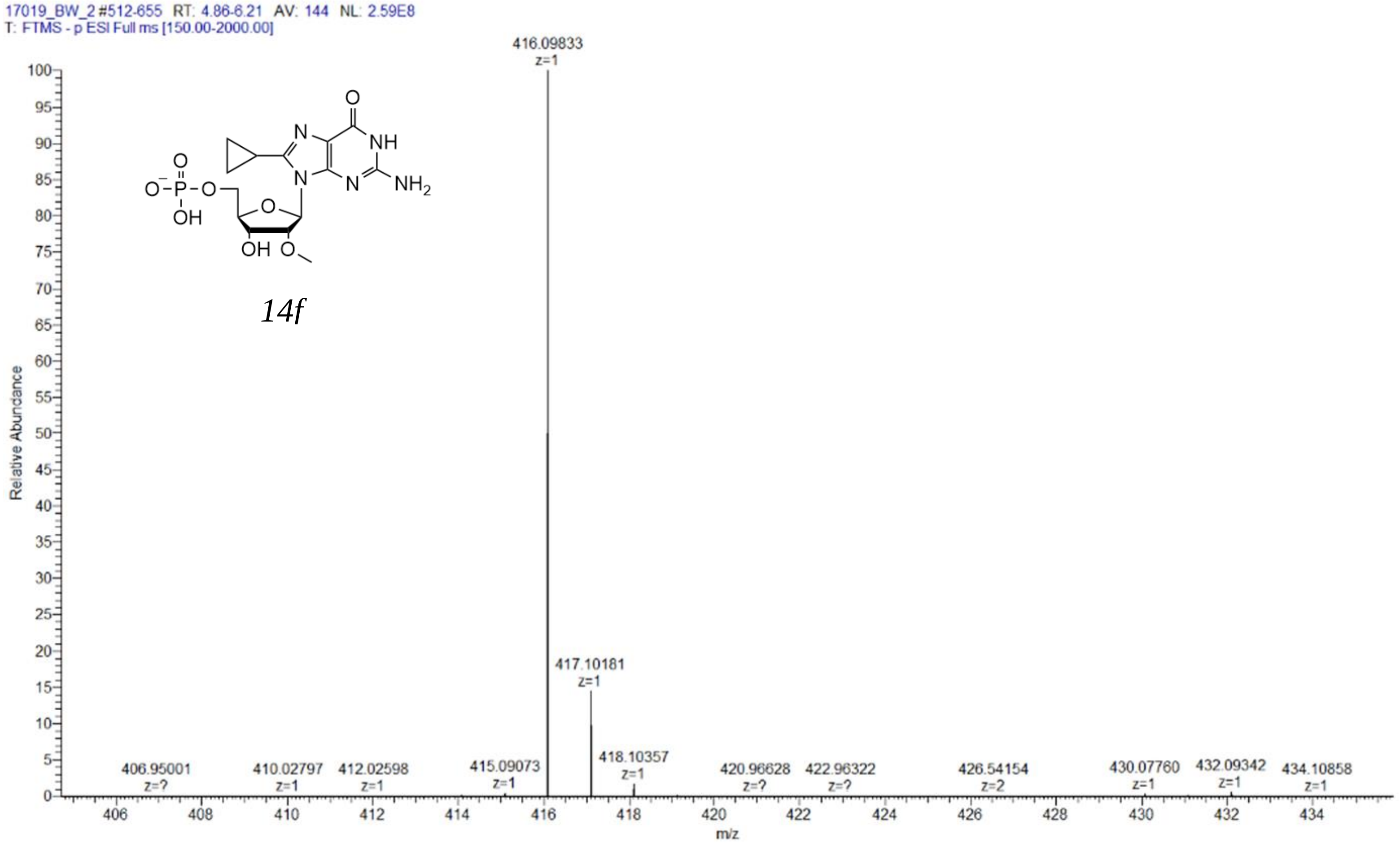

14f

Summary

Rt (A) = 9.13 min; ^{1}H NMR (500 MHz, D_2O) δ 6.20 (d, *J* = 6.7 Hz, 1H), 4.92 (m, 1H), 4.72 (dd, *J* = 5.6 Hz, 3.6 Hz, 1H), 4.29 (q, *J* = 3.8 Hz, 1H), 4.16 (m, 3H), 3.41 (s, 3H), 2.22 (td, *J* = 8.3 Hz, 4.2 Hz, 1H), 1.17 (dd, *J* = 8.3 Hz, 4.8 Hz, 2H), 1.10 (m, 1H), 1.01 (m, 1H); ^{31}P NMR (202 MHz, D_2O) δ 1.20 (t, *J* = 5.6 Hz, 1P); HRMS ESI (-) m/z [M-H]$^-$, calcd for $C_{14}H_{19}N_5O_8P^-$ 416.0977; found 416.0983.

$^{8\text{-}Ph}m^{2'O,7}GMP$ (15b)

Structure

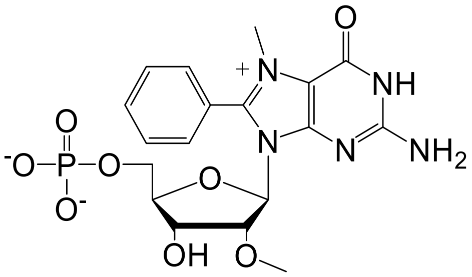

RP-HPLC profile

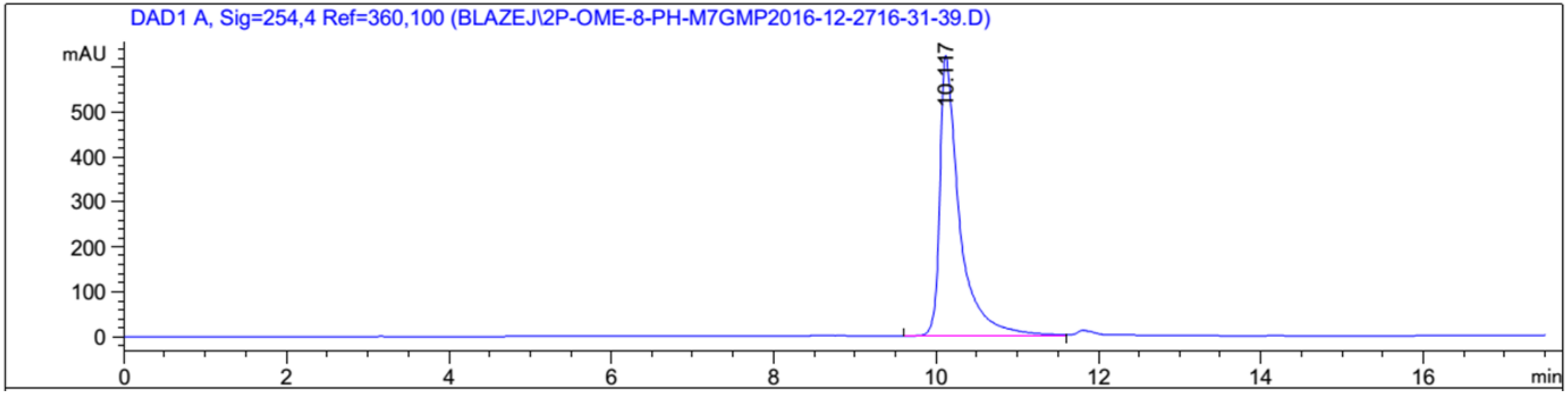

^{1}H NMR

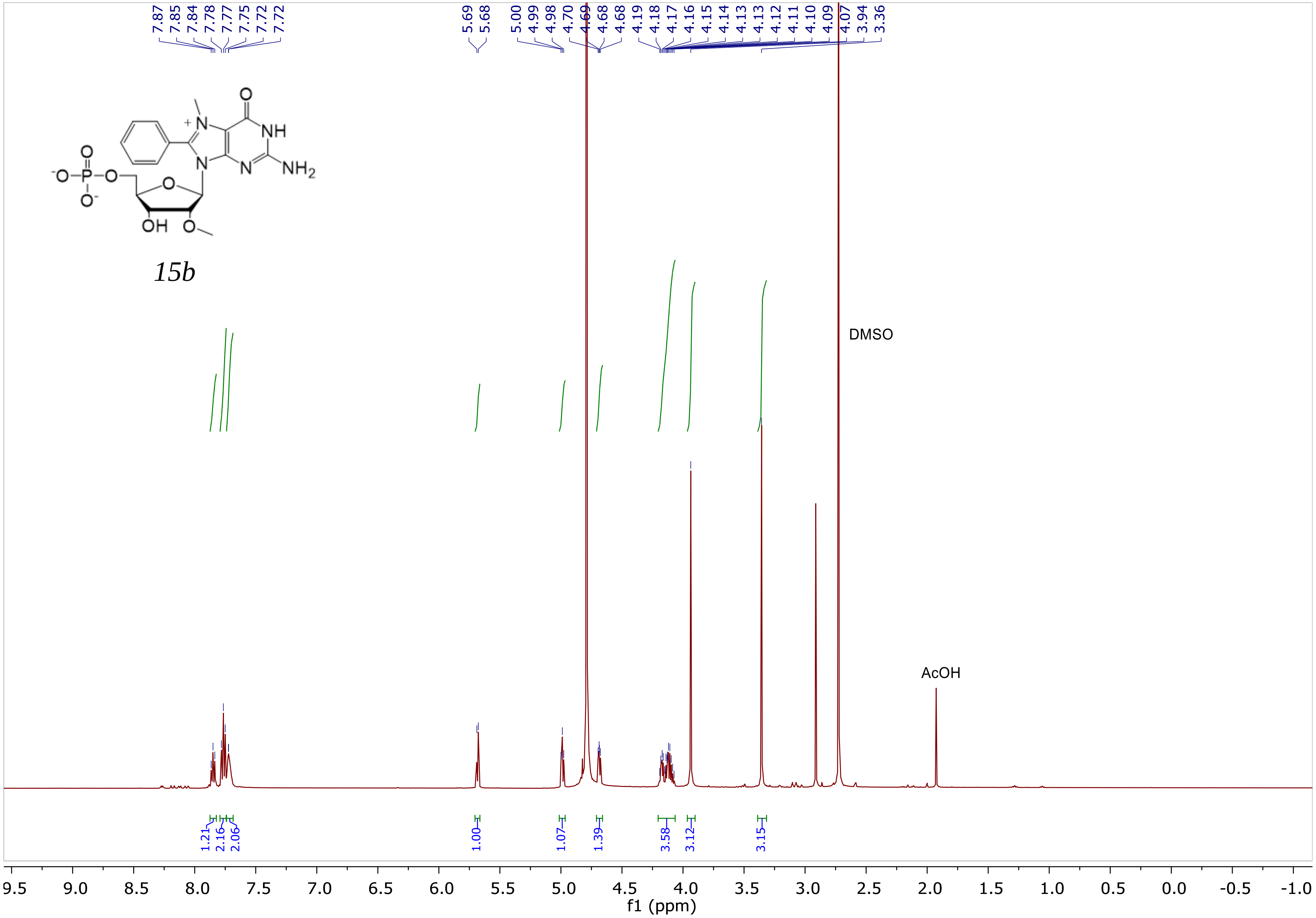
7.87 7.85 7.84 7.78 7.77 7.75 7.72 7.72
5.69 5.68
5.00 4.99 4.98 4.70 4.69 4.68 4.68 4.19 4.18 4.17 4.16 4.15 4.14 4.13 4.13 4.12 4.11 4.10 4.09 4.07 3.94 3.36
15b
DMSO
AcOH
1.21 2.16 2.06 1.00 1.07 1.39 3.58 3.12 3.15
9.5 9.0 8.5 8.0 7.5 7.0 6.5 6.0 5.5 5.0 4.5 4.0 3.5 3.0 2.5 2.0 1.5 1.0 0.5 0.0 -0.5 -1.0
f1 (ppm)

^{31}P NMR

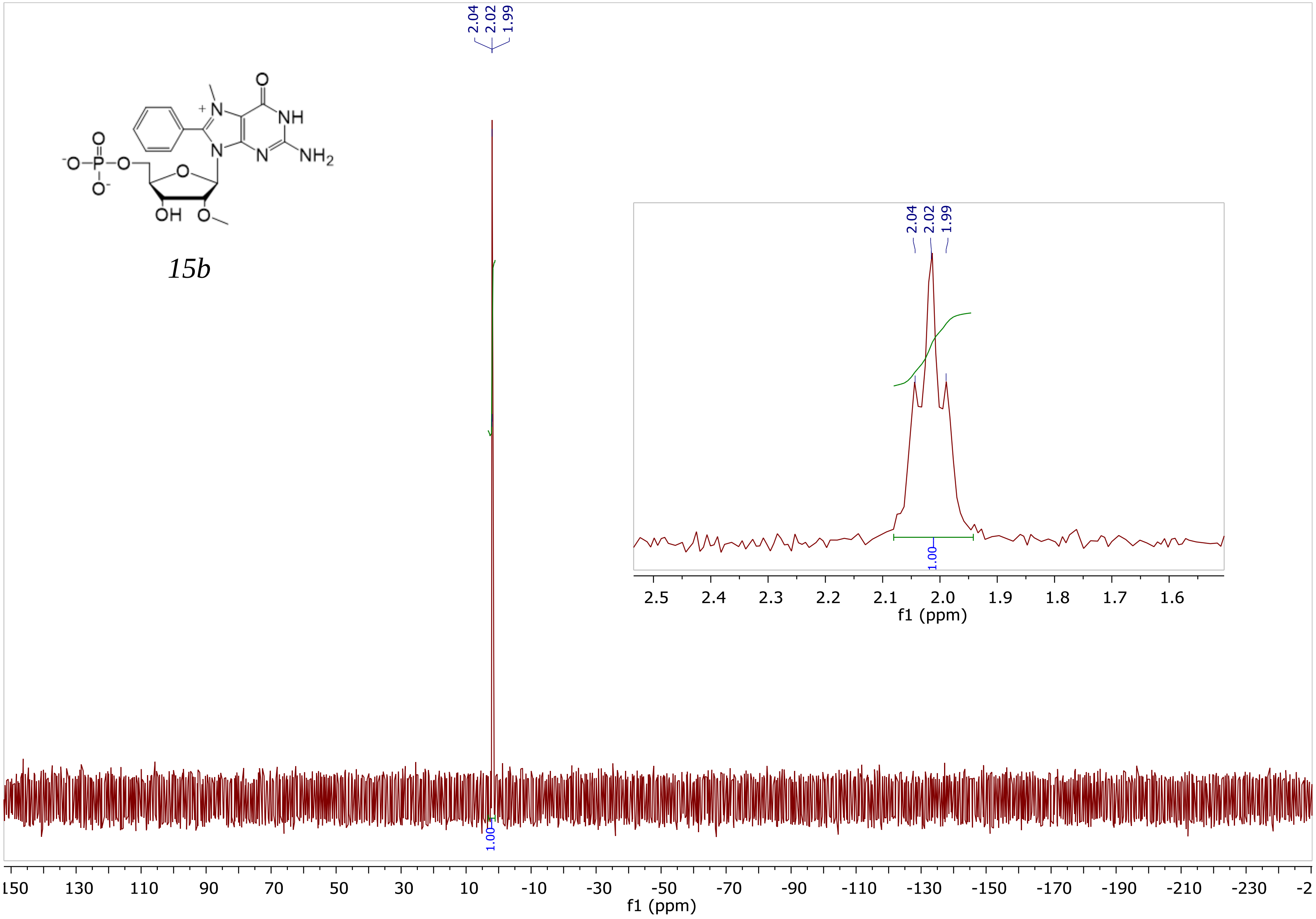
15b
2.04
2.02
1.99
1.00
2.5
2.4
2.3
2.2
2.1
2.0
1.9
1.8
1.7
1.6
f1 (ppm)
150
130
110
90
70
50
30
10
-10
-30
-50
-70
-90
-110
-130
-150
-170
-190
-210
-230
-2
f1 (ppm)

HRMS

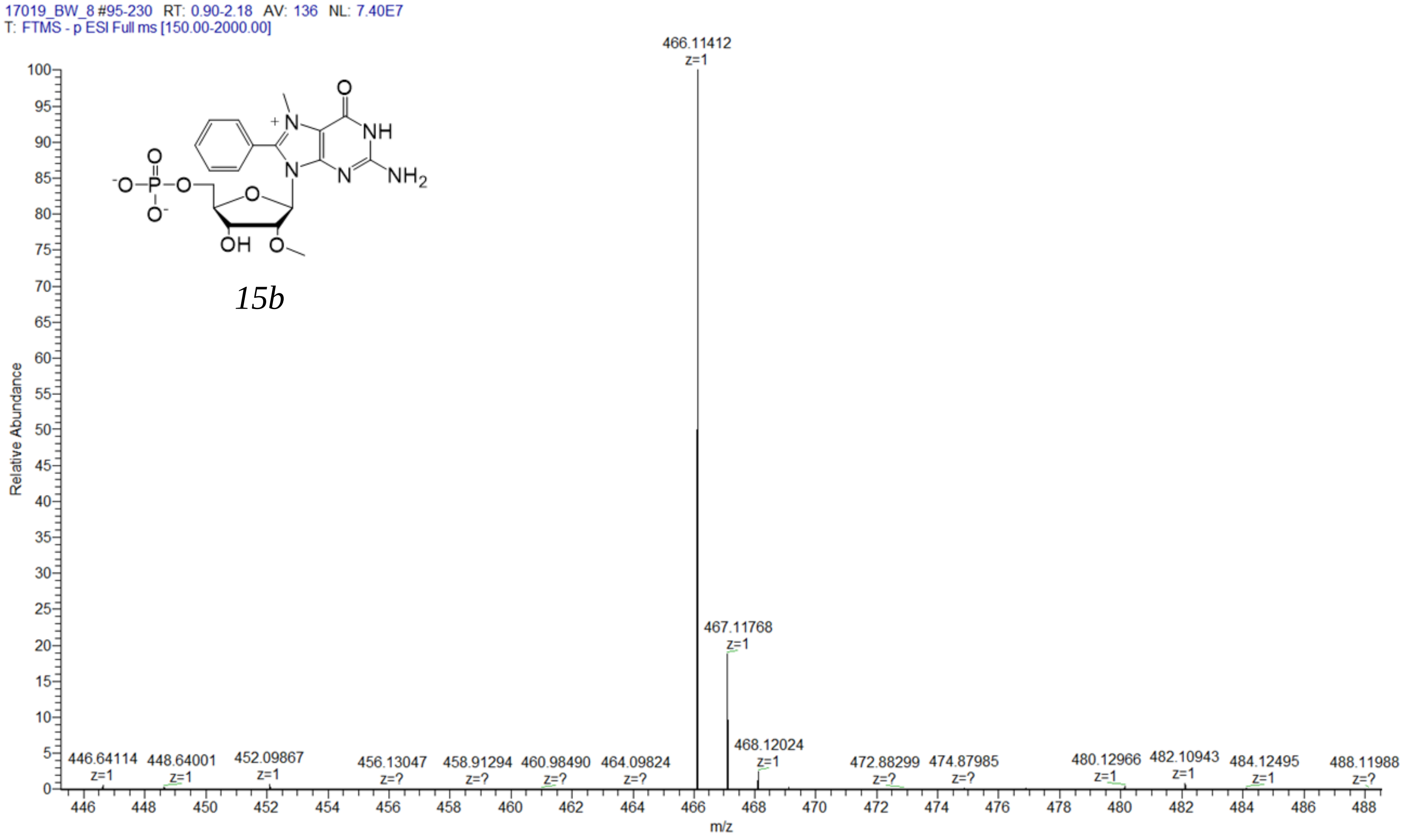

15b

Summary

Rt (D) = 10.12 min; ^{1}H NMR (500 MHz, D_2O) δ 7.85 (t, *J* = 7.4 Hz, 1H), 7.77 (t, *J* = 7.4 Hz, 2H), 7.72 (m, 2H), 5.68 (d, *J* = 5.7 Hz, 1H), 4.99 (dd, *J* = 5.7, 5.4 Hz, 1H), 4.69 (dd, *J* = 5.4 Hz, 4.1 Hz, 1H), 4.22–4.02 (m, 3H), 3.94 (s, 3H), 3.36 (s, 3H); ^{31}P NMR (202 MHz, D_2O) δ 2.02 (t, *J* = 5.5 Hz, 1P); HRMS ESI (-) m/z [M-H]$^-$, calcd for $C_{18}H_{21}N_5O_8P^-$ 466.1133; found 466.1141.

$^{8DMAPh}m^{2'O,7}$GMP (15c)

Structure

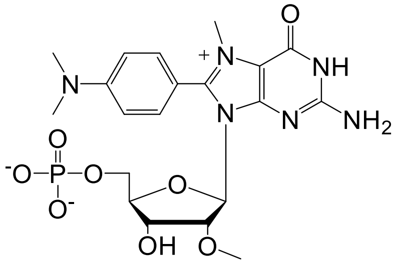

RP-HPLC profile

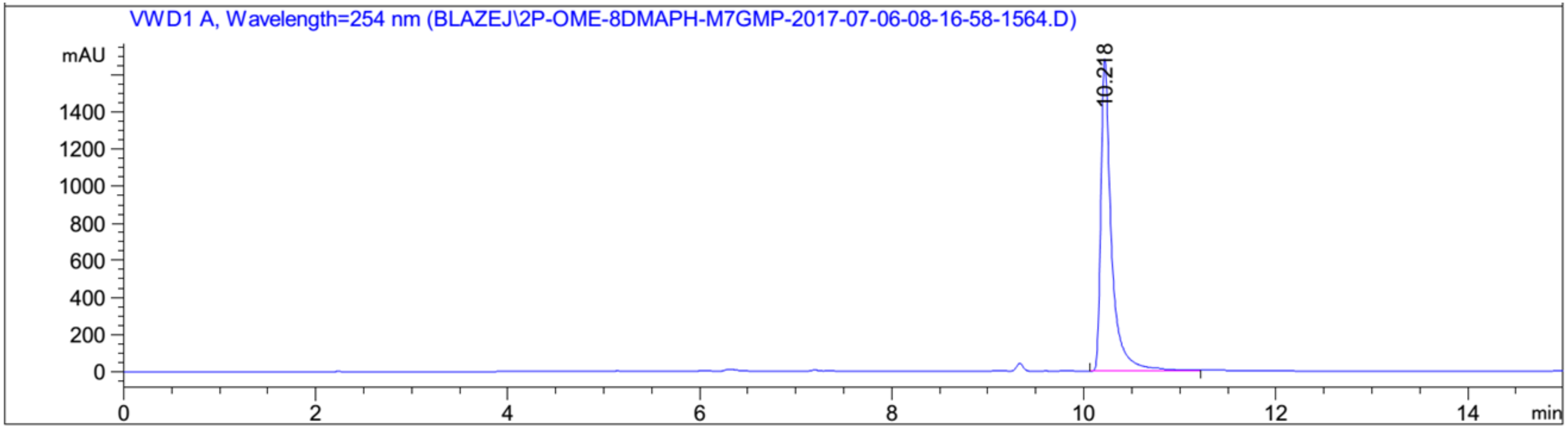

^{1}H NMR

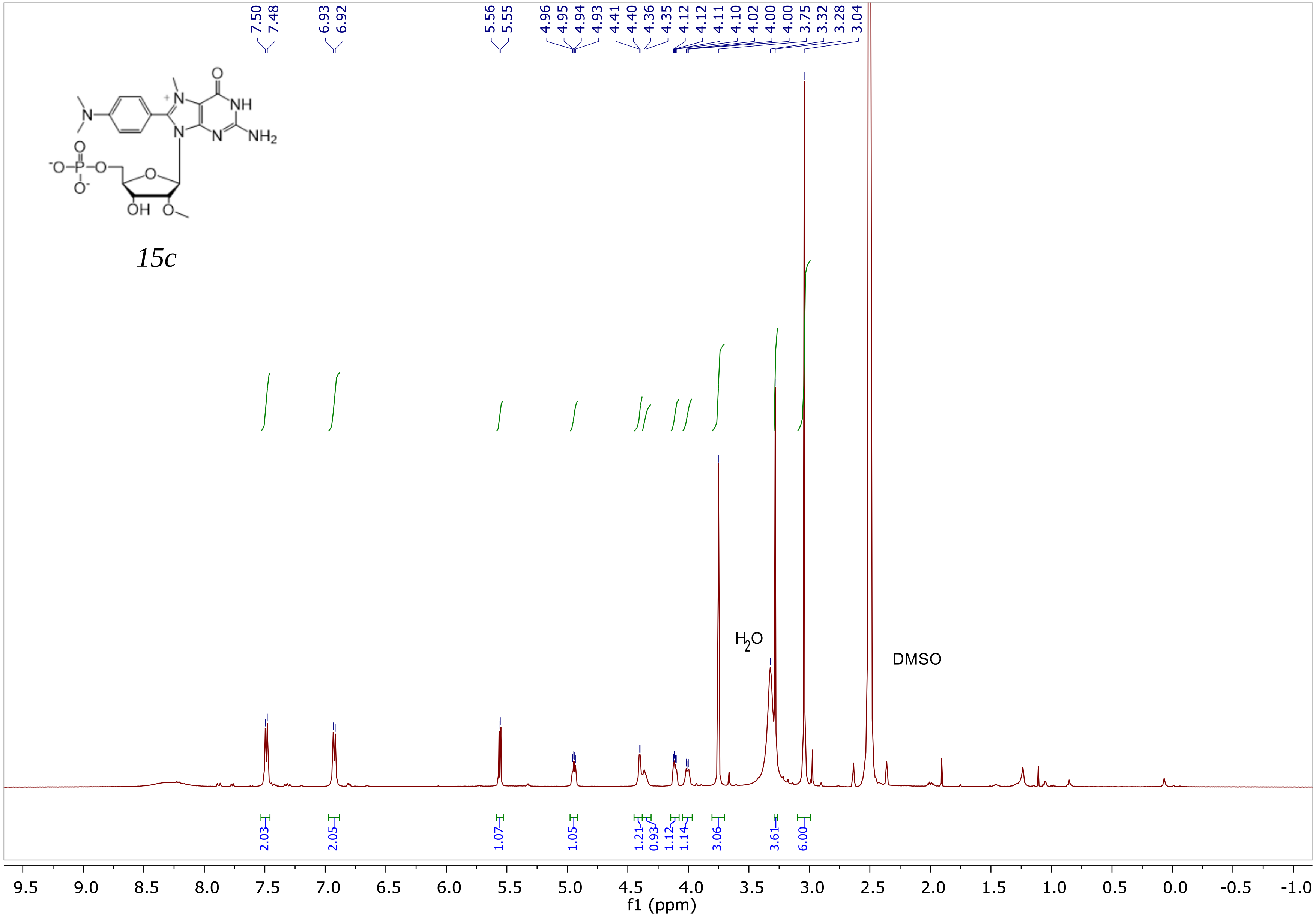

7.50
7.48
6.93
6.92
5.56
5.55
4.96
4.95
4.94
4.93
4.41
4.40
4.36
4.35
4.12
4.12
4.11
4.10
4.02
4.00
4.00
3.75
3.32
3.28
3.04
H2O
DMSO
2.03
2.05
1.07
1.05
1.21
0.93
1.12
1.14
3.06
3.61
6.00
9.5 9.0 8.5 8.0 7.5 7.0 6.5 6.0 5.5 5.0 4.5 4.0 3.5 3.0 2.5 2.0 1.5 1.0 0.5 0.0 -0.5 -1.0
f1 (ppm)

15c

^{1}H-^{1}H COSY NMR

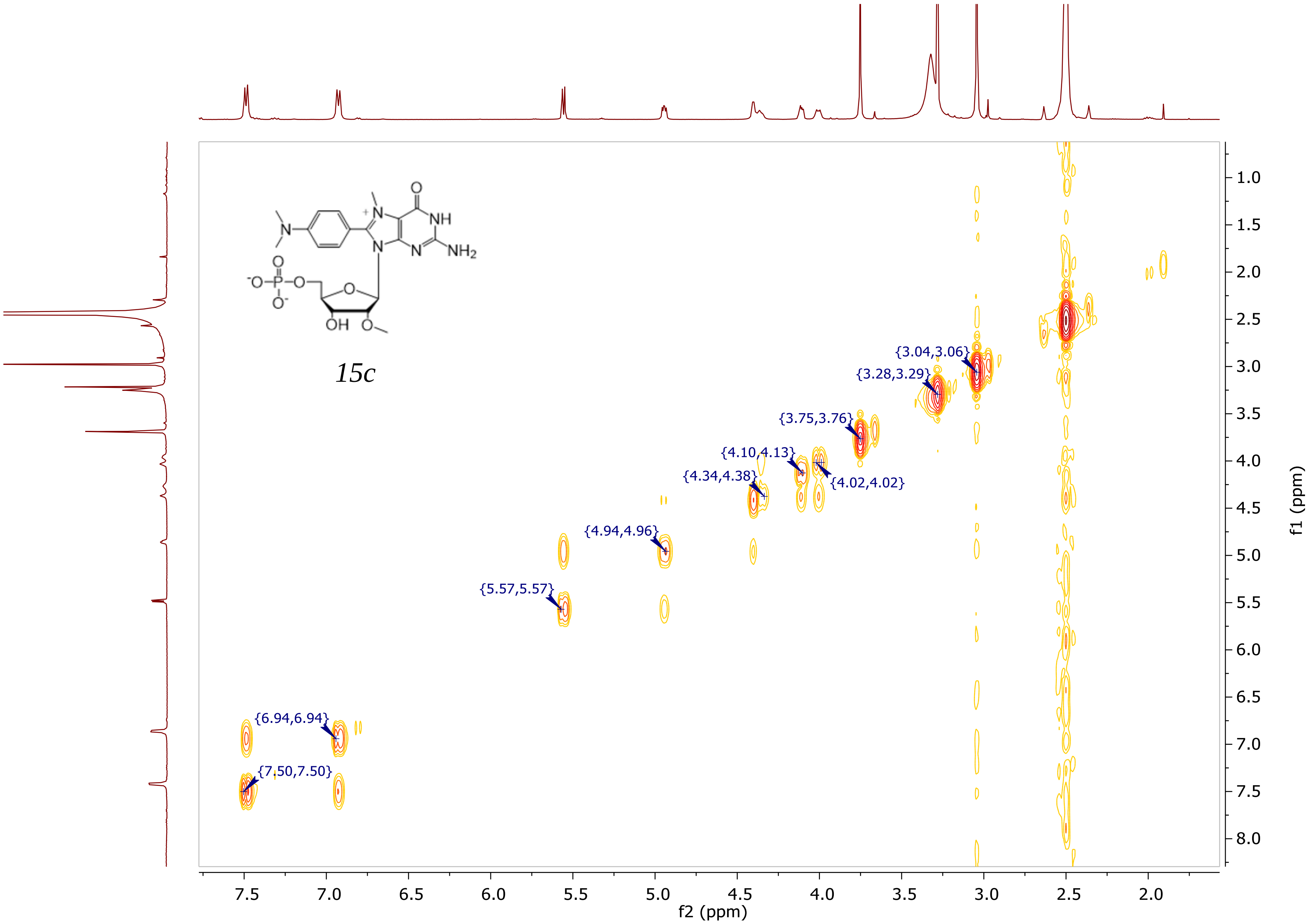

^{31}P NMR

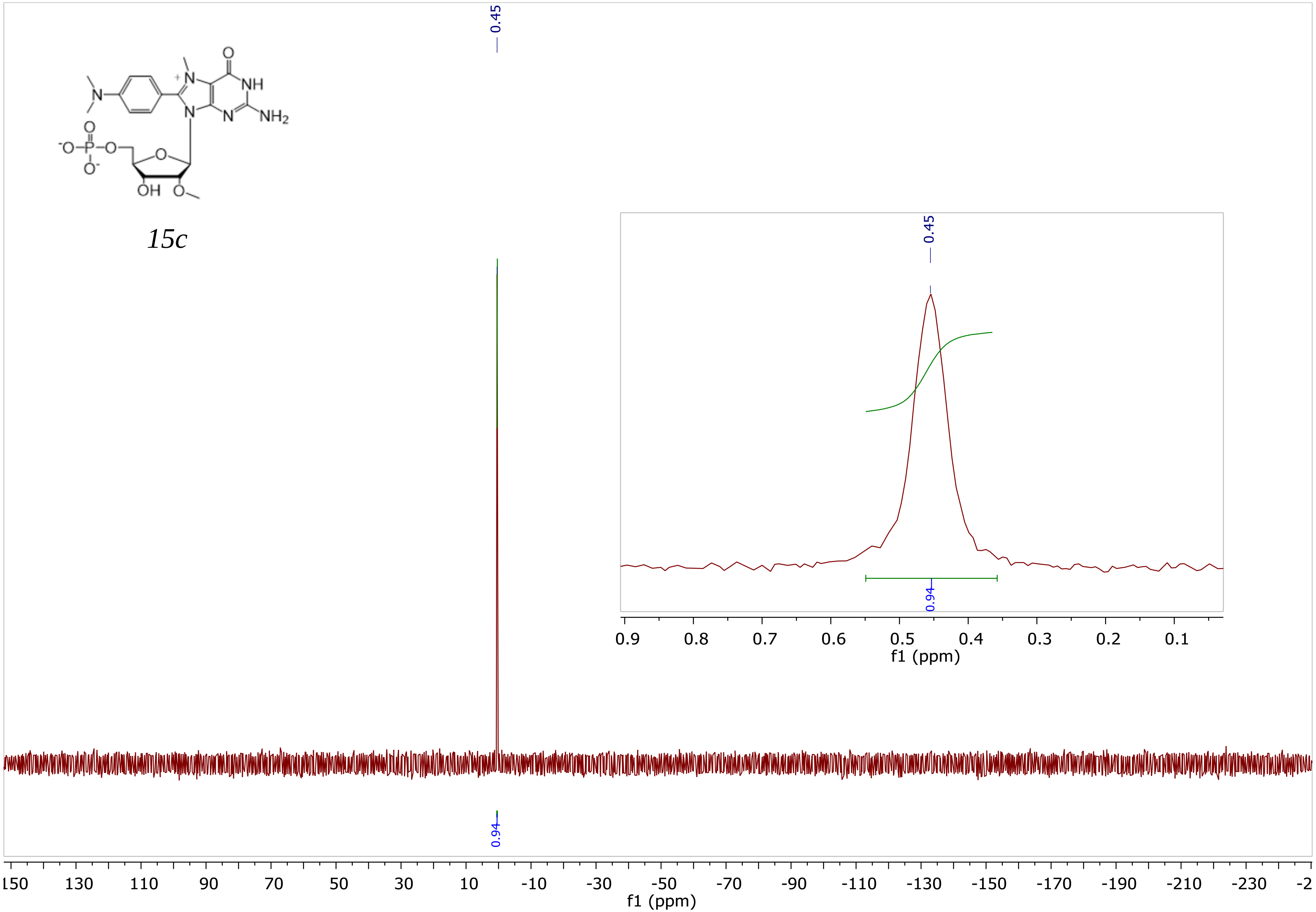

O
NH
N
NH2
O
OH
15c
0.45
0.94
0.9
0.8
0.7
0.6
0.5
0.4
0.3
0.2
0.1
f1 (ppm)
150
130
110
90
70
50
30
10
-10
-30
-50
-70
-90
-110
-130
-150
-170
-190
-210
-230
f1 (ppm)

HRMS

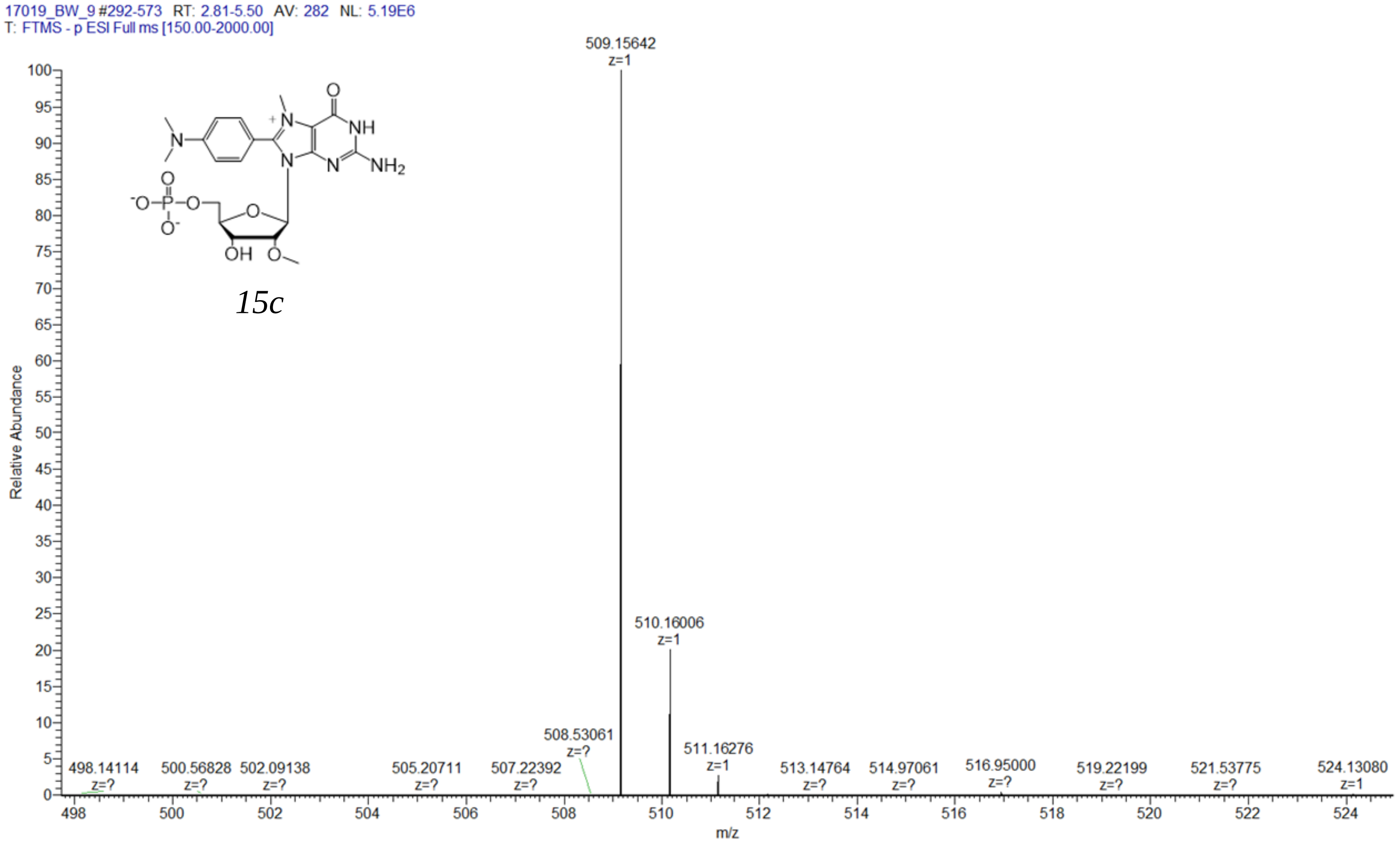

Summary

Rt (D) = 10.22 min; ^{1}H NMR (500 MHz, DMSO-d_6) δ 7.49 (d, *J* = 8.5 Hz, 2H), 6.93 (d, *J* = 8.5 Hz, 2H), 5.56 (d, *J* = 7.4 Hz, 1H), 4.94 (dd, *J* = 7.4 Hz, 4.0 Hz, 1H), 4.40 (d, *J* = 4.0 Hz, 1H), 4.36 (m, 1H), 4.11 (dd, *J* = 7.9 Hz, 3.8 Hz, 1H), 4.01 (m, 2H), 3.75 (s, 3H), 3.04 (s, 6H); ^{31}P NMR (202 MHz, DMSO-d_6) δ 0.45 (s, 1P); HRMS ESI (-) m/z [M-H]$^-$, calcd for $C_{20}H_{26}N_6O_8P^-$ 509.1555; found 509.1564.

$^{8PhCN}m^{2'O,7}GMP$ (15d)

Structure

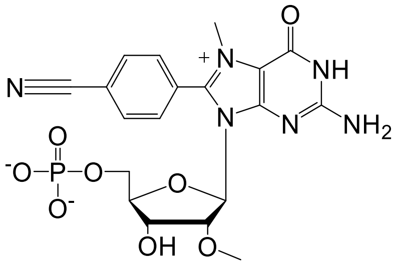

RP-HPLC profile

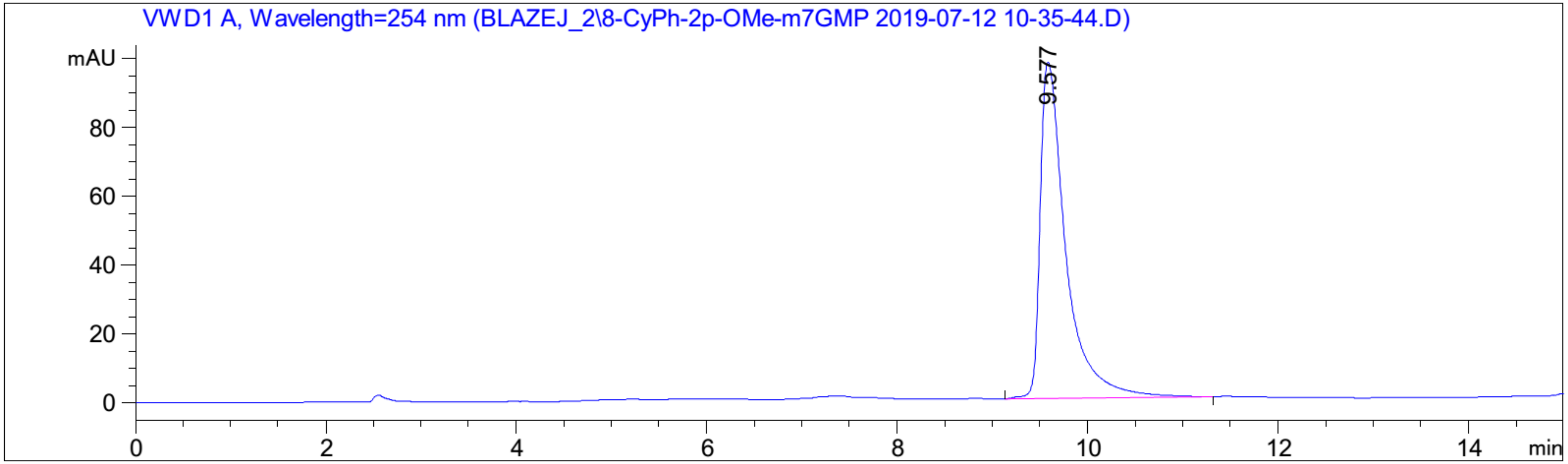

^1H NMR

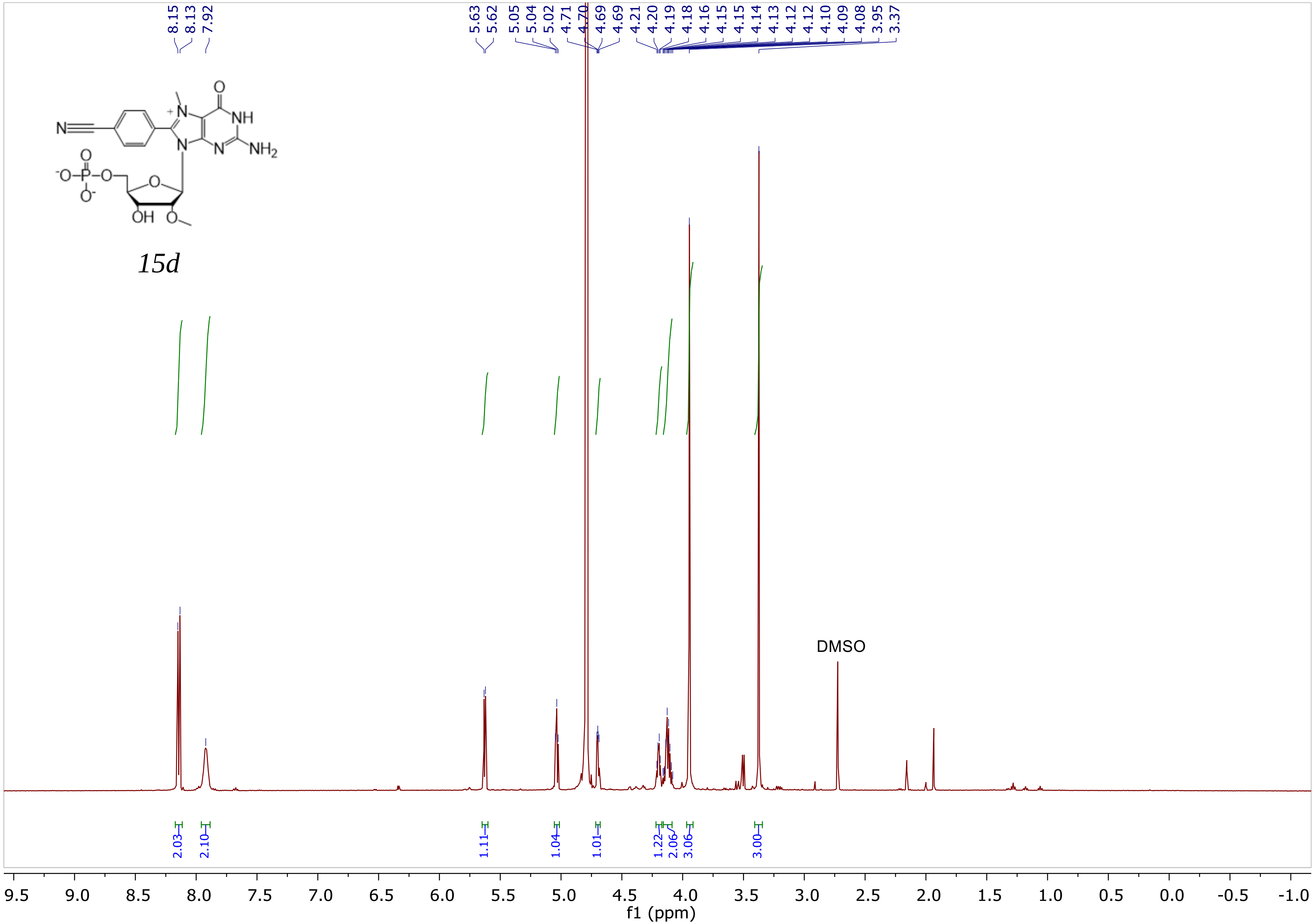

N
NH
NH2
O
OH
15d
8.15
8.13
7.92
5.63
5.62
5.05
5.04
5.02
4.71
4.70
4.69
4.69
4.21
4.20
4.19
4.18
4.16
4.15
4.15
4.14
4.13
4.12
4.12
4.10
4.09
4.08
3.95
3.37
DMSO
2.03
2.10
1.11
1.04
1.01
1.22
2.06
3.06
3.00
9.5
9.0
8.5
8.0
7.5
7.0
6.5
6.0
5.5
5.0
4.5
4.0
3.5
3.0
2.5
2.0
1.5
1.0
0.5
0.0
-0.5
-1.0
f1 (ppm)

^1H-^1H COSY NMR

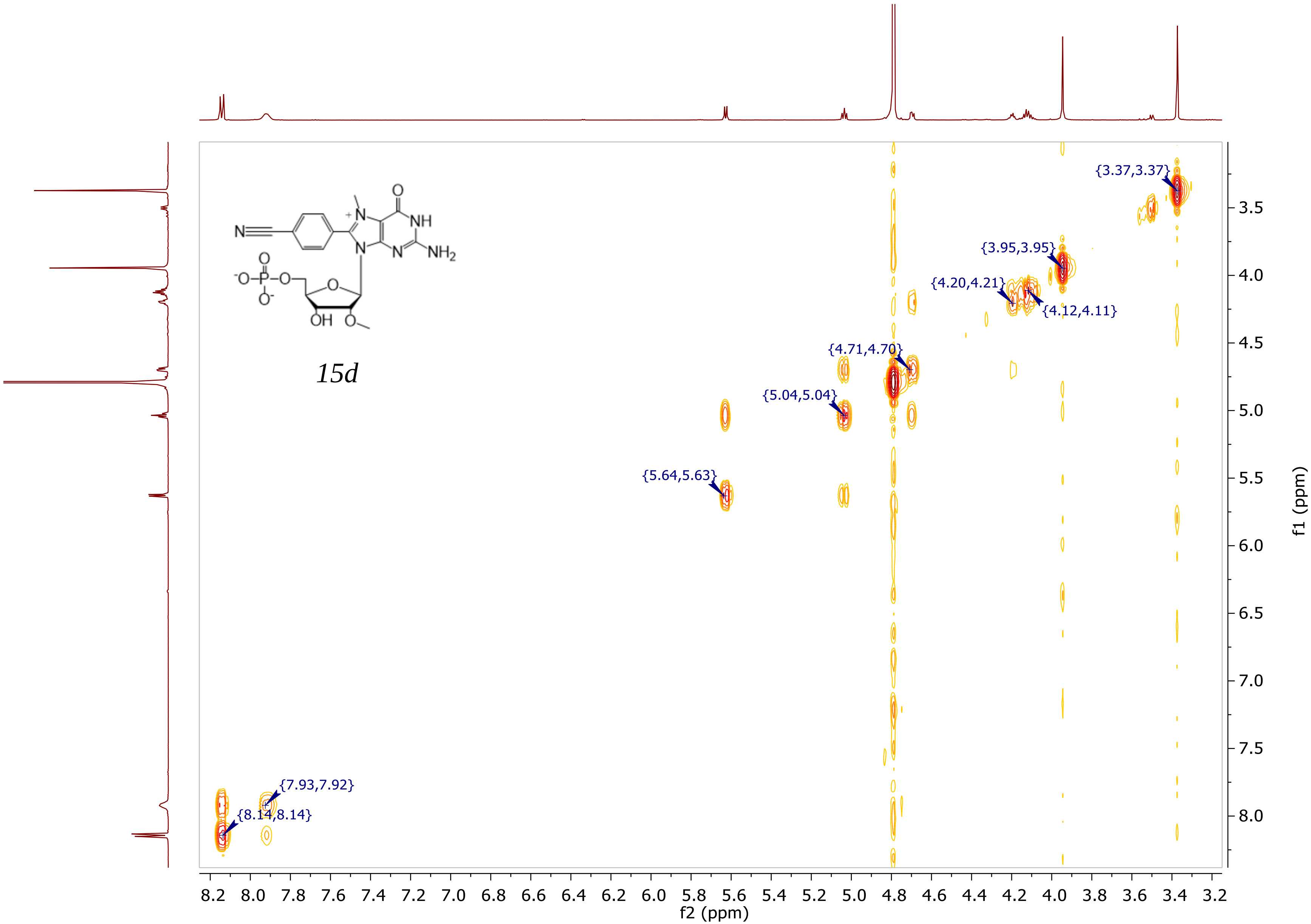

^{31}P NMR

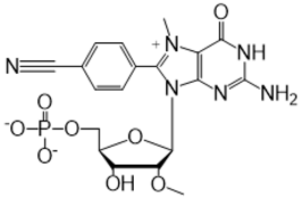

15d

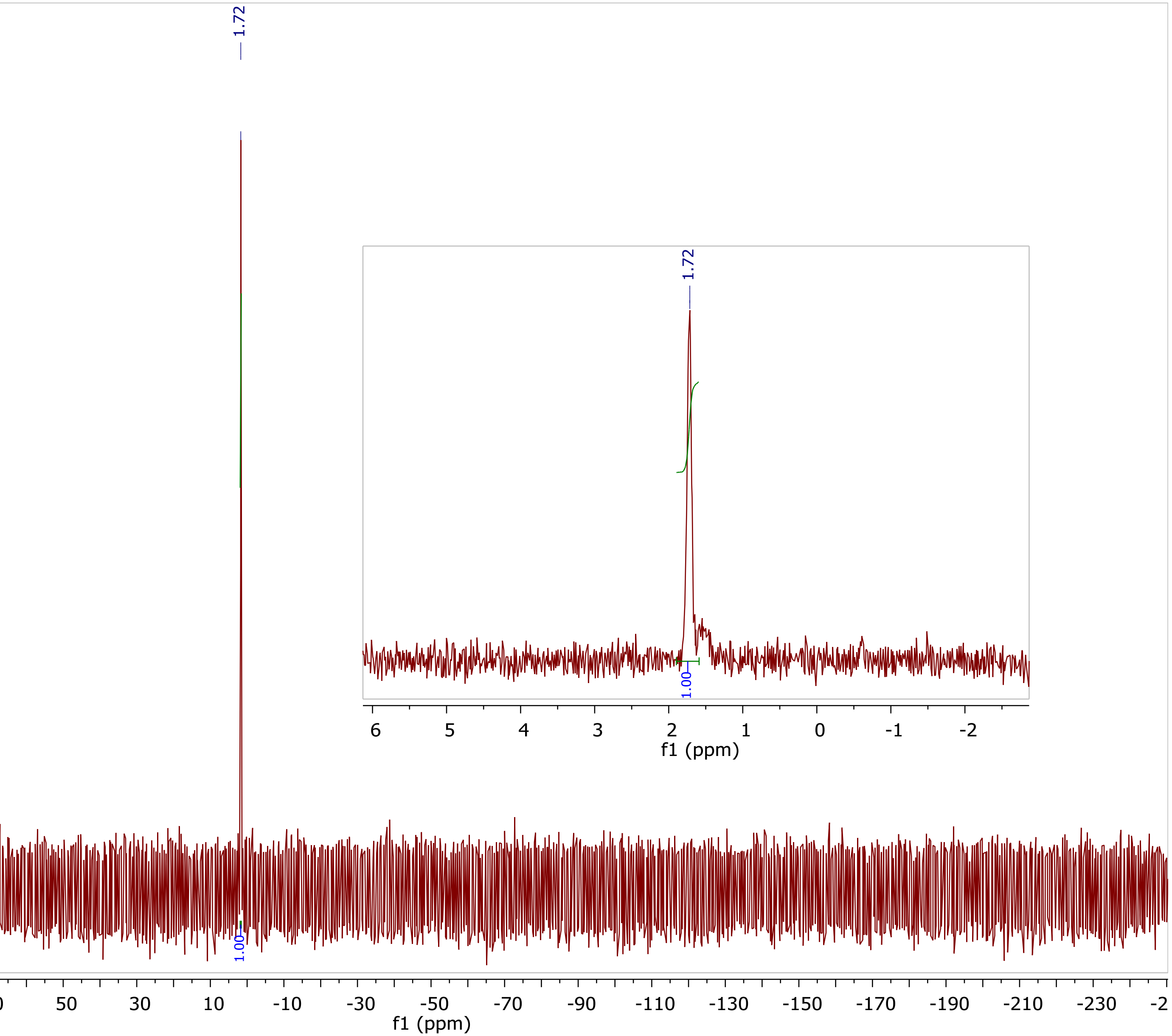

1.72
1.00
6
5
4
3
2
1
0
-1
-2
f1 (ppm)
150
130
110
90
70
50
30
10
-10
-30
-50
-70
-90
-110
-130
-150
-170
-190
-210
-230
-2
f1 (ppm)

HRMS

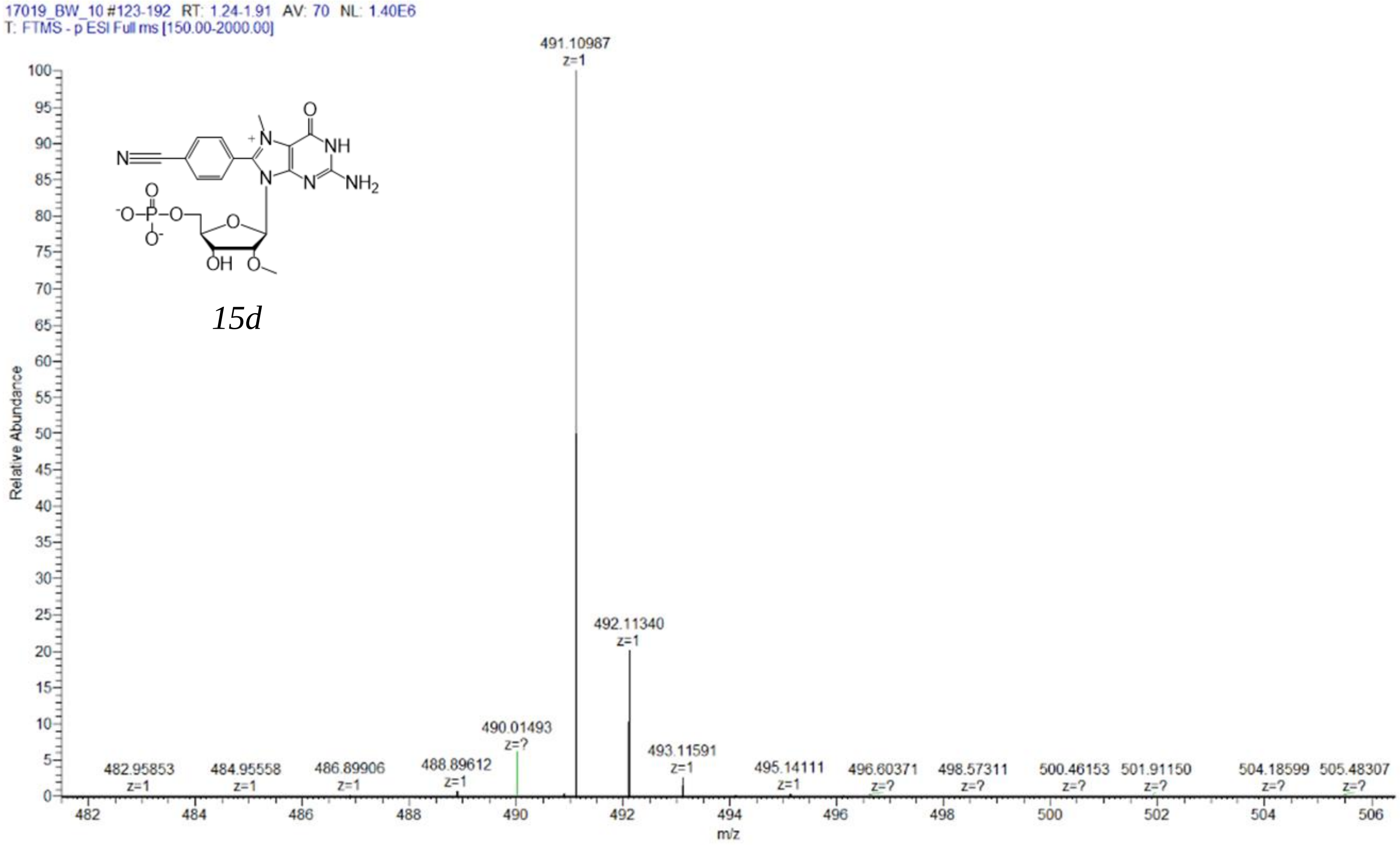

15d

Summary

Rt (D) = 7.07 min; ^{1}H NMR (500 MHz, D_2O) δ 8.14 (d, *J* = 8.8 Hz, 1H), 7.92 (bs, 2H), 5.63 (d, *J* = 5.9 Hz, 1H), 5.04 (dd, *J* = 5.9 Hz, 5.4 Hz, 1H), 4.70 (dd, *J* = 5.4 Hz, 3.8 Hz, 1H), 4.20 (m, 1H), 4.12 (m, 2H), 3.95 (s, 3H), 3.37 (s, 3H); ^{31}P NMR (202 MHz, D_2O) δ 1.72 (s, 1P); HRMS ESI (-) m/z [M-H]$^-$, calcd for $C_{19}H_{21}N_6O_8P^-$ 491.1086; found 491.1099.

$^{8\text{-}Me}m^{2'O,7}GMP$ (15e)

Structure

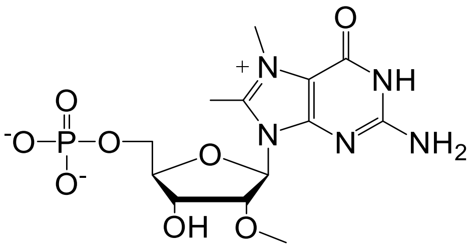

RP-HPLC profile

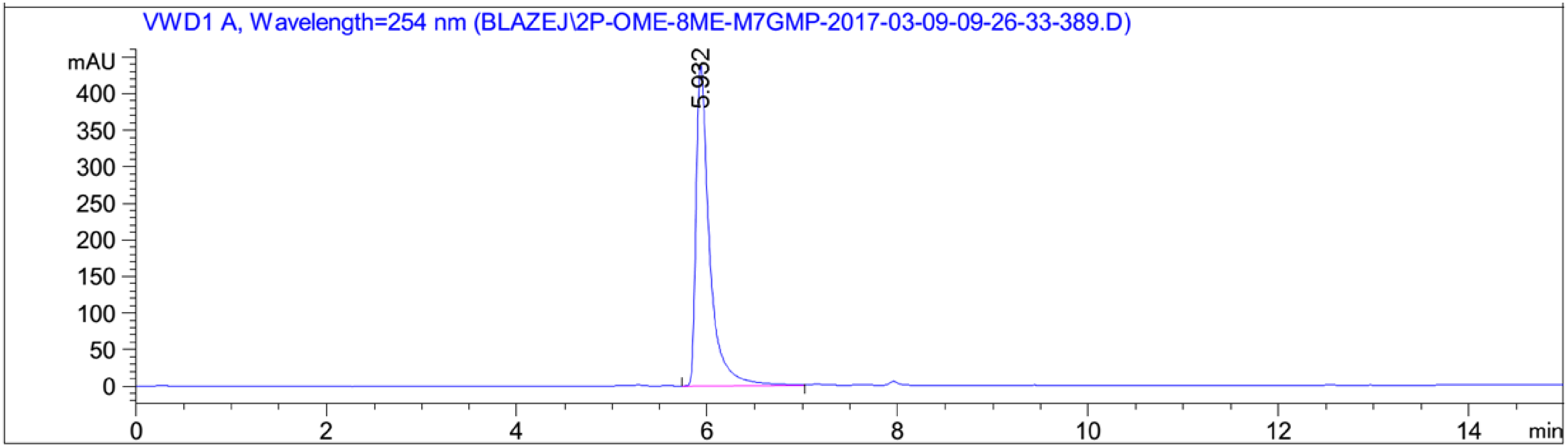

^{1}H NMR

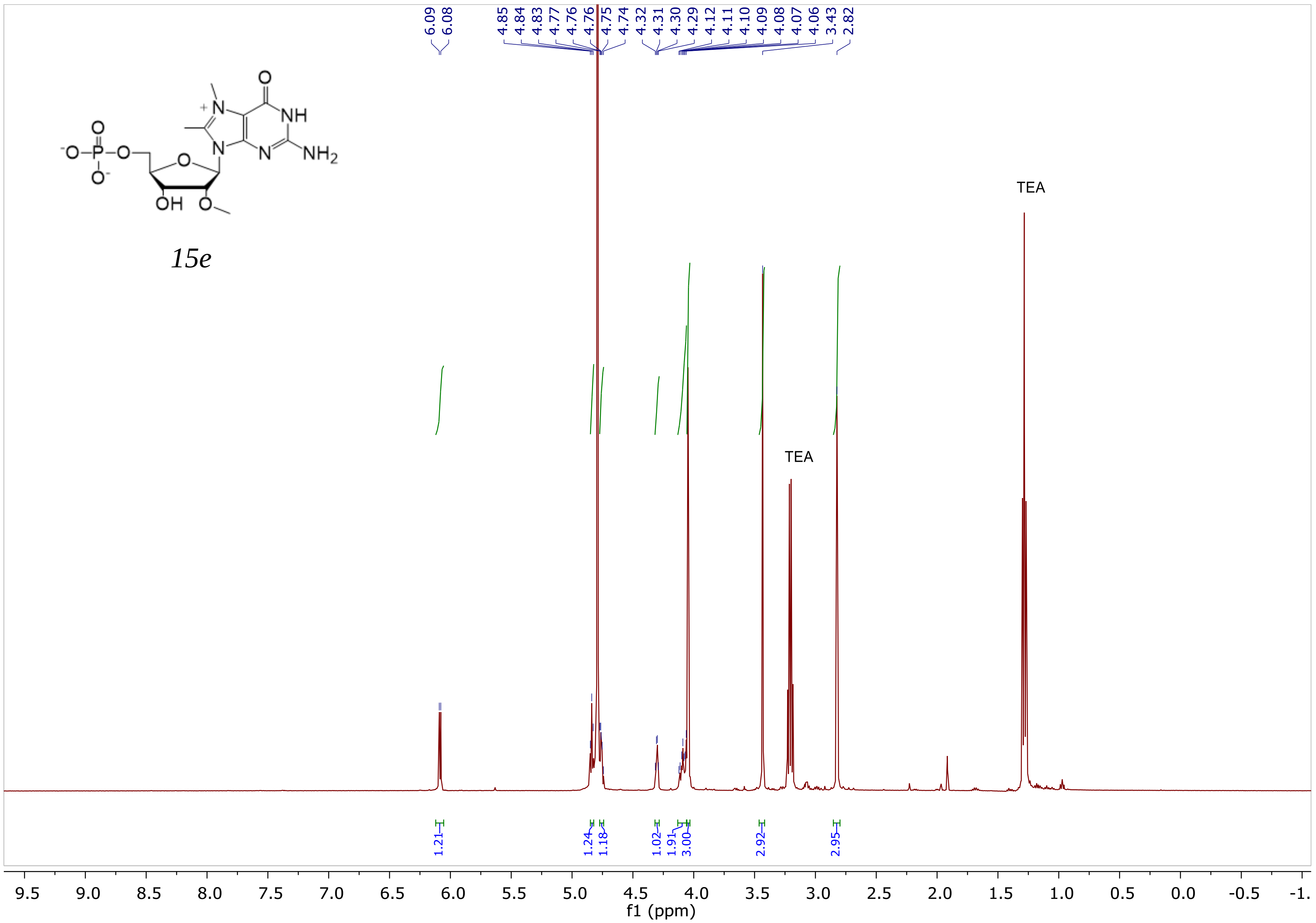
6.09
6.08
4.85
4.84
4.83
4.77
4.76
4.76
4.75
4.74
4.32
4.31
4.30
4.29
4.12
4.11
4.10
4.09
4.08
4.07
4.06
3.43
2.82
TEA
TEA
1.21
1.24
1.18
1.02
1.91
3.00
2.92
2.95
f1 (ppm)

15e

^{31}P NMR

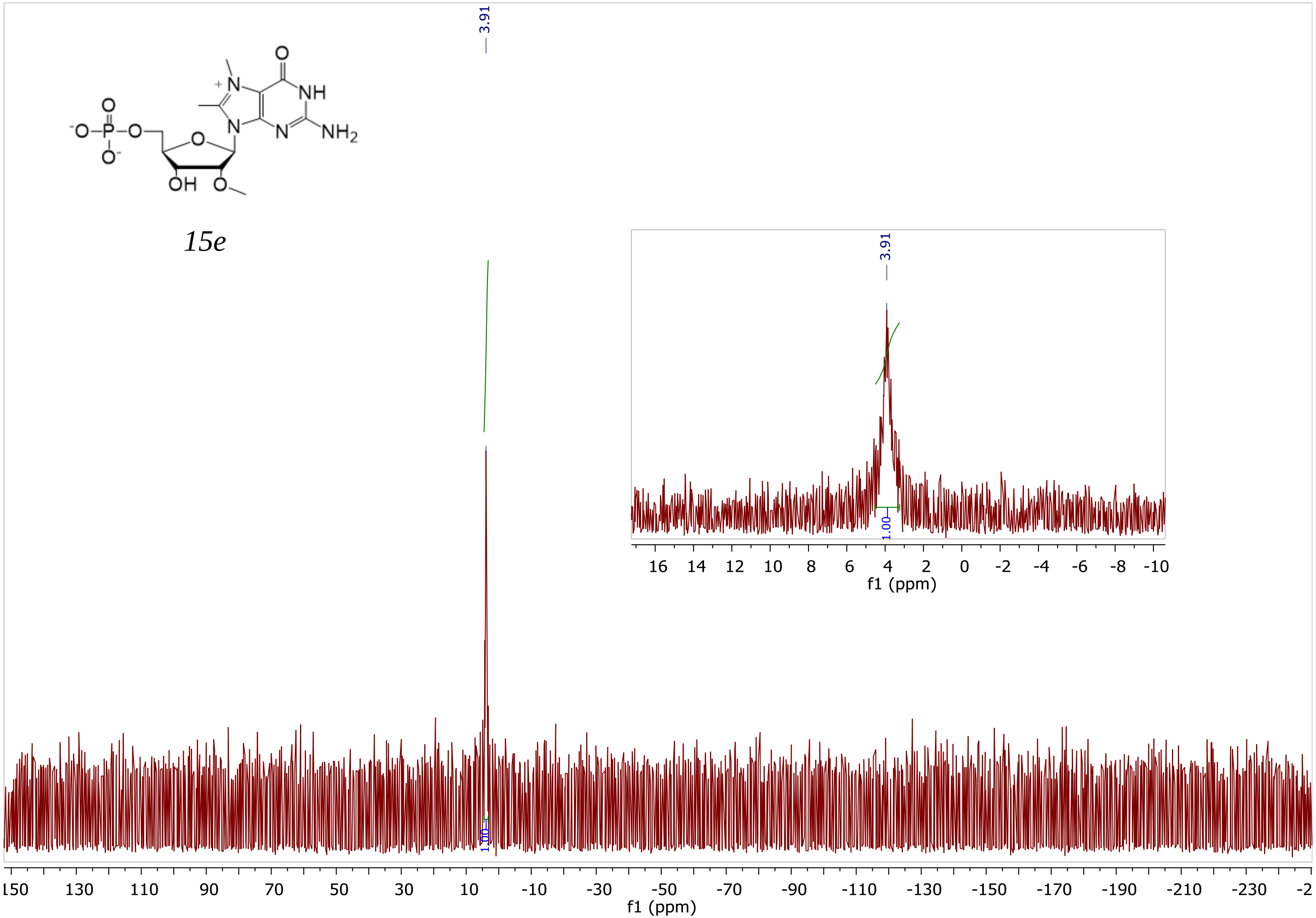

3.91
15e
3.91
1.00
16 14 12 10 8 6 4 2 0 -2 -4 -6 -8 -10
f1 (ppm)
1.00
150 130 110 90 70 50 30 10 -10 -30 -50 -70 -90 -110 -130 -150 -170 -190 -210 -230
f1 (ppm)

HRMS

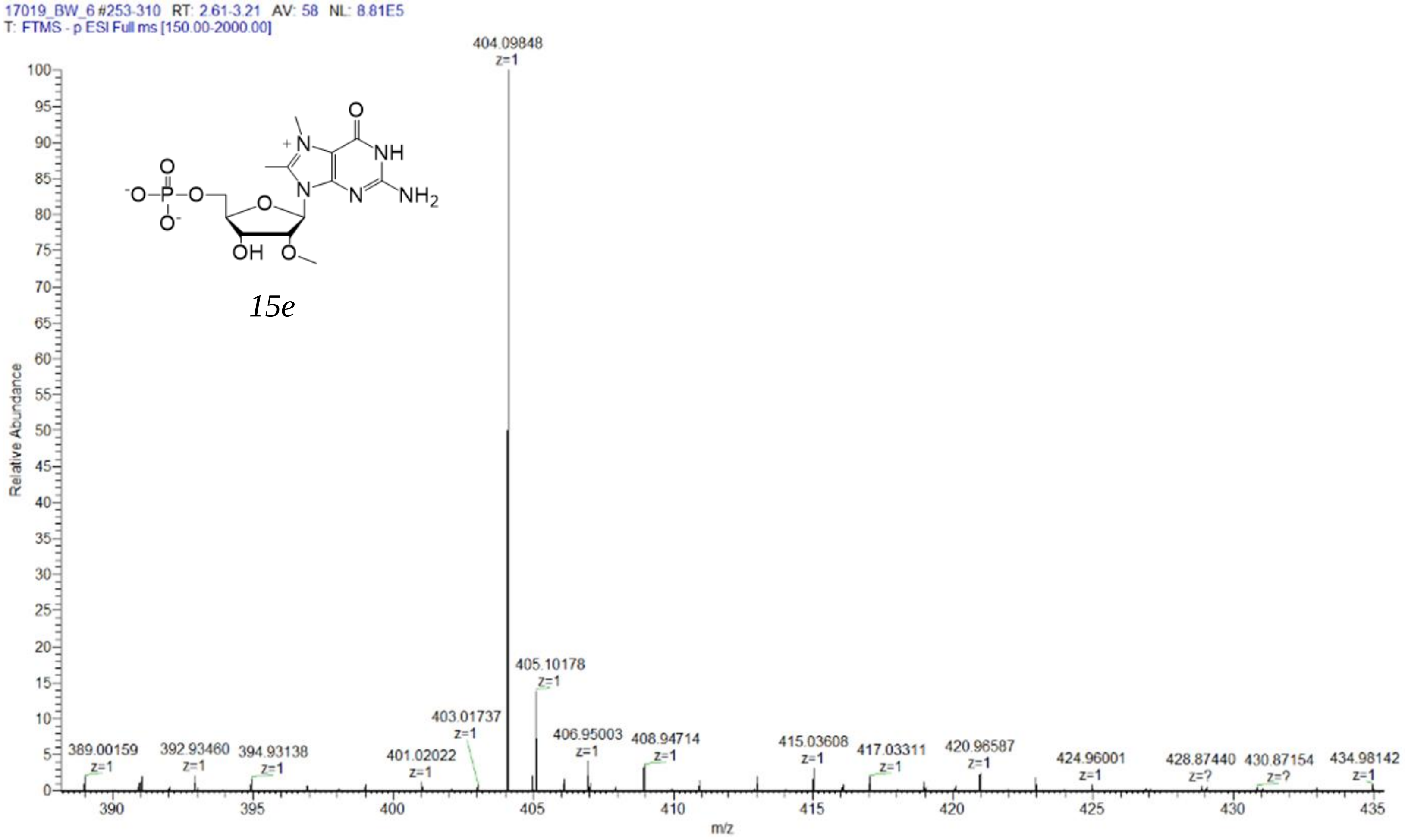

15e

Summary

Rt (A) = 5.93 min; ^{1}H NMR (500 MHz, D_2O) δ 6.08 (d, *J* = 6.1 Hz, 1H), 4.84 (dd, *J* = 6.1, 5.7 Hz, 1H), 4.76 (m, 1H), 4.30 (m, 1H), 4.09 (m, 2H), 4.05 (s, 3H), 3.43 (s, 3H), 2.82 (s, 3H); ^{31}P NMR (202 MHz, D_2O) δ 3.91 (m, 1P); HRMS ESI (-) m/z $[M-H]^-$, calcd for $C_{13}H_{19}N_5O_8P^-$ 404.0977; found 404.0985.

$^{8cPr}m^{2'O,7}GMP$ (15f)

Structure

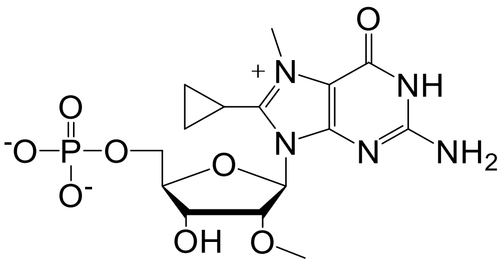

RP-HPLC profile

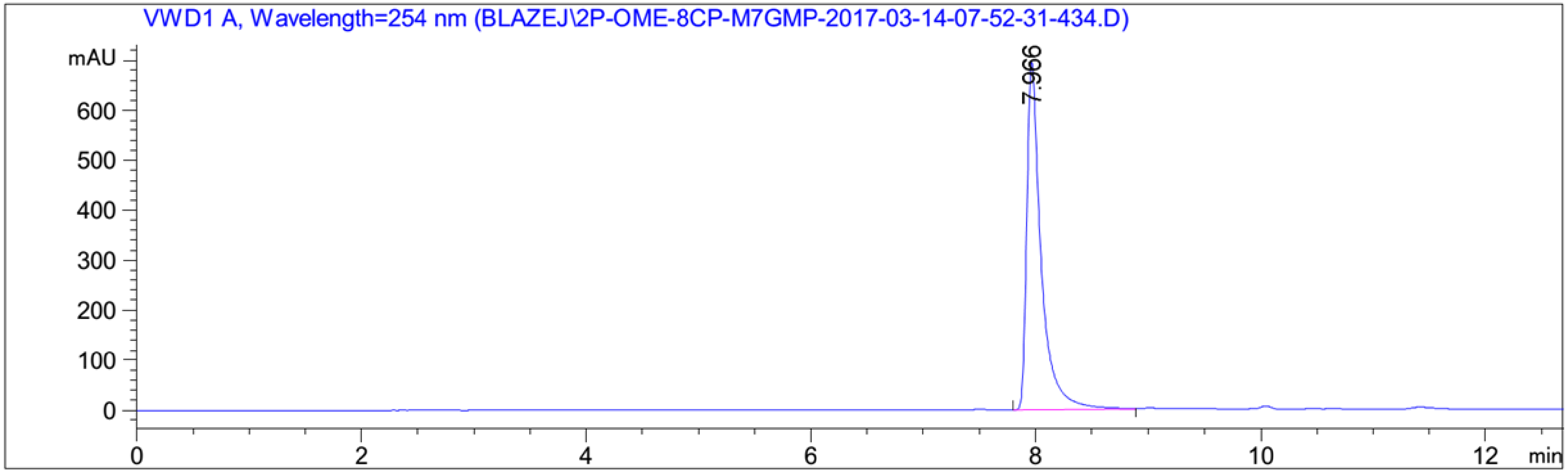

^{1}H NMR

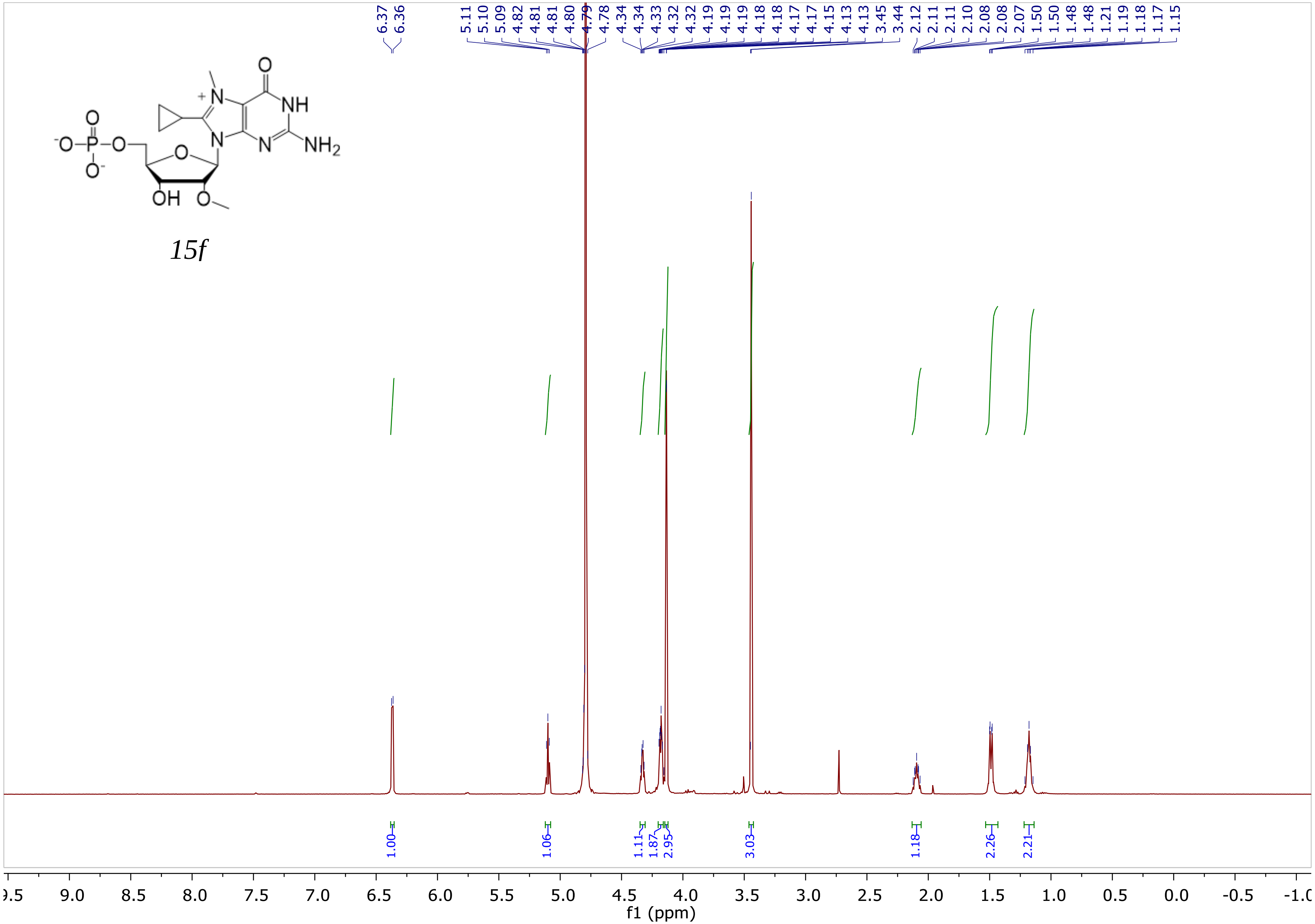

6.37
6.36
5.11
5.10
5.09
4.82
4.81
4.81
4.80
4.79
4.78
4.34
4.34
4.33
4.32
4.32
4.19
4.19
4.19
4.18
4.18
4.17
4.17
4.15
4.13
4.13
3.45
3.44
2.12
2.11
2.11
2.10
2.08
2.08
2.07
1.50
1.50
1.48
1.48
1.21
1.19
1.18
1.17
1.15
1.00
1.06
1.11
1.87
2.95
3.03
1.18
2.26
2.21
9.5
9.0
8.5
8.0
7.5
7.0
6.5
6.0
5.5
5.0
4.5
4.0
3.5
3.0
2.5
2.0
1.5
1.0
0.5
0.0
-0.5
-1.0
f1 (ppm)

15f

^{1}H-^{1}H COSY NMR

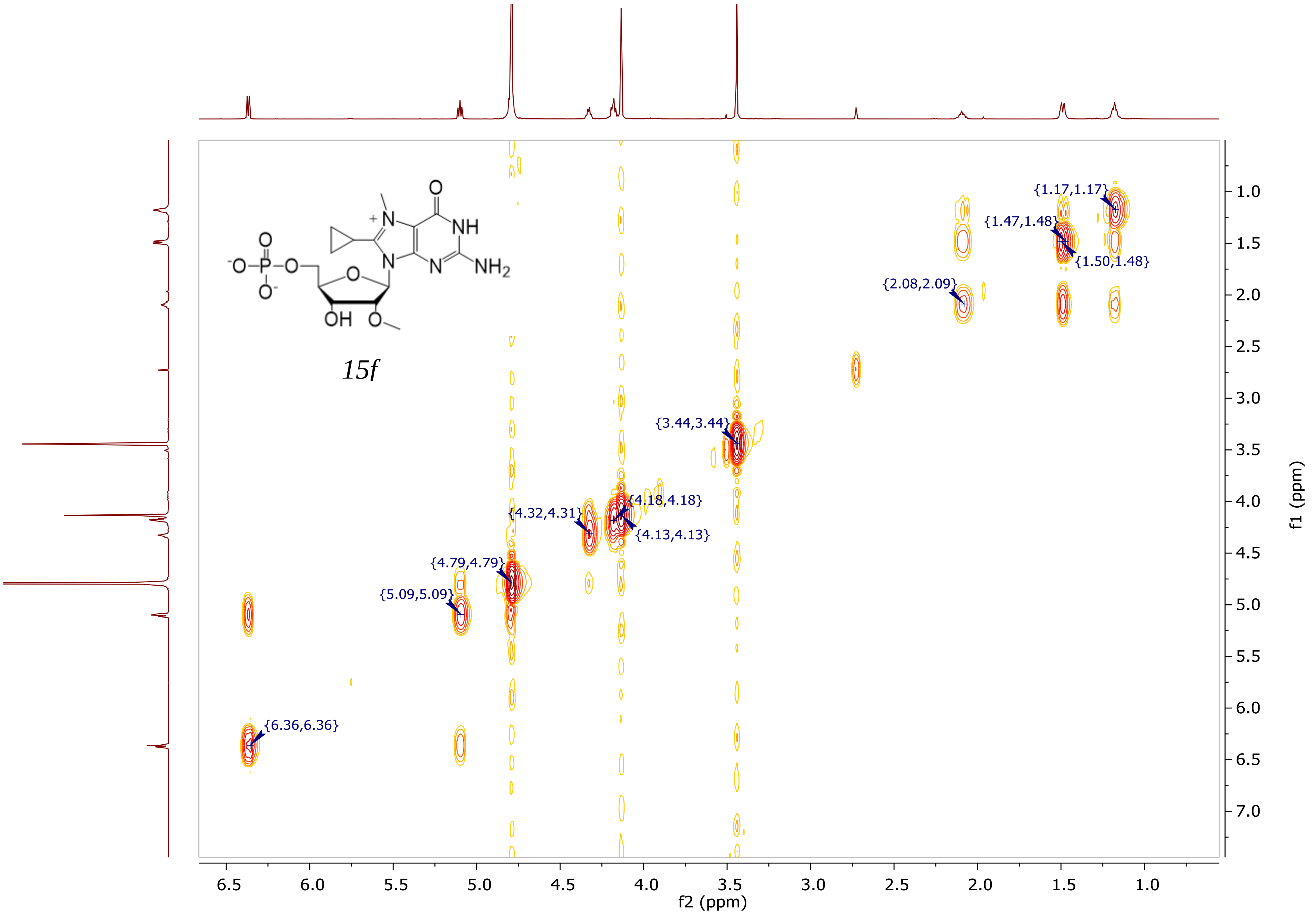

15f

^{31}P NMR

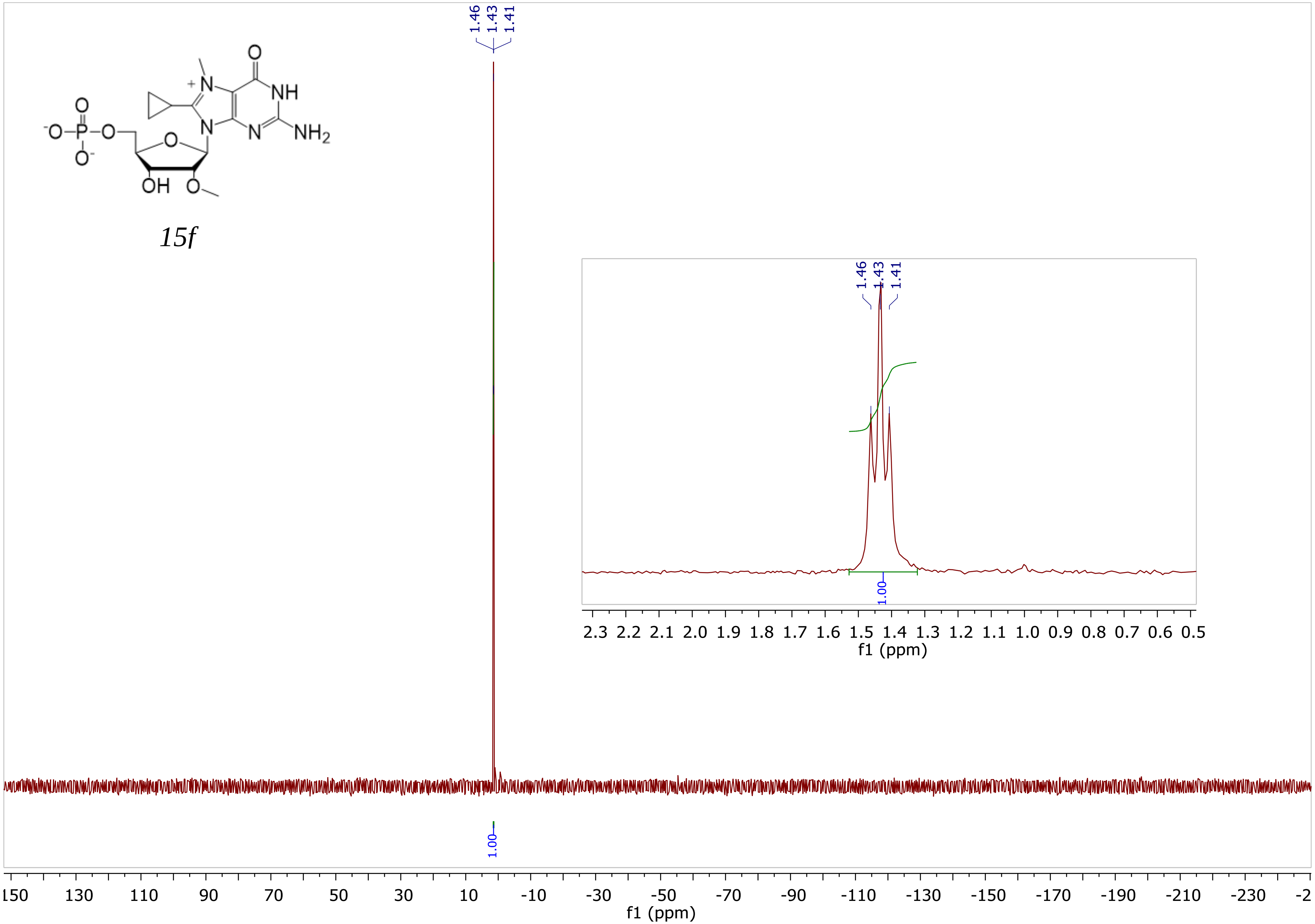

15f

1.46
1.43
1.41
1.46
1.43
1.41
1.00
2.3 2.2 2.1 2.0 1.9 1.8 1.7 1.6 1.5 1.4 1.3 1.2 1.1 1.0 0.9 0.8 0.7 0.6 0.5
f1 (ppm)
1.00
150 130 110 90 70 50 30 10 -10 -30 -50 -70 -90 -110 -130 -150 -170 -190 -210 -230 -2
f1 (ppm)

HRMS

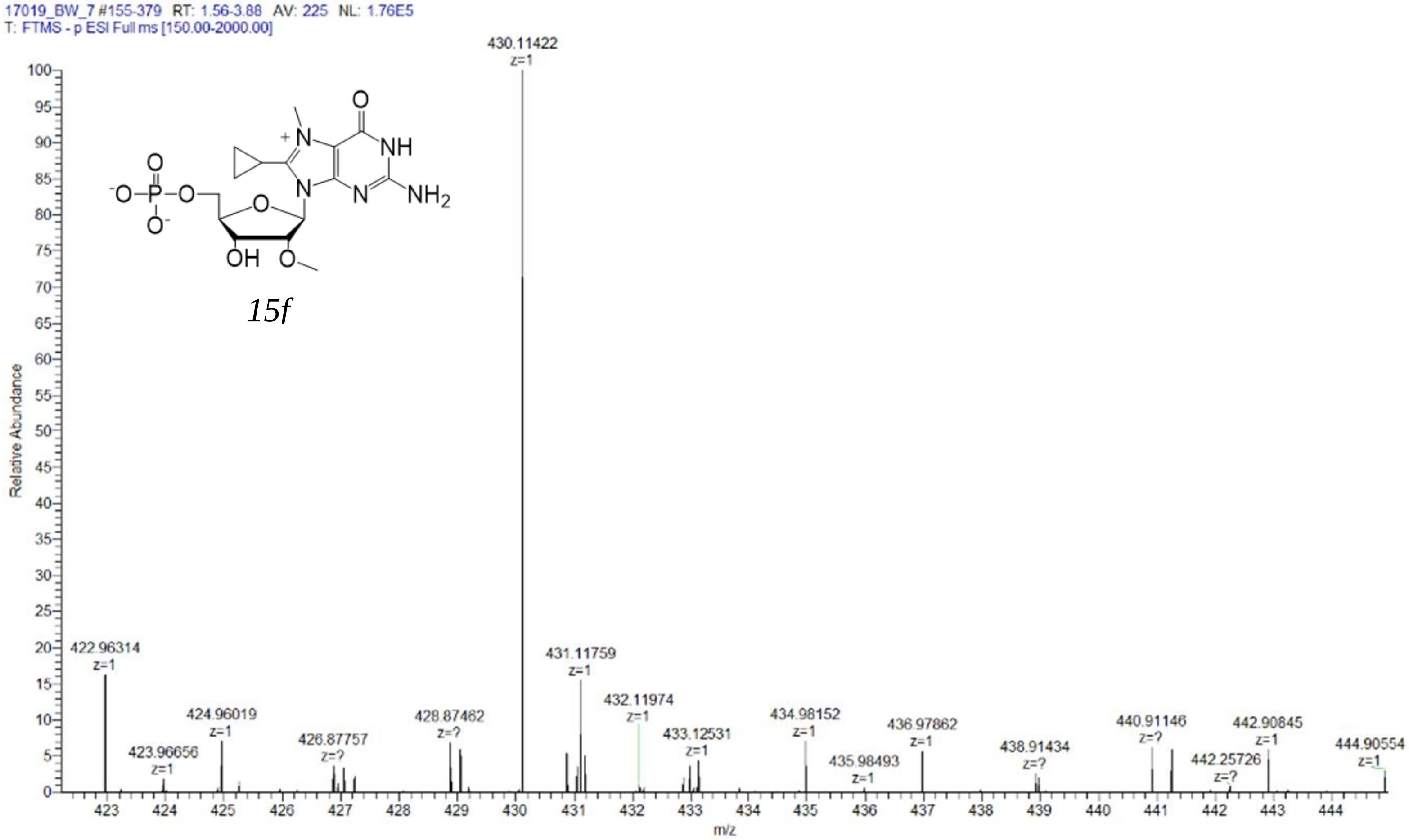

Summary

Rt (A) = 7.97 min; ^{1}H NMR (500 MHz, D_2O) δ 6.37 (d, *J* = 6.2 Hz, 1H), 5.10 (t, *J* = 6.2 Hz, 1H), 4.82–4.79 (m, 1H, overlapped with solvent signal), 4.33 (q, *J* = 6.2 Hz, 4.8 Hz, 1H), 4.18 (m, 2H), 4.13 (s, 3H), 3.44 (s, 3H), 2.10 (dt, *J* = 14.5 Hz, 9.5 Hz, 5.5 Hz, 1H), 1.49 (m, 2H), 1.18 (m, 2H); ^{31}P NMR (202 MHz, D_2O) δ 1.43 (t, *J* = 5.6 Hz, 1P); HRMS ESI (-) m/z [M-H]$^-$, calcd for $C_{15}H_{21}N_5O_8P^-$ 430.1133; found 430.1142.

$^{8\text{-DMAPh}}m^{7}GMP$ (16c)

Structure

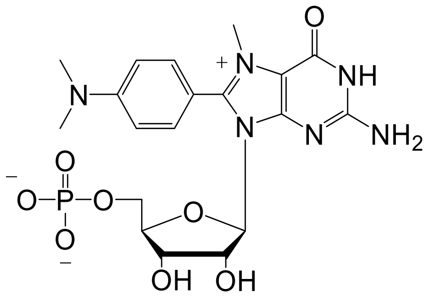

RP-HPLC profile

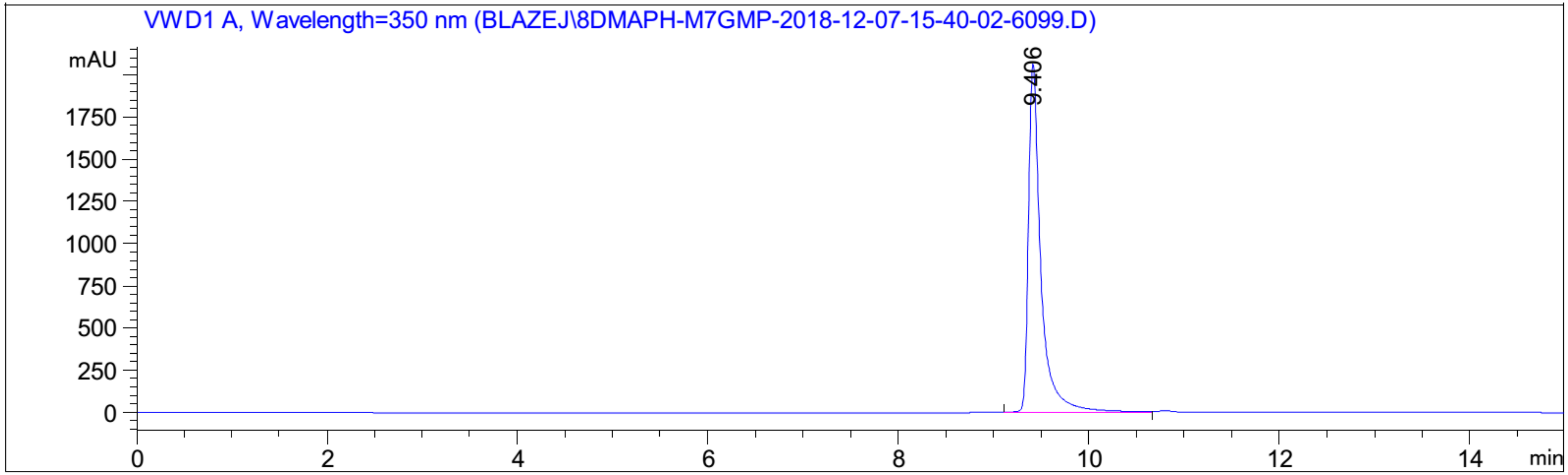

^{1}H NMR

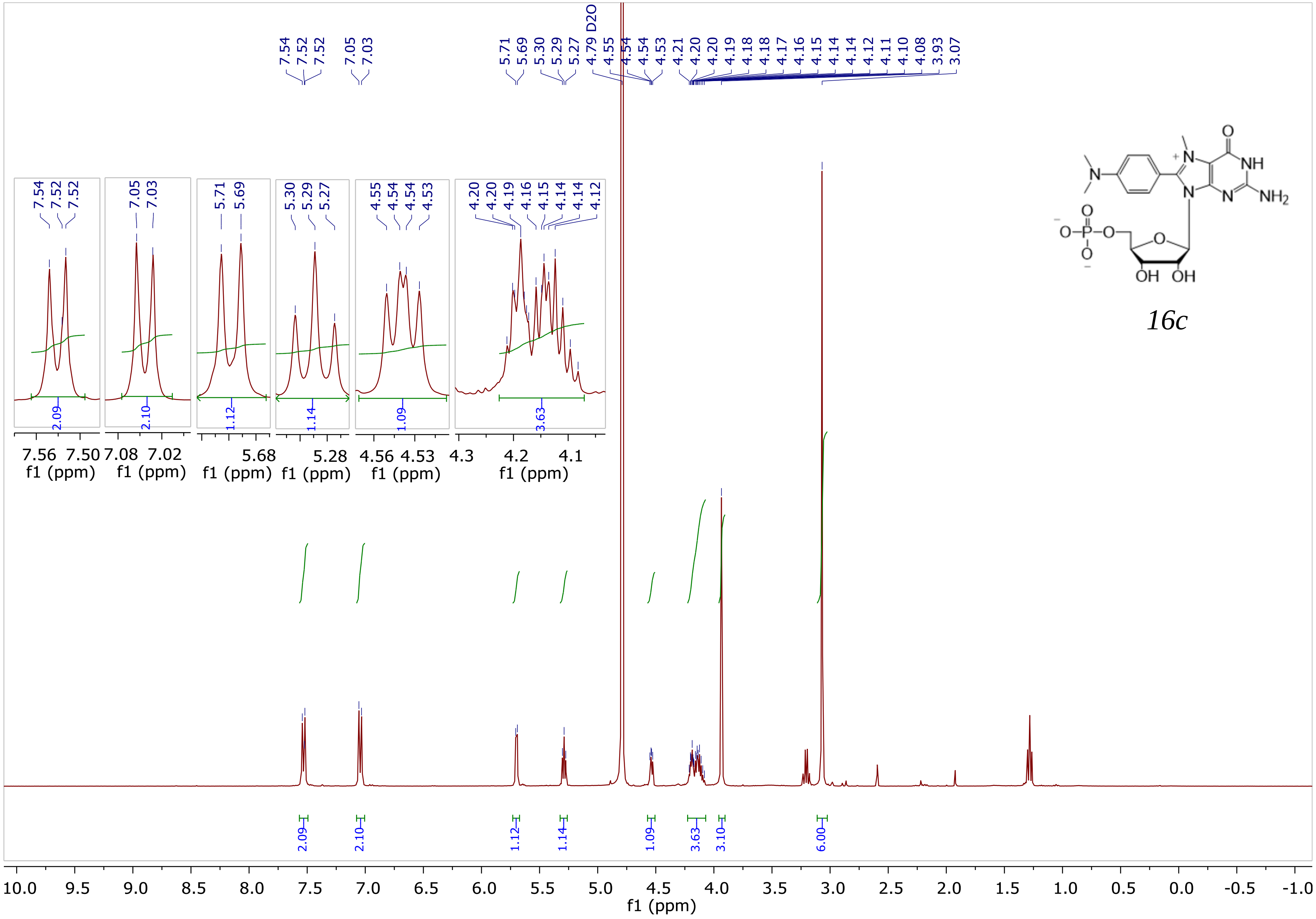

16c

^1H-^1H COSY NMR

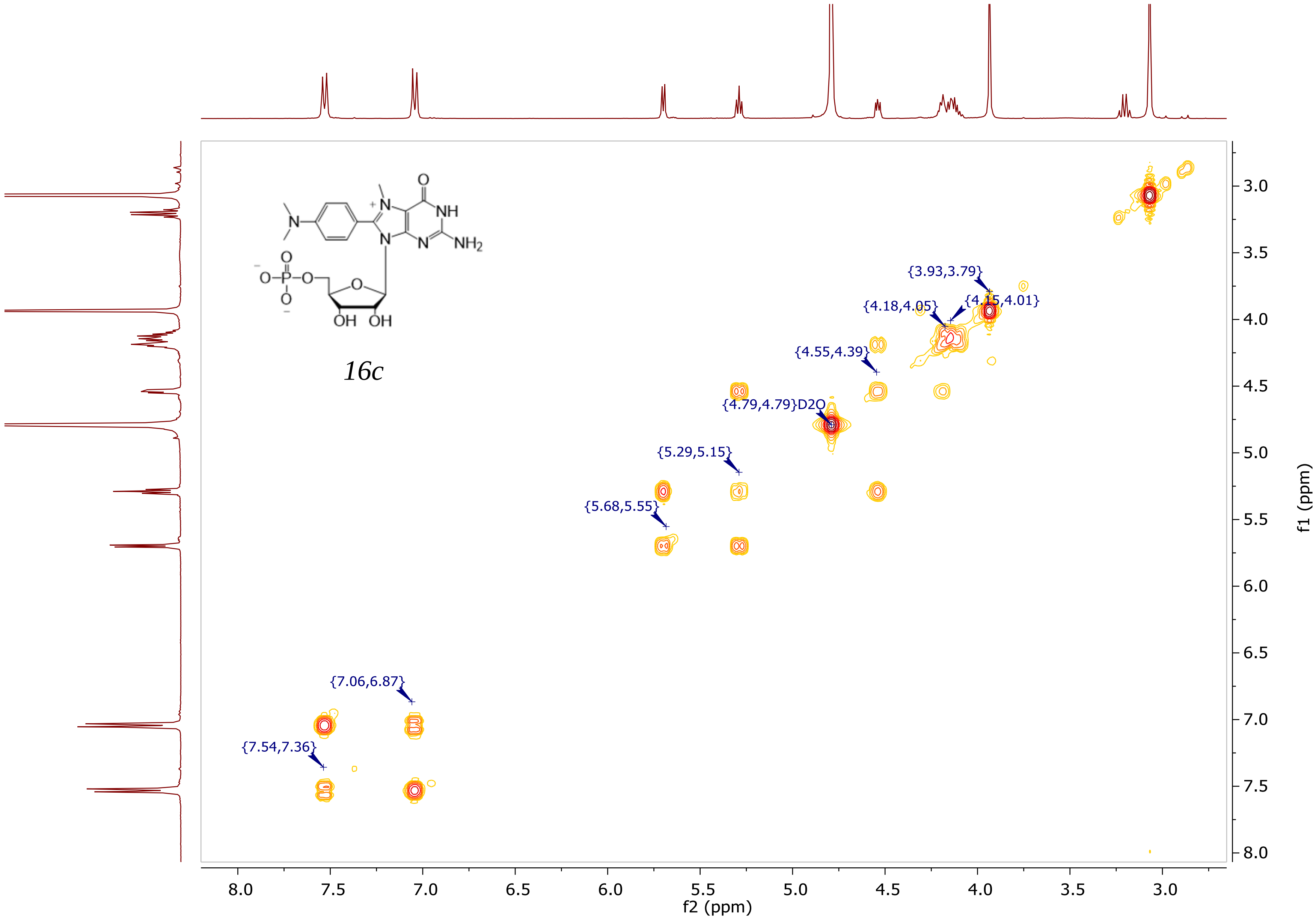

^{31}P NMR

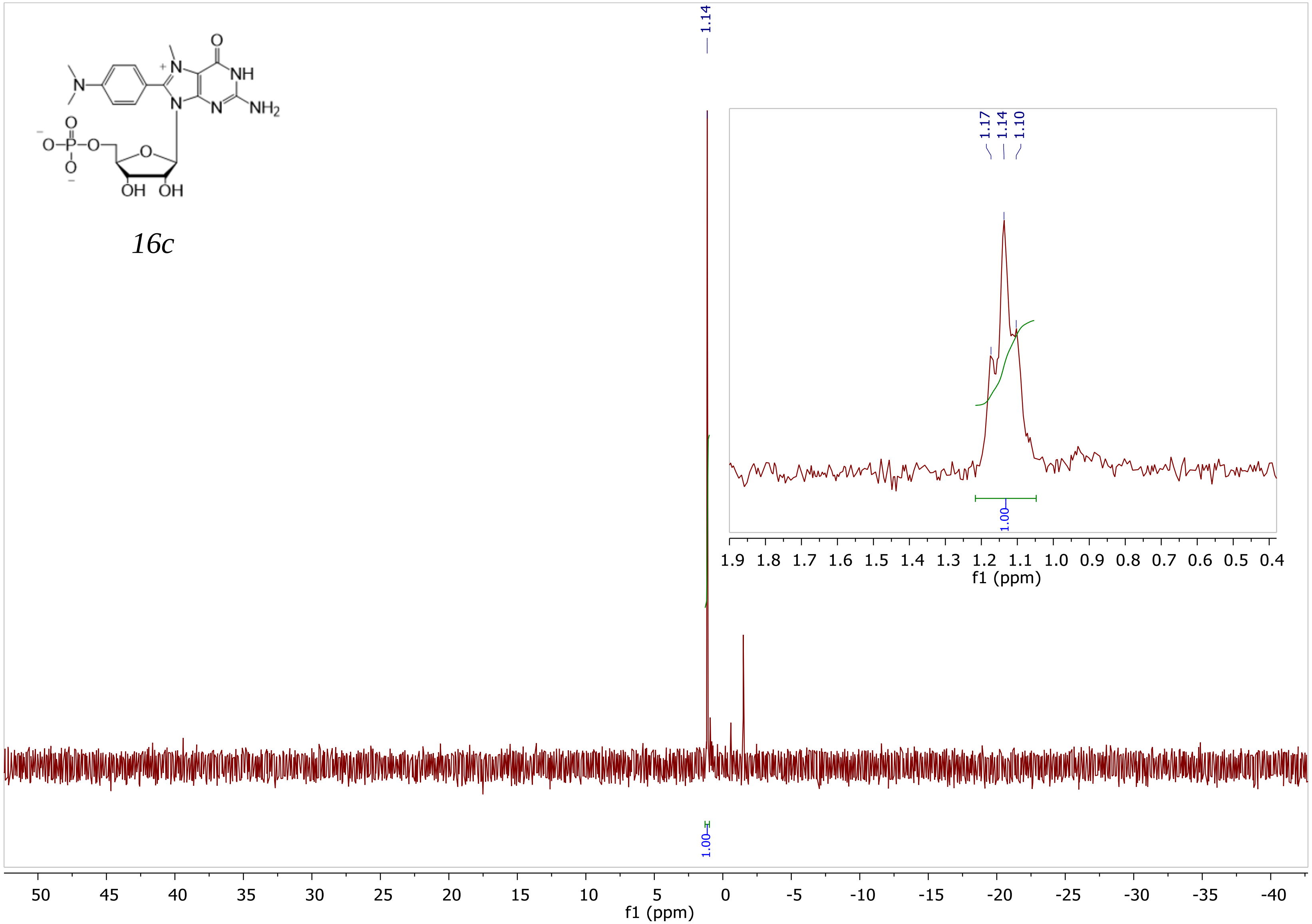

HRMS

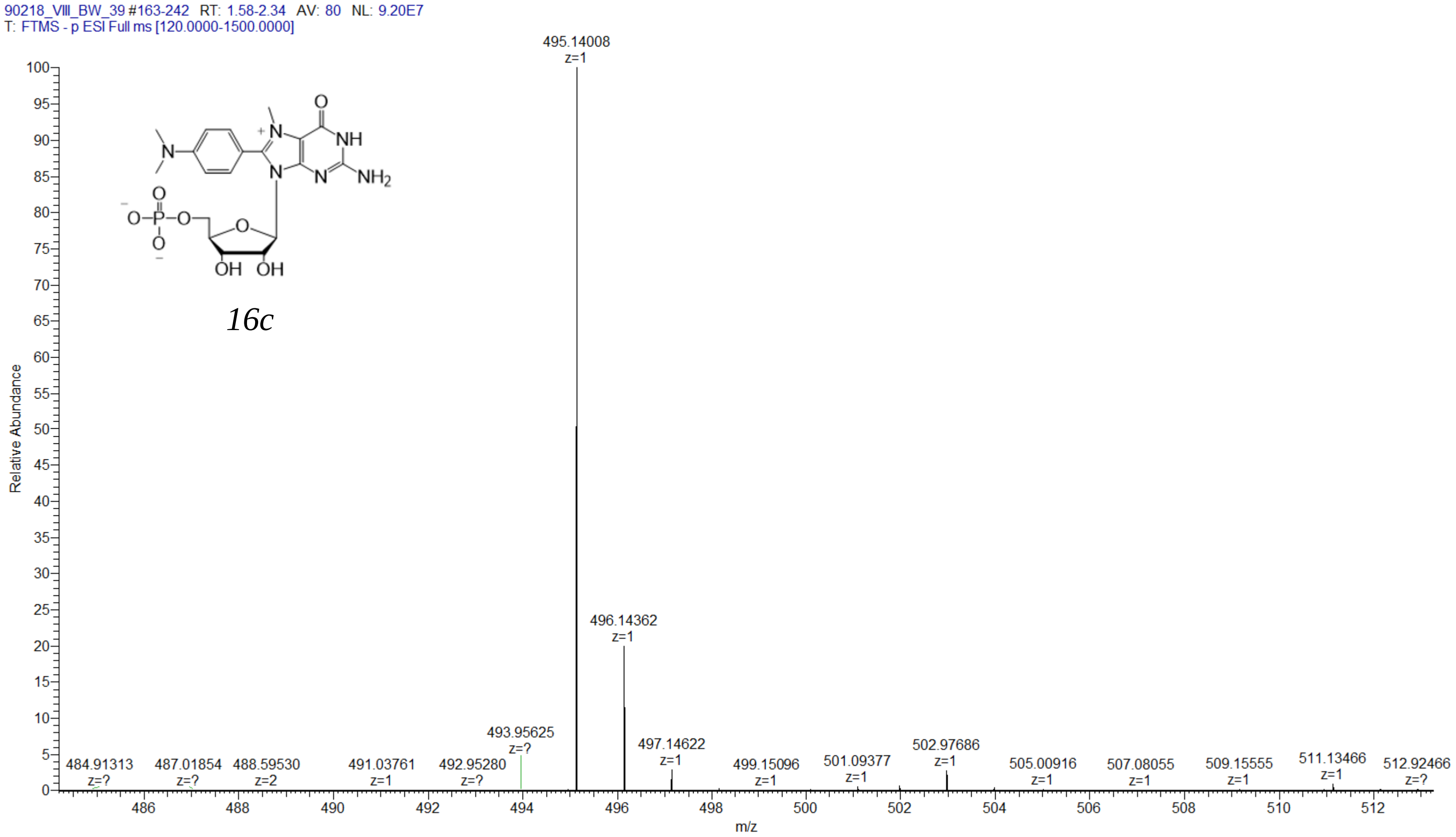

Summary

Rt (D) = 9.41 min; ^{1}H NMR (500 MHz, DMSO-d_6) δ = 7.53 (d, J=9.0 Hz, 2H), 7.04 (d, J=9.0 Hz, 2H), 5.70 (d, J=5.8 Hz, 1H), 5.29 (t, J=5.8 Hz, 1H), 4.54 (dd, J=5.8 Hz, 3.9 Hz, 1H), 4.19 (m, 1H), 4.12 (m, 2H), 3.93 (s, 3H), 3.07 (s, 6H); ^{31}P NMR {1H BB} (162 MHz, Deuterium Oxide) δ = 1.14 (s, *1P*), ^{31}P NMR δ = 1.14 (t, *J*=5.7 Hz, 1P); HRMS ESI (-) m/z [M-H]$^-$, calcd for $C_{19}H_{24}N_6O_8P^-$ 495.1398; found 495.1400.

$m^7GpppG^{8\text{-}Br}$ (1)

Structure

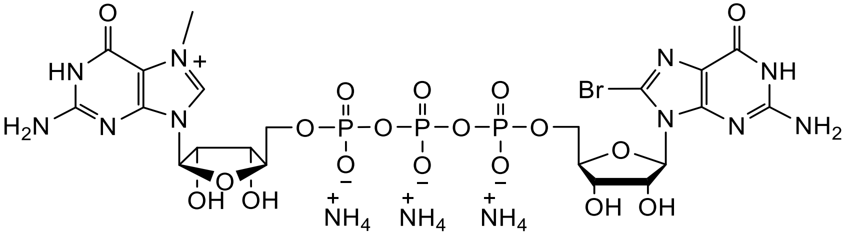

RP-HPLC profile

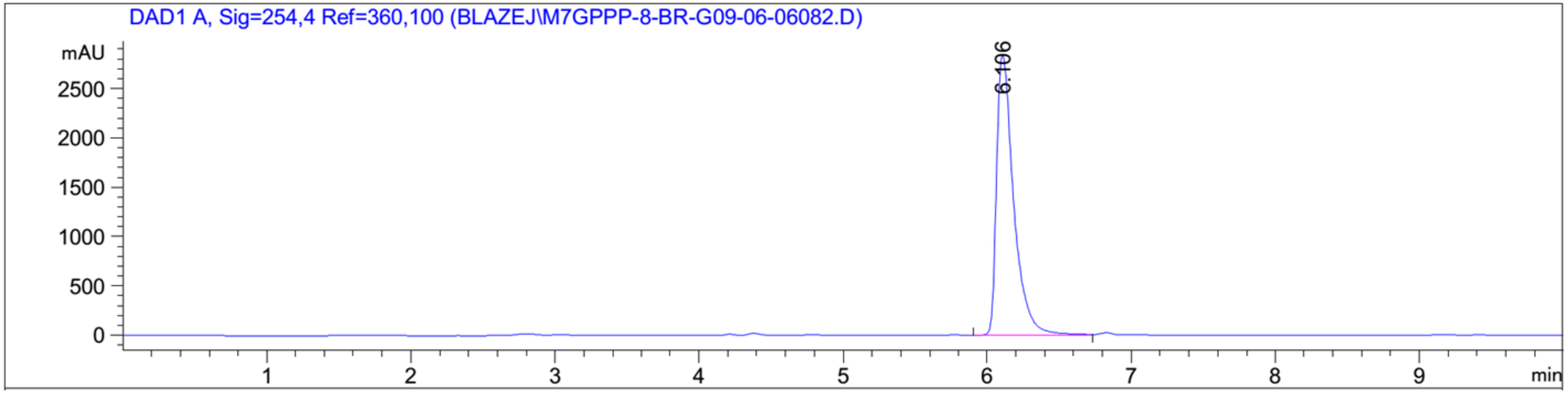

^{1}H NMR

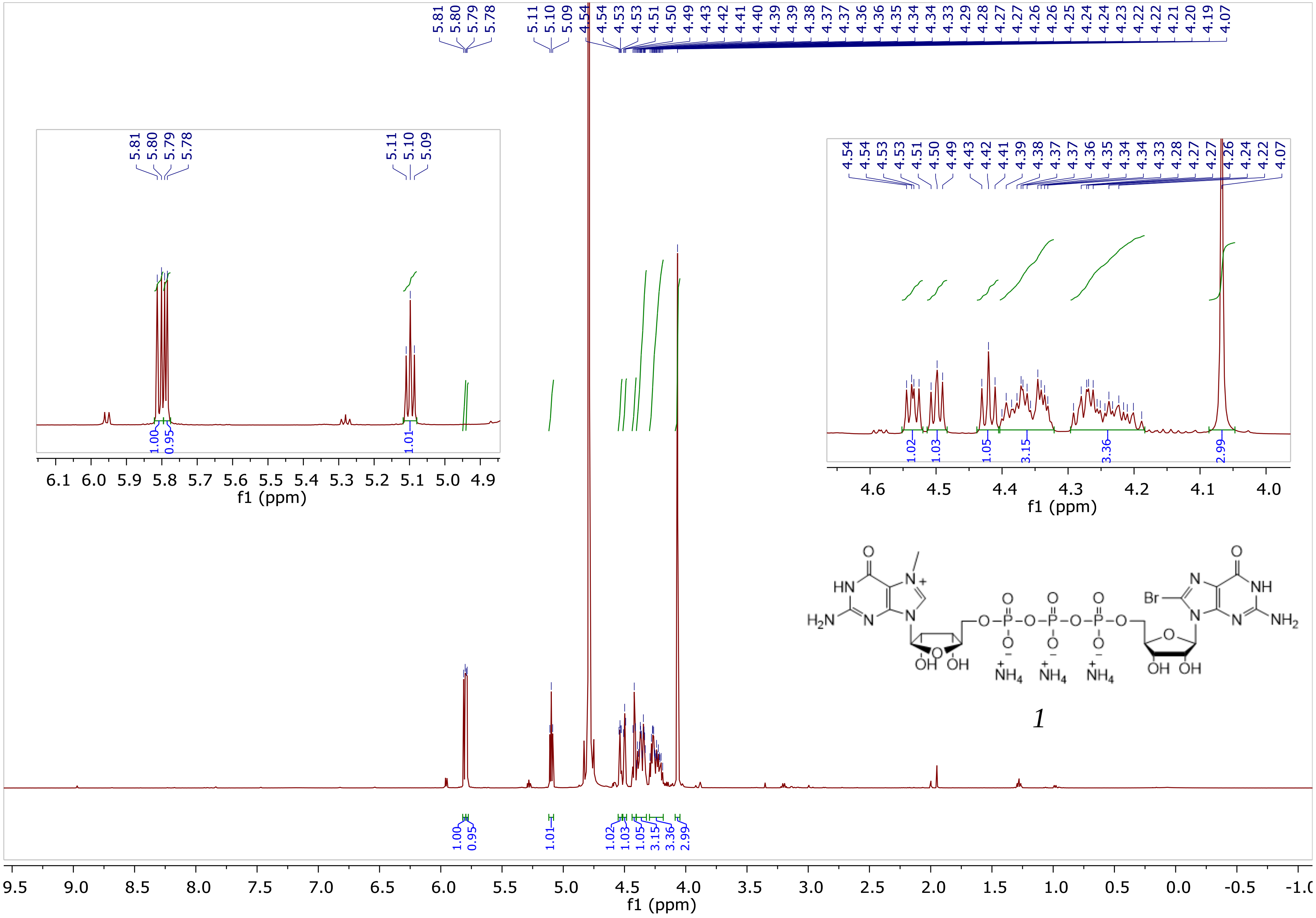

1

^{1}H-^{1}H COSY NMR

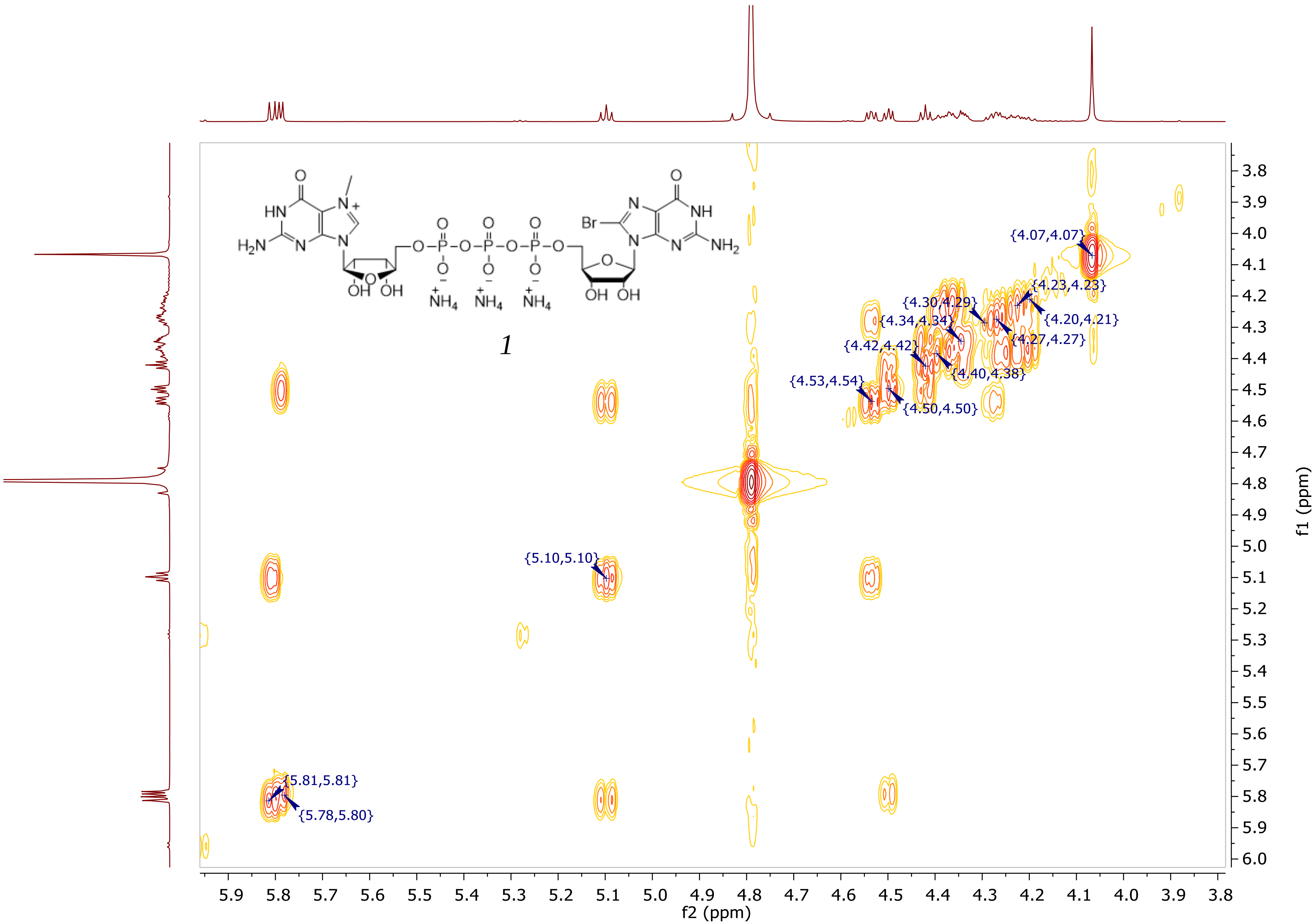
1
{4.07,4.07}
{4.23,4.23}
{4.30,4.29}
{4.20,4.21}
{4.34,4.34}
{4.27,4.27}
{4.42,4.42}
{4.40,4.38}
{4.53,4.54}
{4.50,4.50}
{5.10,5.10}
{5.81,5.81}
{5.78,5.80}
f2 (ppm)
f1 (ppm)

^{31}P NMR

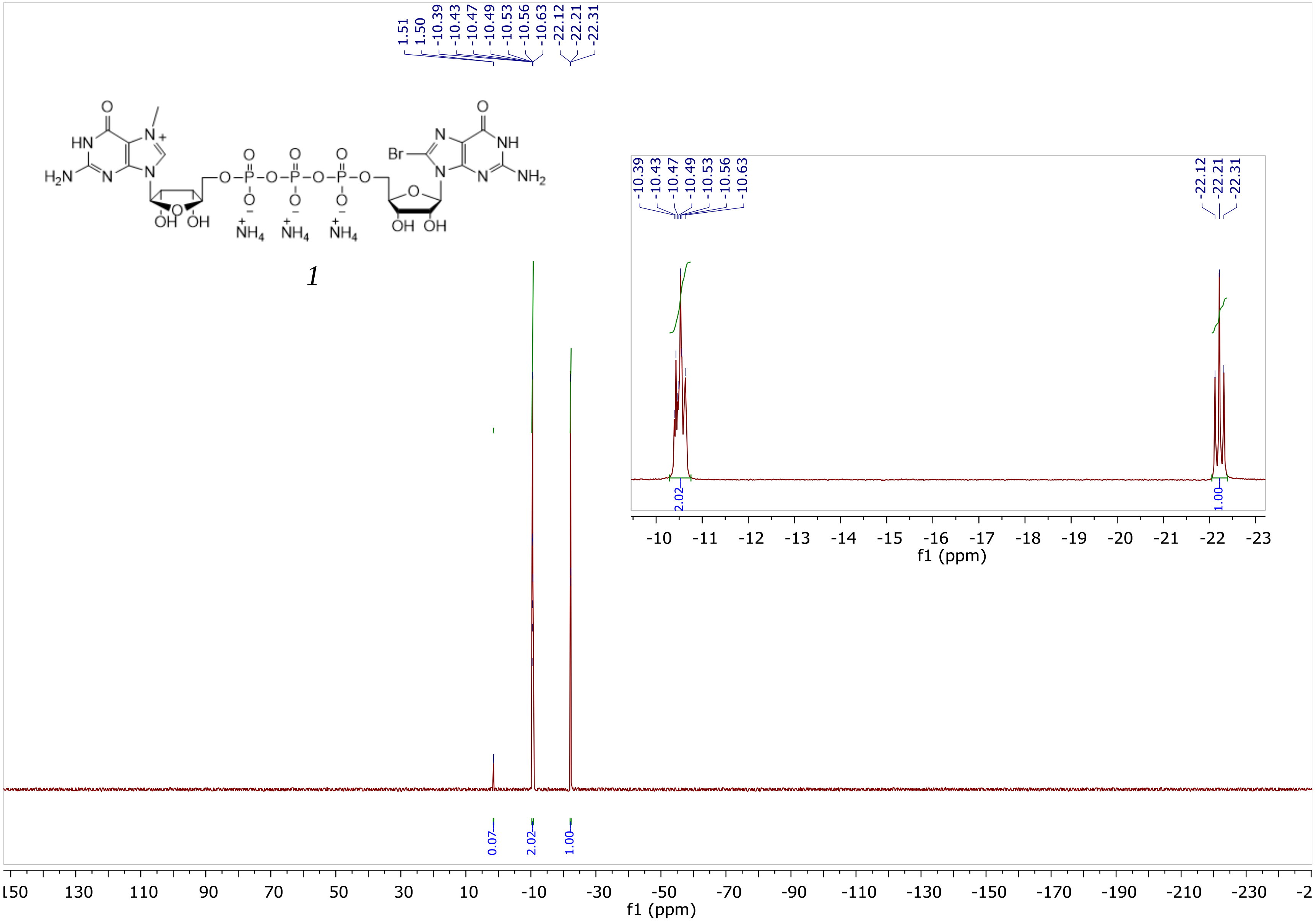
1.51
1.50
-10.39
-10.43
-10.47
-10.49
-10.53
-10.56
-10.63
-22.12
-22.21
-22.31
1
2.02
1.00
0.07
f1 (ppm)

HRMS

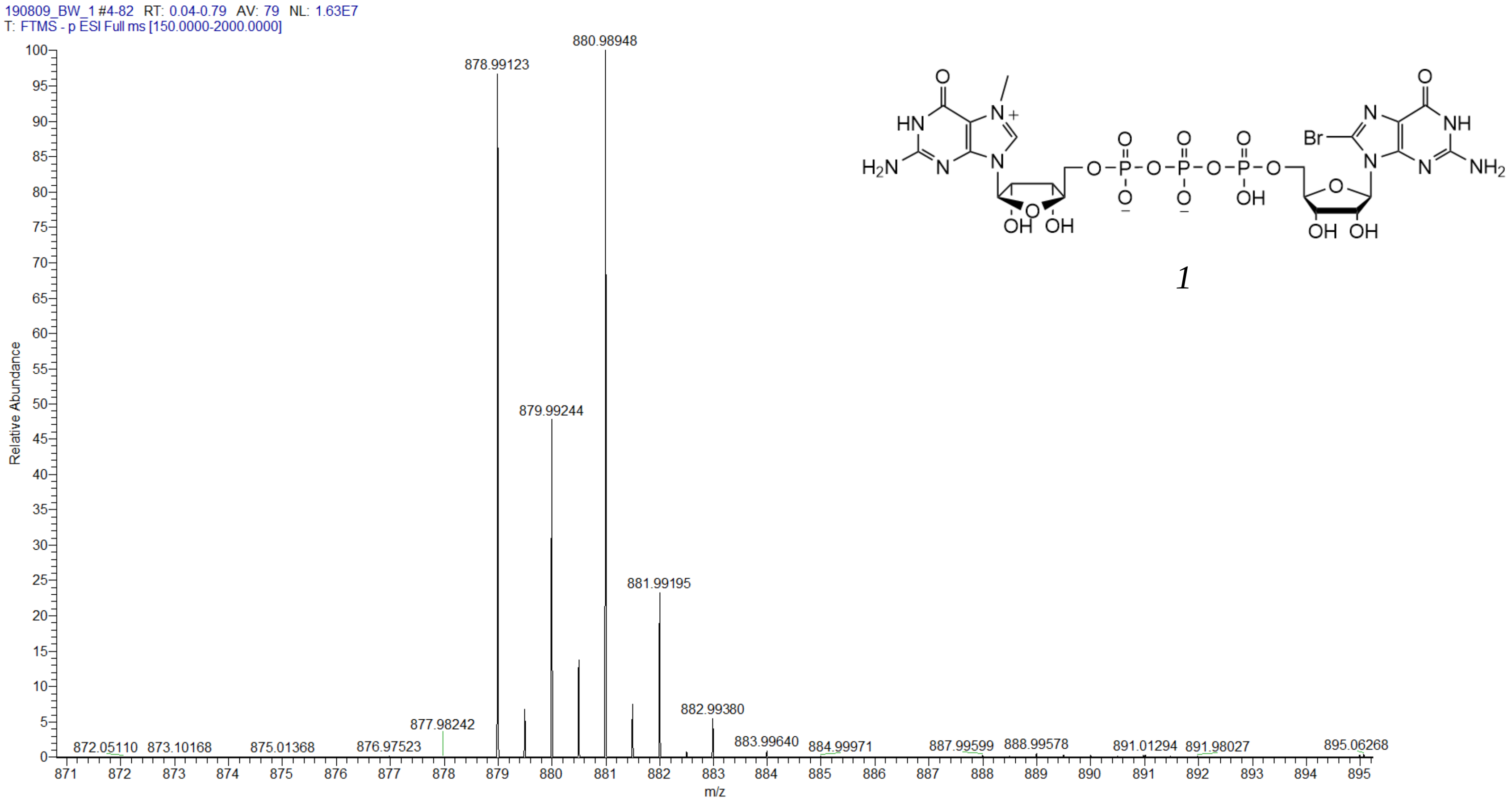

1

Summary

Rt (A) = 6.11 min; ^{1}H NMR (500 MHz, D_2O) δ 5.81 (d, *J* = 6.0 Hz, 1H), 5.79 (d, *J* = 3.9 Hz, 1H), 5.10 (dd, *J* = 6.0 Hz, 5.6 Hz, 1H), 4.54 (dd, *J* = 5.6 Hz, 3.9 Hz, 1H), 4.50 (dd, *J* = 5.0 Hz, 3.9 Hz, 1H), 4.42 (t, *J* = 5.0 Hz, 1H), 4.40–4.32 (m, 3H), 4.30–4.18 (m, 3H), 4.07 (s, 3H); ^{31}P NMR (202 MHz, D_2O) δ –10.63 to –10.39 (m, 2P), –22.21 (t, *J* = 19.4 Hz, 1P); HRMS ESI (-) m/z [M-H]$^-$, calcd for $C_{21}H_{27}BrN_{10}O_{18}P_3^-$ [M-H]$^-$ 878.9906, 880.9886; found 878.9920, 879.9917.

$m^{2'O,7}GpppG^{8Br}$ (2)

Structure

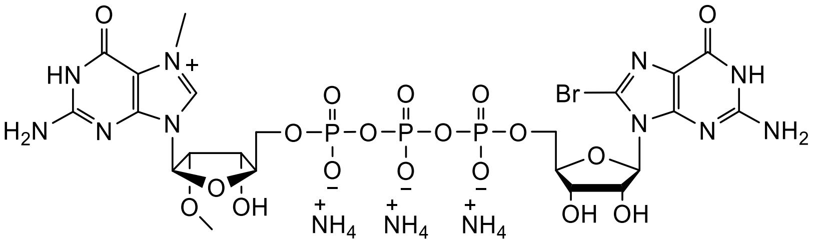

RP-HPLC profile

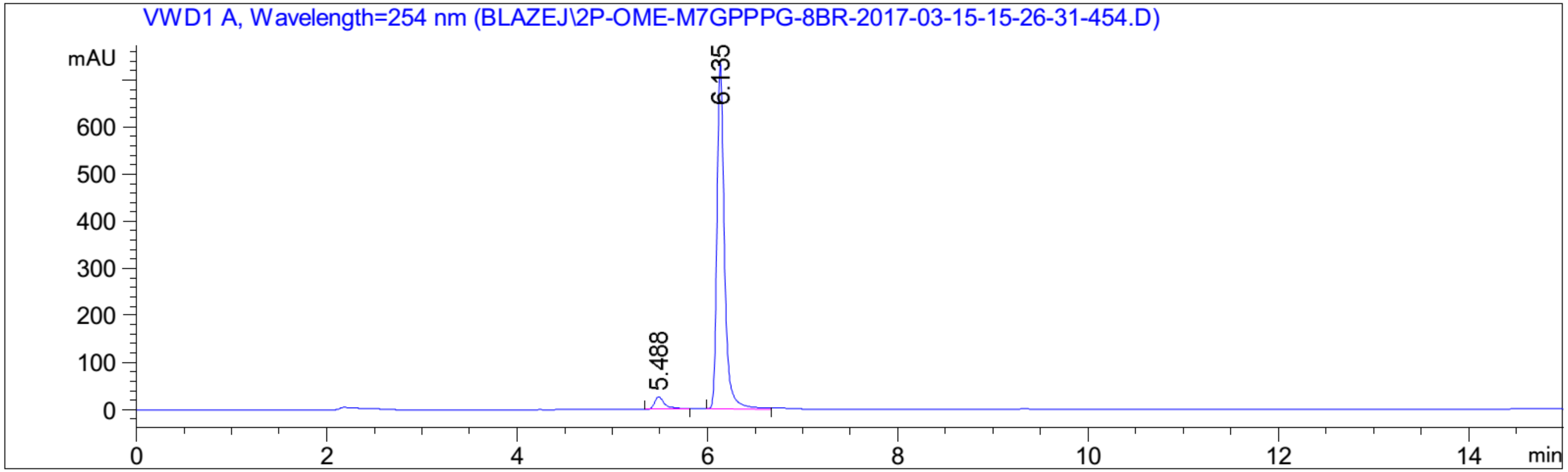

^{1}H NMR

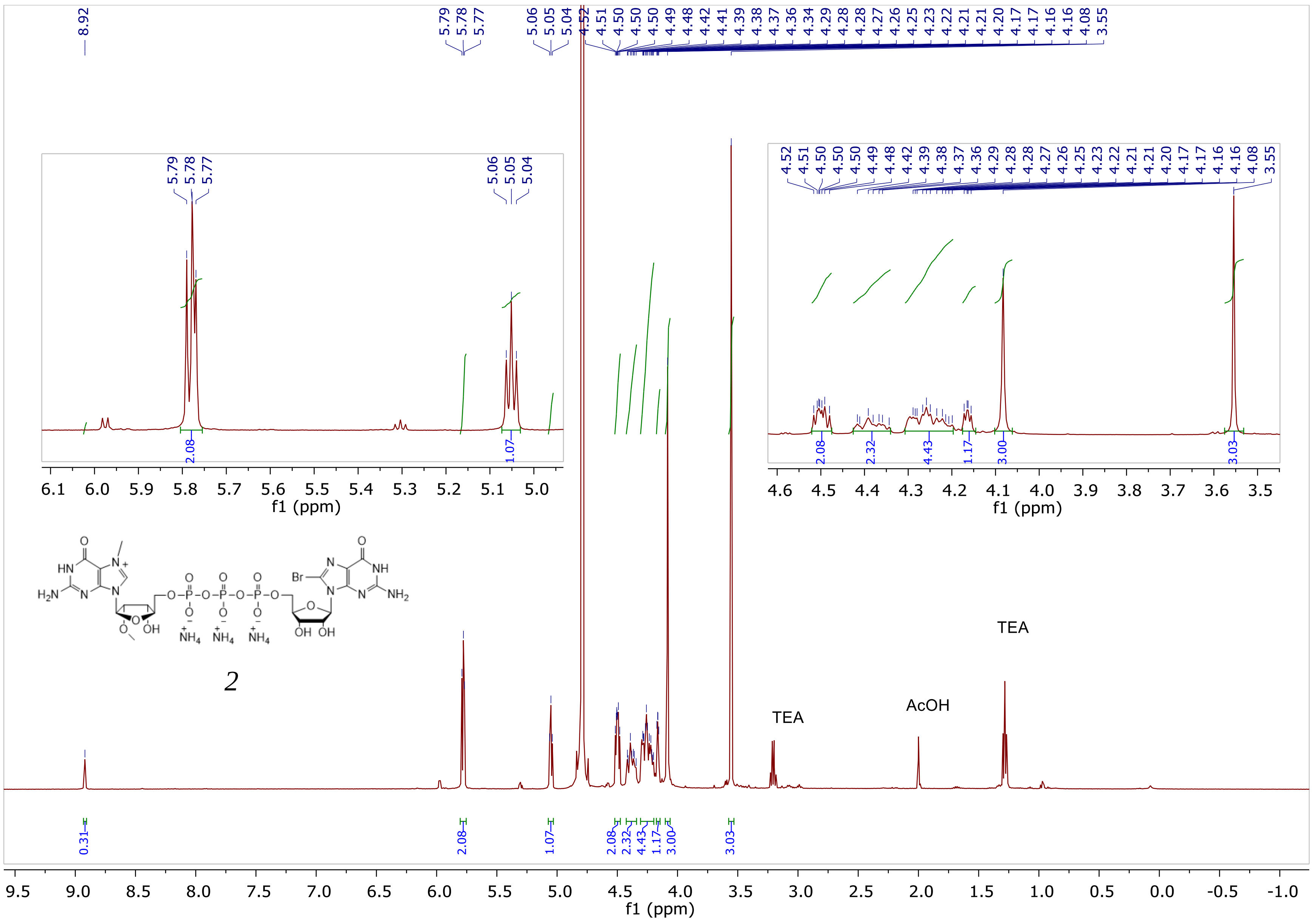

^{31}P NMR

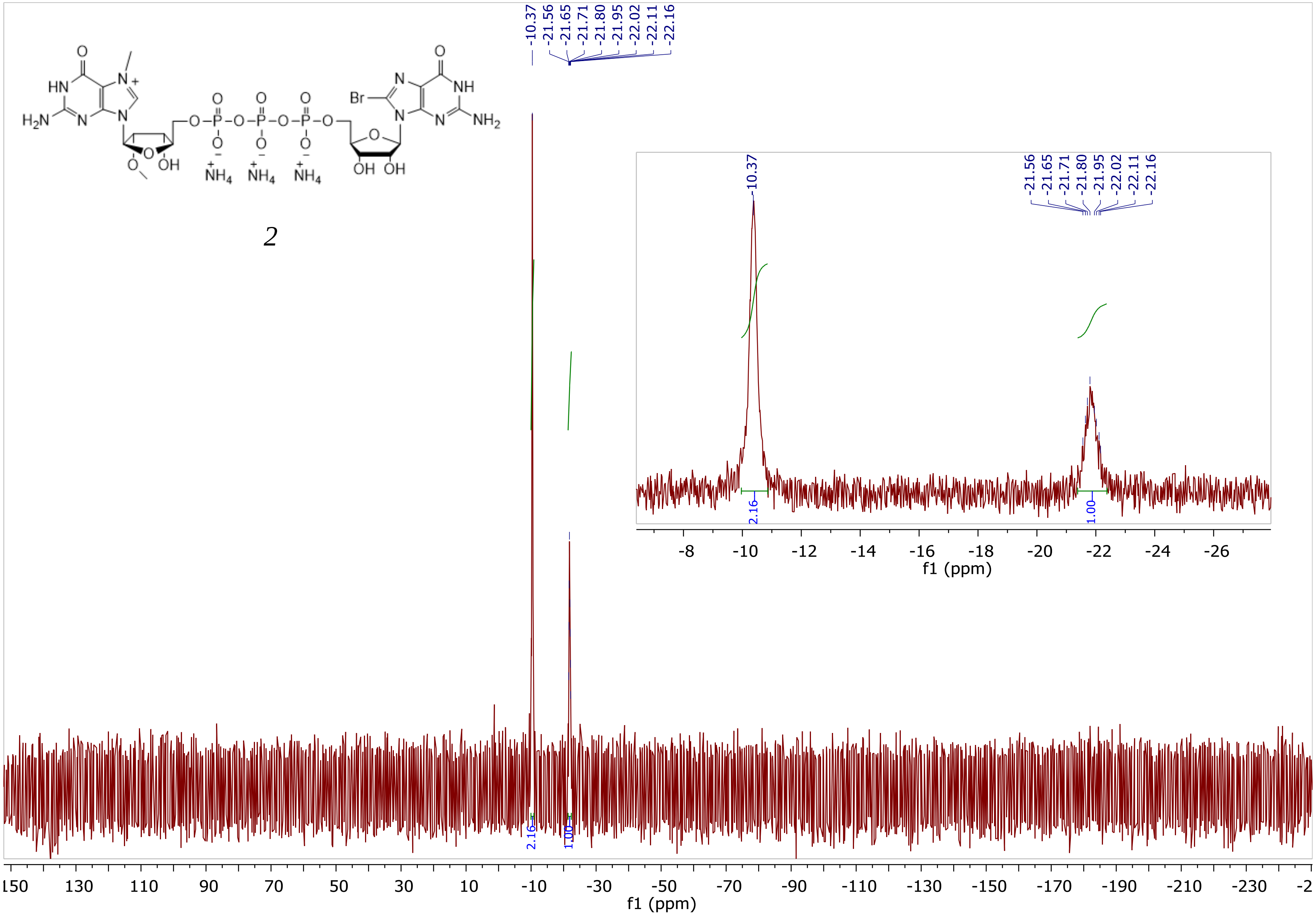

2

HRMS

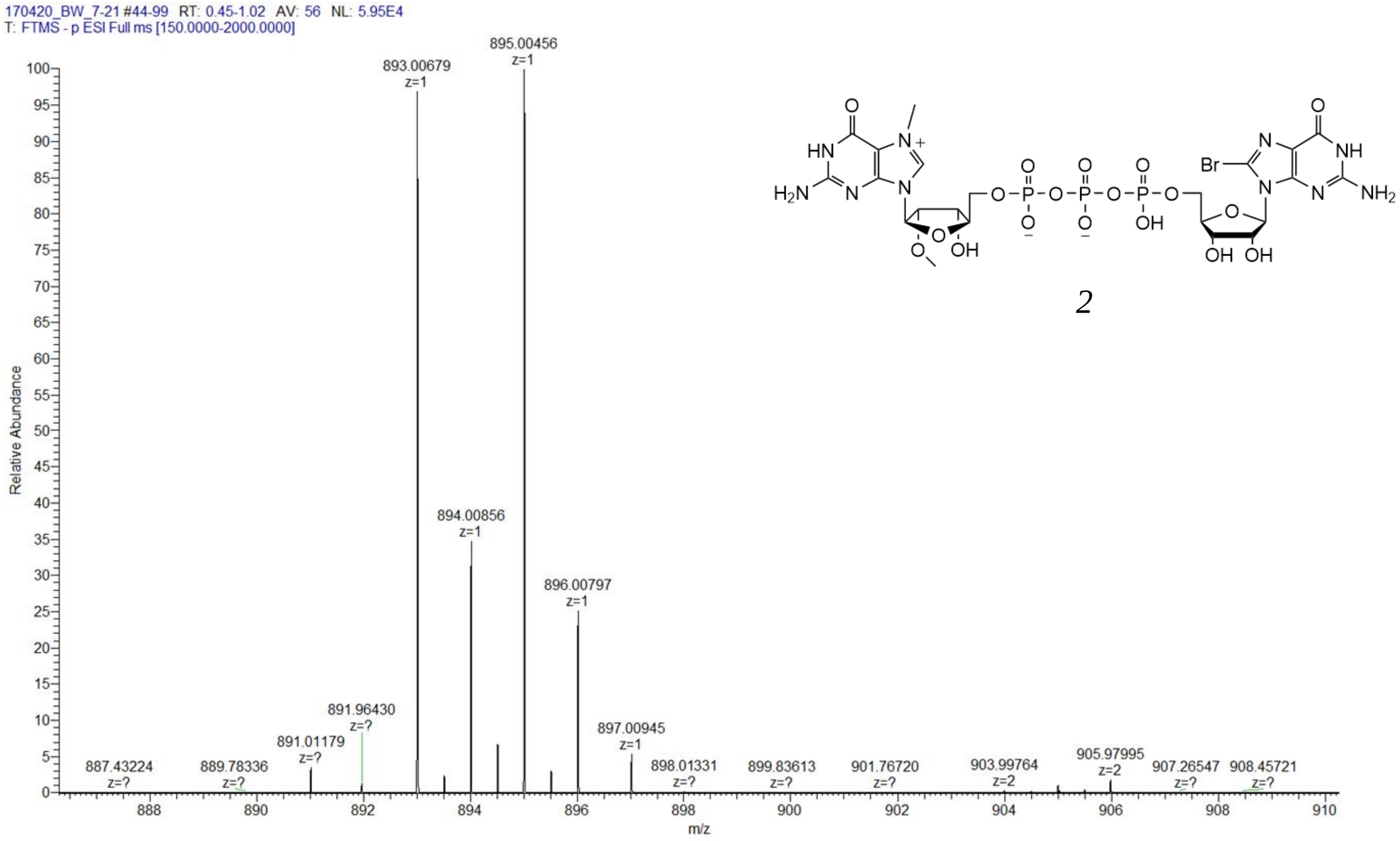

2

Summary

: Rt (A) = 6.13 min; ^{1}H NMR (500 MHz, deuterium oxide) δ 8.92 (s, 1H), 5.78 (m, 2H), 5.05 (t, *J* = 5.8 Hz, 1H), 4.50 (m, 2H), 4.38 (m, 2H), 4.25 (m, 4H), 4.16 (m, 1H), 4.08 (s, 3H), 3.55 (s, 3H); ^{31}P NMR (202 MHz, deuterium oxide) δ –10.37 (m, 2P), –22.16 to –21.56 (m, 1P); HRMS ESI (-) m/z [M-H]$^{-}$, calcd. *m/z* for $C_{22}H_{29}BrN_{10}O_{18}P_3^{-}$ [M-H]$^{-}$ 893.0063, 895.0043; found 893.0068, 895.0046.

m^{7}GpppG^{8Py} (3a)

Structure

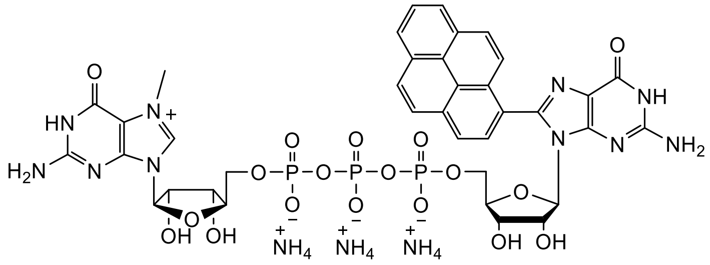

RP-HPLC profile

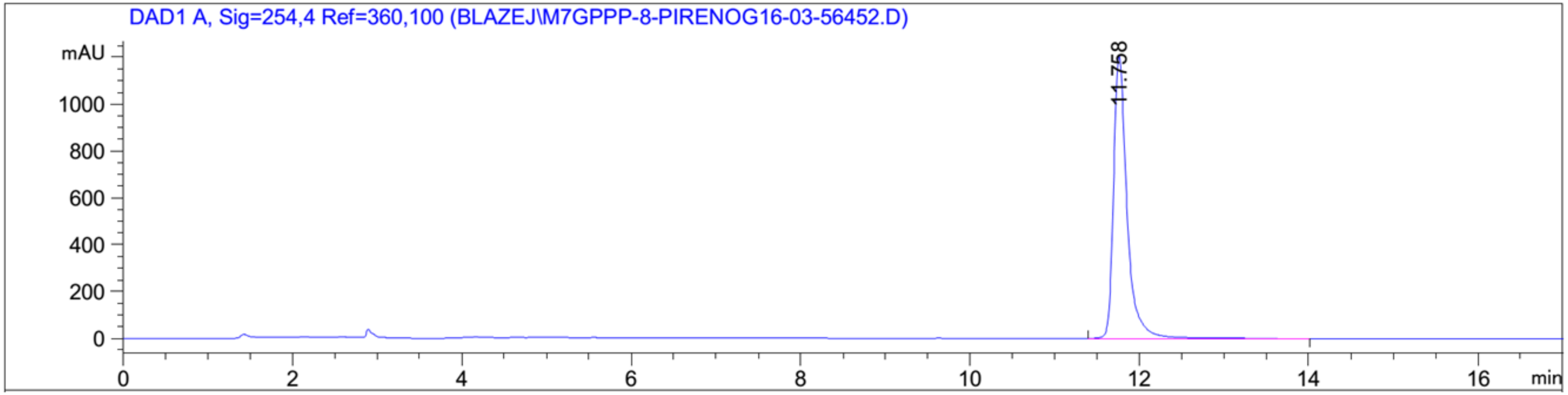

^{1}H NMR

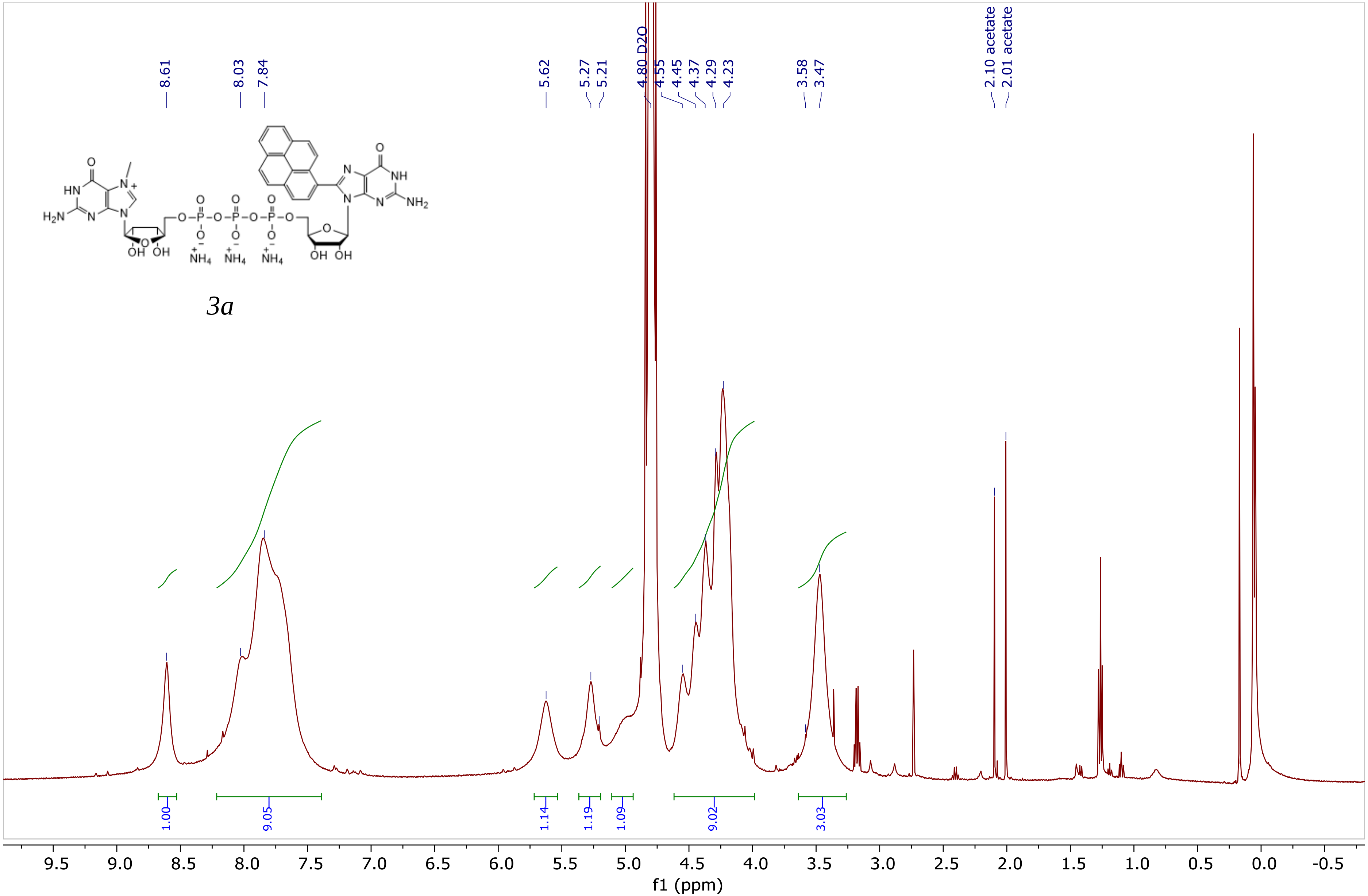

8.61
8.03
7.84
5.62
5.27
5.21
4.80 D2O
4.55
4.45
4.37
4.29
4.23
3.58
3.47
2.10 acetate
2.01 acetate
1.00
9.05
1.14
1.19
1.09
9.02
3.03
3a
9.5 9.0 8.5 8.0 7.5 7.0 6.5 6.0 5.5 5.0 4.5 4.0 3.5 3.0 2.5 2.0 1.5 1.0 0.5 0.0 -0.5
f1 (ppm)

^{31}P NMR

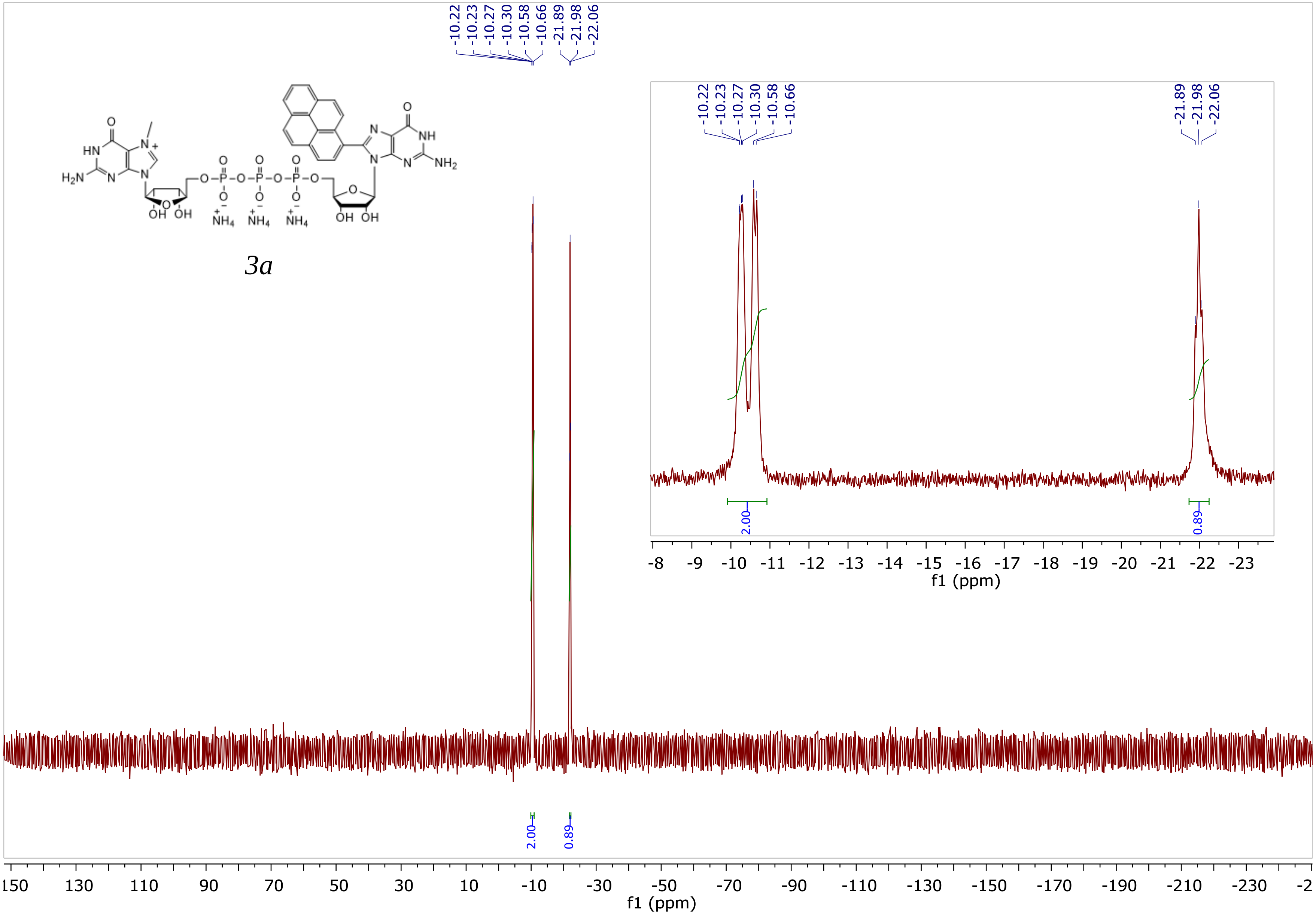

3a

HRMS

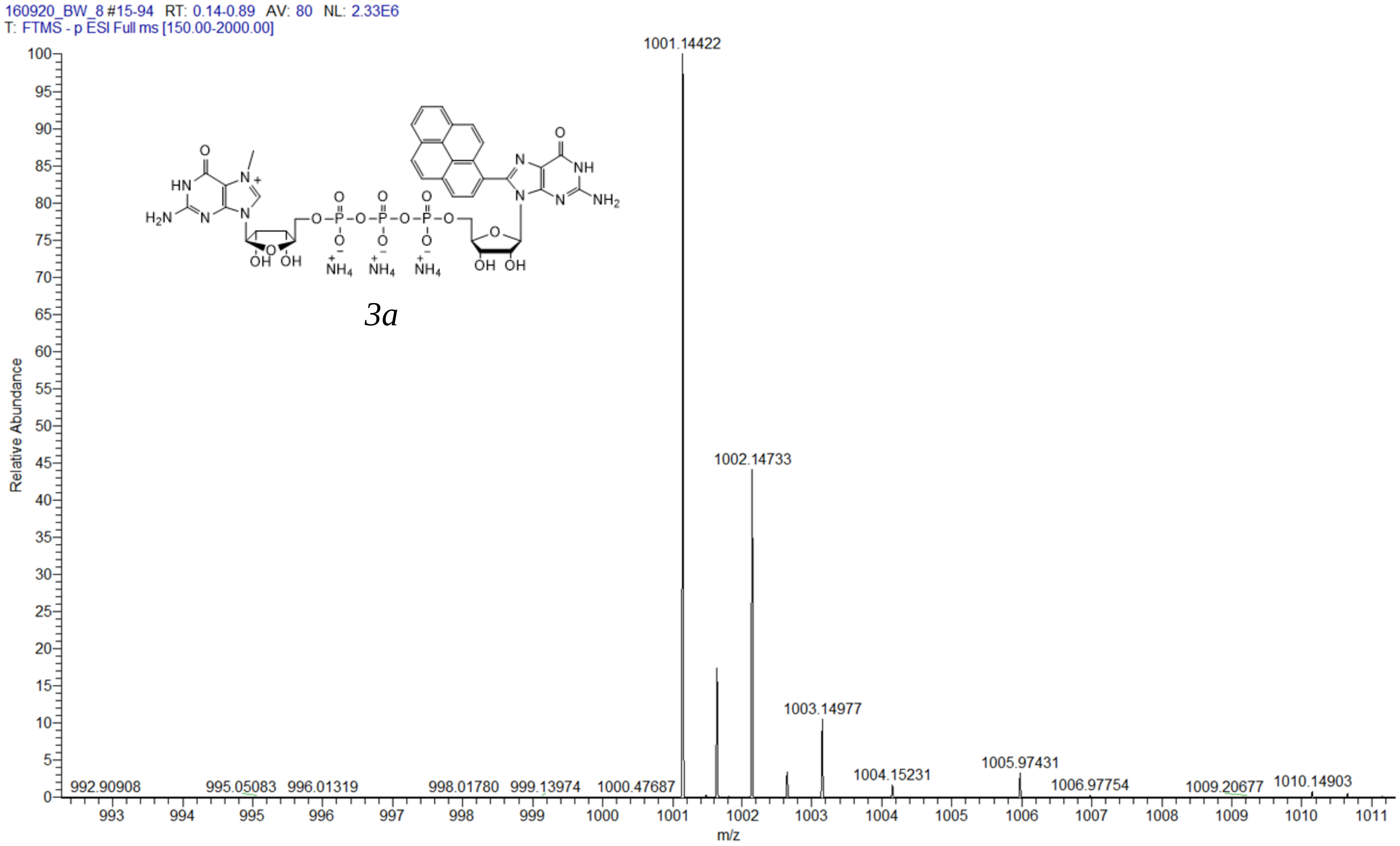

Summary

Rt (C) = 11.76 min; ^{1}H NMR (500 MHz, deuterium oxide) δ 8.61 (s, 1H), 8.22–7.39 (m, 9H), 5.62 (s, 1H), 5.24 (s, 1H), 5.11–4.92 (m, 1H, overlapped with signal from HDO), 4.66–3.96 (m, 9H), 3.62–3.32 (m, 3H); ^{31}P NMR (202 MHz, deuterium oxide) δ –10.12 to –10.76 (m, 2P), –21.80 to –22.16 (m, 1P); HRMS ESI (-) m/z [M-H]$^{-}$, calcd for $C_{37}H_{36}N_{10}O_{18}P_{3}^{-}$ [M-H]$^{-}$ 1001.1427; found 1001.1442.

m⁷GpppG⁸ᴾʰ (3b)

Structure

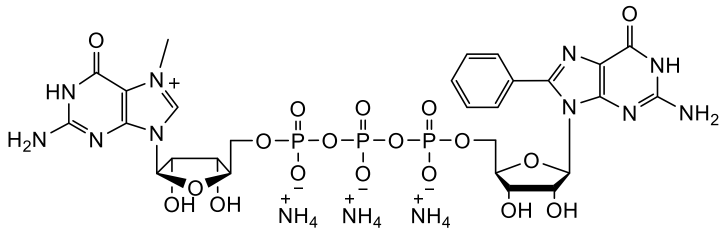

RP-HPLC profile

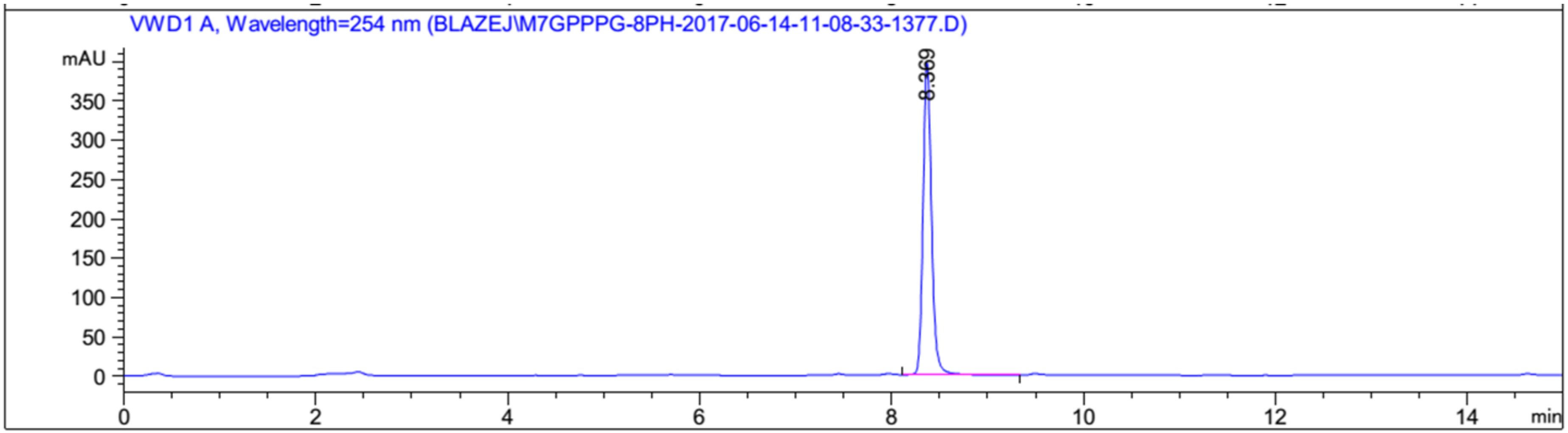

^{1}H NMR

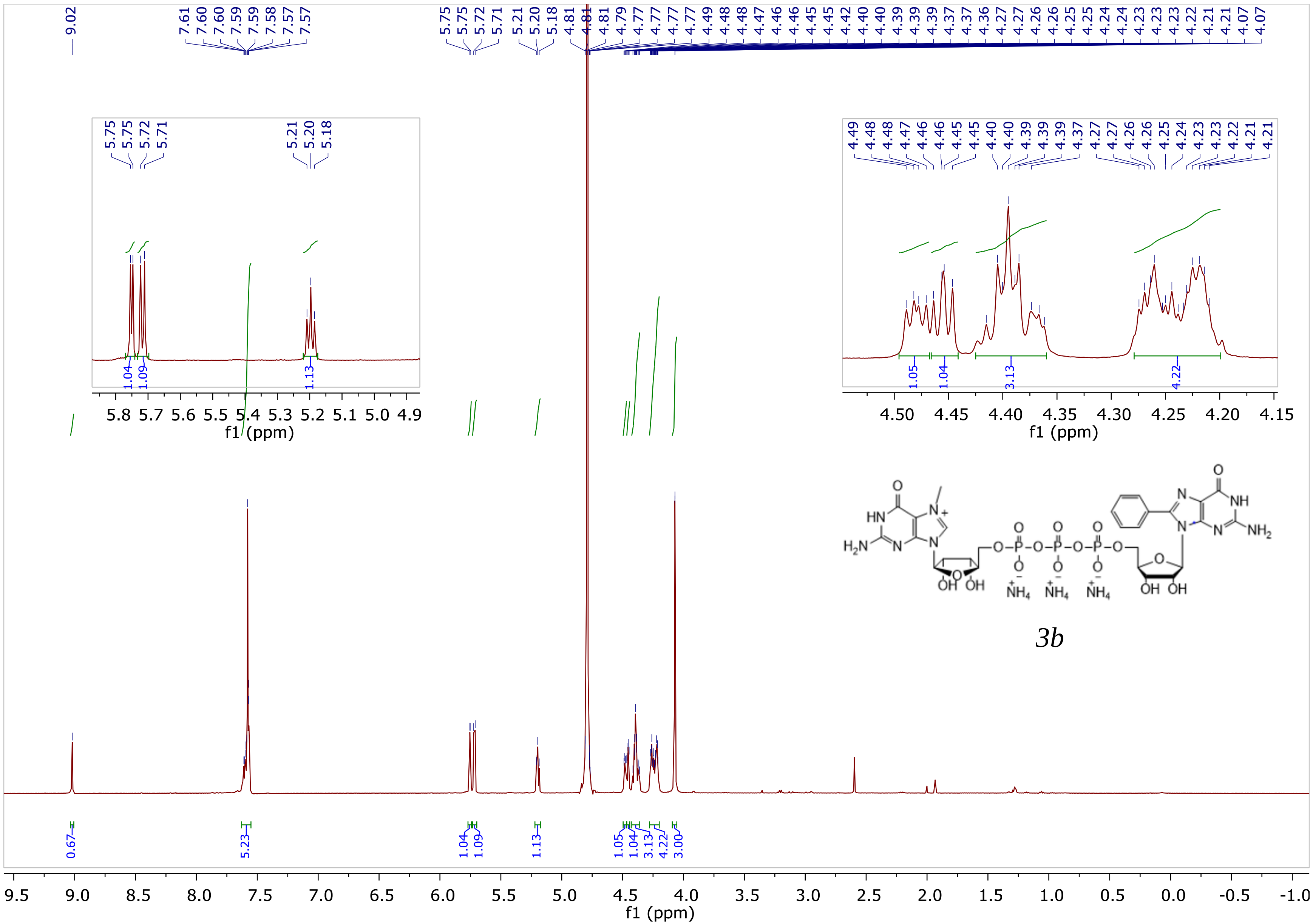

3b

^1H-^1H COSY NMR

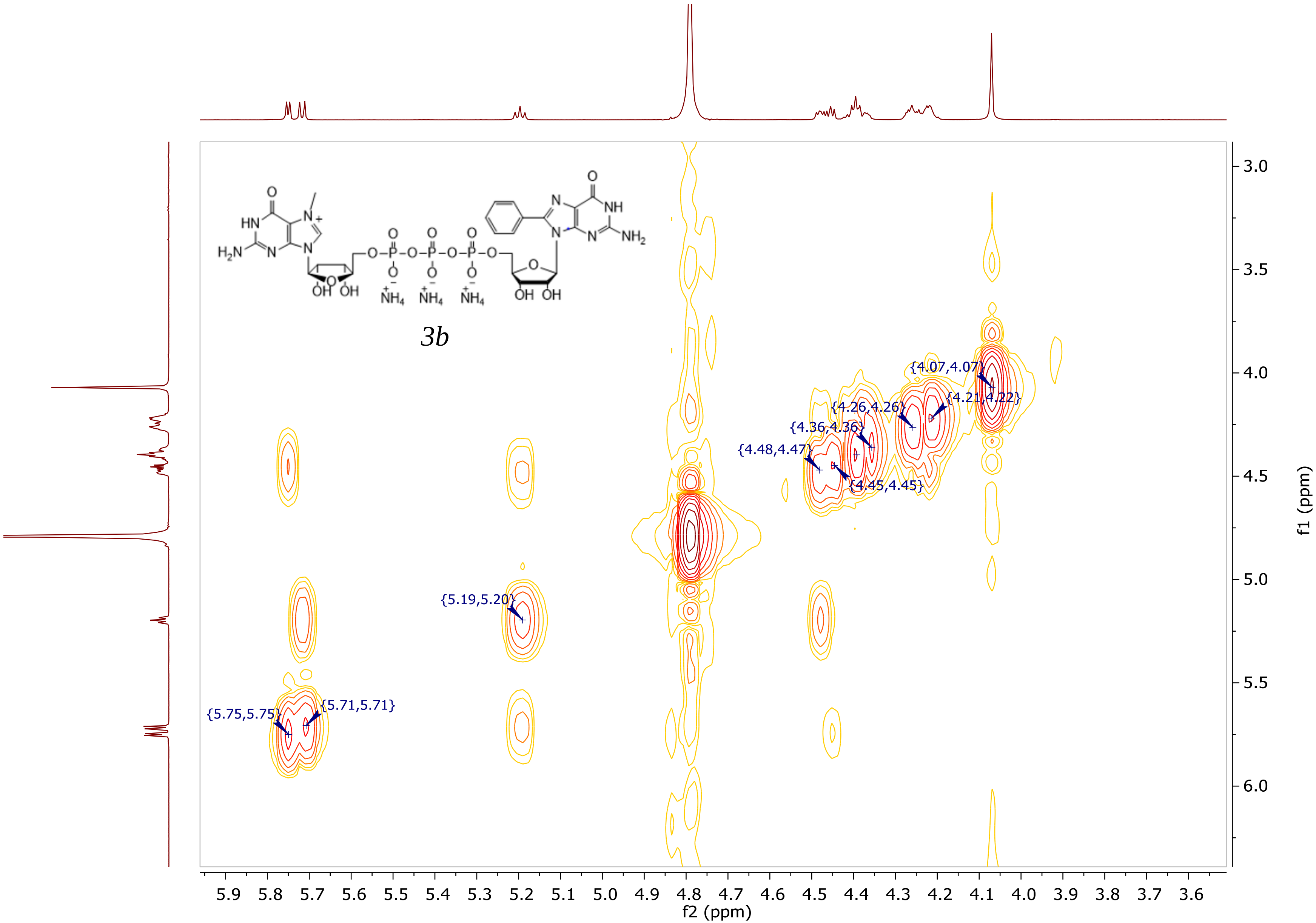

^{31}P NMR

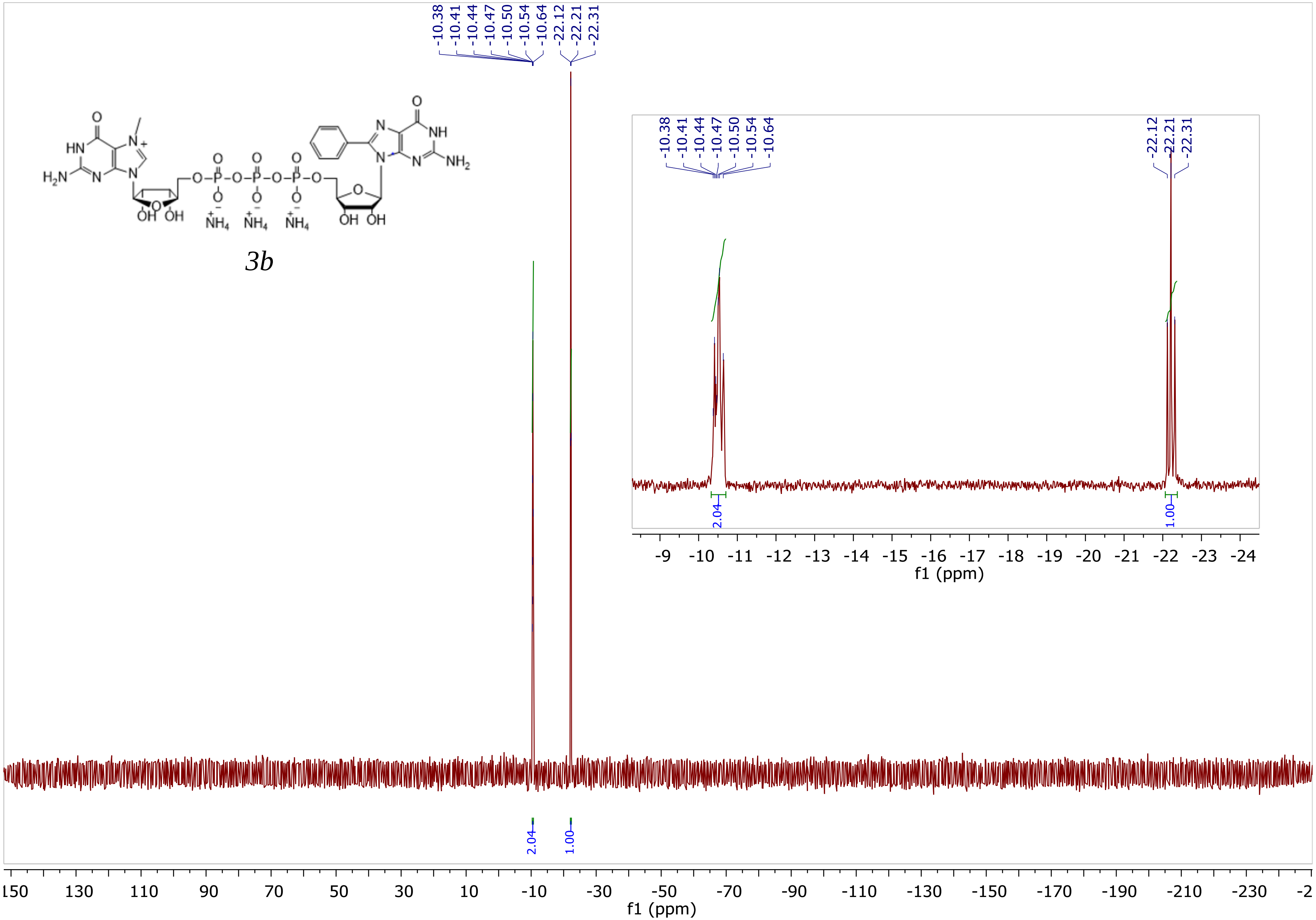
-10.38
-10.41
-10.44
-10.47
-10.50
-10.54
-10.64
-22.12
-22.21
-22.31
3b
2.04
1.00
-9 -10 -11 -12 -13 -14 -15 -16 -17 -18 -19 -20 -21 -22 -23 -24
f1 (ppm)
130 110 90 70 50 30 10 -10 -30 -50 -70 -90 -110 -130 -150 -170 -190 -210 -230
f1 (ppm)

HRMS

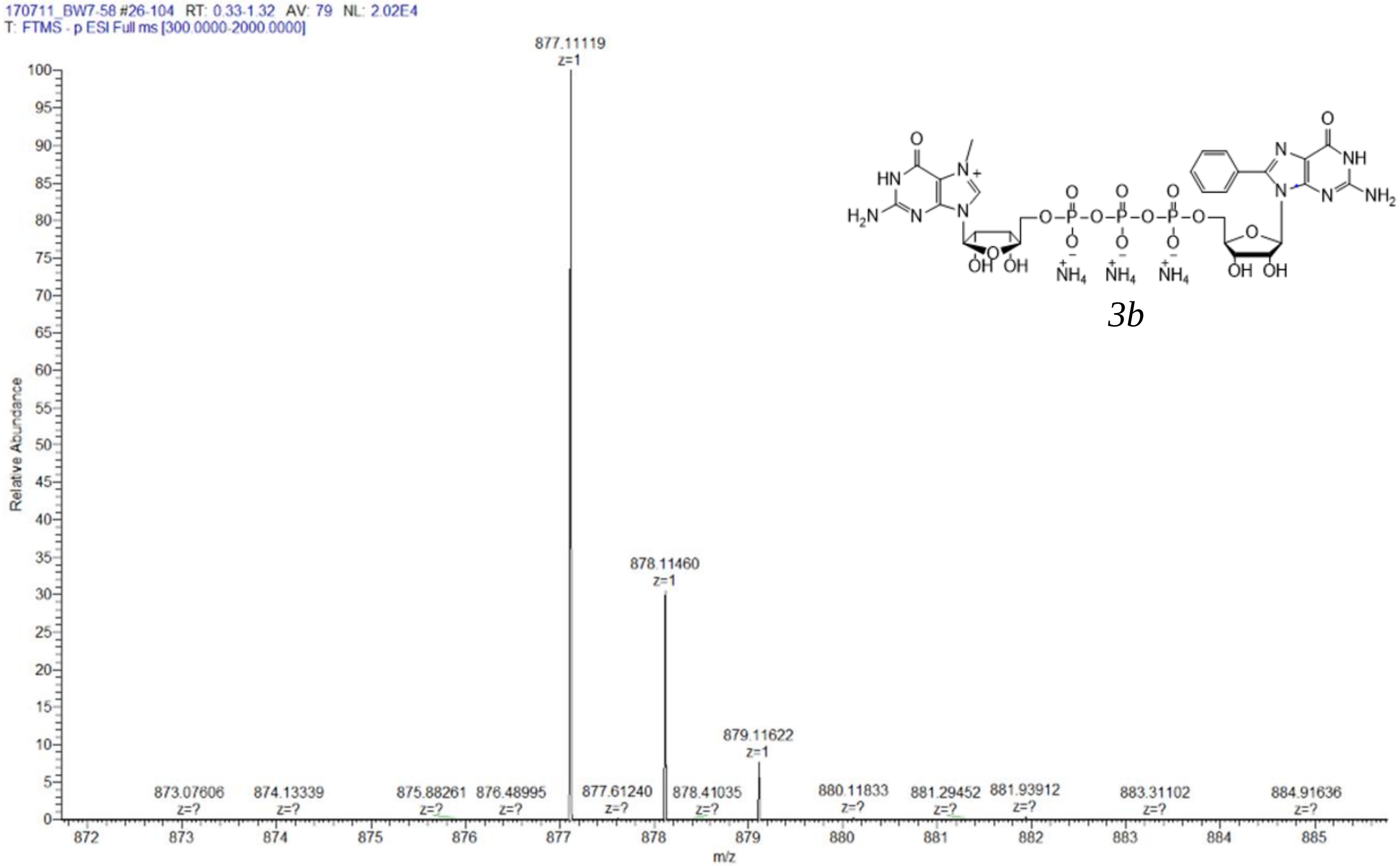

3b

Summary

Rt (A) = 8.37 min; ^{1}H NMR (500 MHz, deuterium oxide) δ 9.02 (s, 1H), 7.57 (m, 5H), 5.75 (d, *J* = 3.9 Hz, 1H), 5.72 (d, *J* = 6.1 Hz, 1H), 5.20 (dd, *J* = 6.1 Hz, 5.7 Hz, 1H), 4.48 (dd, *J* = 5.7 Hz, 3.5 Hz, 1H), 4.46 (dd, *J* = 4.9 Hz, 3.9 Hz, 1H), 4.39 (m, 3H), 4.24 (m, 4H), 4.07 (m, 3H); ^{31}P NMR (202 MHz, deuterium oxide) δ –10.30 to –10.70 (m, 2P), –22.21 (t, *J* = 19.3 Hz, 1P); HRMS ESI (-) m/z [M-H]$^{-}$, calcd for $C_{27}H_{32}N_{10}O_{18}P_3^{-}$ [M-H]$^{-}$ 877.1114; found 877.1112.

m^7GpppG^{8DMAPh} (3c)

Structure

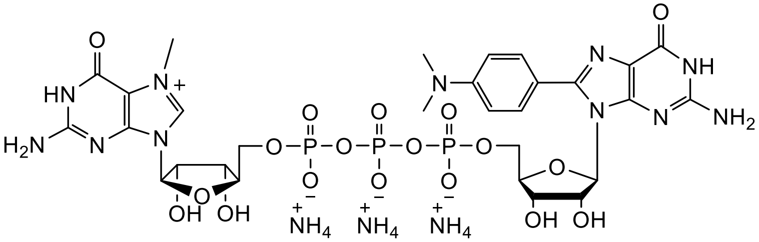

RP-HPLC profile

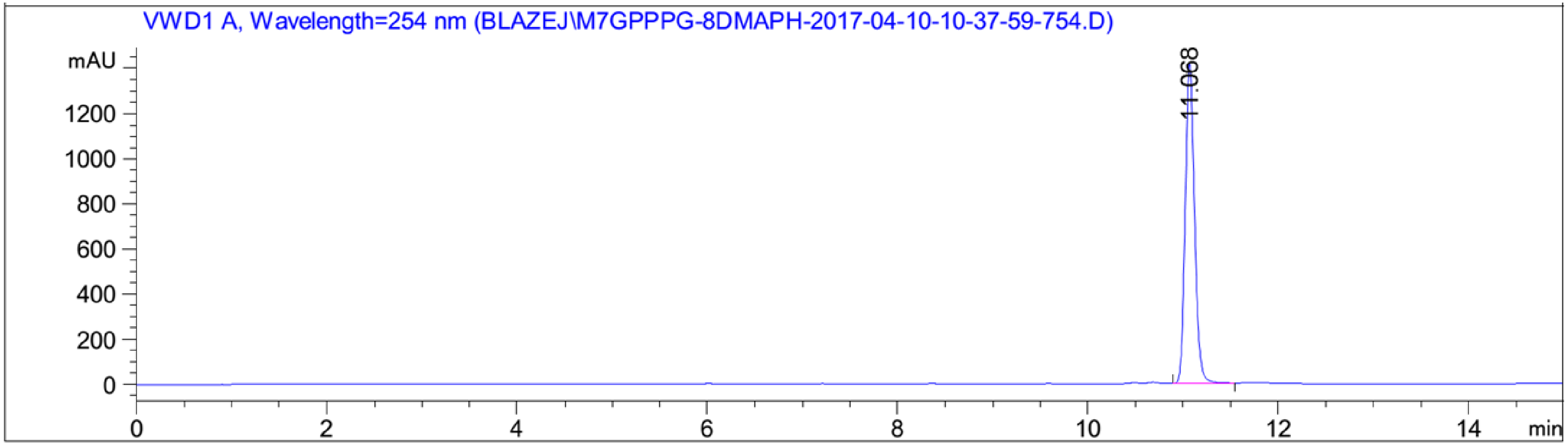

^{1}H NMR

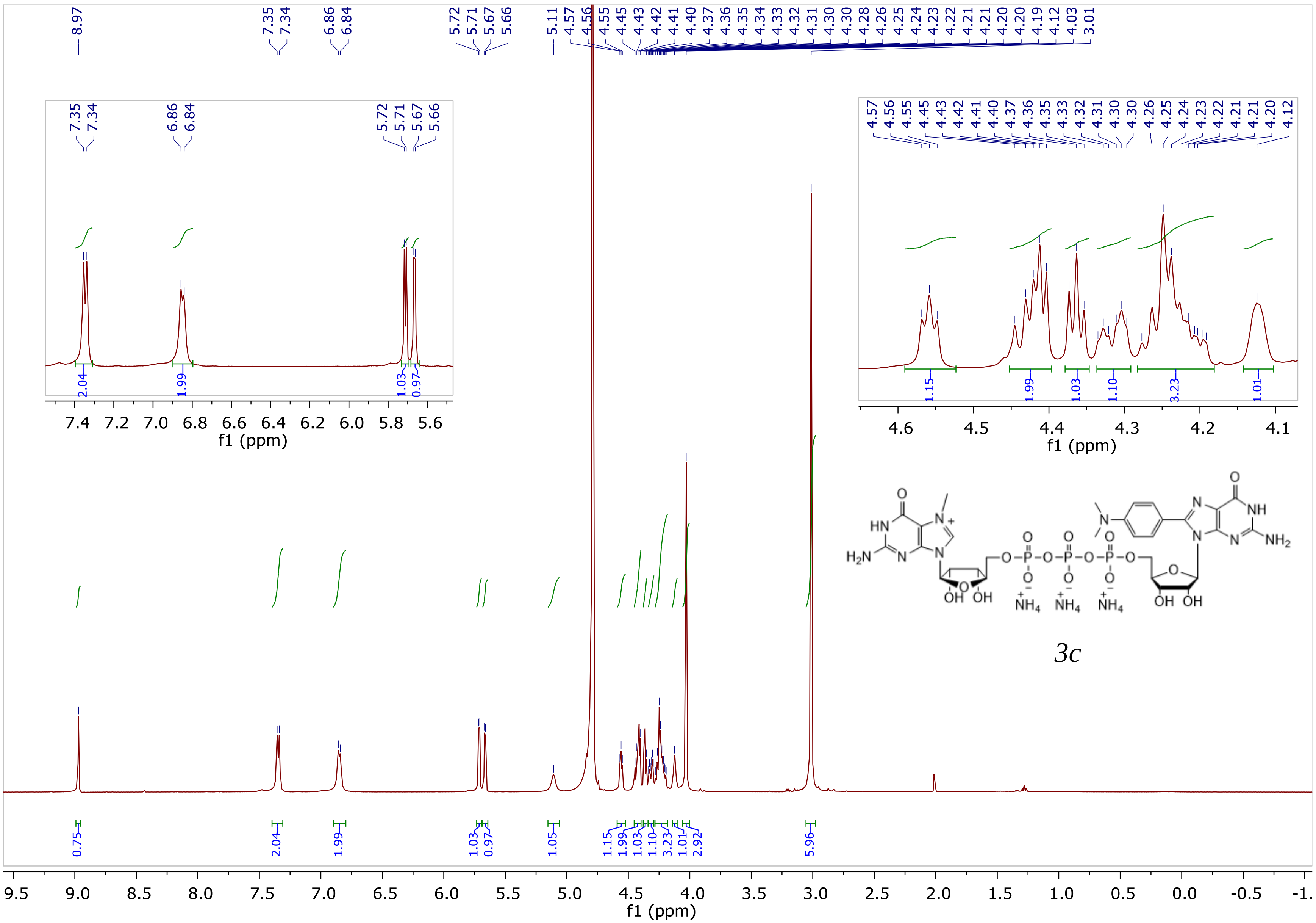

3c

^{31}P NMR

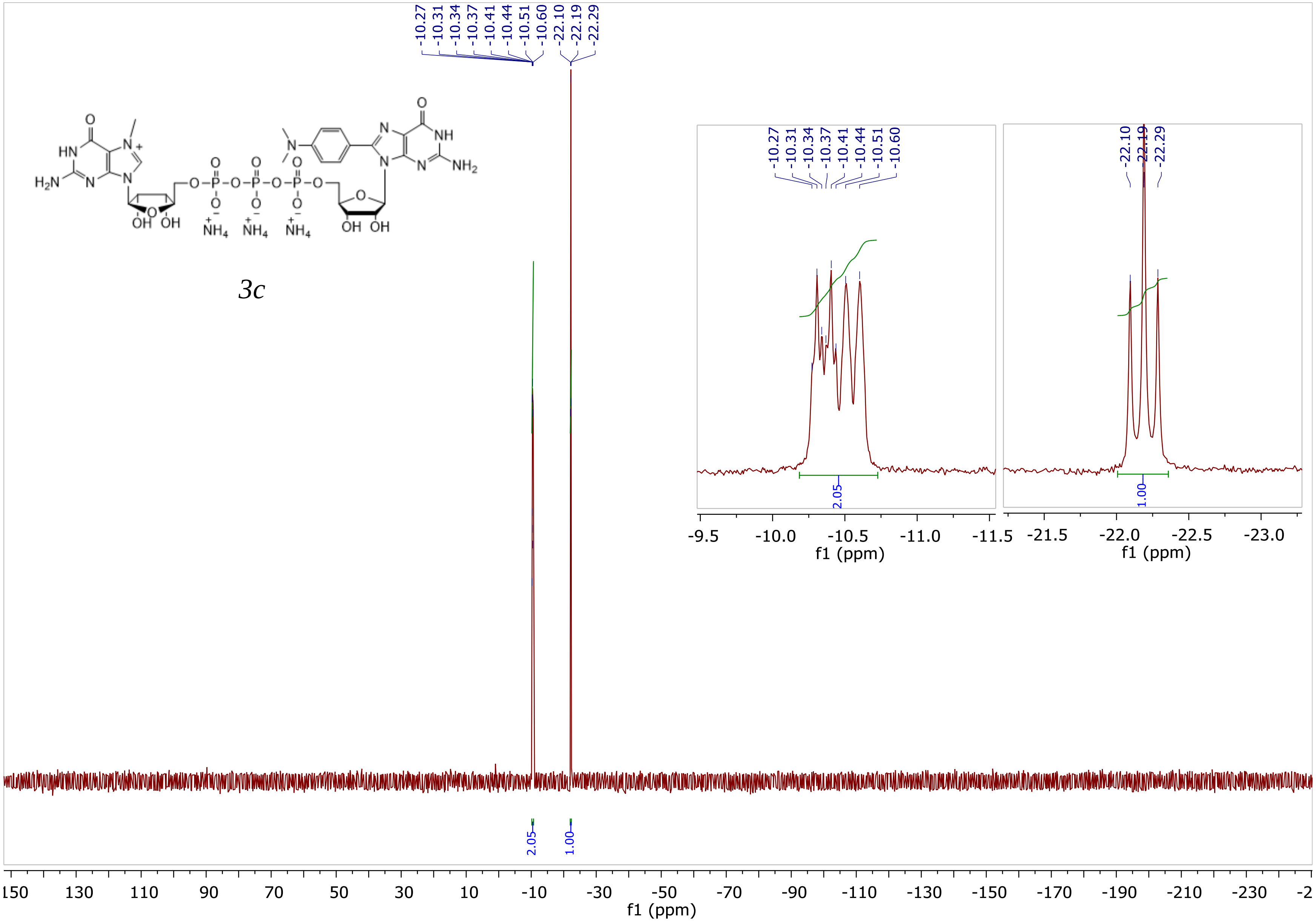
-10.27
-10.31
-10.34
-10.37
-10.41
-10.44
-10.51
-10.60
-22.10
-22.19
-22.29
2.05
1.00
f1 (ppm)

3c

HRMS

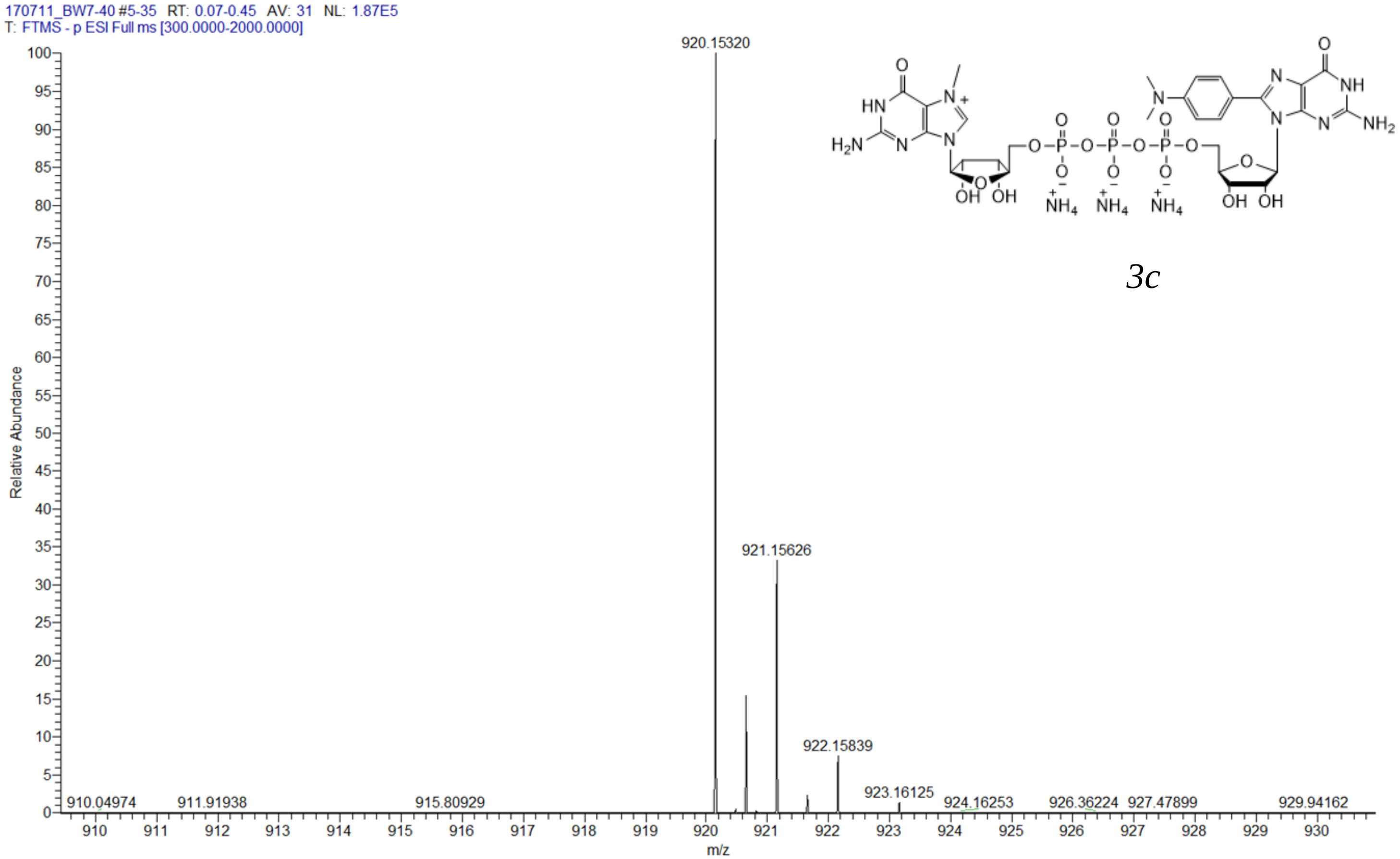

3c

Summary

Rt (A) = 11.07 min; ^{1}H NMR (500 MHz, deuterium oxide) δ 8.97 (s, 1H), 7.35 (d, *J* = 8.4 Hz, 2H), 6.85 (d, *J* = 8.4 Hz, 2H), 5.71 (d, *J* = 5.1 Hz, 1H), 5.67 (d, *J* = 3.9 Hz, 1H), 5.11 (s, 1H), 4.56 (t, *J* = 5.1 Hz, 1H), 4.46–4.39 (m, 2H), 4.36 (t, *J* = 5.0 Hz, 1H), 4.32 (dt, *J* = 12.0 Hz, 3.4 Hz, 1H), 4.23 (m, 3H), 4.12 (m, 1H), 4.03 (s, 3H), 3.01 (s, 6H); ^{31}P NMR (202 MHz, deuterium oxide) δ –10.20 to –10.70 (m, 2P), –22.19 (t, *J* = 19.4 Hz, 1P); HRMS ESI (-) m/z [M-H]$^{-}$, calcd for $C_{29}H_{37}N_{11}O_{18}P_3^{-}$ [M-H]$^{-}$ 920.1536; found 920.1532.

m^7GpppG^{8PhCN} (3d)

Structure

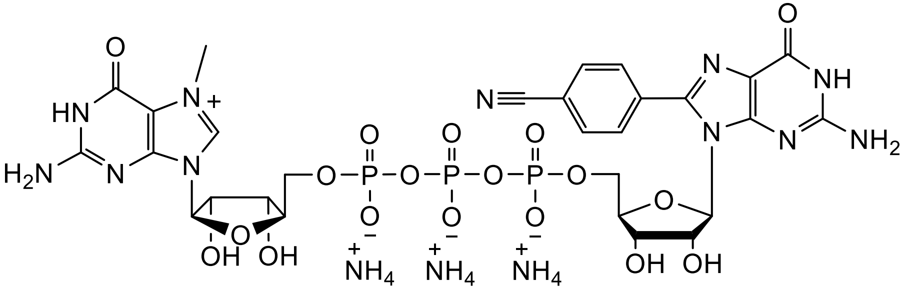

RP-HPLC profile

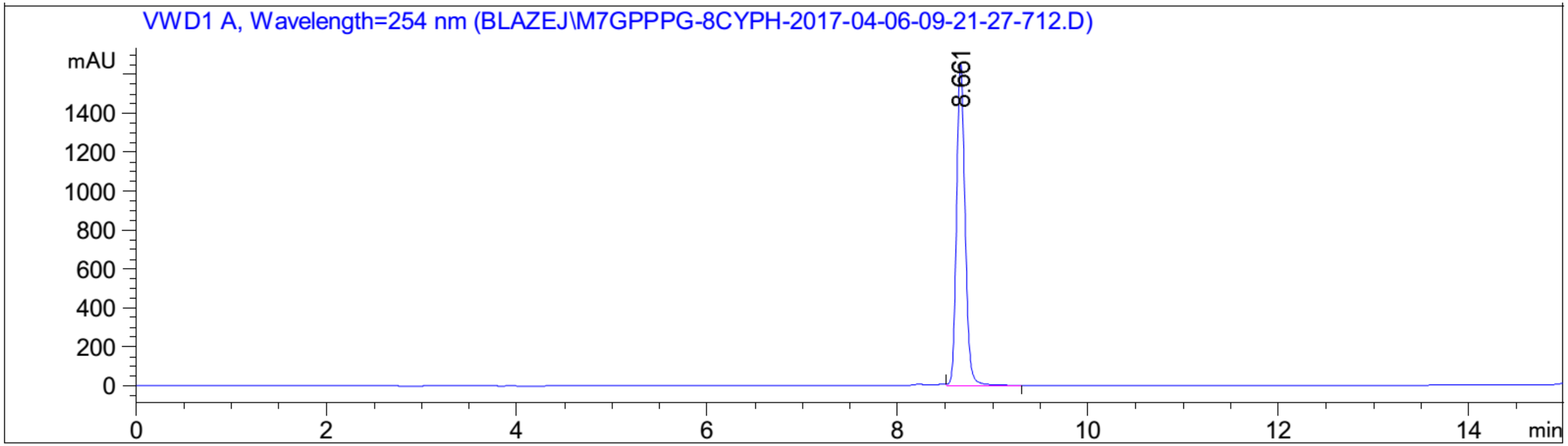

^{1}H NMR

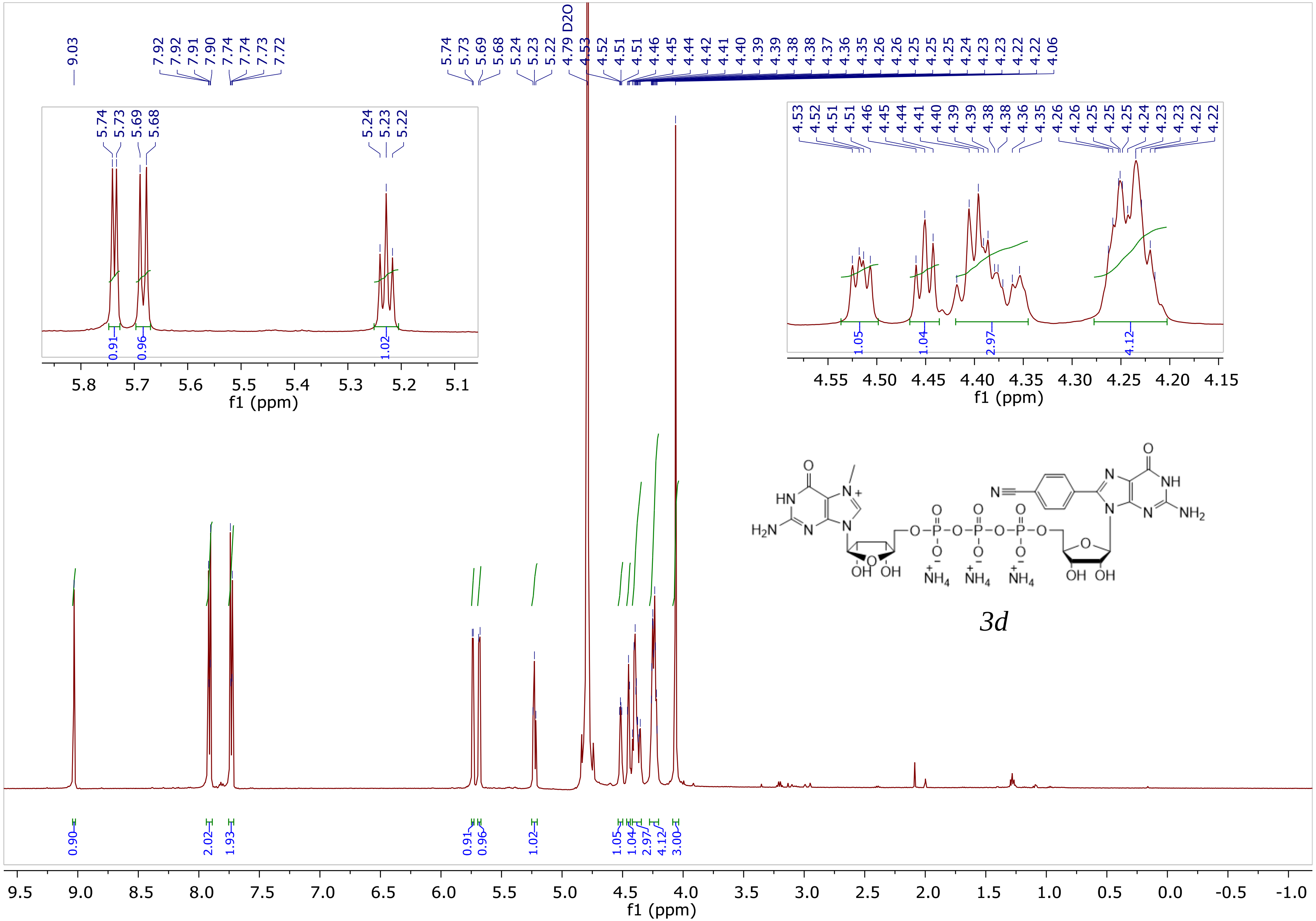

3d

31P NMR

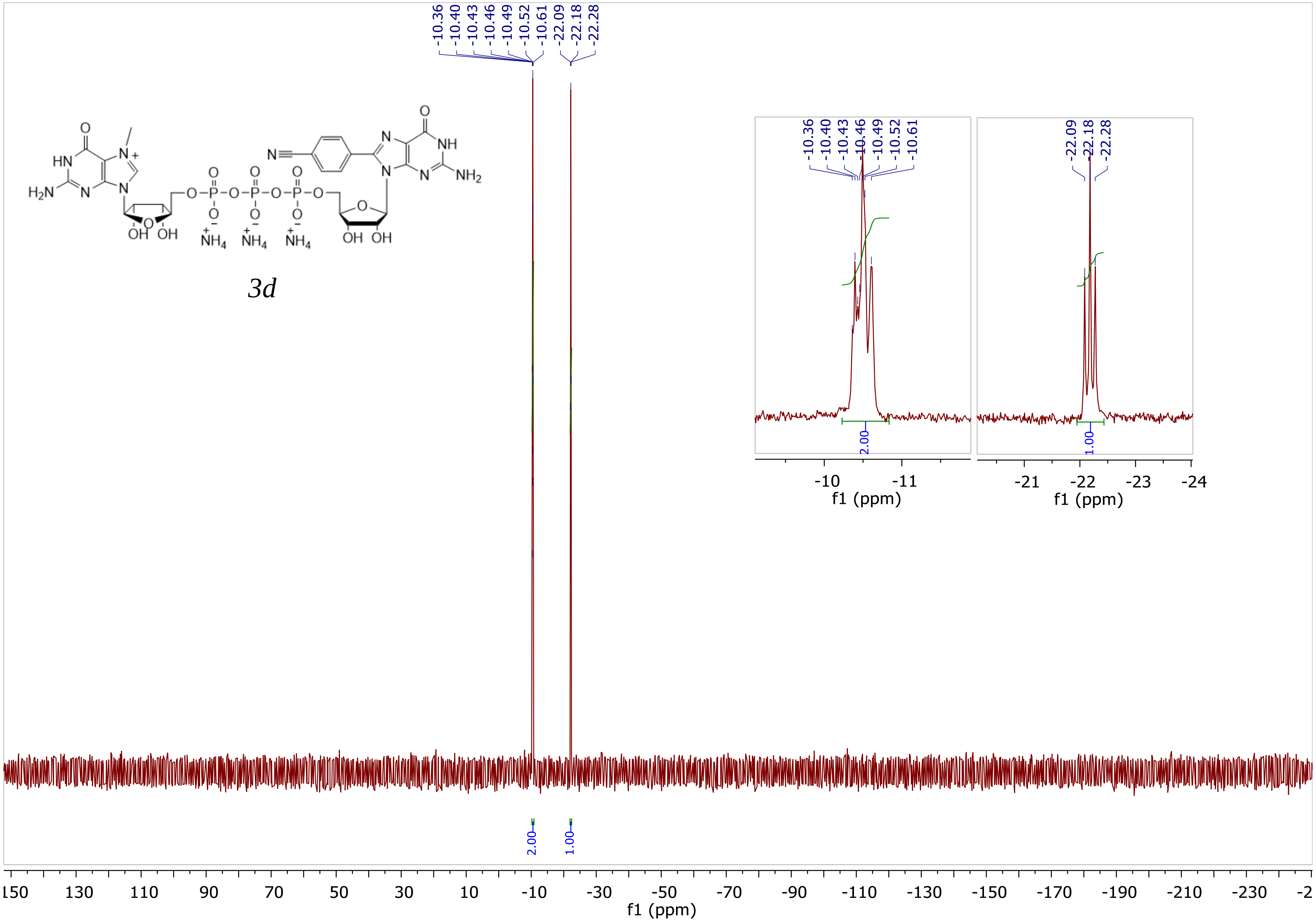

-10.36
-10.40
-10.43
-10.46
-10.49
-10.52
-10.61
-22.09
-22.18
-22.28
3d
2.00
1.00
-10
-11
f1 (ppm)
-21
-22
-23
-24
f1 (ppm)
150
130
110
90
70
50
30
10
-10
-30
-50
-70
-90
-110
-130
-150
-170
-190
-210
-230
-2
f1 (ppm)

HRMS

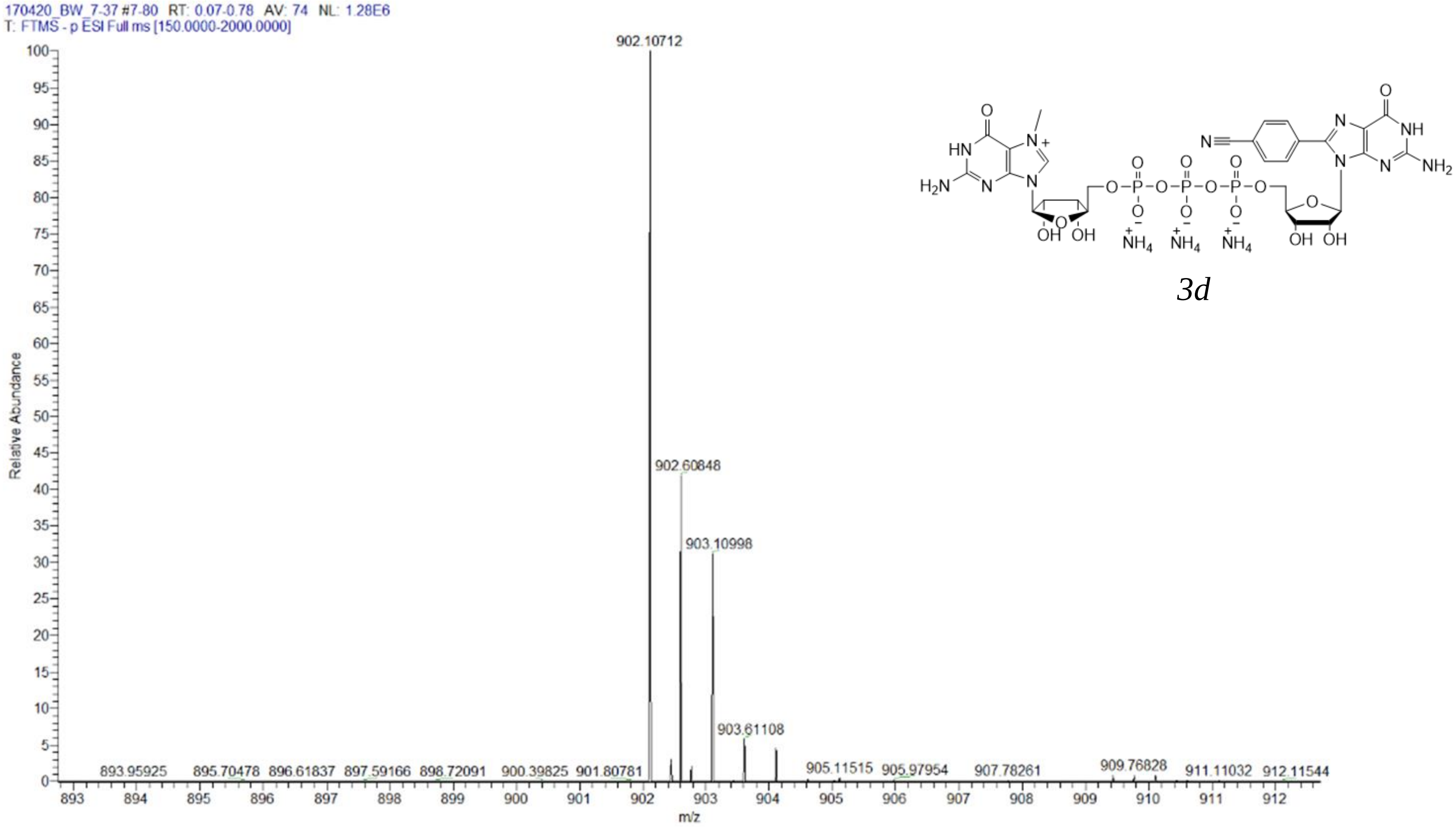

3d

Summary

Rt (A) = 8.66 min; ^{1}H NMR (500 MHz, deuterium oxide) δ 9.03 (s, 1H), 7.91 (d, *J* = 8.4 Hz, 2H), 7.73 (d, *J* = 8.4 Hz, 2H), 5.74 (d, *J* = 3.9 Hz, 1H), 5.68 (d, *J* = 6.0 Hz, 1H), 5.23 (t, *J* = 6.0 Hz, 1H), 4.52 (dd, *J* = 5.8 Hz, 3.9 Hz, 1H), 4.45 (t, *J* = 4.6 Hz, 4.0 Hz, 1H), 4.39 (m, 3H), 4.24 (m, 4H), 4.06 (s, 3H); ^{31}P NMR (202 MHz, deuterium oxide) δ –10.32 to –10.67 (m, 2P), –22.18 (t, *J* = 19.3 Hz, 1P); HRMS ESI (-) m/z [M-H]$^{-}$, calcd for $C_{28}H_{31}N_{11}O_{18}P_3^{-}$ [M-H]$^{-}$ 902.1067; found 902.1071.

$m^{2'O,7}GpppG^{8Py}$ (4a)

Structure

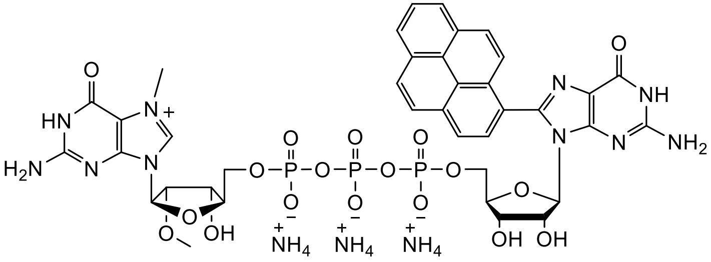

RP-HPLC profile

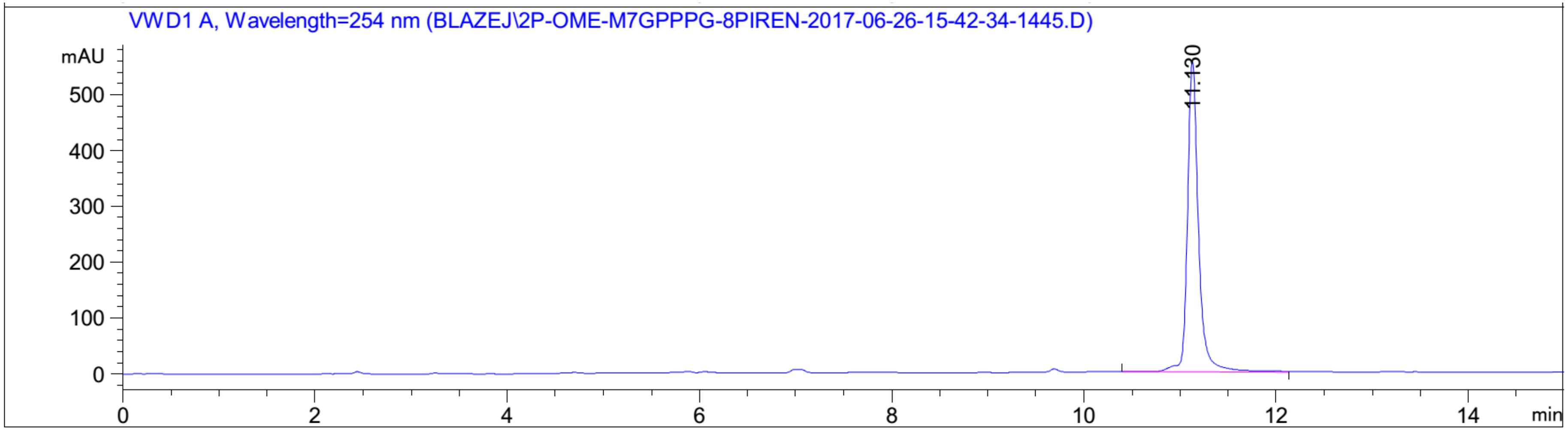

1H NMR

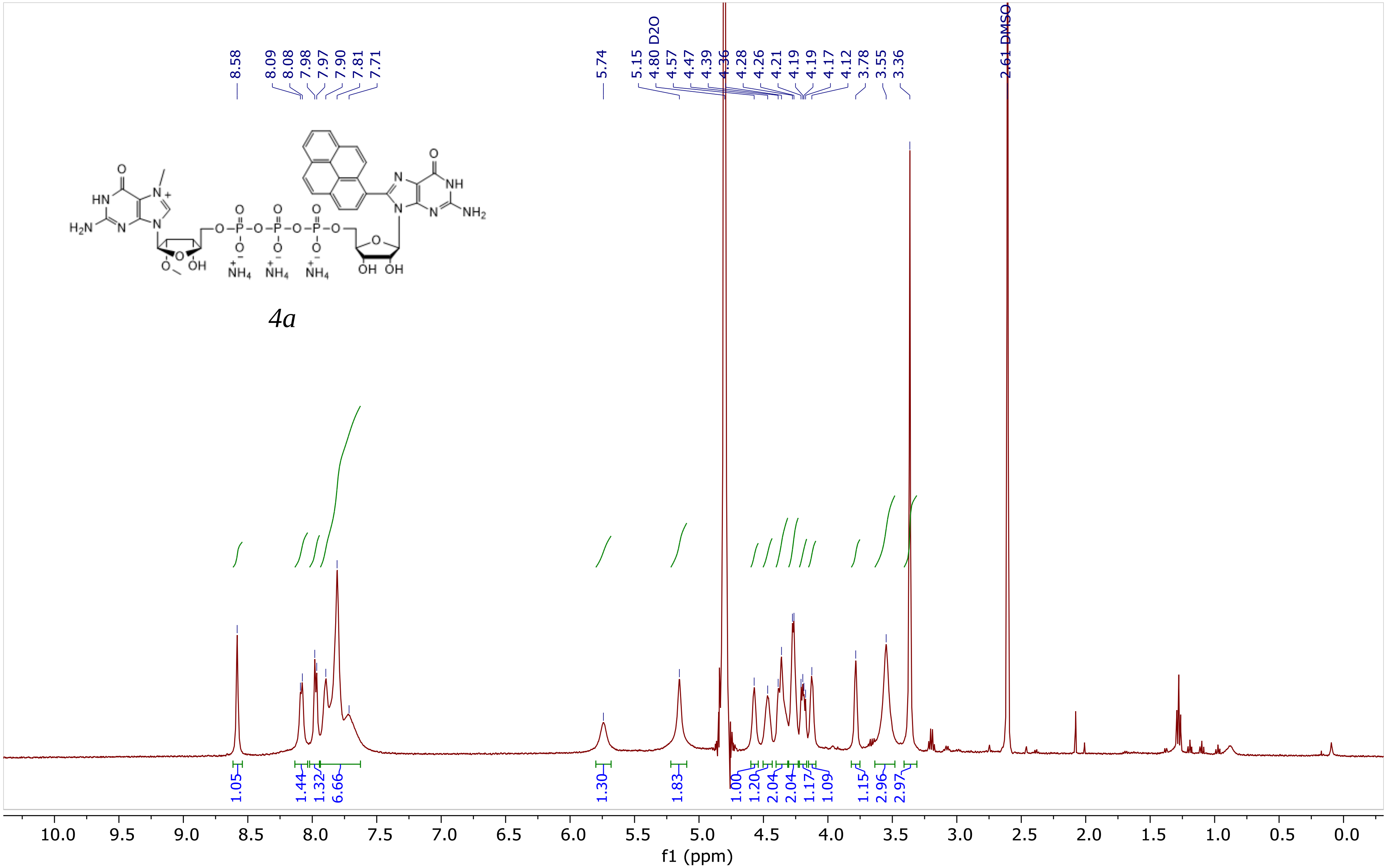
8.58
8.09
8.08
7.98
7.97
7.90
7.81
7.71
5.74
5.15
4.80 D2O
4.57
4.47
4.39
4.36
4.28
4.26
4.21
4.19
4.19
4.17
4.12
3.78
3.55
3.36
2.61 DMSO
4a
1.05
1.44
1.32
6.66
1.30
1.83
1.00
1.20
2.04
2.04
1.17
1.09
1.15
2.96
2.97
f1 (ppm)

31P NMR

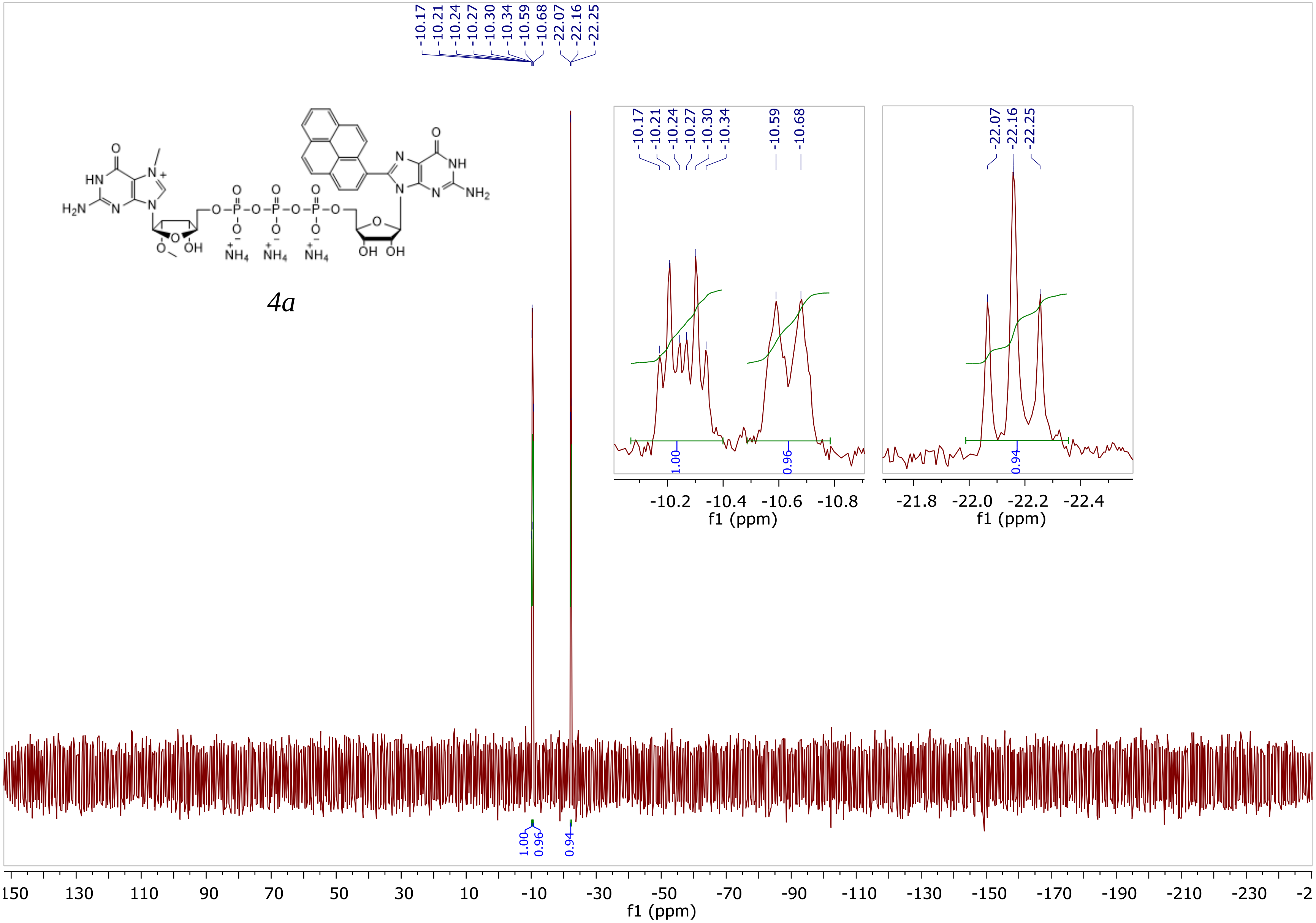

-10.17
-10.21
-10.24
-10.27
-10.30
-10.34
-10.59
-10.68
-22.07
-22.16
-22.25
HN
N+
H2N
N
N
O
O
OH
O
P
O
NH4
NH
NH2
OH OH
4a
1.00
0.96
0.94
-10.2 -10.4 -10.6 -10.8
f1 (ppm)
-21.8 -22.0 -22.2 -22.4
f1 (ppm)
150 130 110 90 70 50 30 10 -10 -30 -50 -70 -90 -110 -130 -150 -170 -190 -210 -230
f1 (ppm)

HRMS

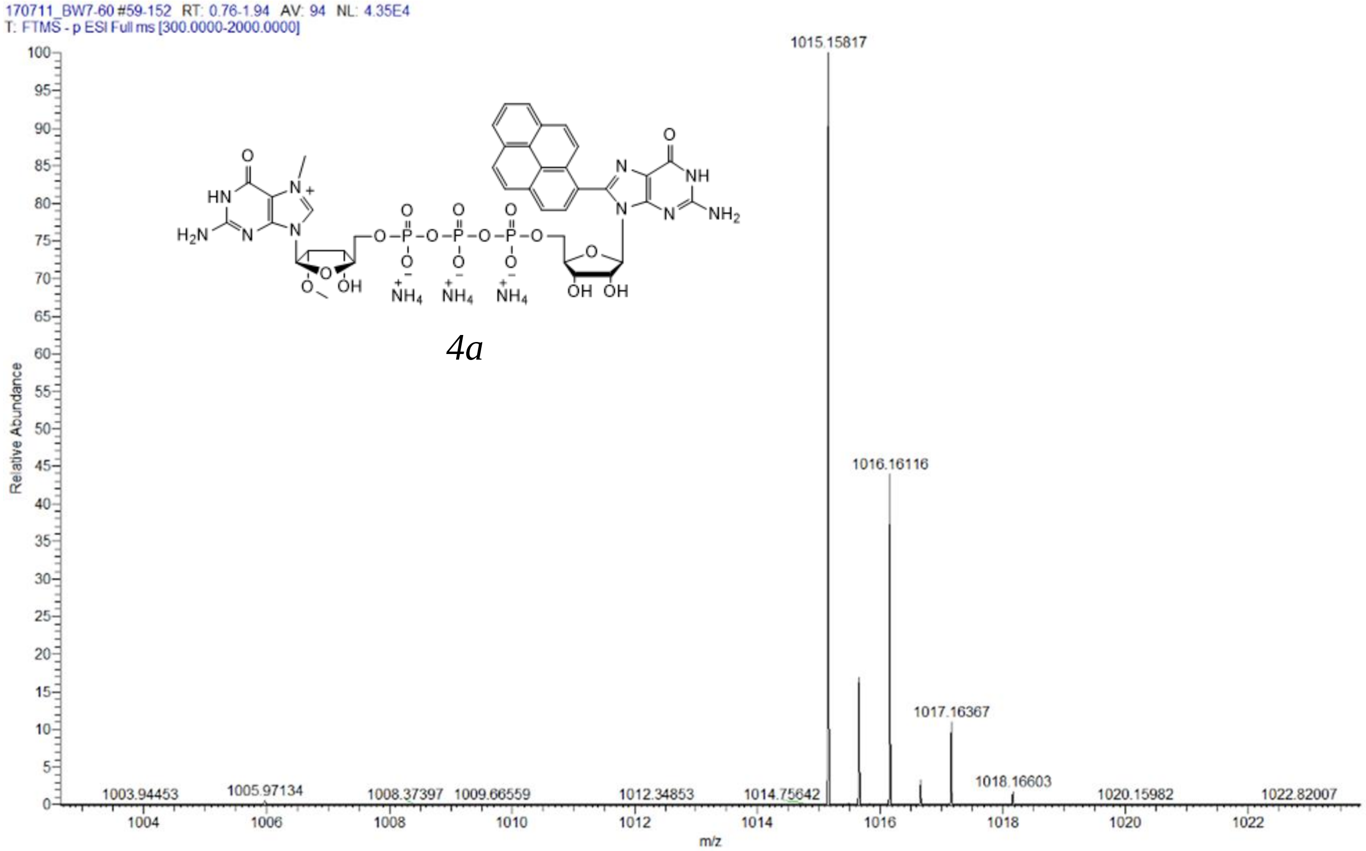

Summary

RP-HPLC: Rt (C) = 11.13 min; ^{1}H NMR (500 MHz, deuterium oxide) δ 8.58 (s, 1H), 8.08 (d, *J* = 7.8 Hz, 1H), 7.97 (d, *J* = 7.8 Hz, 1H), 7.94–7.63 (m, 7H), 5.74 (s, 1H), 5.15 (s, 2H), 4.57 (s, 1H), 4.52–4.41 (m, 1H), 4.41–4.29 (m, 2H), 4.29-4.22 (m, 2H), 4.19 (dd, *J* = 11.0 Hz, 6.6, 1H), 4.12 (dd, 1H, *J* = 11.0 Hz, 6.6 Hz), 3.78 (s, 1H), 3.55 (s, 3H), 3.36 (s, 3H); ^{31}P NMR (202 MHz, deuterium oxide) δ –10.26 (dt, *J* = 19.3 Hz, 7.1 Hz, 1P), –10.64 (d, *J* = 17.9 Hz, 1P), –22.16 (t, *J* = 19.3 Hz, 1P); HRMS ESI (-) m/z [M-H]$^{-}$, calcd for $C_{38}H_{38}N_{10}O_{18}P_3^{-}$ [M-H]$^{-}$ 1015.1584; found 1015.1582.

$m^{2'O,7}GpppG^{8Ph}$ (4b)

Structure

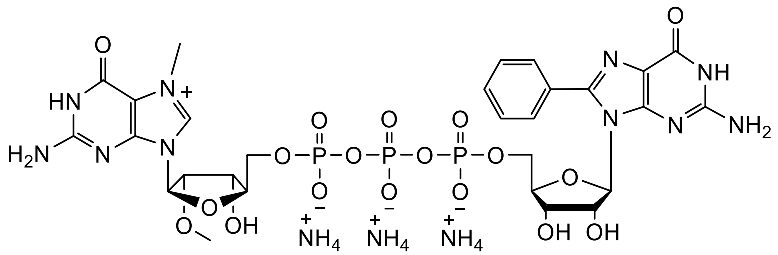

RP-HPLC profile

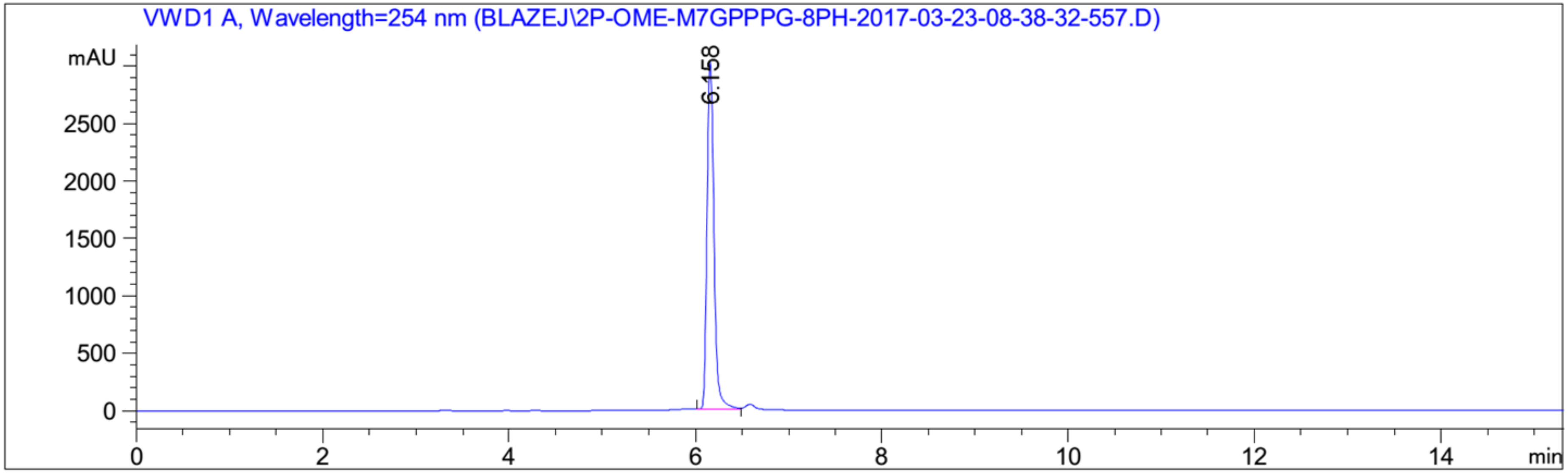

1H NMR

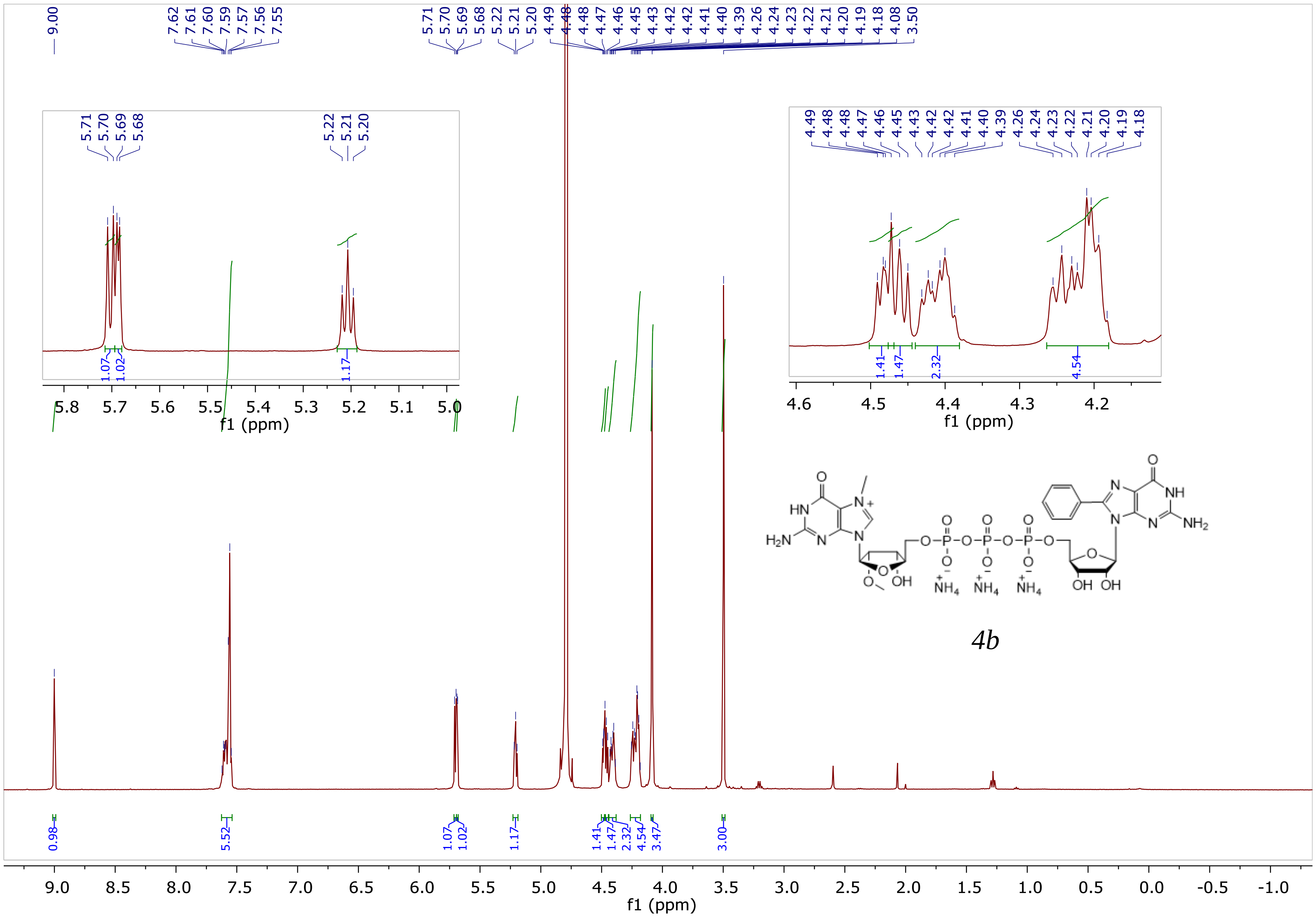

4b

31P NMR

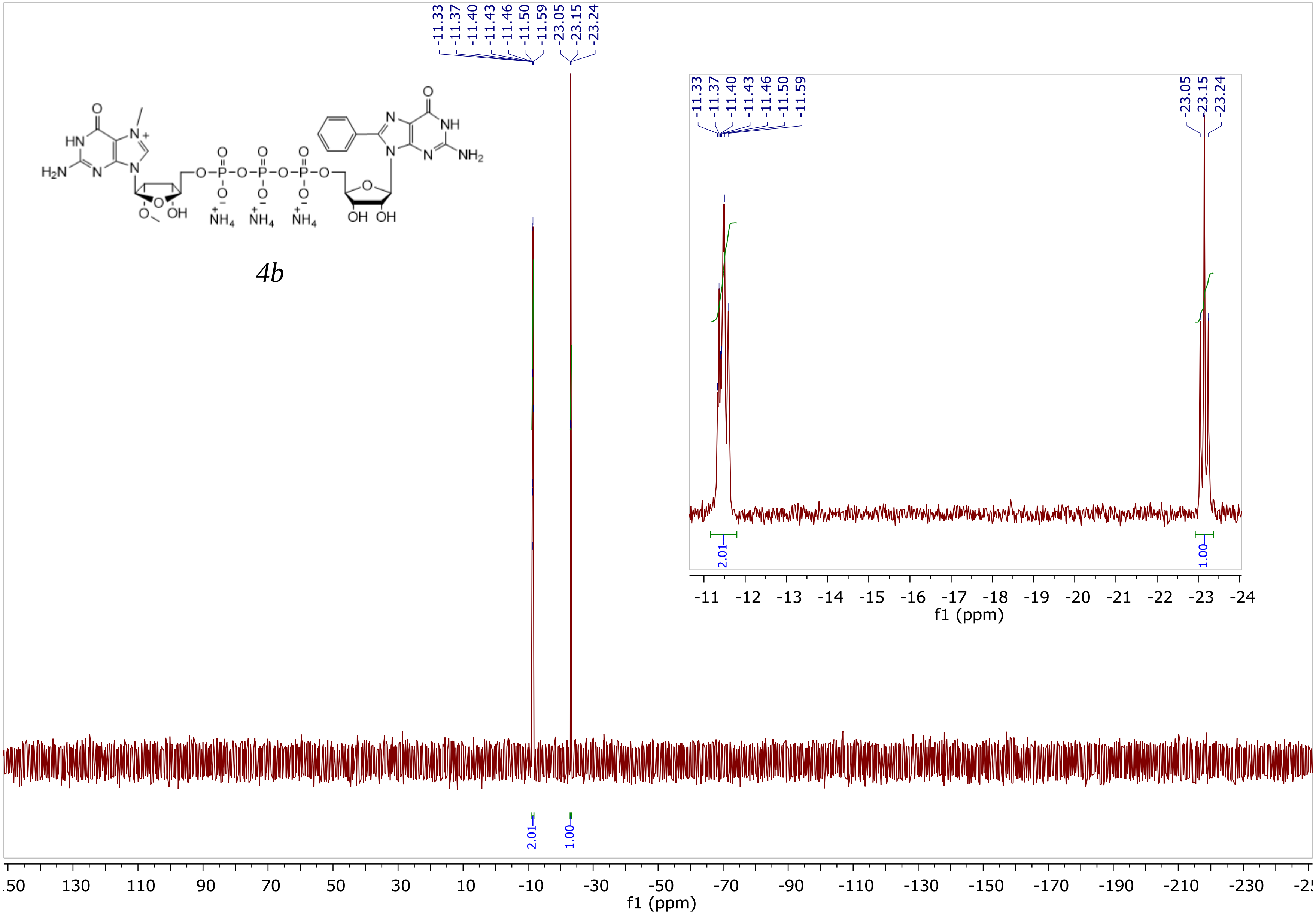

-11.33
-11.37
-11.40
-11.43
-11.46
-11.50
-11.59
-23.05
-23.15
-23.24
4b
2.01
1.00
f1 (ppm)

HRMS

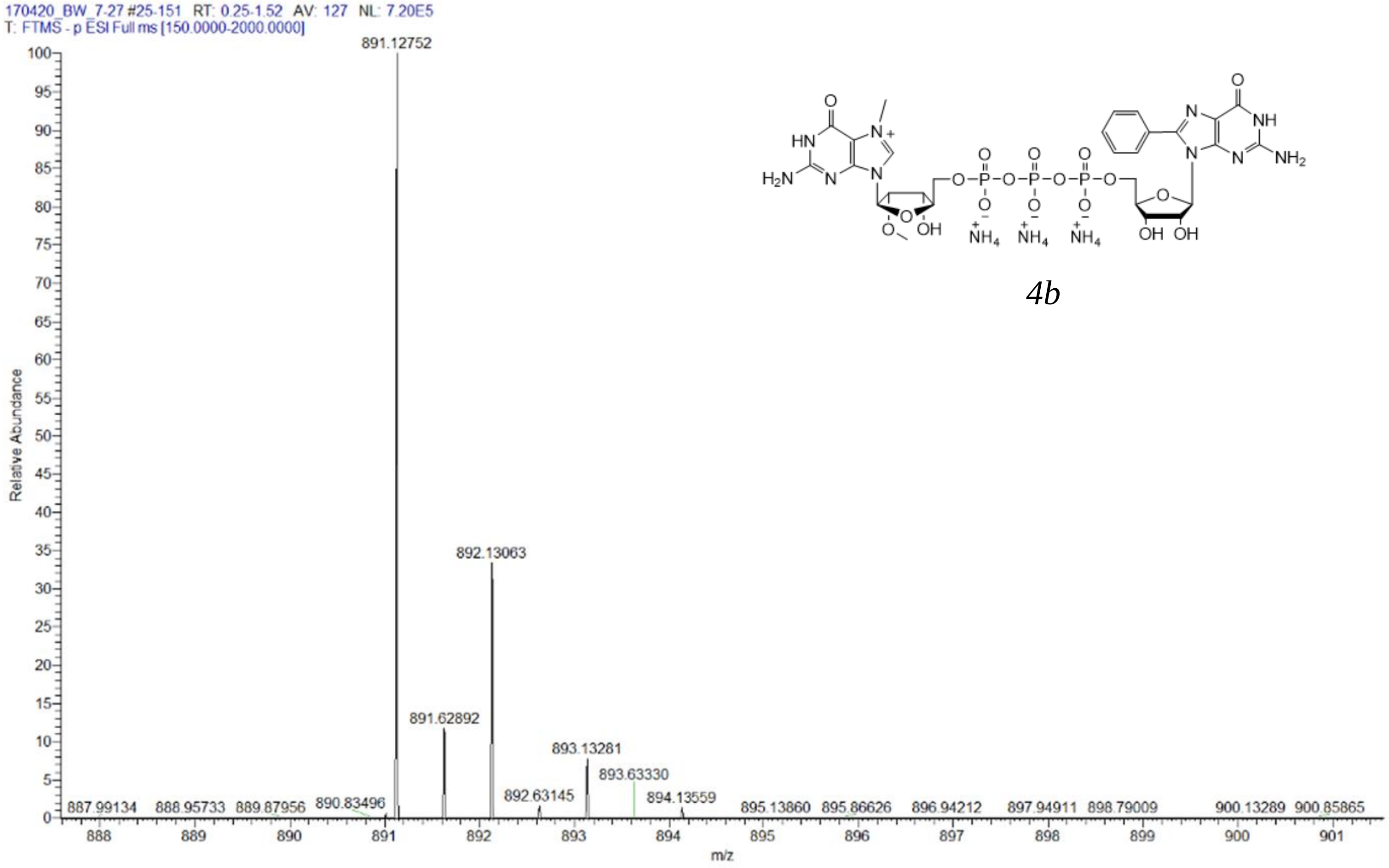

4b

Summary

RP-HPLC: Rt (A) = 6.16 min; ^{1}H NMR (500 MHz, deuterium oxide) δ 9.00 (s, 1H), 7.58 (m, 5H), 5.70 (d, *J* = 5.8 Hz, 1H), 5.69 (d, *J* = 2.8 Hz, 1H), 5.21 (t, *J* = 5.8 Hz, 1H), 4.48 (dd *J* = 5.8 Hz, 3.8 Hz, 1H), 4.46 (t, *J* = 5.8 Hz, 5.4 Hz, 1H), 4.44–4.38 (m, 2H), 4.26–4.18 (m, 5H), 4.08 (s, 3H), 3.50 (s, 3H); ^{31}P NMR (202 MHz, deuterium oxide) δ –11.25 to –11.60 (m, 2P), –23.15 (t, *J* = 19.6 Hz, 1P); HRMS ESI (-) m/z [M-H]$^-$, calcd for $C_{28}H_{34}N_{10}O_{18}P_3^-$ [M-H]$^-$ 891.1271; found 891.1275.

$m^{2'O,7}GpppG^{8Me}$ (4e)

Structure

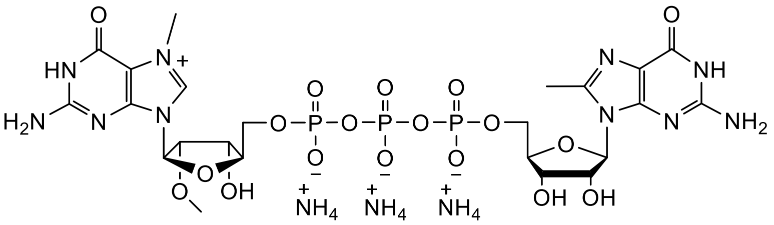

RP-HPLC profile

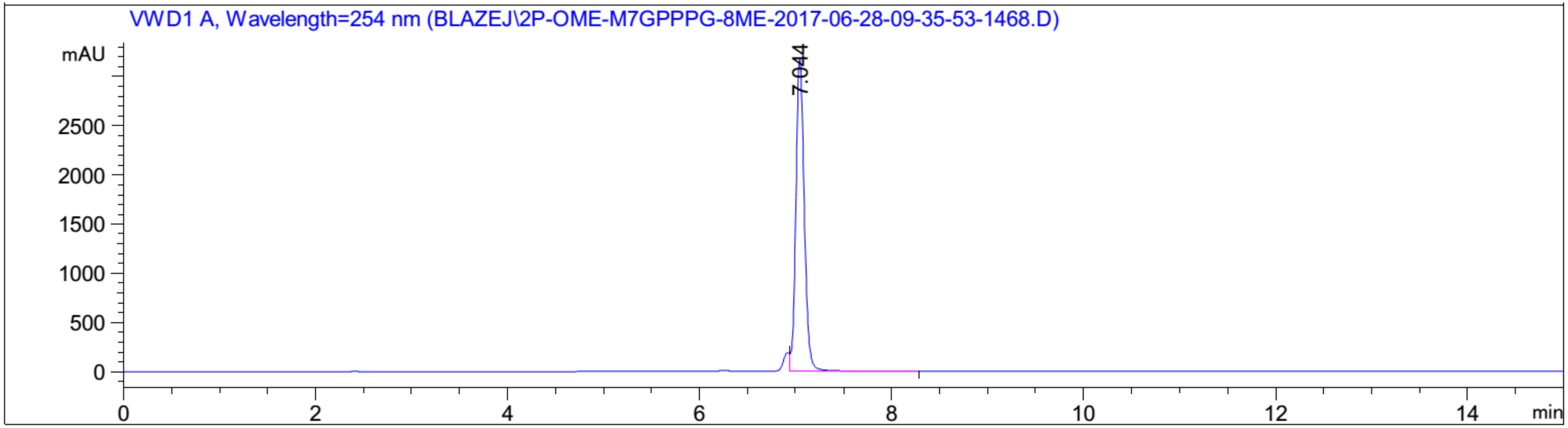

1H NMR

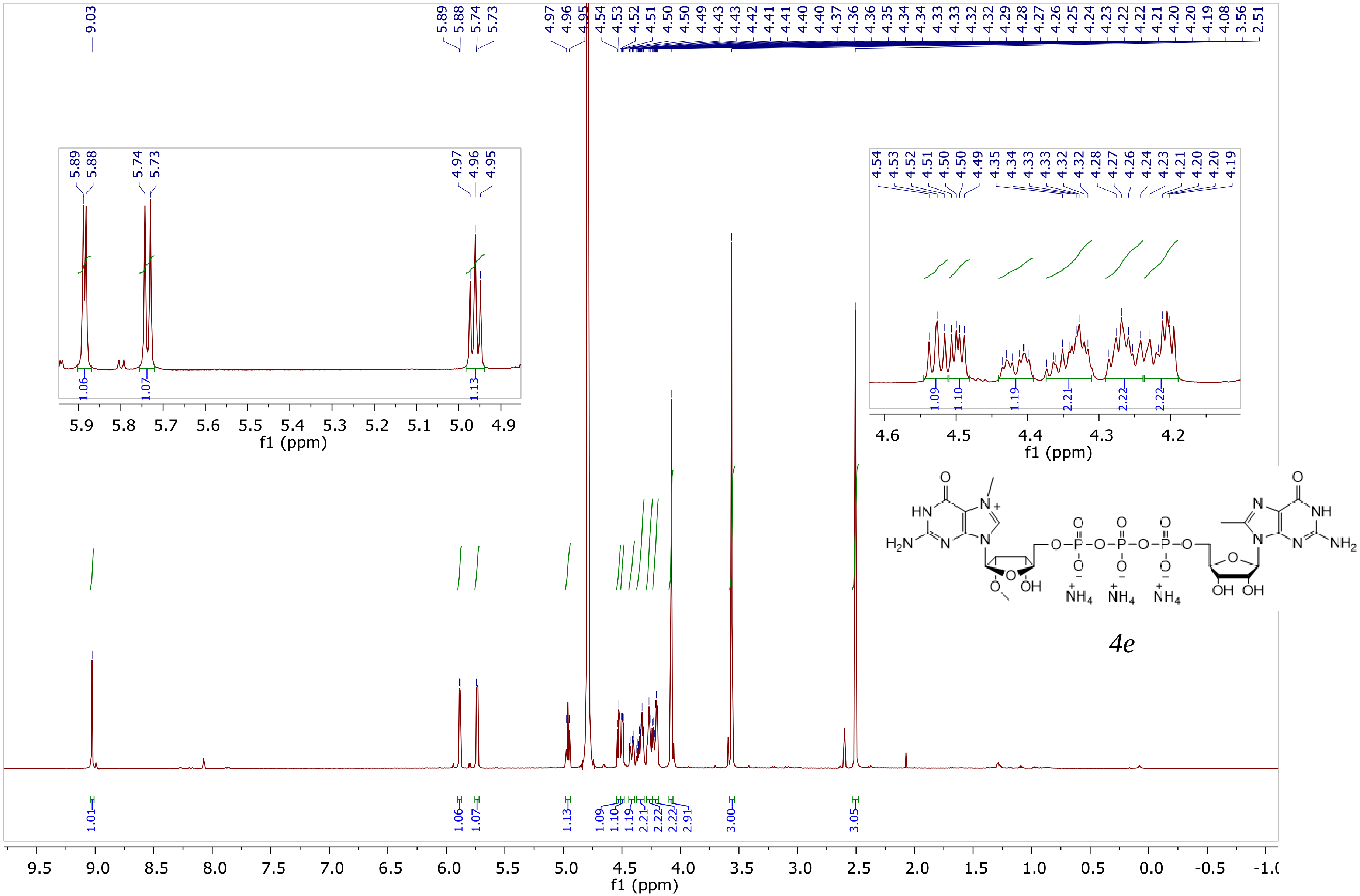

4e

1H-1H COSY NMR

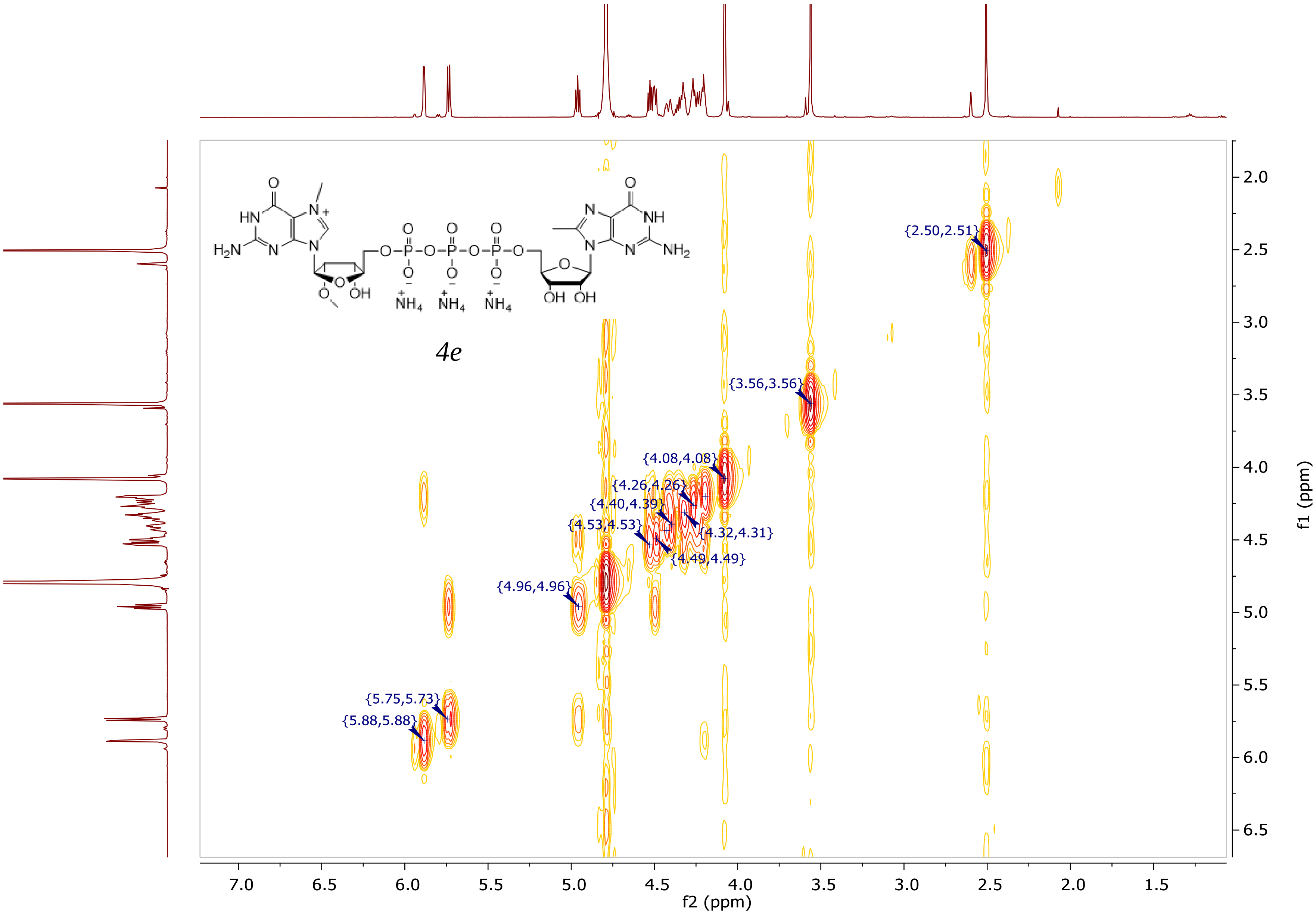

31P NMR

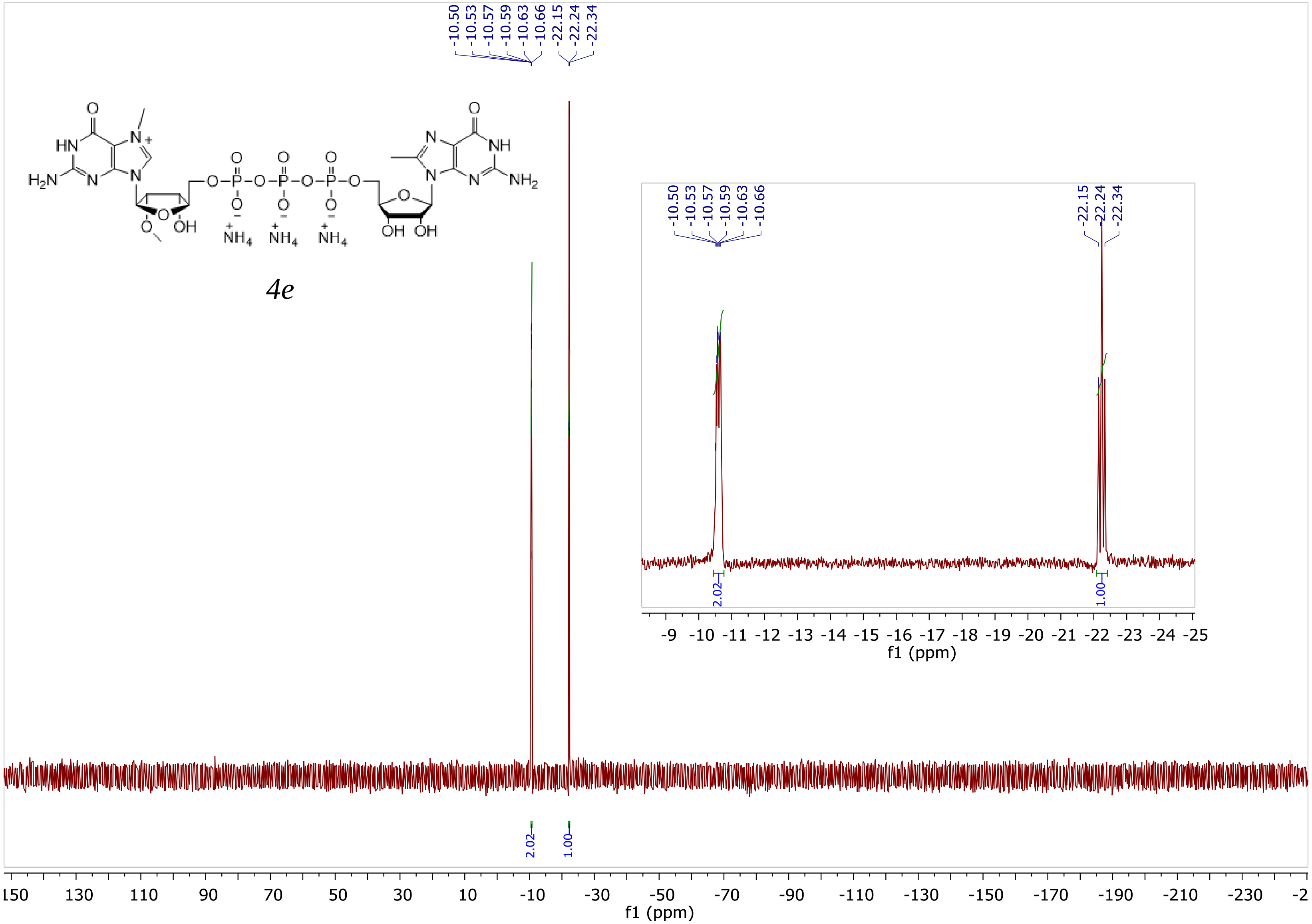

-10.50
-10.53
-10.57
-10.59
-10.63
-10.66
-22.15
-22.24
-22.34
4e
2.02
1.00
-9 -10 -11 -12 -13 -14 -15 -16 -17 -18 -19 -20 -21 -22 -23 -24 -25
f1 (ppm)
150 130 110 90 70 50 30 10 -10 -30 -50 -70 -90 -110 -130 -150 -170 -190 -210 -230
f1 (ppm)

HRMS

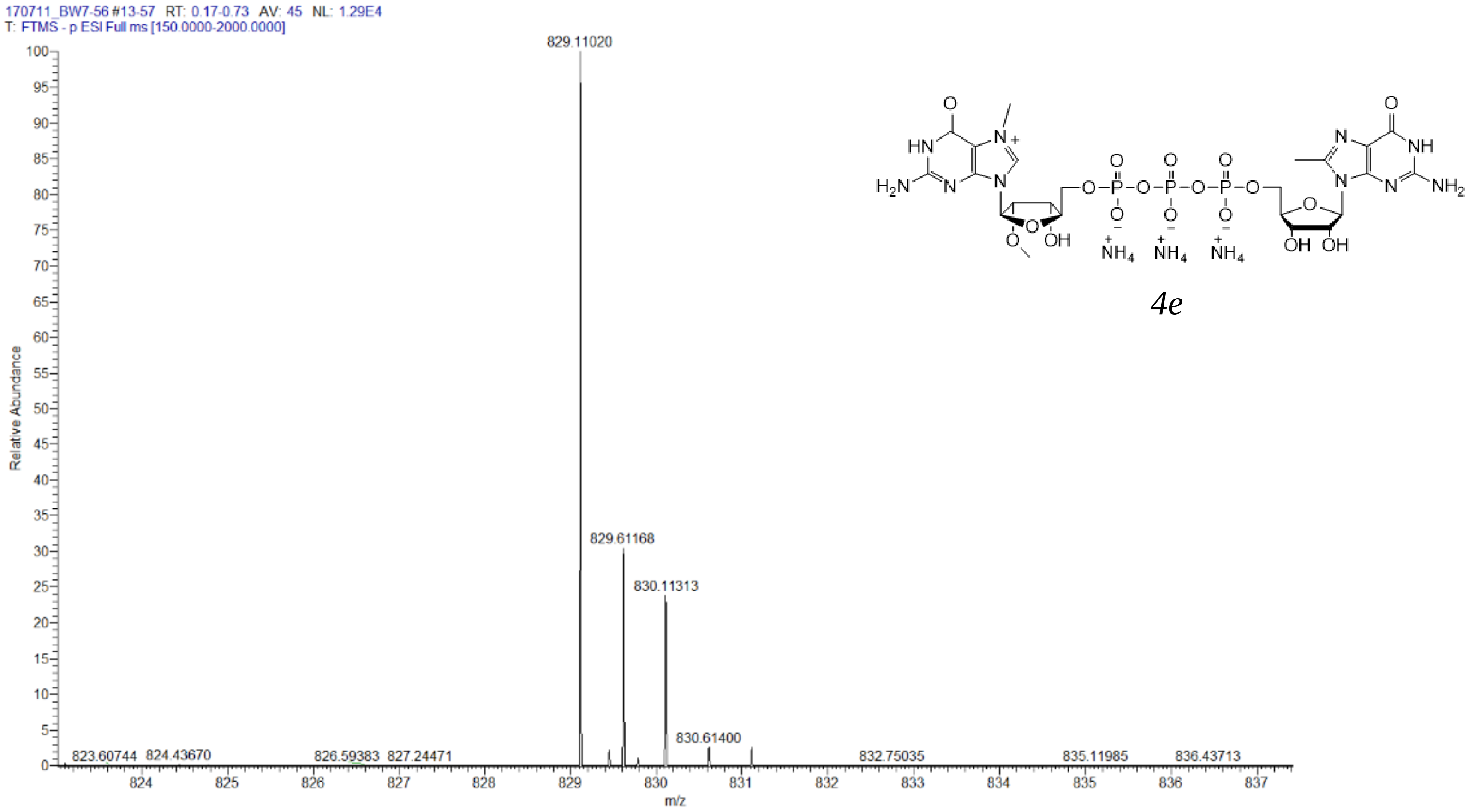

4e

Summary

Rt (A) = 7.04 min; ^{1}H NMR (500 MHz, deuterium oxide) δ 9.03 (s, 1H), 5.89 (d, *J* = 3.2 Hz, 1H), 5.74 (d, *J* = 6.5 Hz, 1H), 4.96 (t, *J* = 6.5 Hz, 6.0 Hz, 1H), 4.53 (dd, *J* = 6.0 Hz, 5.6 Hz, 1H), 4.50 (dd, *J* = 5.6 Hz, 3.4 Hz, 1H), 4.44–4.41 (m, 1H), 4.40–4.39 (m, 1H), 4.38–4.31 (m, 2H), 4.29–4.19 (m, 3H), 4.08 (s, 3H), 3.56 (s, 3H), 2.51 (s, 3H); ^{31}P NMR (202 MHz, deuterium oxide) δ –10.50 to –10.66 (m, 2P), –22.24 (t, *J* = 19.4 Hz, 1P); HRMS ESI (-) m/z [M-H]$^-$, calcd for $C_{23}H_{32}N_{10}O_{18}P_3^-$ [M-H]$^-$ 829.1114; found 829.1102.

$^{8\text{-Ph}}m^{2'O,7}$GpppG (5b)

Structure

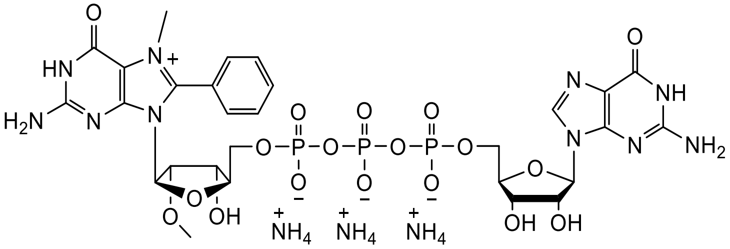

RP-HPLC profile

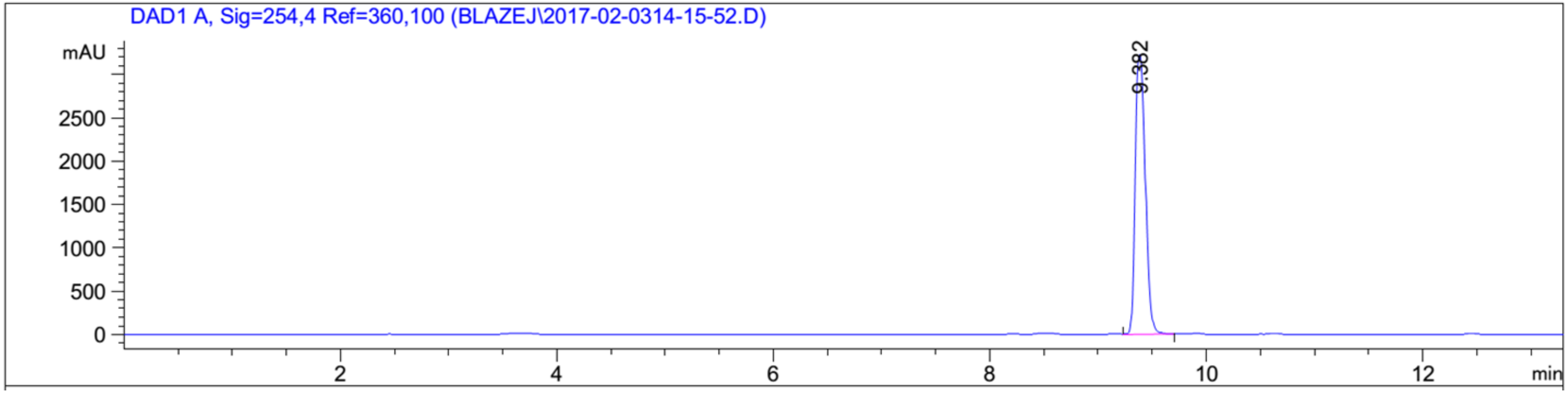

1H NMR

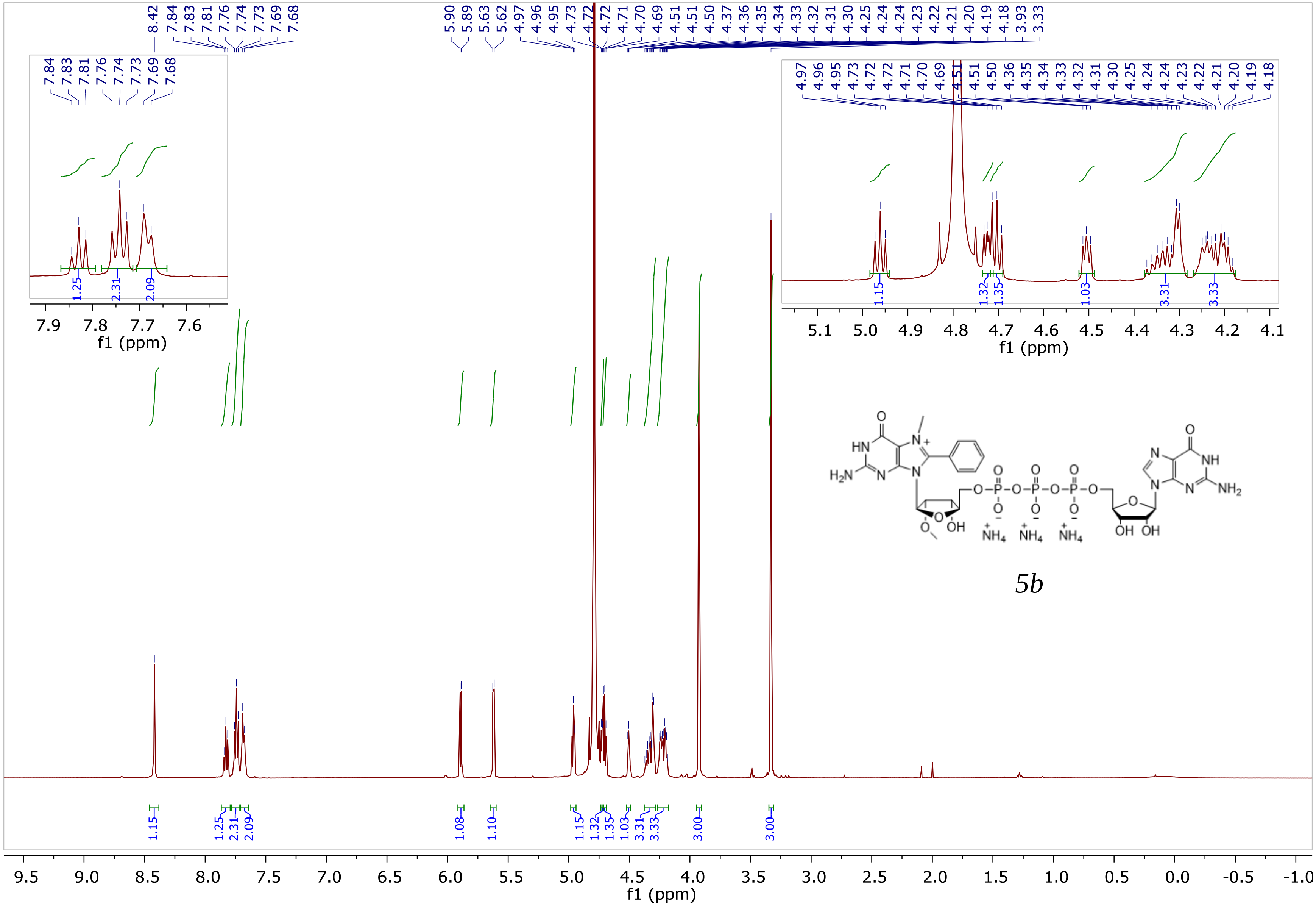

1H-1H COSY NMR

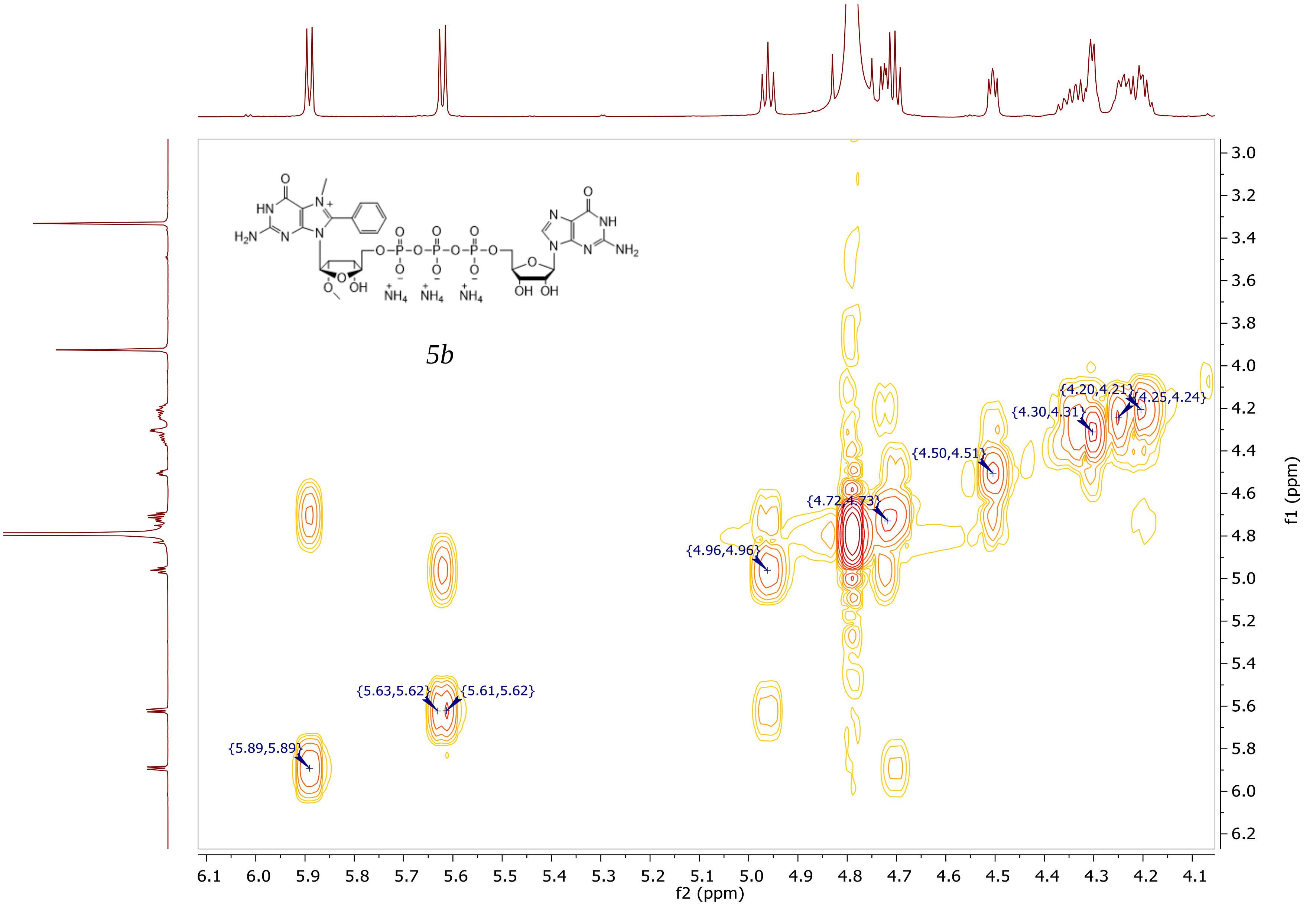

31P NMR

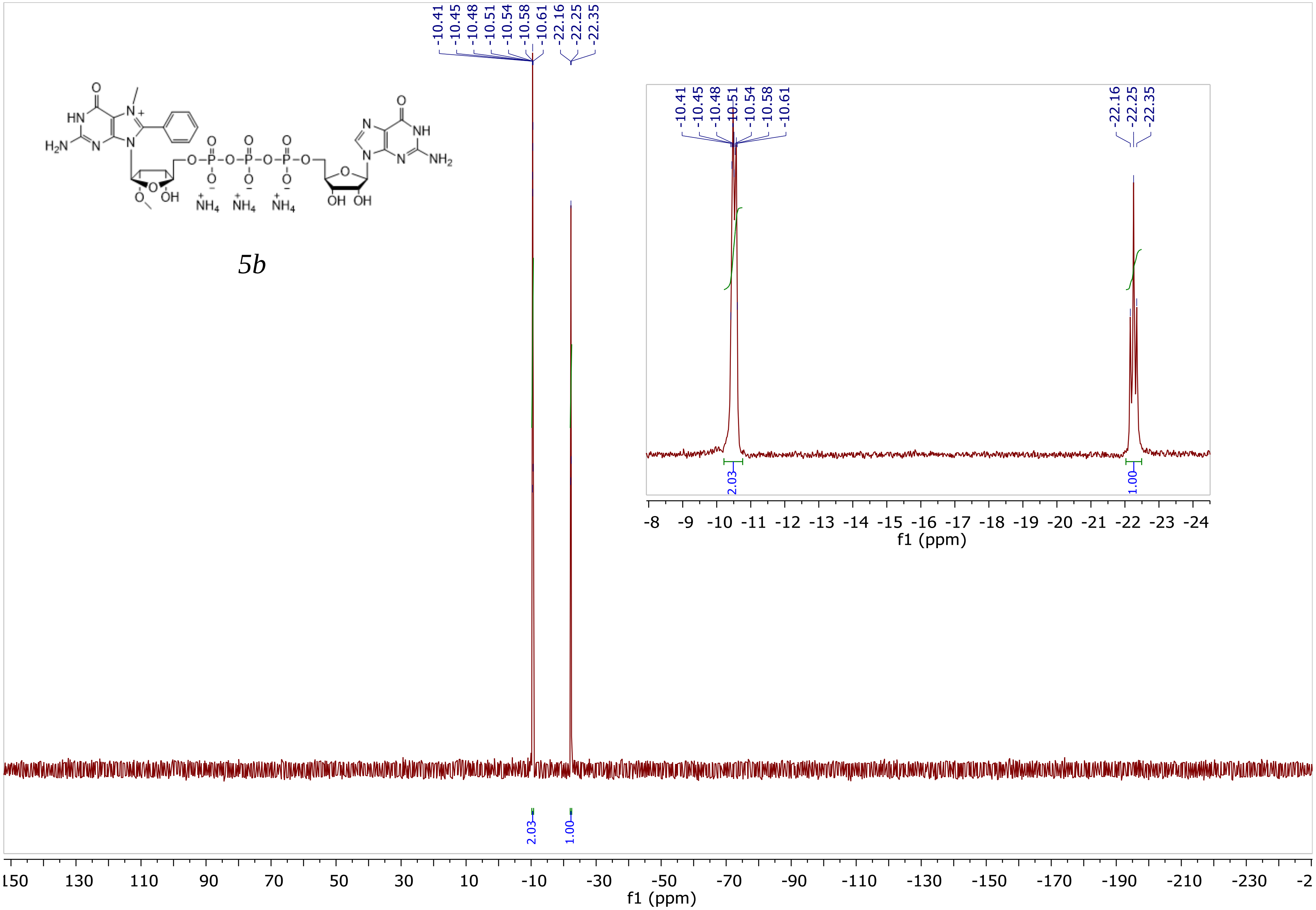
-10.41
-10.45
-10.48
-10.51
-10.54
-10.58
-10.61
-22.16
-22.25
-22.35
5b
2.03
1.00
-8 -9 -10 -11 -12 -13 -14 -15 -16 -17 -18 -19 -20 -21 -22 -23 -24
f1 (ppm)
150 130 110 90 70 50 30 10 -10 -30 -50 -70 -90 -110 -130 -150 -170 -190 -210 -230
f1 (ppm)

HRMS

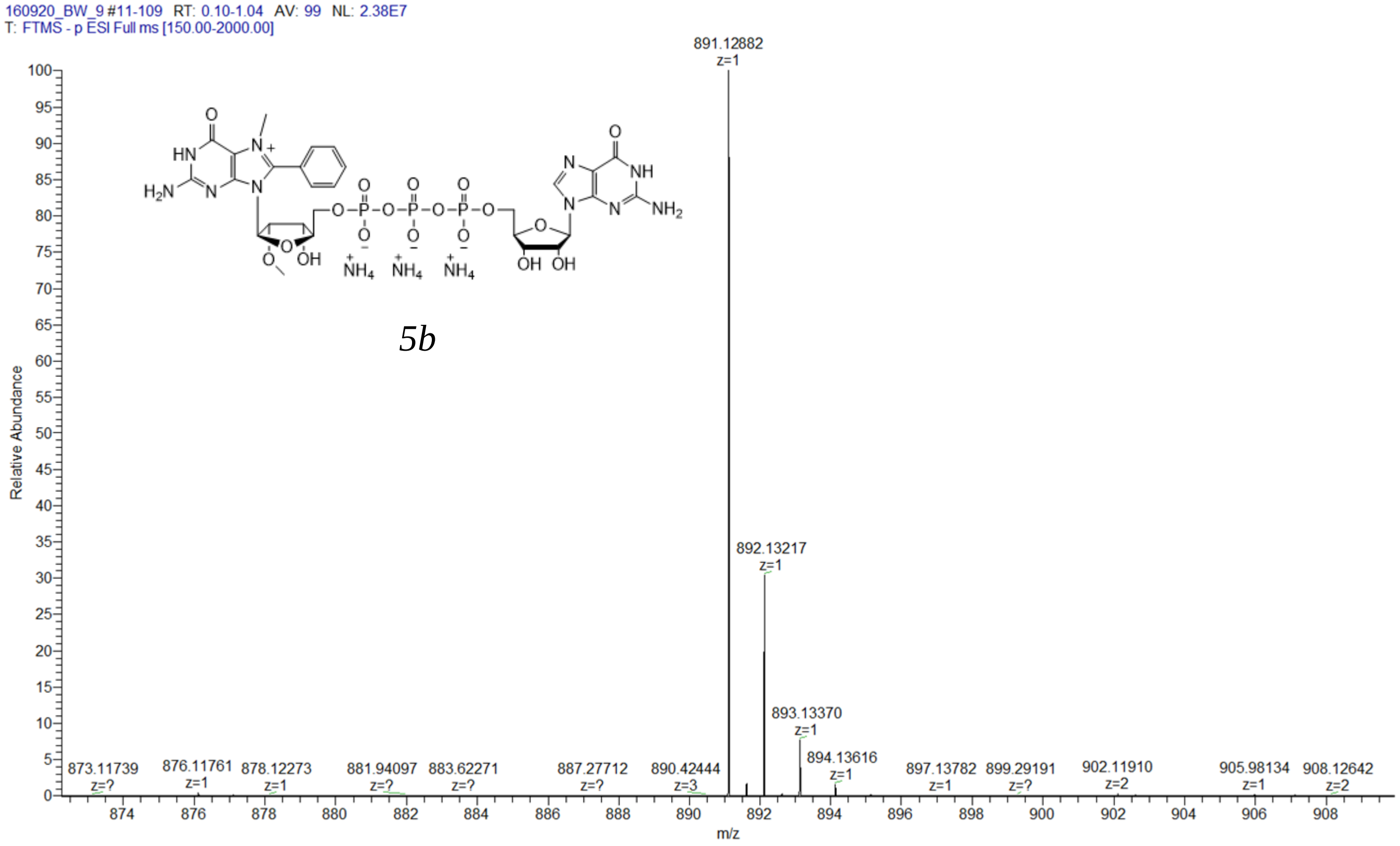

Summary

Rt (A) = 9.38 min; ^{1}H NMR (500 MHz, deuterium oxide) δ 8.42 (s, 1H), 7.83 (m, 1H), 7.74 (m, 2H), 7.68 (m, 2H), 5.89 (d, *J* = 5.2 Hz, 1H), 5.62 (d, *J* = 5.8 Hz, 1H), 4.96 (dd, *J* = 6.2 Hz, 5.2 Hz, 1H), 4.72 (dd, *J* = 5.8 Hz, 3.3 Hz, 1H), 4.70 (dd, *J* = 5.6 Hz, 5.2 Hz, 1H), 4.50 (dd, *J* = 5.2 Hz, 3.3 Hz, 1H), 4.32 (m, 3H), 4.22 (m, 3H), 3.93 (s, 3H), 3.33 (s, 3H); ^{31}P NMR (202 MHz, deuterium oxide) δ –10.30 to –10.70 (m, 2P), –22.25 (t, *J* = 19.6 Hz, 1P); HRMS ESI (-) m/z [M-H]$^-$, calcd for $C_{28}H_{34}N_{10}O_{18}P_3^-$ [M-H]$^-$ 891.1271; found 891.1288.

$^{8DMAPh}m^{2'O,7}GpppG$ (5c)

Structure

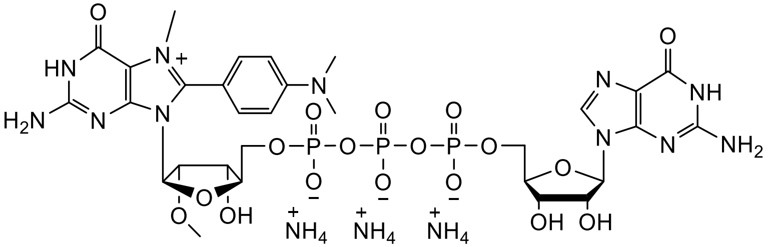

RP-HPLC profile

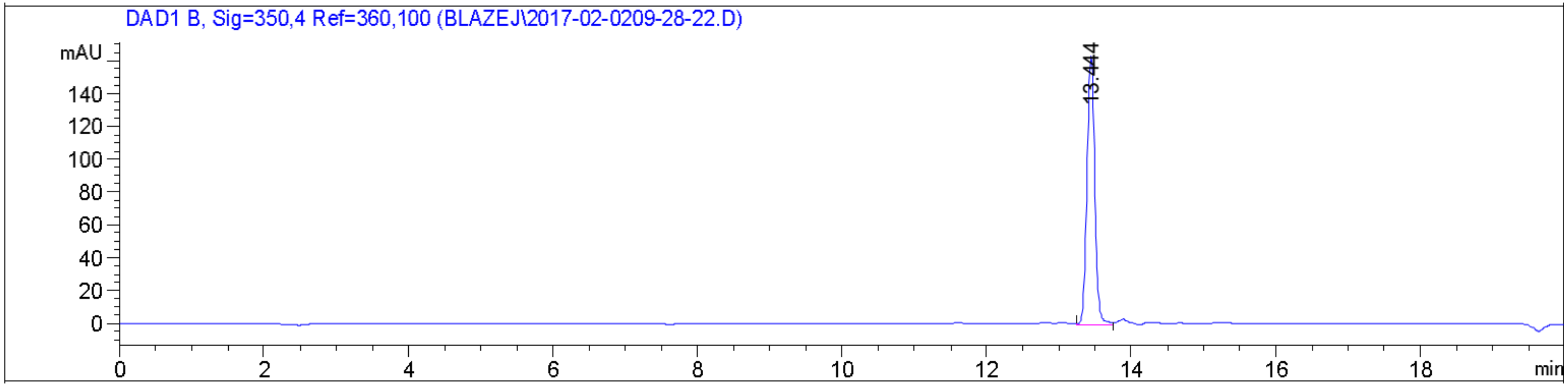

1H NMR

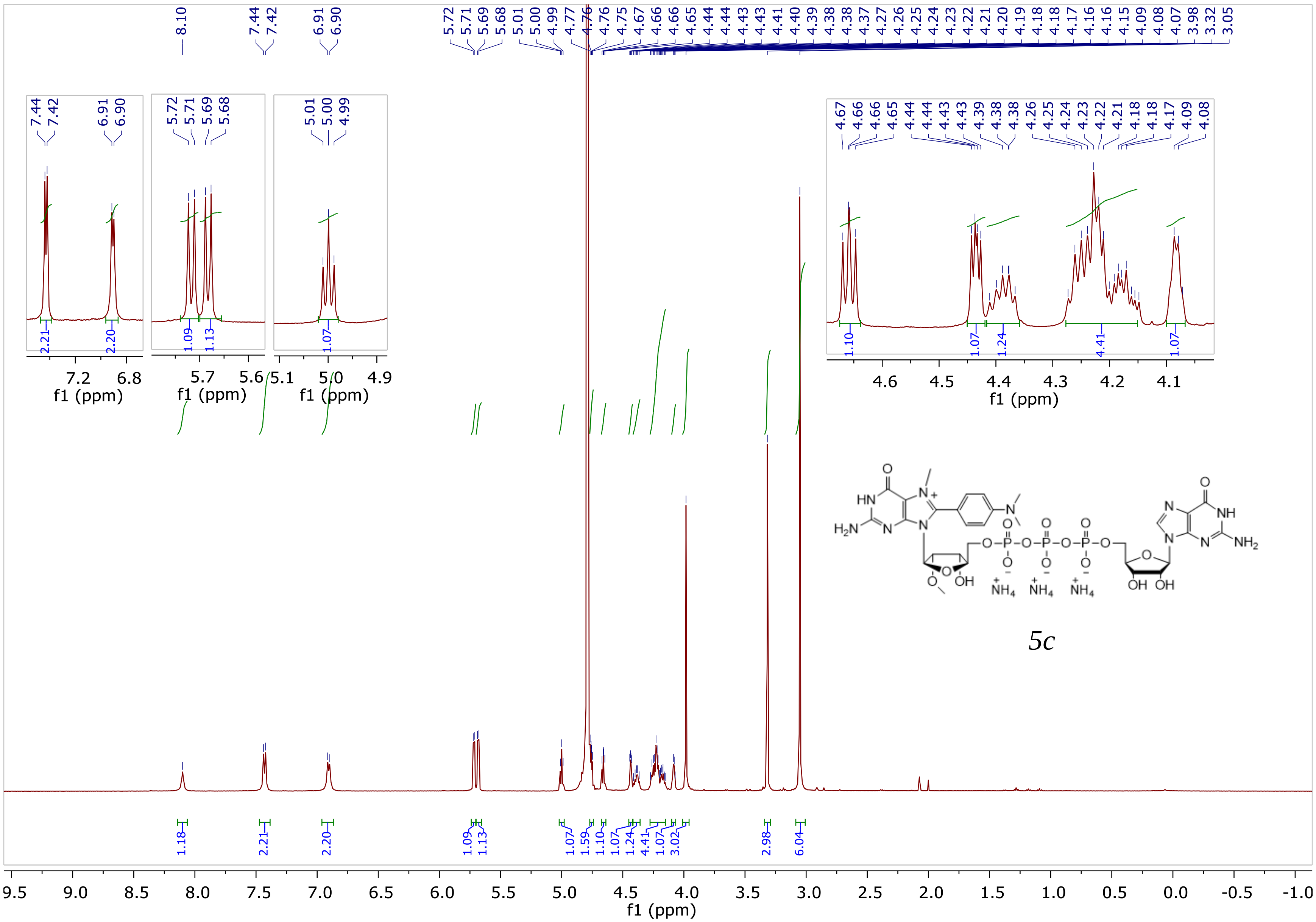

5c

1H-1H COSY NMR

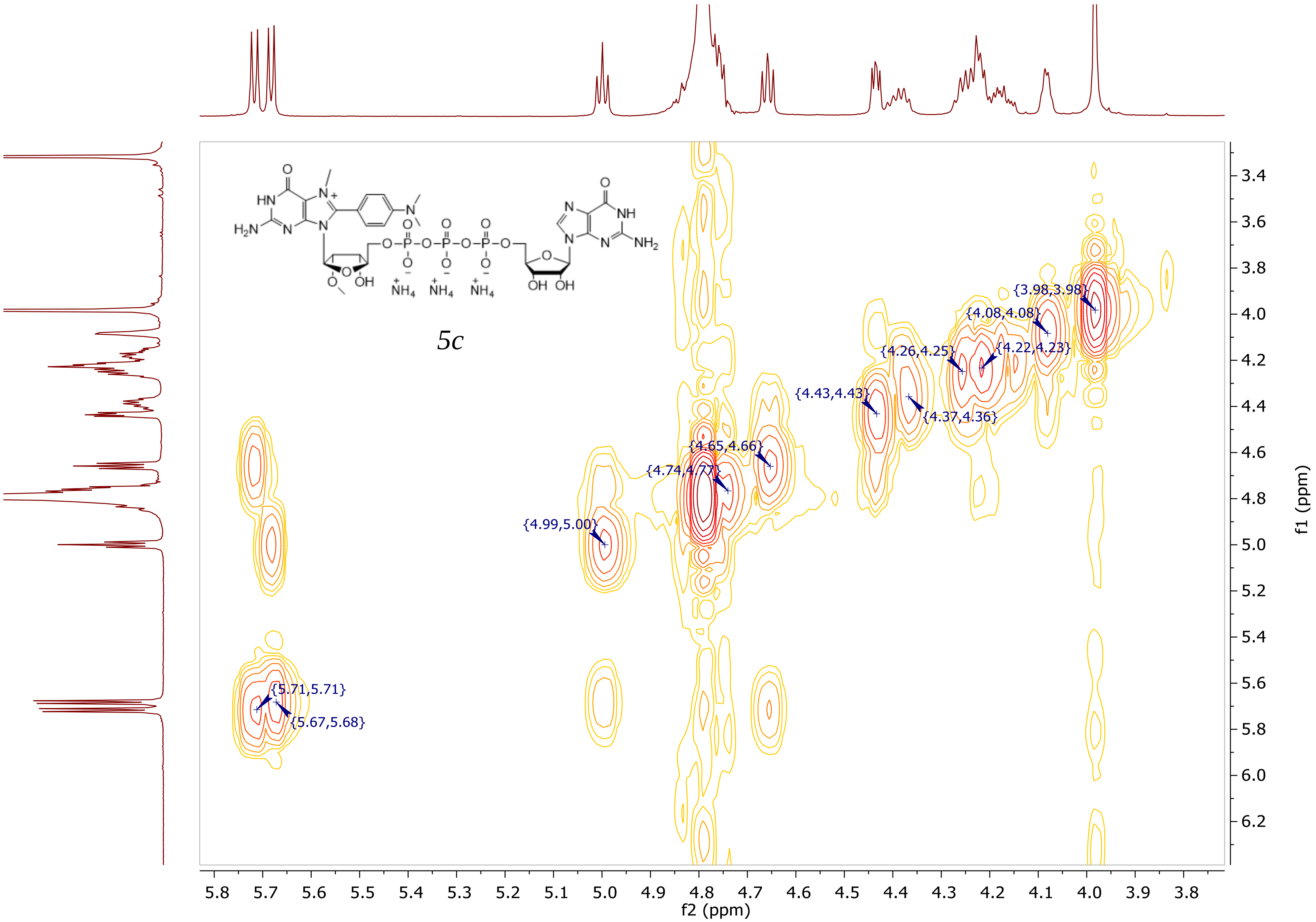

31P NMR

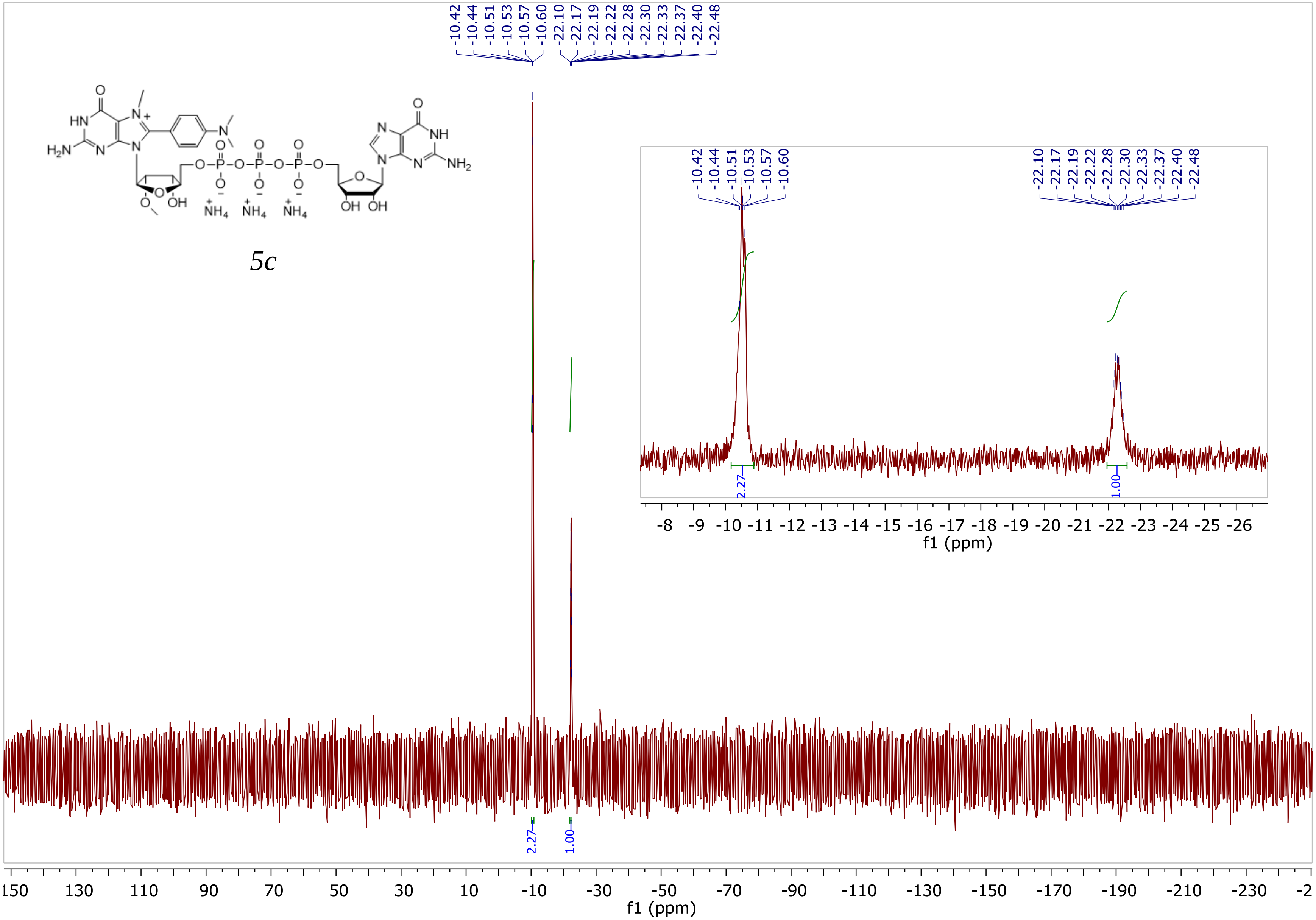
-10.42
-10.44
-10.51
-10.53
-10.57
-10.60
-22.10
-22.17
-22.19
-22.22
-22.28
-22.30
-22.33
-22.37
-22.40
-22.48
5c
2.27
1.00
-8 -9 -10 -11 -12 -13 -14 -15 -16 -17 -18 -19 -20 -21 -22 -23 -24 -25 -26
f1 (ppm)
150 130 110 90 70 50 30 10 -10 -30 -50 -70 -90 -110 -130 -150 -170 -190 -210 -230 -2
f1 (ppm)

HRMS

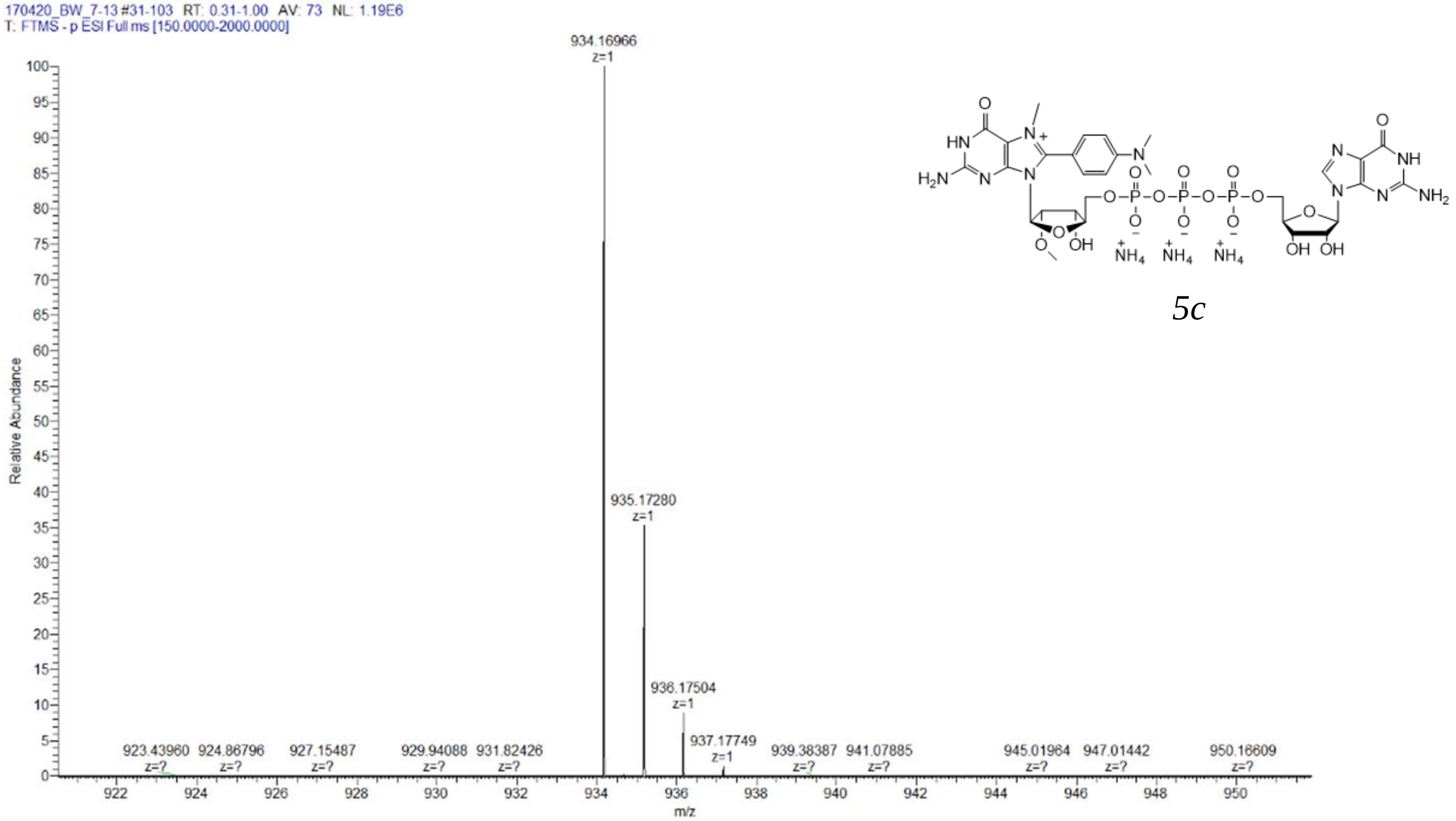

5c

Summary

Rt (B) = 13.44 min; ^{1}H NMR (500 MHz, deuterium oxide) δ 8.10 (bs, 1H), 7.43 (d, *J* = 8.6 Hz, 2H), 6.91 (d, *J* = 8.6 Hz, 2H), 5.72 (d, *J* = 6.3 Hz, 1H), 5.68 (d, *J* = 5.7 Hz, 1H), 5.00 (t, *J* = 5.7 Hz, 1H), 4.76 (dd, *J* = 5.7 Hz, 3.9 Hz, 1H, overlapped with solvent signal), 4.66 (dd, *J* = 6.3 Hz, 5.1 Hz, 1H), 4.43 (dd, *J* = 5.1 Hz, 3.1 Hz, 1H), 4.39 (m, 1H), 4.24 (m, 3H), 4.17 (m, 1H), 4.08 (m, 1H), 3.98 (s, 3H), 3.32 (s, 3H), 3.05 (s, 6H); ^{31}P NMR (202 MHz, deuterium oxide) δ –10.22 to –10.70 (m, 2P), –22.00 to –22.58 (m, 1P); HRMS ESI (-) m/z [M-H]$^{-}$, calcd for $C_{30}H_{39}N_{11}O_{18}P_3^{-}$ [M-H]$^{-}$ 934.1693; found 934.1697.

$^{8PhCN}m^{2'O,7}GpppG$ ***(5d)***

Structure

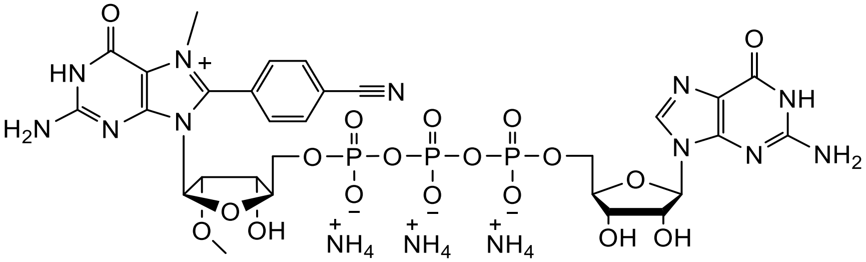

RP-HPLC profile

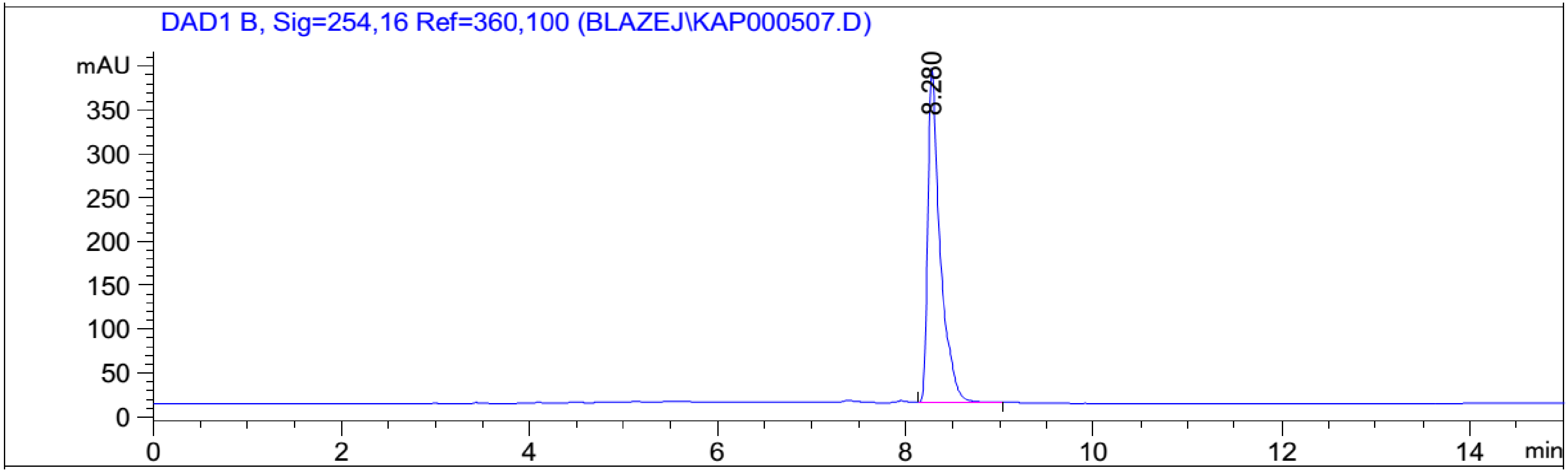

1H NMR

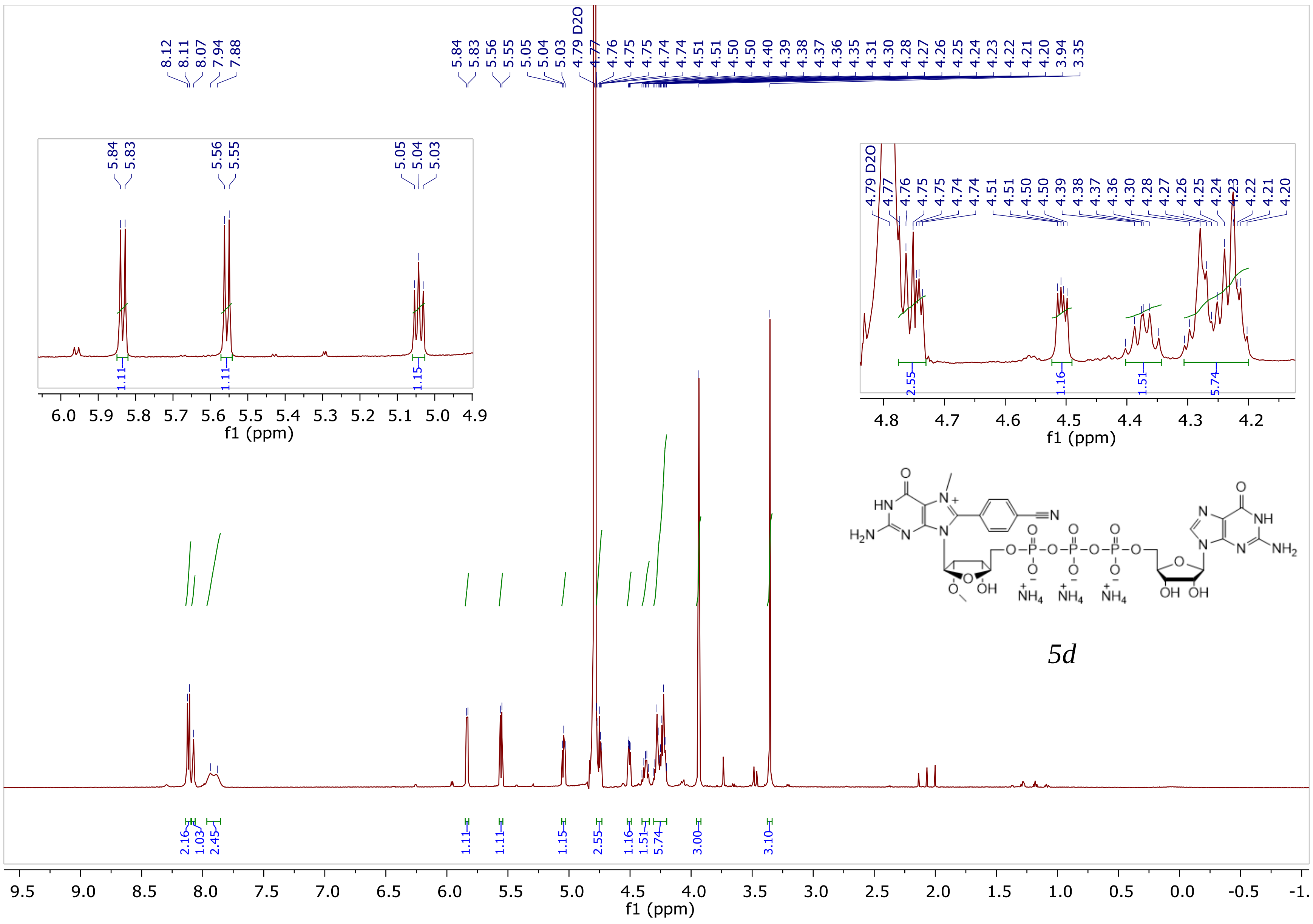

5d

1H-1H COSY NMR

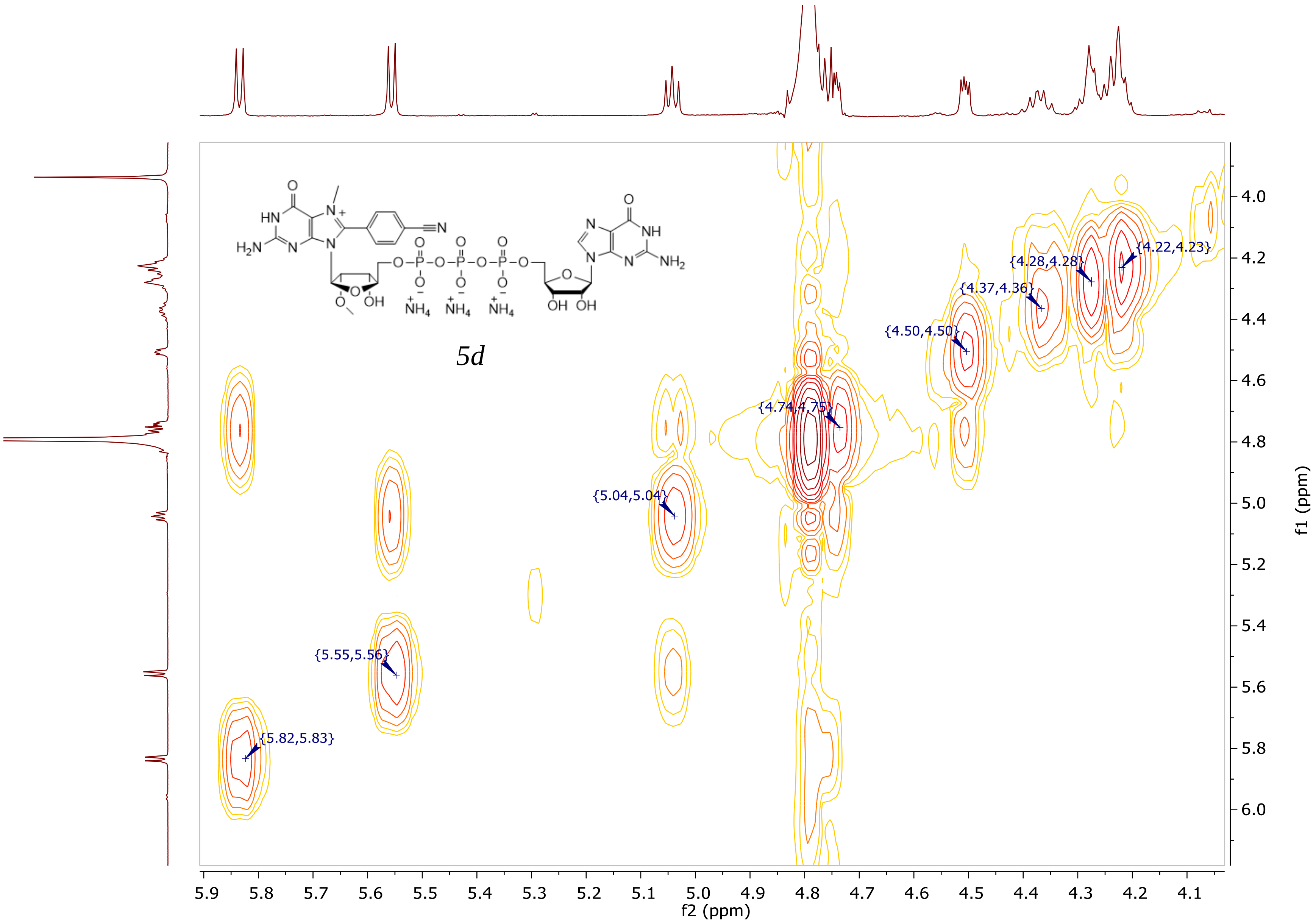
5d
{5.82,5.83}
{5.55,5.56}
{5.04,5.04}
{4.74,4.75}
{4.50,4.50}
{4.37,4.36}
{4.28,4.28}
{4.22,4.23}
f2 (ppm)
f1 (ppm)

31P NMR

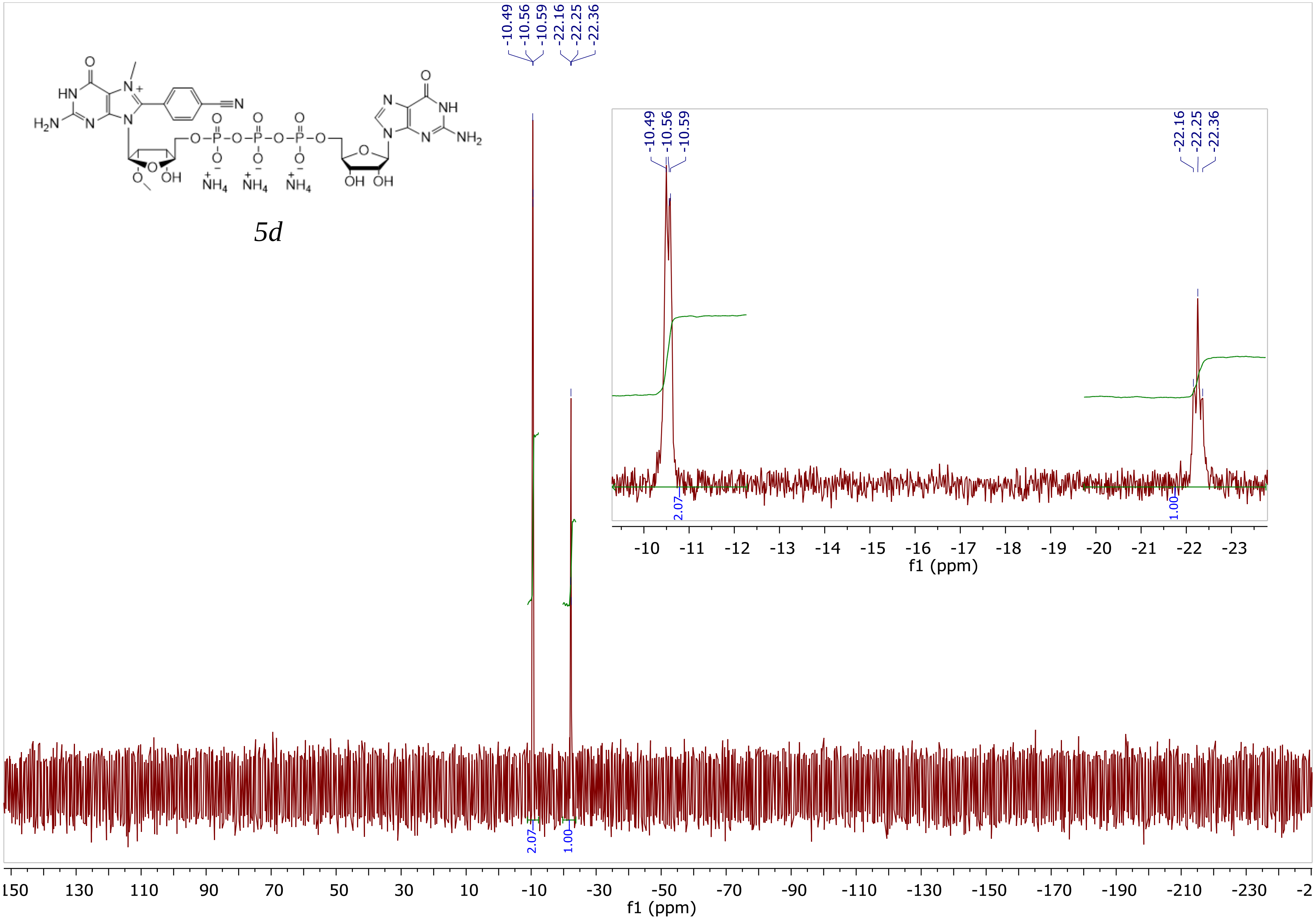

HRMS

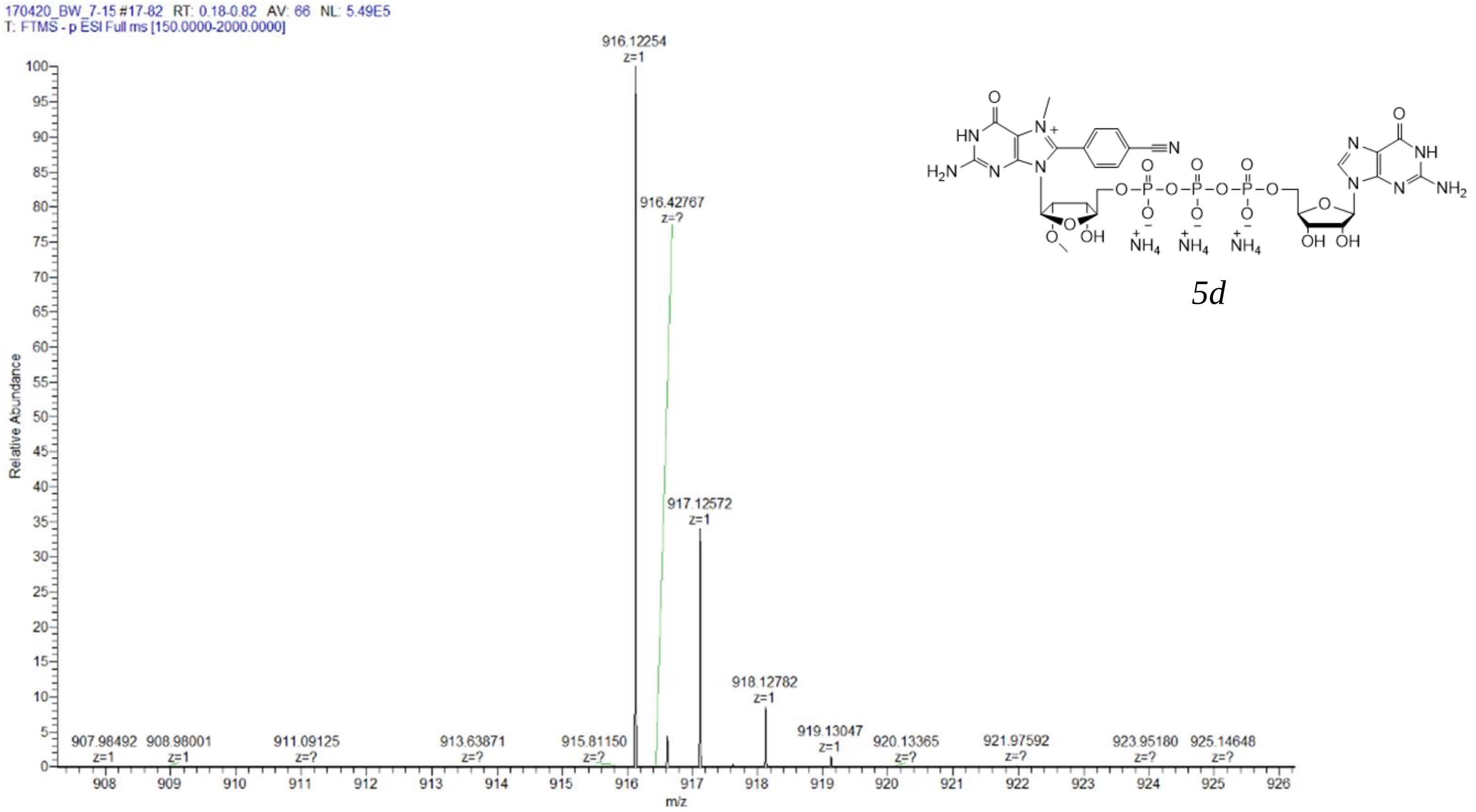

5d

Summary

Rt (B) = 8.28 min; ^{1}H NMR (500 MHz, deuterium oxide) δ 8.14–8.10 (m, 2H), 8.07 (s, 1H), 7.98–7.84 (m, 2H), 5.83 (d, *J* = 6.2 Hz, 1H), 5.56 (d, *J* = 6.0 Hz, 1H), 5.04 (dd, *J* = 6.0 Hz, 5.2 Hz, 1H), 4.77–4.73 (m, 2H), 4.51 (dd, *J* = 5.2 Hz, 2.8 Hz, 1H), 4.37 (m, 2H), 4.25 (m, 4H), 3.94 (s, 3H), 3.35 (s, 3H); ^{31}P NMR (202 MHz, deuterium oxide) δ –10.42 to –10.70 (m, 2P), –22.25 (t, *J* = 19.5 Hz, 1P); HRMS ESI (-) m/z [M-H]$^-$, calcd for $C_{29}H_{33}N_{11}O_{18}P_3^-$ [M-H]$^-$ 916.1223; found 916.1225.

$^{8Me}m^{2'O,7}$GpppG (5e)

Structure

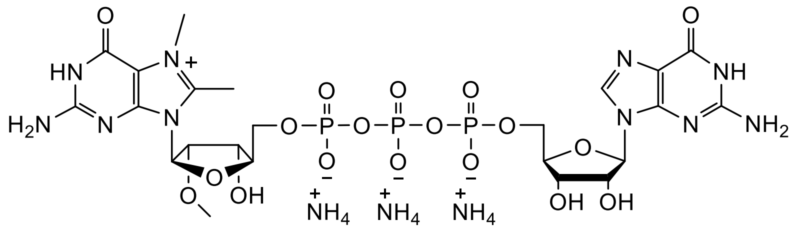

RP-HPLC profile

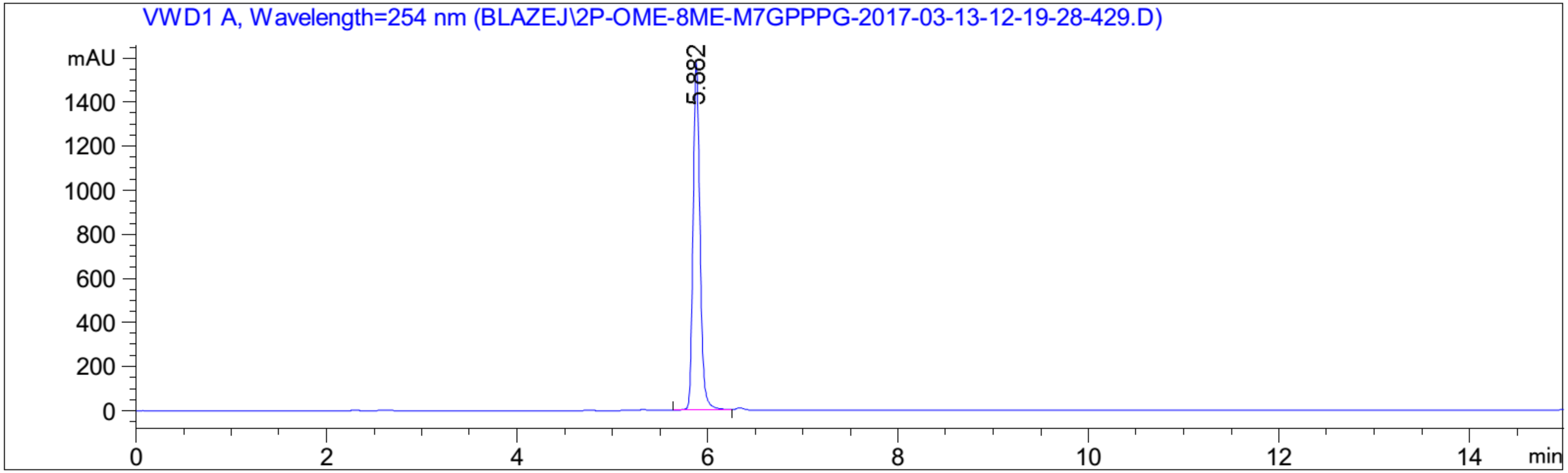

1H NMR

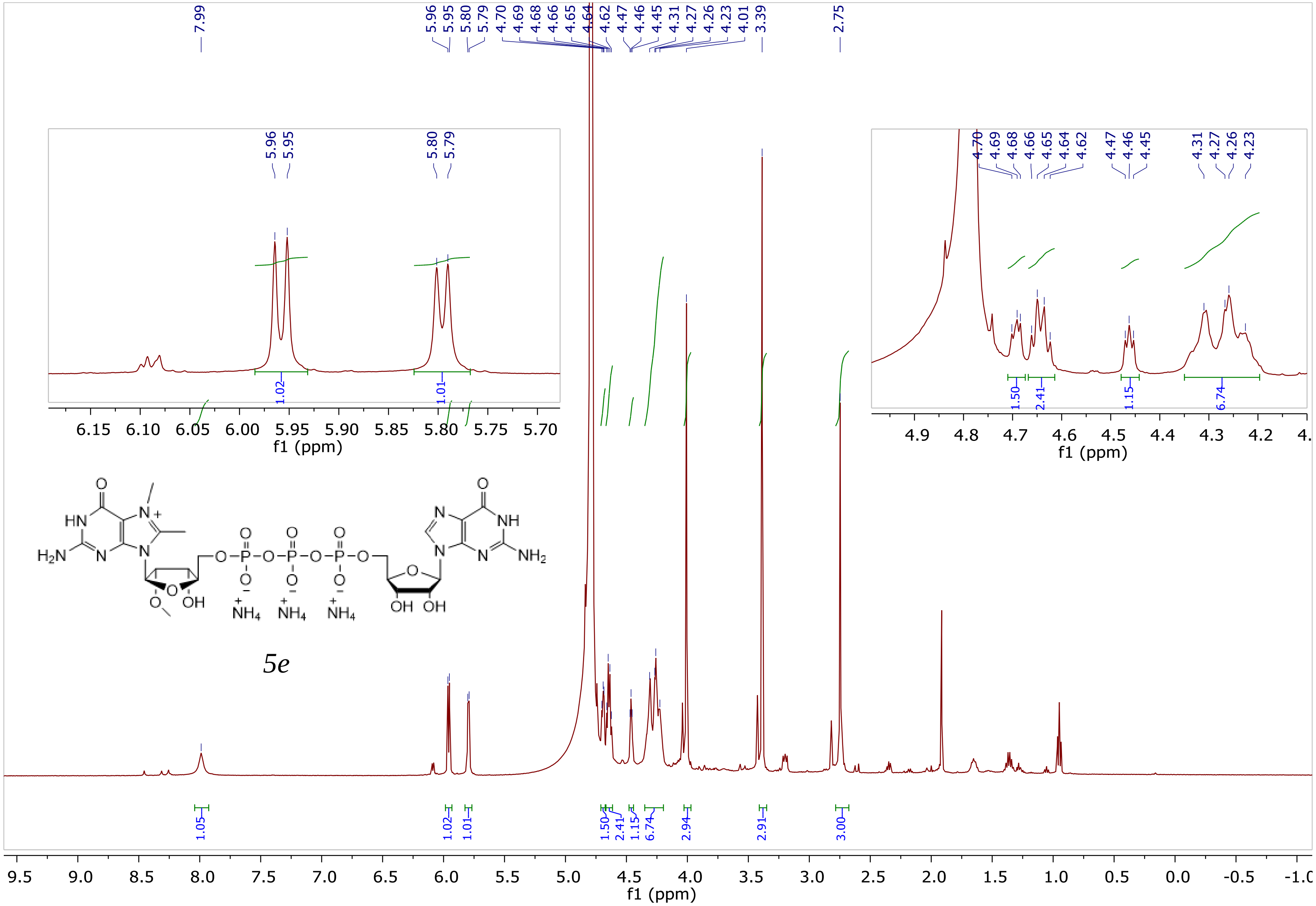

7.99
5.96
5.95
5.80
5.79
4.70
4.69
4.68
4.66
4.65
4.64
4.62
4.47
4.46
4.45
4.31
4.27
4.26
4.23
4.01
3.39
2.75
1.05
1.02
1.01
1.50
2.41
1.15
6.74
2.94
2.91
3.00
f1 (ppm)
5e

31P NMR

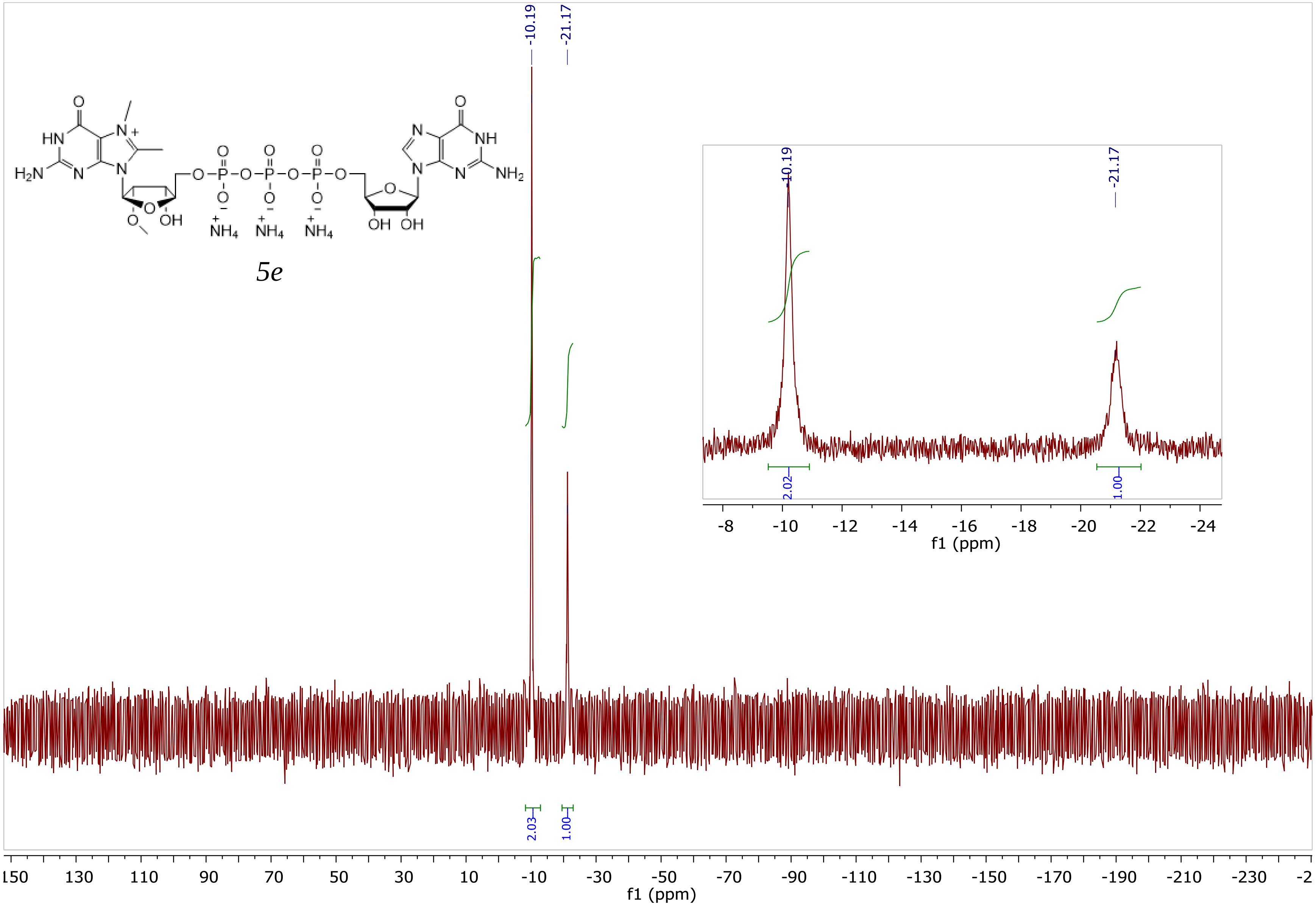

HRMS

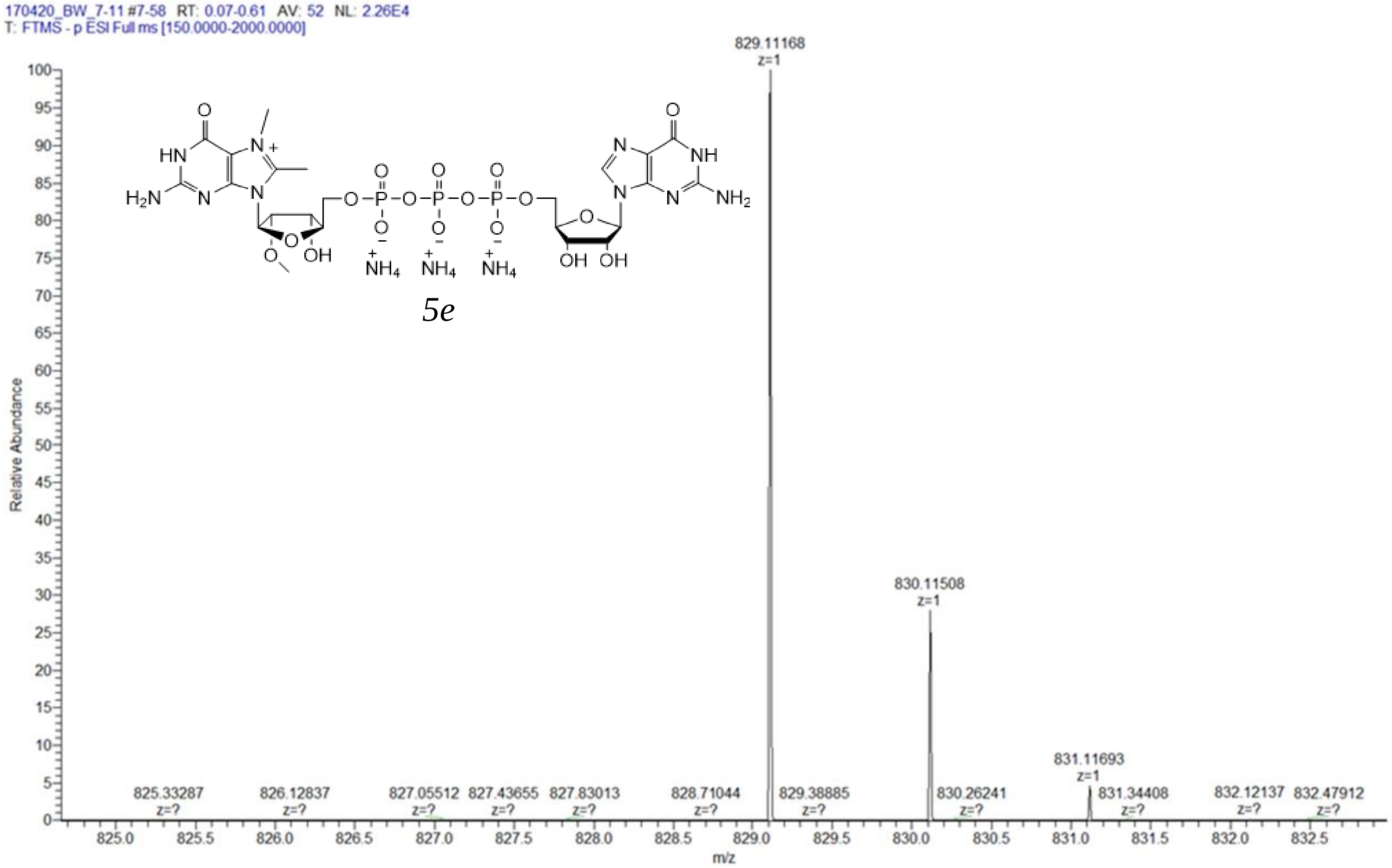

Summary

Rt (A) = 5.88 min; ^{1}H NMR (500 MHz, deuterium oxide) δ 7.99 (s, 1H), 5.96 (d, *J* = 6.2 Hz, 1H), 5.80 (d, *J* = 5.6 Hz, 1H), 4.69 (m, 1H), 4.64 (m, 2H), 4.46 (m, 1H), 4.27 (m, 6H), 4.01 (s, 3H), 3.39 (s, 3H), 2.75 (s, 3H); ^{31}P NMR (202 MHz, deuterium oxide) δ –9.72 to –10.71 (m, 2P), –20.60 to –21.65 (m, 1P); HRMS ESI (-) m/z [M-H]$^-$, calcd for $C_{23}H_{32}N_{10}O_{18}P_3^-$ [M-H]$^-$ 829.1114; found 829.1117.

$^{8cPr}m^{2'O,7}$GpppG (5f)

Structure

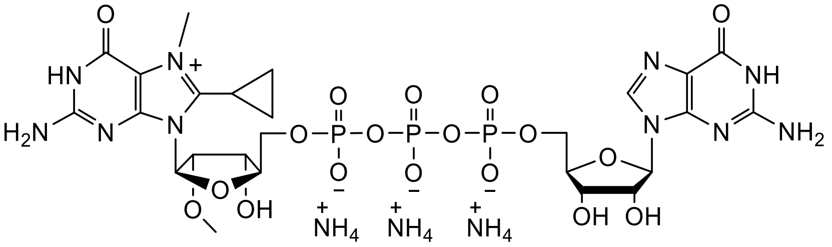

RP-HPLC profile

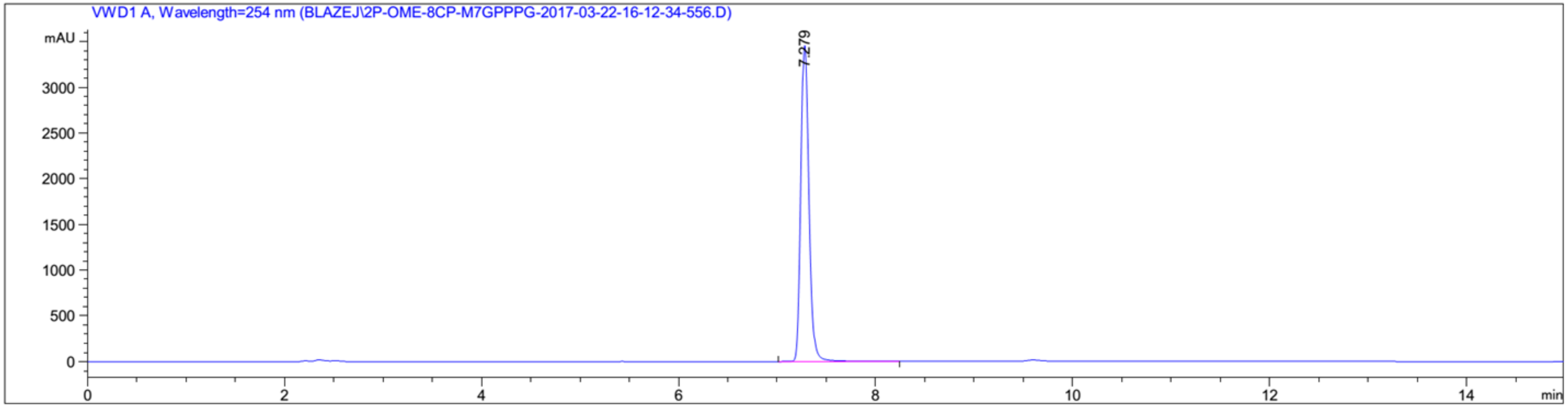

1H NMR

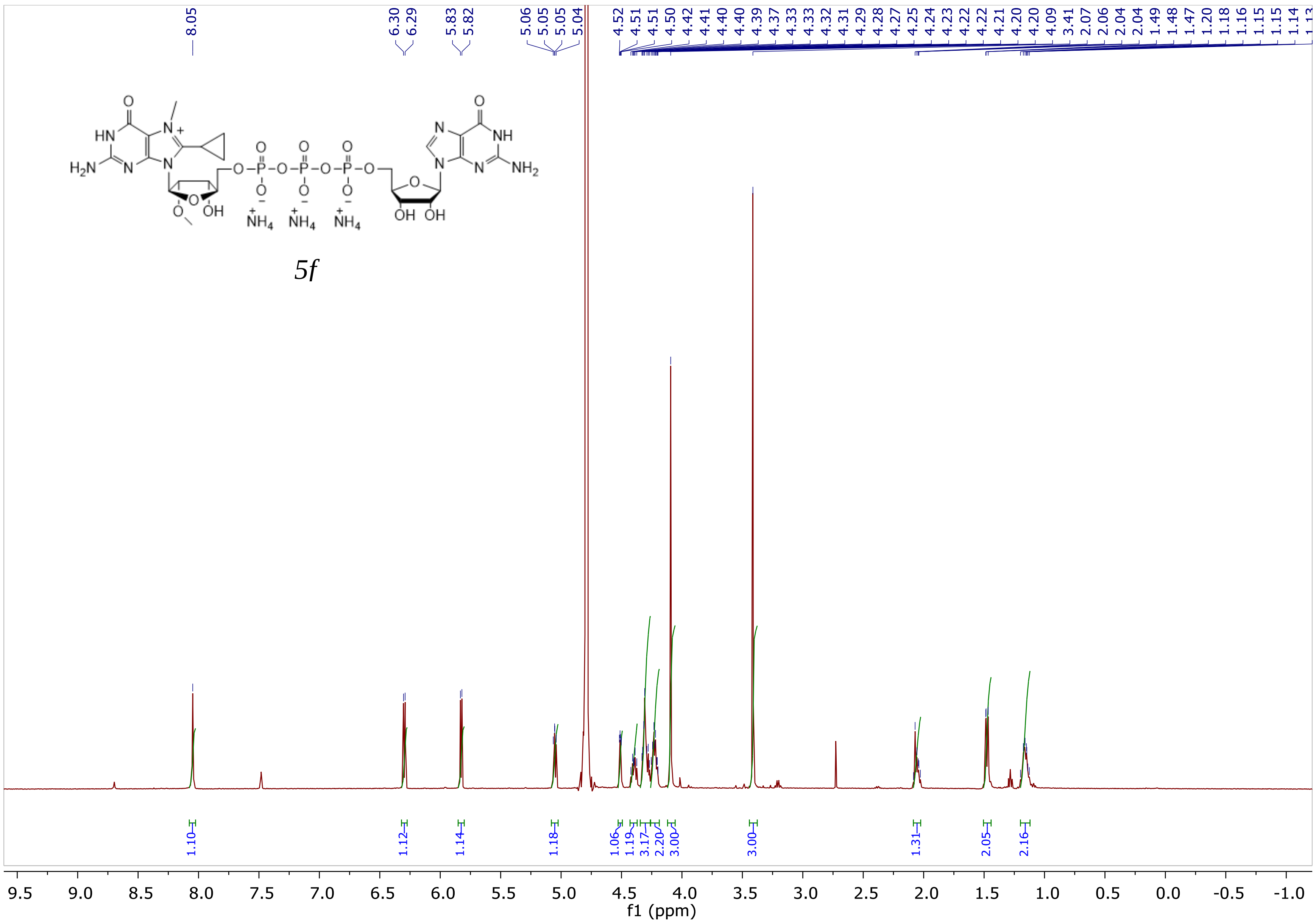

1H-1H COSY NMR

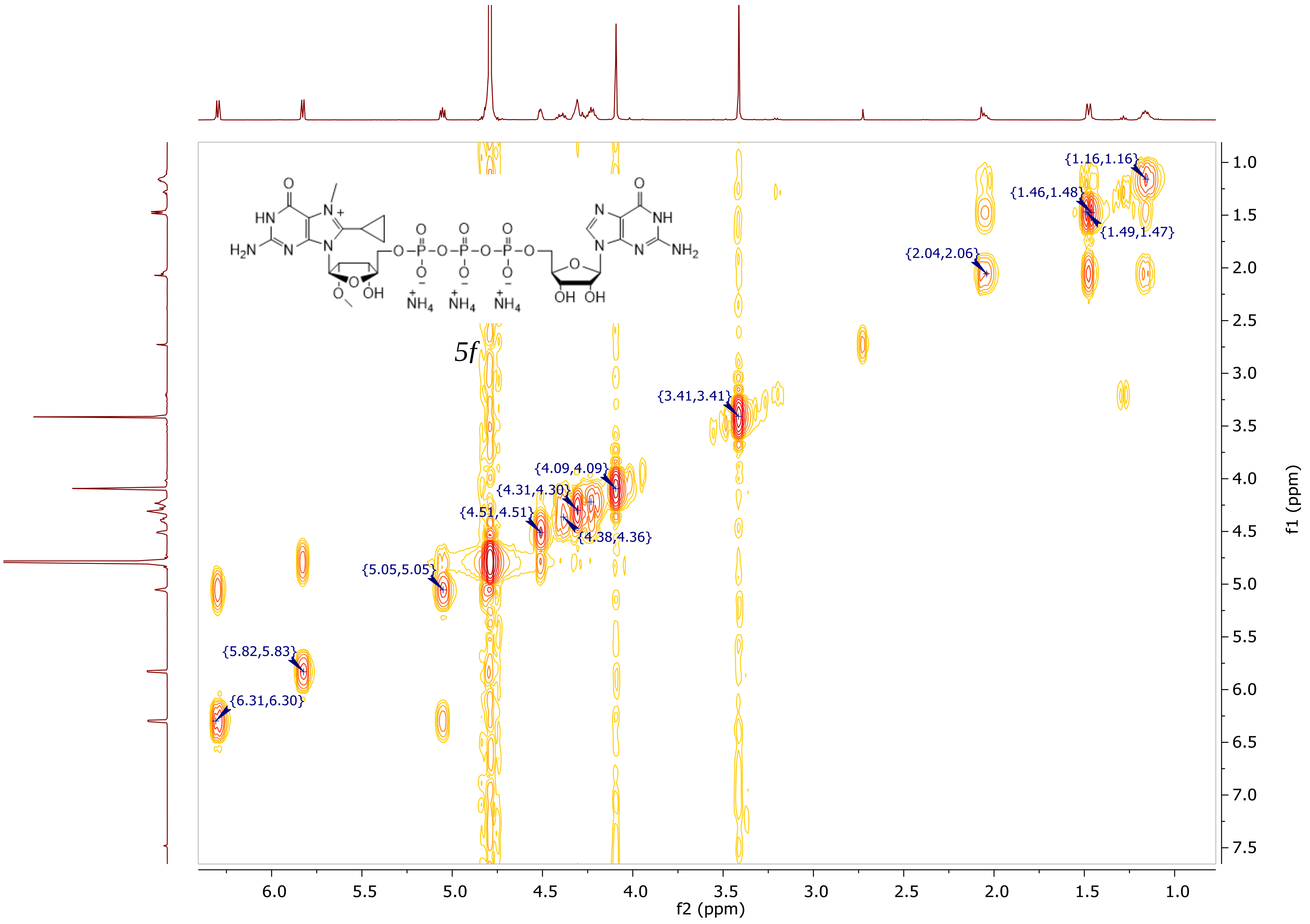
5f
{1.16,1.16}
{1.46,1.48}
{1.49,1.47}
{2.04,2.06}
{3.41,3.41}
{4.09,4.09}
{4.31,4.30}
{4.51,4.51}
{4.38,4.36}
{5.05,5.05}
{5.82,5.83}
{6.31,6.30}
f2 (ppm)
f1 (ppm)

31P NMR

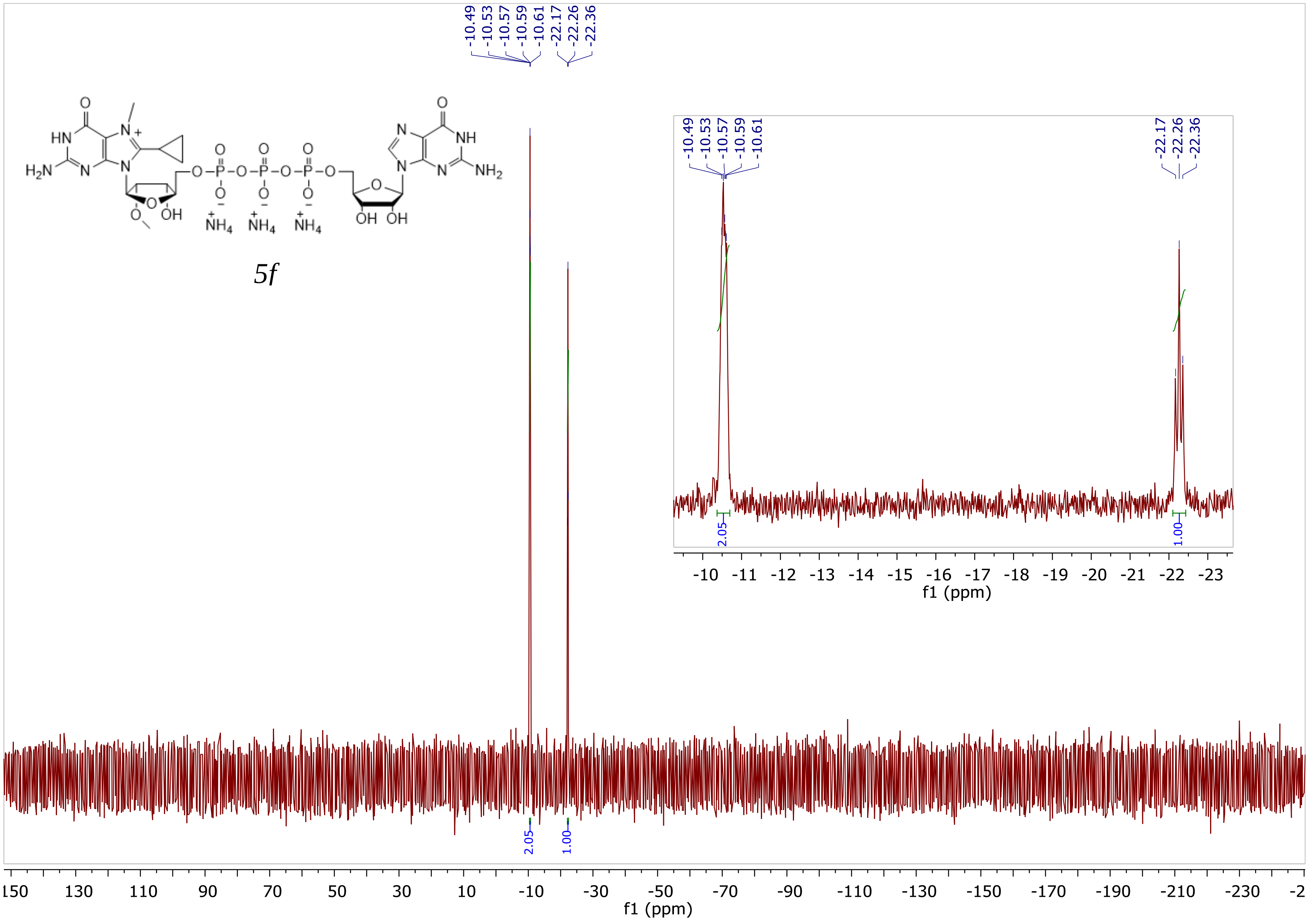

HRMS

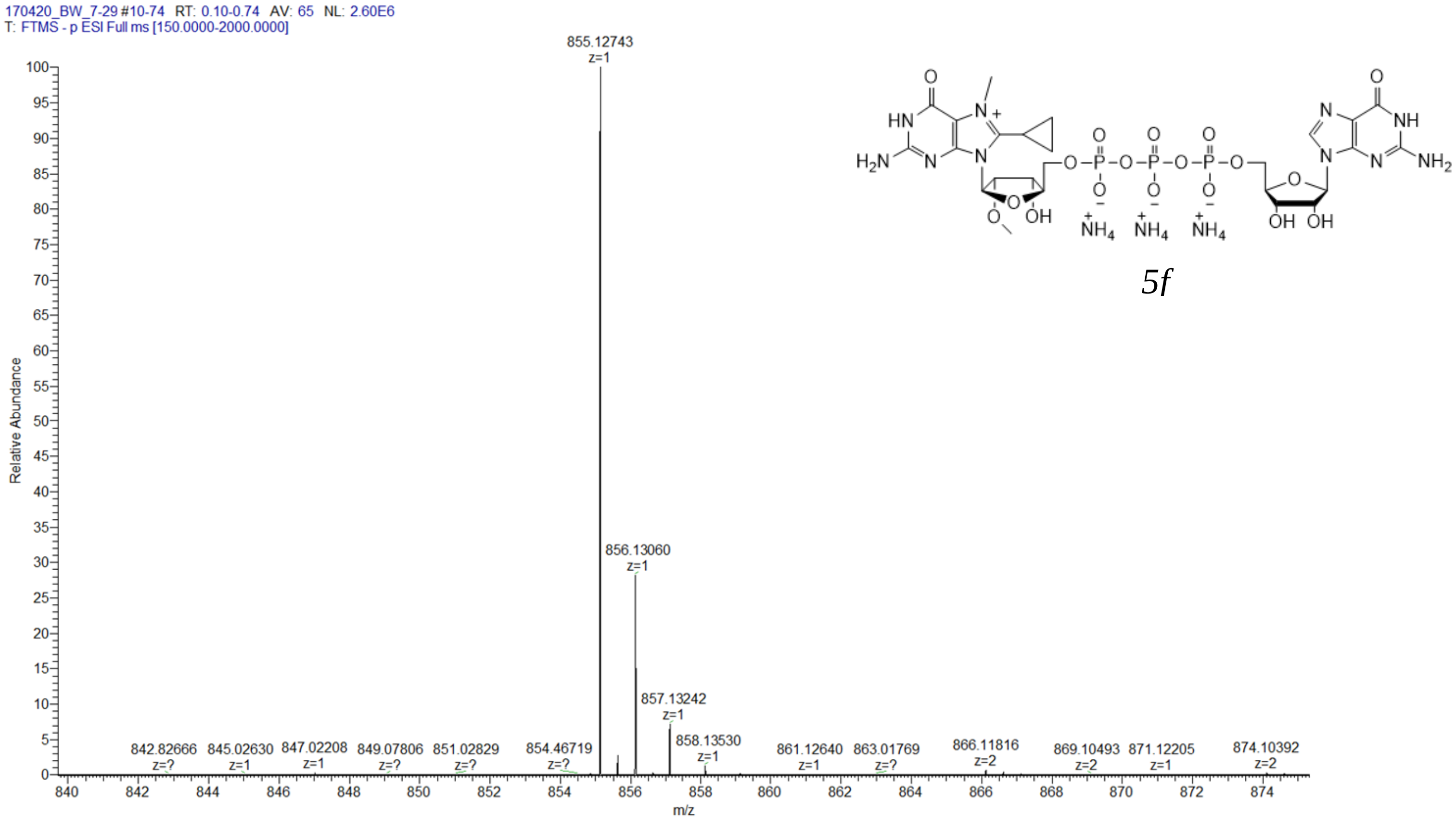

5f

Summary

Rt (A) = 7.28 min; ^{1}H NMR (500 MHz, deuterium oxide) δ 8.05 (s, 1H), 6.30 (d, *J* = 6.7 Hz, 1H), 5.83 (d, *J* = 6.2 Hz, 1H), 5.05 (dd, *J* = 6.7 Hz, 5.2 Hz, 1H), 4.85–4.77 (m, 2H, overlapped with solvent signal), 4.51 (dd, *J* = 5.5 Hz, 2.8 Hz, 1H), 4.40 (dt, *J* = 10.8 Hz, 6.7 Hz, 1H), 4.33–4.26 (m, 3H), 4.25–4.19 (m, 2H), 4.09 (s, 3H), 3.41 (s, 3H), 2.09–2.02 (m, 1H), 1.51–1.44 (m, 2H), 1.20–1.12 (m, 2H); ^{31}P NMR (202 MHz, deuterium oxide) δ –10.40 to –10.72 (m, 2P), –22.10 to –21.45 (m, 1P); HRMS ESI (-) m/z [M-H]$^-$, calcd for $C_{25}H_{34}N_{10}O_{18}P_3^-$ [M-H]$^-$ 855.1271; found 855.1274.

$^{8DMAPh}m^7Gpppm^7G$ (6)

Structure

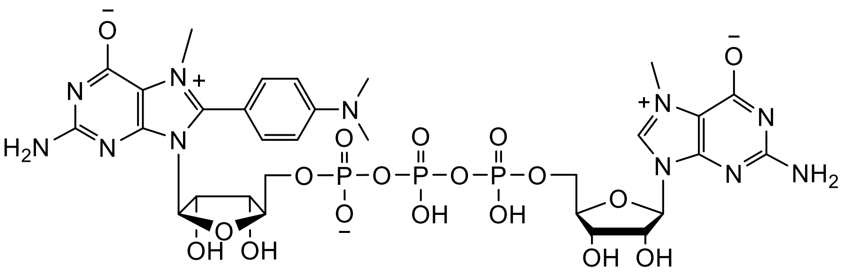

RP-HPLC profile

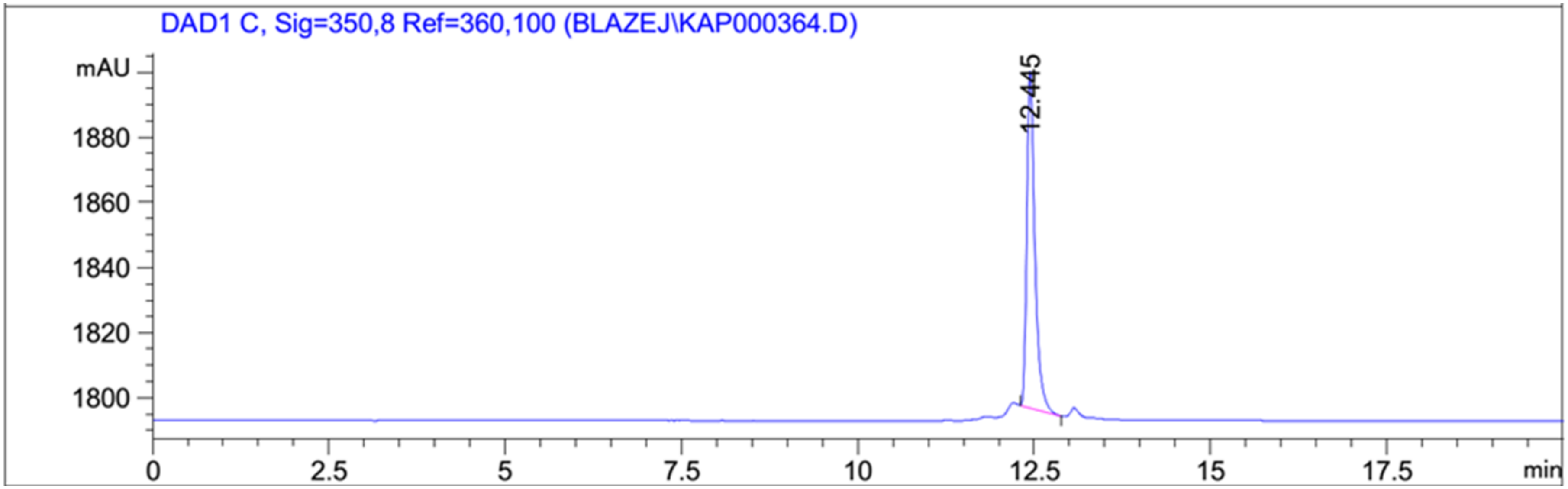

1H NMR

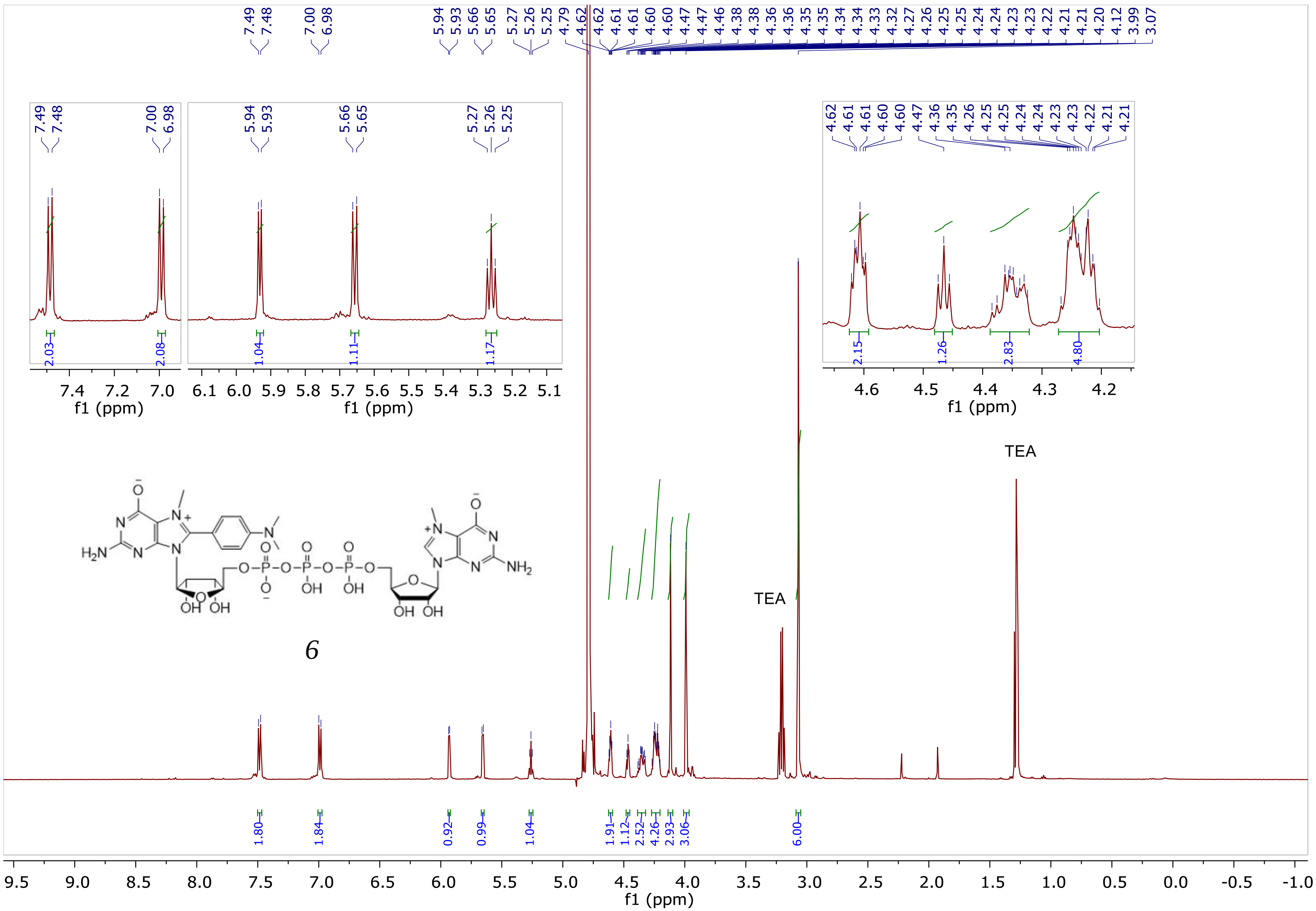

1H-1H COSY NMR

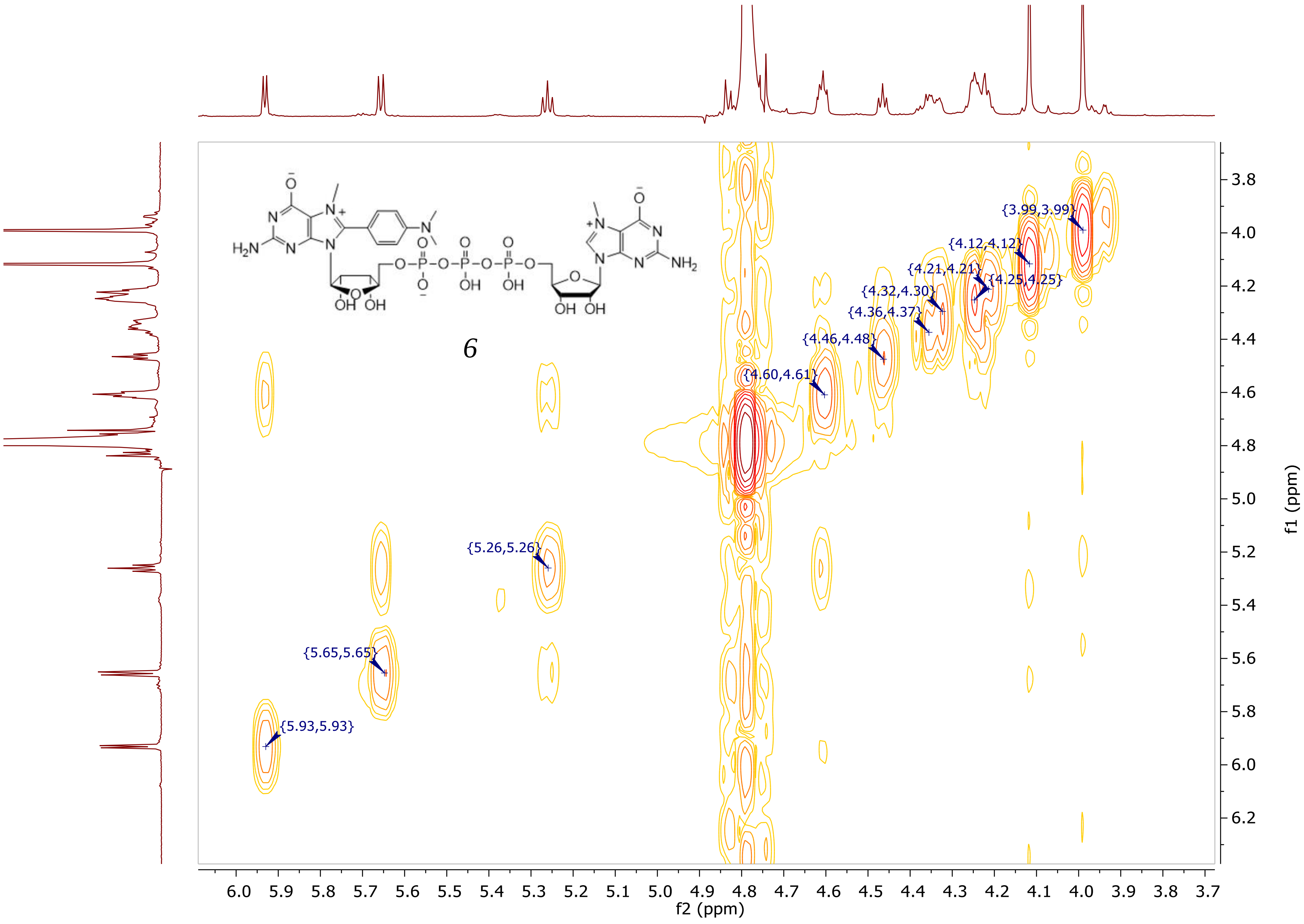
6
{3.99,3.99}
{4.12,4.12}
{4.21,4.21}
{4.25,4.25}
{4.32,4.30}
{4.36,4.37}
{4.46,4.48}
{4.60,4.61}
{5.26,5.26}
{5.65,5.65}
{5.93,5.93}
f2 (ppm)
f1 (ppm)

31P NMR

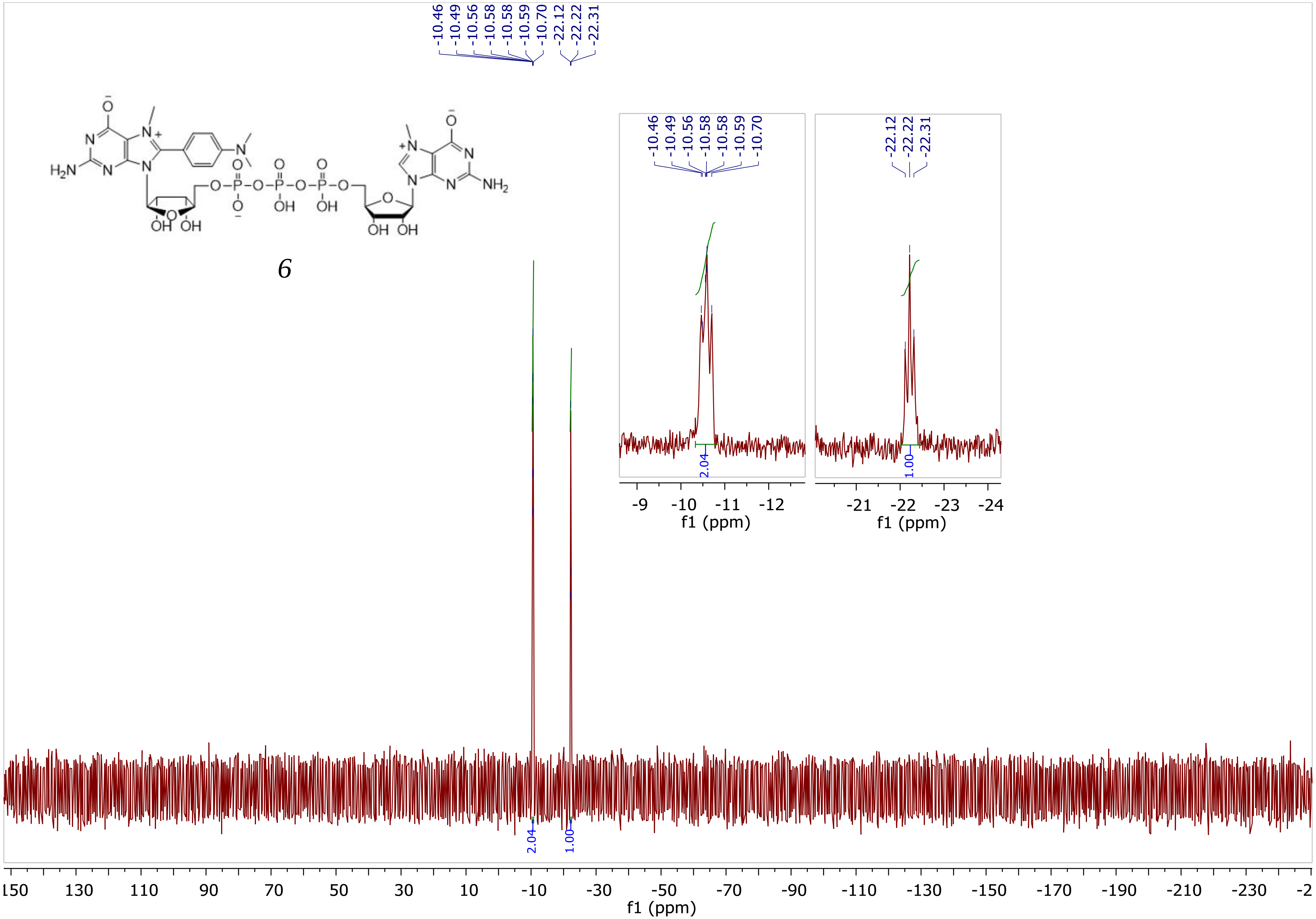
-10.46
-10.49
-10.56
-10.58
-10.58
-10.59
-10.70
-22.12
-22.22
-22.31
6
2.04
1.00
f1 (ppm)

HRMS

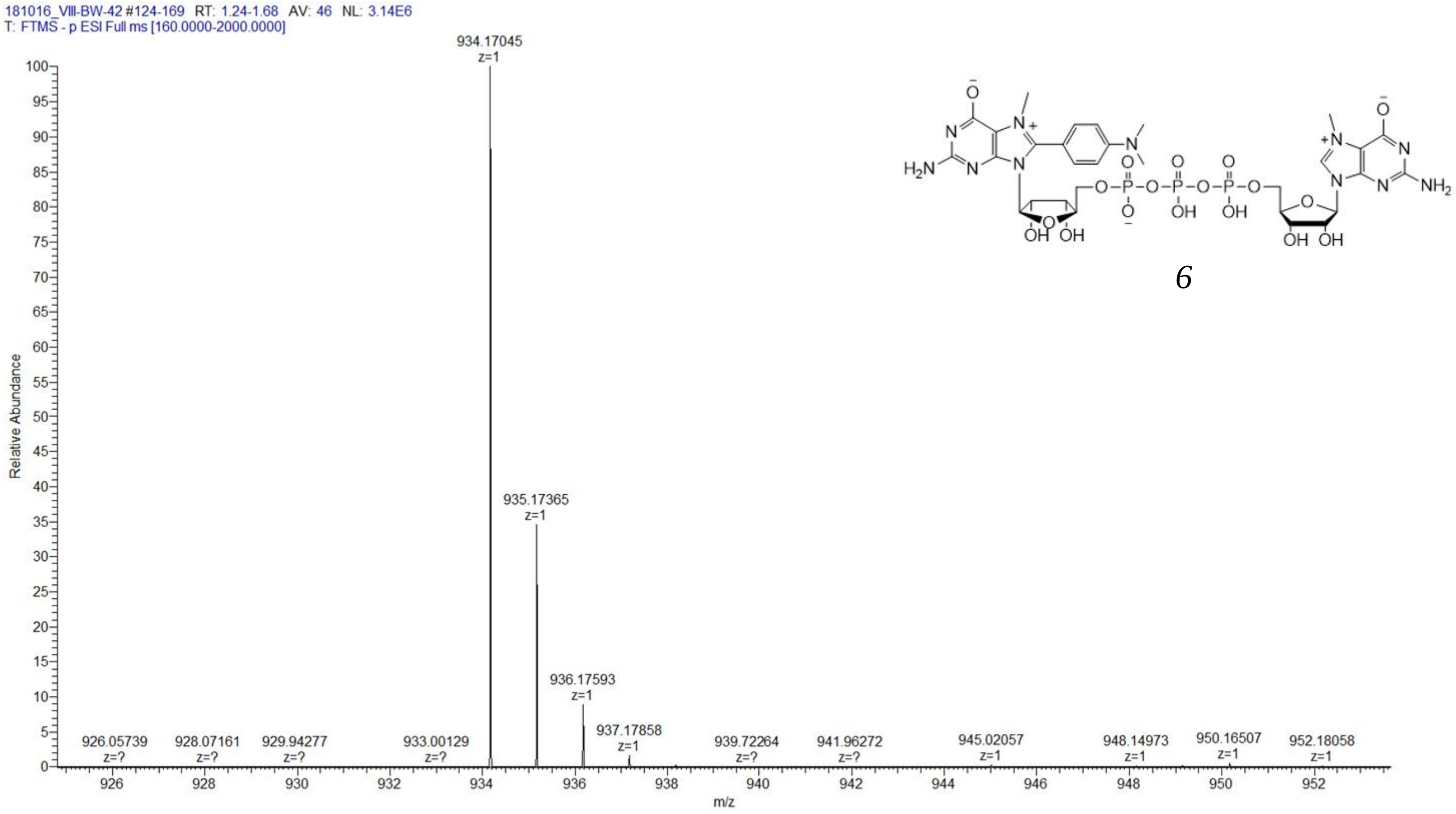

6

Summary

Rt (A) = 12.44 min; ^{1}H NMR (500 MHz, deuterium oxide) δ 7.49 (d, *J* = 8.6 Hz, 2H), 6.99 (d, *J* = 8.6 Hz, 2H), 5.93 (d, *J* = 4.2 Hz, 1H), 5.66 (d, *J* = 5.6 Hz, 1H), 5.26 (t, *J* = 5.6 Hz, 1H), 4.60–4.58 (m, 2H), 4.47 (t, *J* = 4.8 Hz, 1H), 4.39–4.32 (m, 2H), 4.23 (m, 4H), 4.12 (s, 3H), 3.99 (s, 3H), 3.07 (s, 6H); ^{31}P NMR (202 MHz, deuterium oxide) δ –10.40 to –10.60 (m, 2P), –22.22 (t, *J* = 19.7 Hz, 1P); HRMS ESI (-) m/z [M-H]$^{-}$, calcd for $C_{30}H_{39}N_{11}O_{18}P_3^{-}$ [M-H]$^{-}$ 934.1693; found 934.1704.

$^{8DMAPh}m^7Gppppm^7G$ (7)

Structure

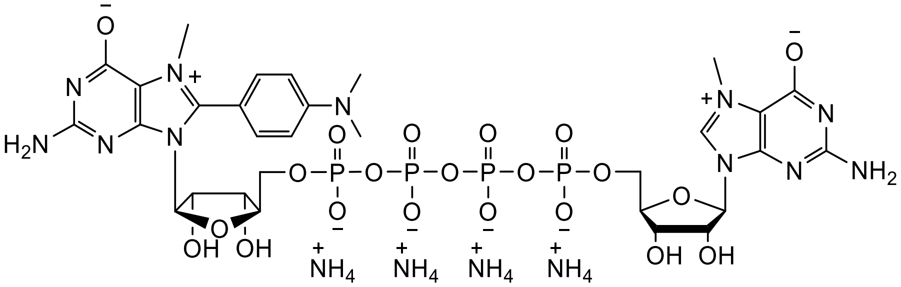

RP-HPLC profile

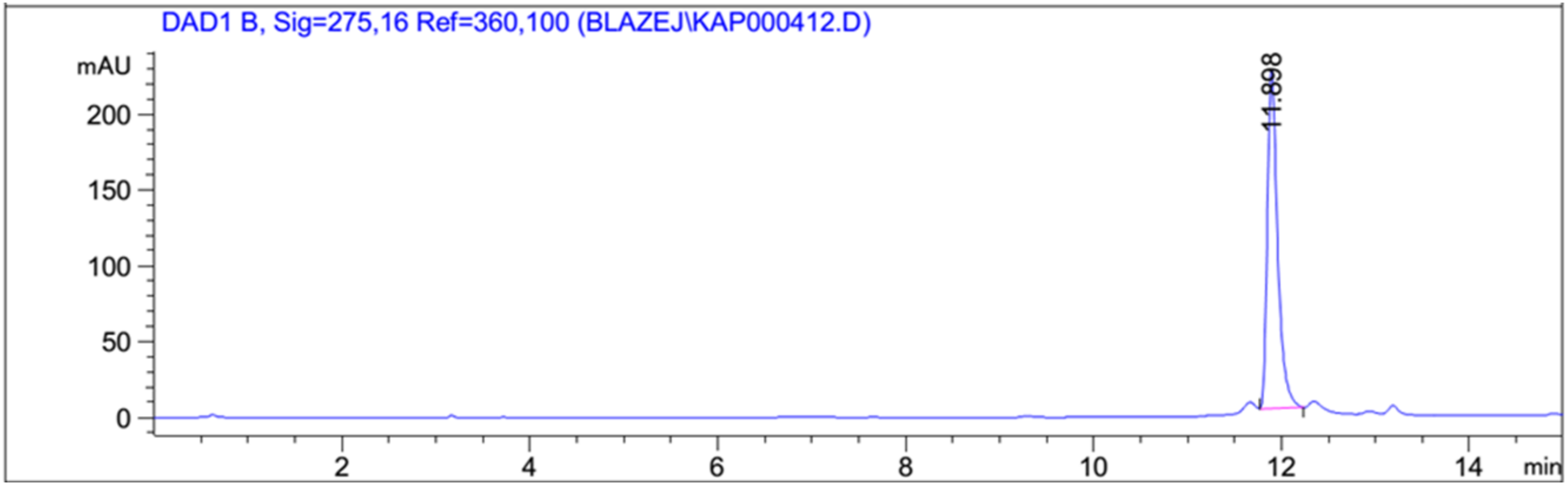

1H NMR

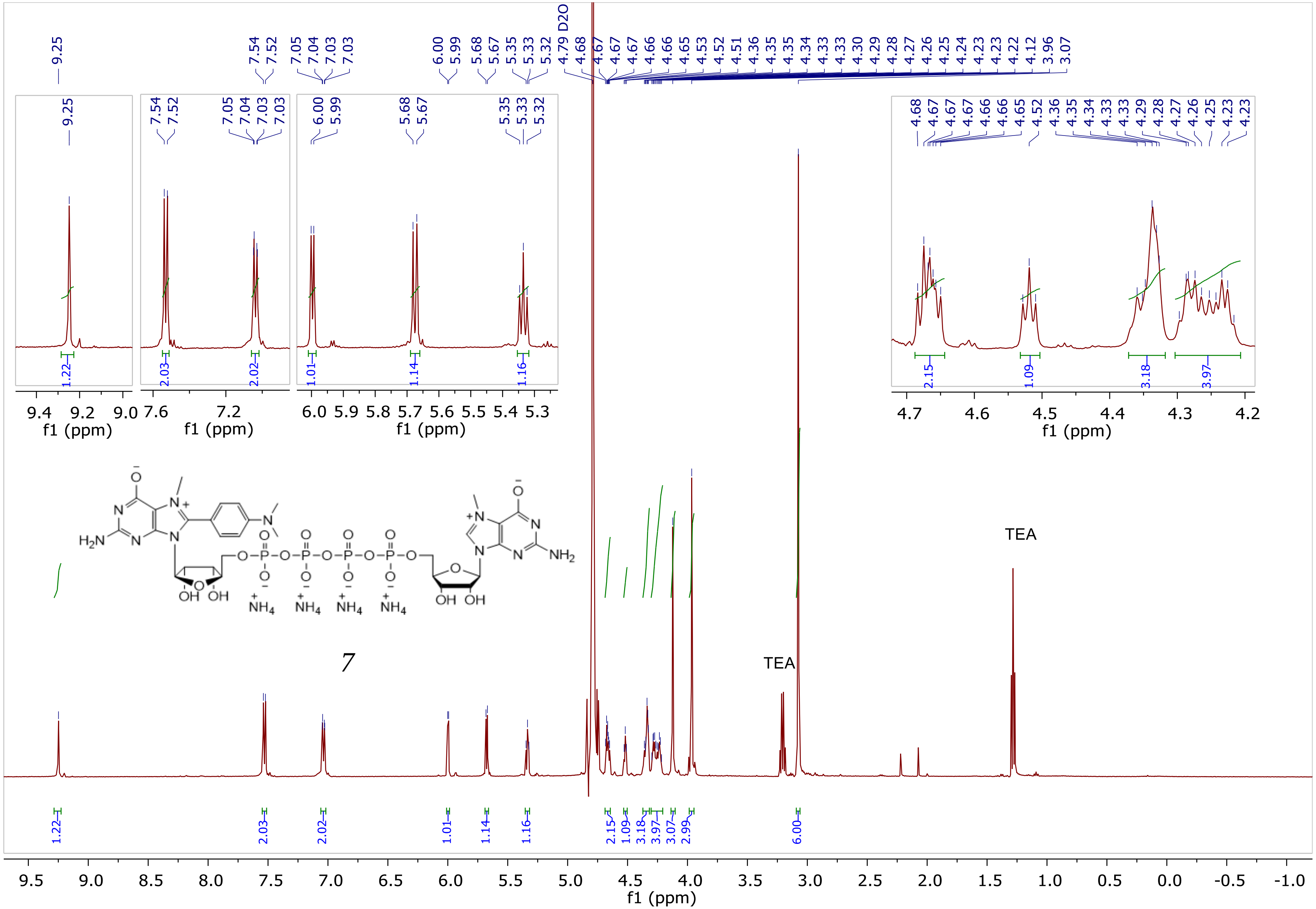

1H-1H COSY NMR

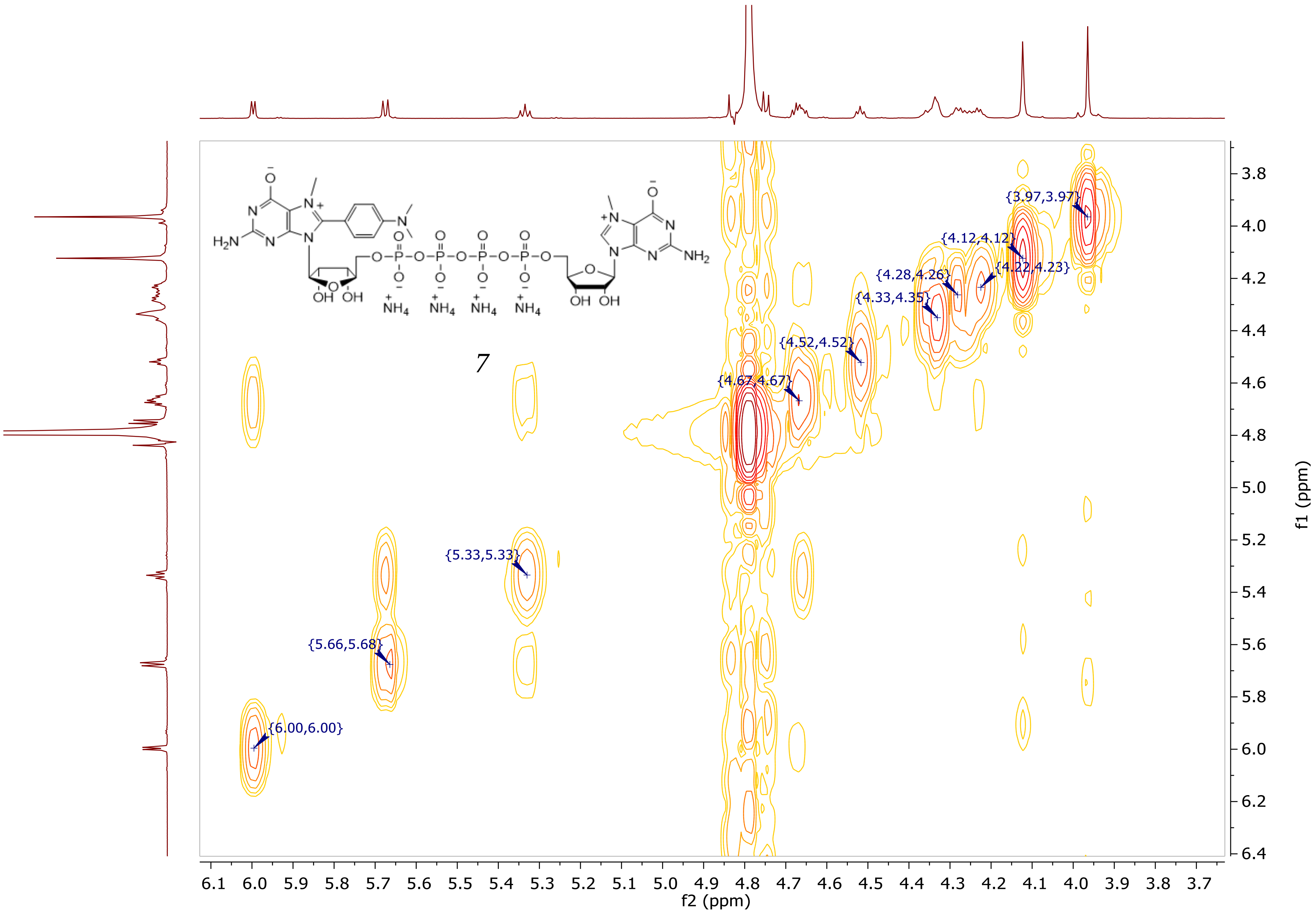
7
{3.97,3.97}
{4.12,4.12}
{4.22,4.23}
{4.28,4.26}
{4.33,4.35}
{4.52,4.52}
{4.67,4.67}
{5.33,5.33}
{5.66,5.68}
{6.00,6.00}
f2 (ppm)
f1 (ppm)

31P NMR

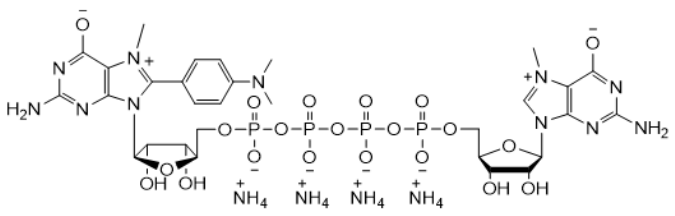

7

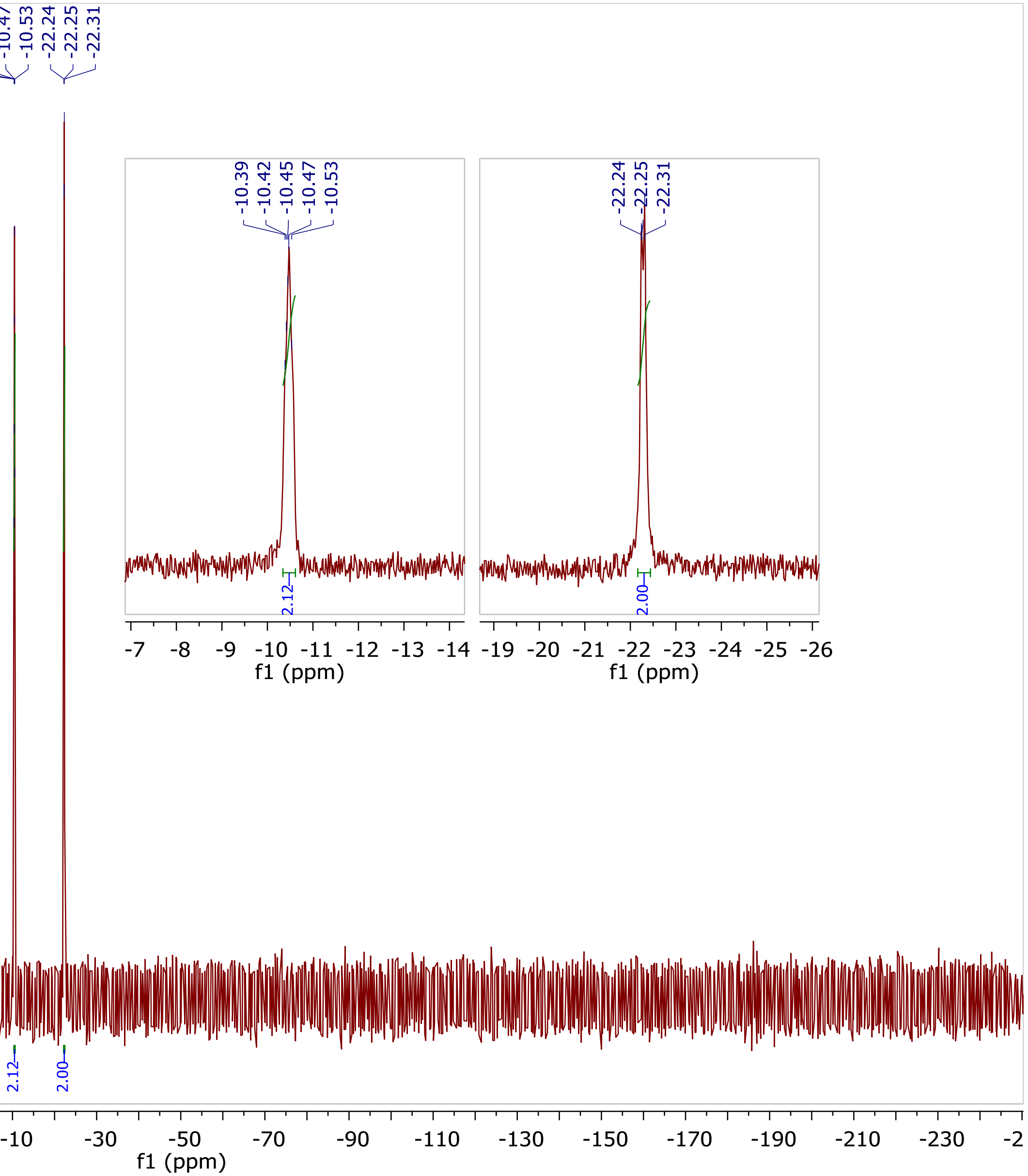

HRMS

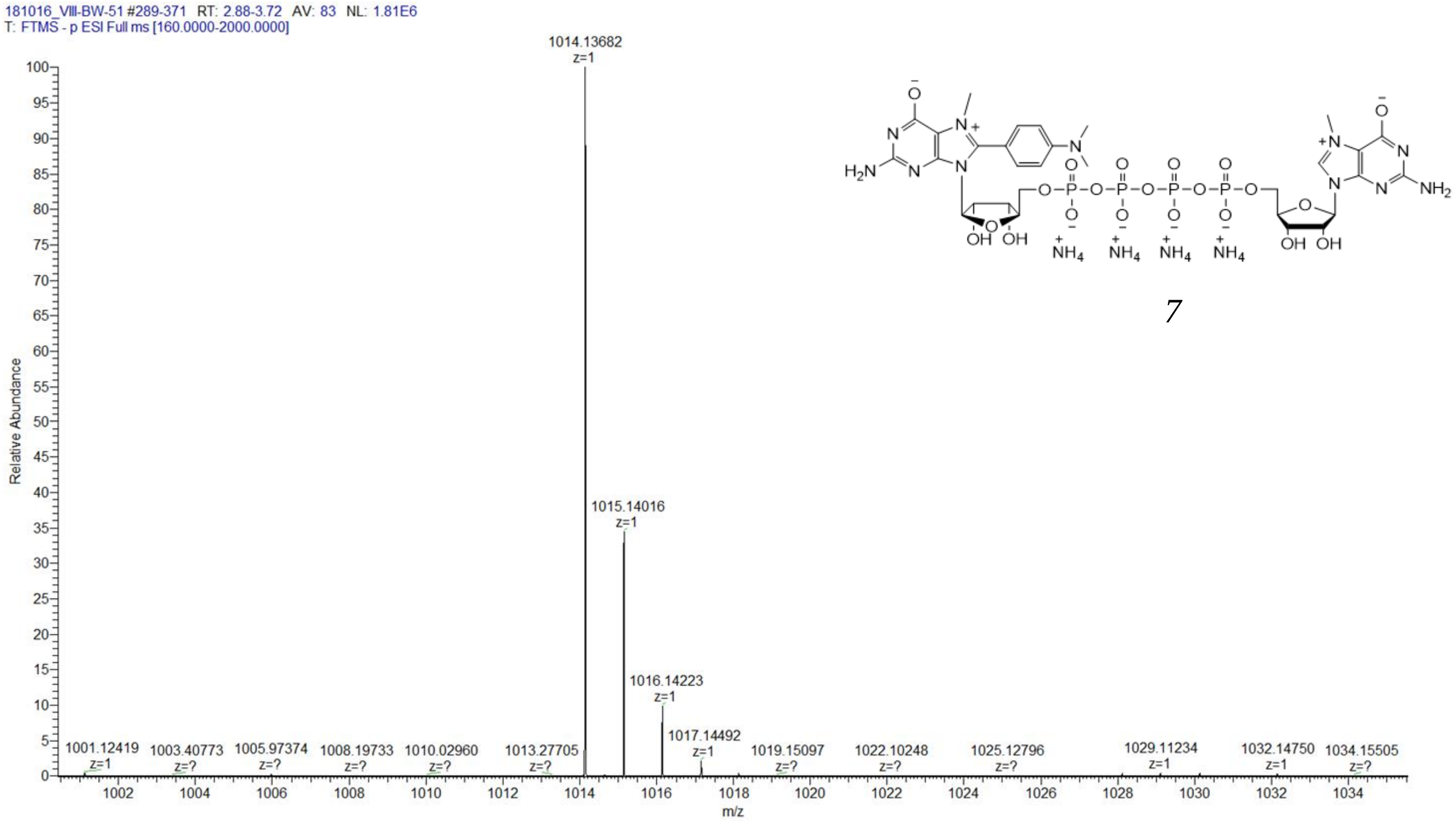

7

Summary

Rt (A) = 11.90 min; ^{1}H NMR (500 MHz, deuterium oxide) δ 9.25 (s, 1H), 7.53 (d, *J* = 8.3 Hz, 2H), 7.04 (d, *J* = 8.3 Hz, 2H), 6.00 (d, *J* = 4.2 Hz, 1H), 5.68 (d, *J* = 5.9 Hz, 1H), 5.33 (t, *J* = 5.9 Hz, 1H), 4.69-4.65 (m, 2H), 4.52 (t, *J* = 4.7 Hz, 1H), 4.40–4.30 (m, 3H), 4.28–4.20 (m, 3H), 4.12 (s, 3H), 3.96 (s, 3H), 3.07 (s, 6H); ^{31}P NMR (202 MHz, deuterium oxide) δ –10.30 to –10.60 (m, 2P), –22.15 to –22.15 (m, 2P); HRMS ESI (-) m/z [M-H]$^-$, calcd for $C_{30}H_{40}N_{11}O_{21}P_4^-$ [M-H]$^-$ 1014.1356; found 1014.1368.

$^{8DMAPh}m^7Gpppm^{2,2,7}G$ (8)

Structure

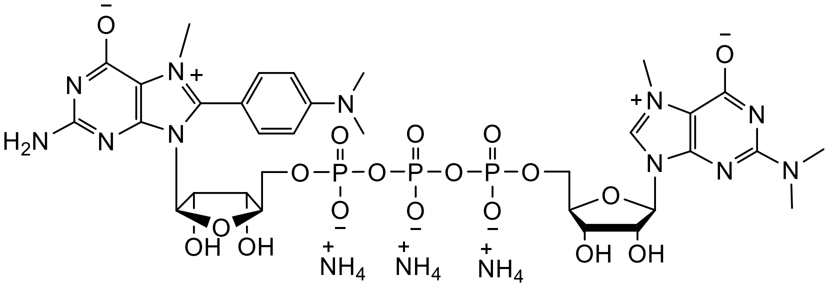

RP-HPLC profile

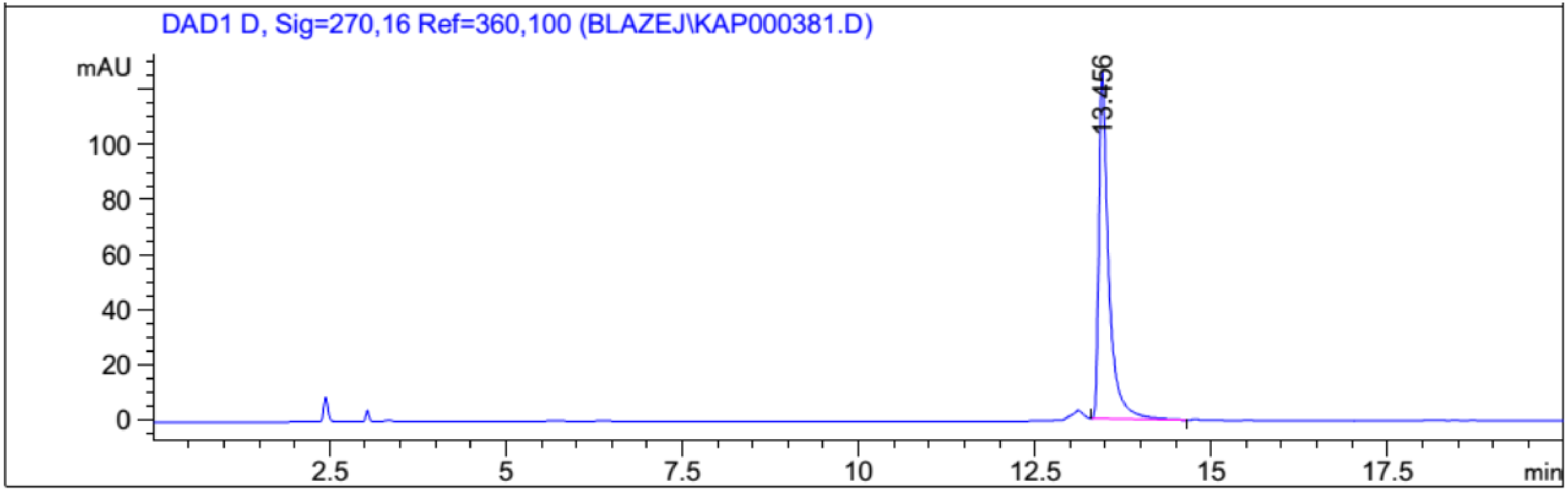

1H NMR

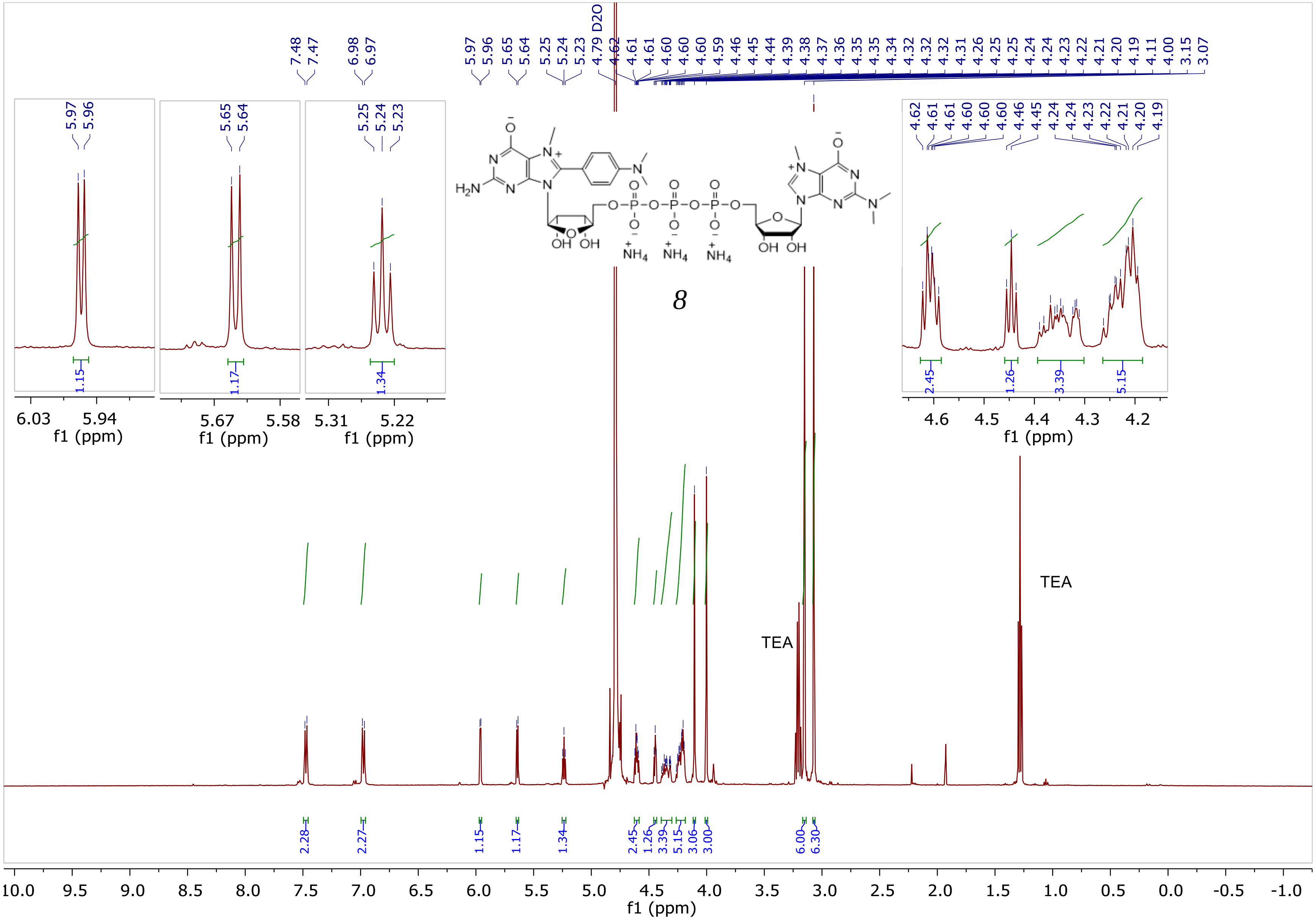

1H-1H COSY NMR

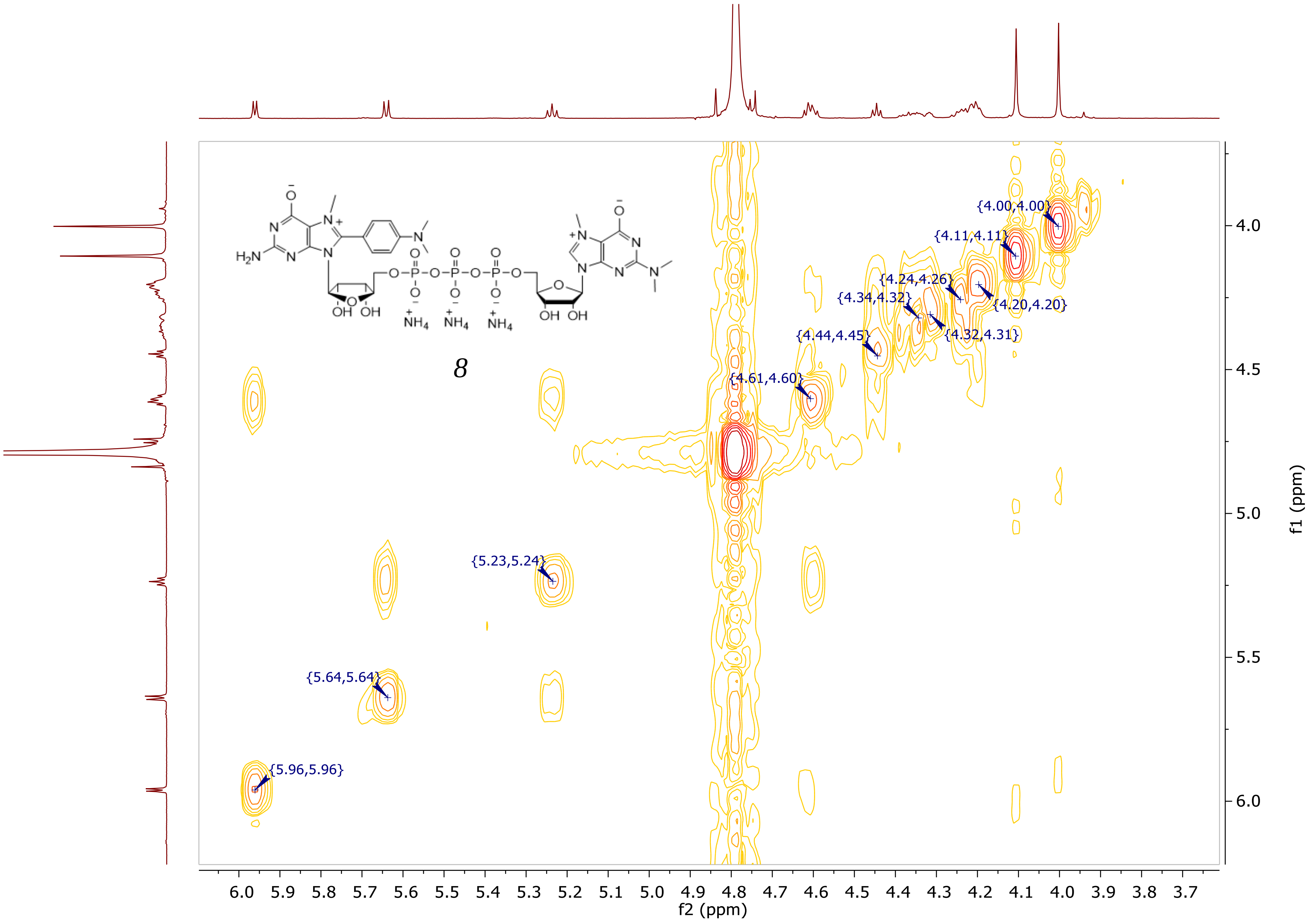

31P NMR

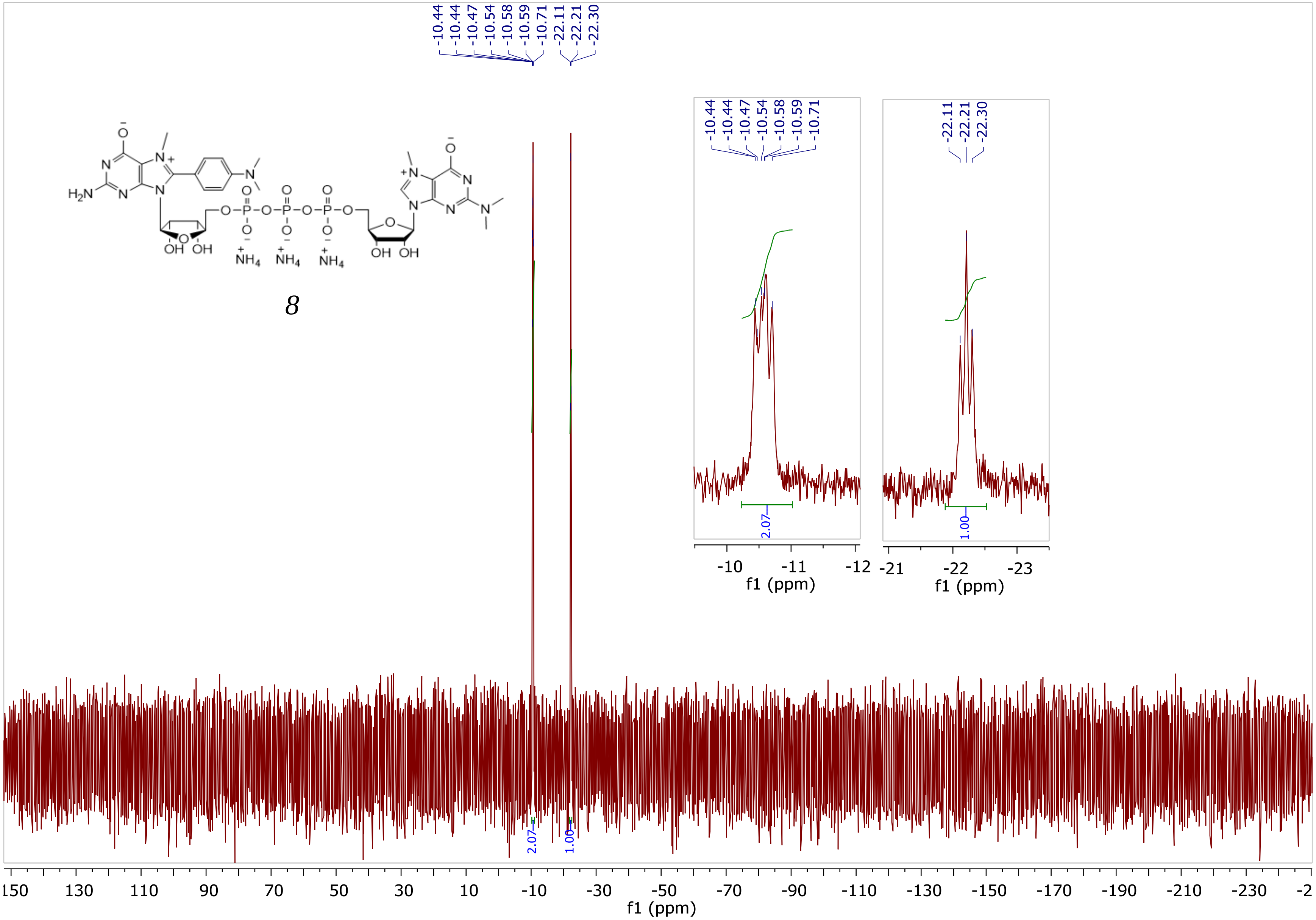

-10.44
-10.44
-10.47
-10.54
-10.58
-10.59
-10.71
-22.11
-22.21
-22.30
2.07
1.00
f1 (ppm)

8

HRMS

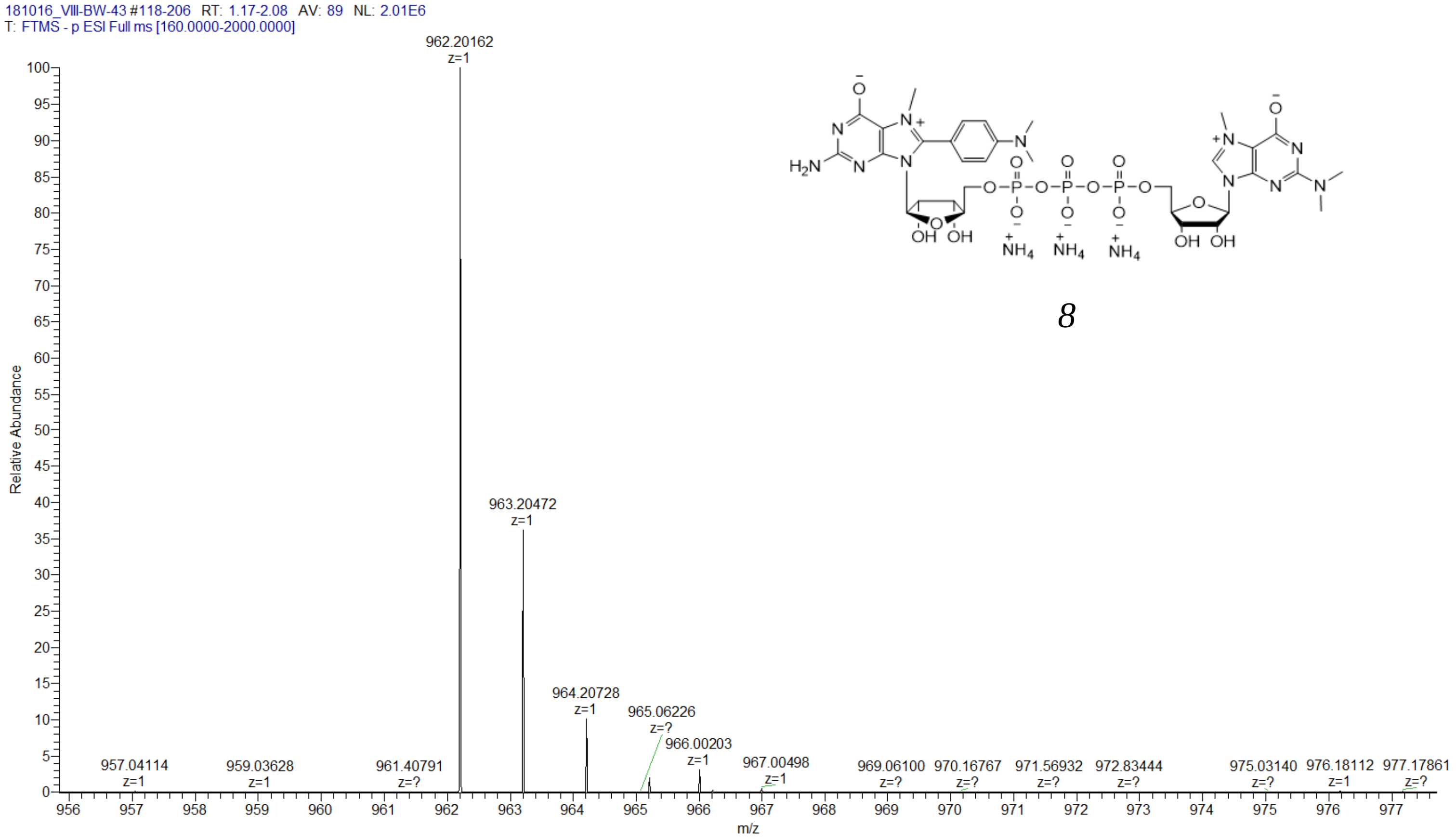

8

Summary

Rt (A) = 13.45 min; ^{1}H NMR (500 MHz, deuterium oxide) δ 7.47 (d, *J* = 8.6 Hz, 2H), 6.98 (d, *J* = 8.6 Hz, 2H), 5.96 (d, *J* = 4.1 Hz, 1H), 5.64 (d, *J* = 5.6 Hz, 1H), 5.24 (t, *J* = 5.6 Hz, 1H), 4.63-4.59 (m, 2H), 4.45 (t, *J* = 4.7 Hz, 1H), 4.35 (m, 3H), 4.22 (m, 3H), 4.11 (s, 3H), 4.00 (s, 3H), 3.15 (s, 6H), 3.07 (s, 6H); ^{31}P NMR (202 MHz, deuterium oxide) δ –10.44 to –10.71 (m, 2P), –22.21 (t, *J* = 19.1 Hz, 1P); HRMS ESI (-) m/z [M-H]$^-$, calcd for $C_{32}H_{43}N_{11}O_{18}P_4^-$ [M-H]$^-$ 962.2006; found 962.2016.
